# The Effects of Graded Levels of Calorie Restriction: X. Transcriptomic Responses of Epididymal Adipose Tissue

**DOI:** 10.1093/gerona/glx101

**Published:** 2017-05-27

**Authors:** Davina Derous, Sharon E Mitchell, Cara L Green, Yingchun Wang, Jing Dong J Han, Luonan Chen, Daniel E L Promislow, David Lusseau, Alex Douglas, John R Speakman

**Affiliations:** 1 Institute of Biological and Environmental Sciences, University of Aberdeen, UK; 2 Centre for Genome Enabled Biology and Medicine, University of Aberdeen, UK; 3 State Key laboratory of Molecular Developmental Biology, Institute of Genetics and Developmental Biology, Chinese Academy of Sciences, Chaoyang, Beijing, China; 4 Chinese Academy of Sciences Key Laboratory of Computational Biology, Chinese Academy of Sciences, Max Planck Partner Institute for Computational Biology, Shanghai Institutes for Biological Sciences, Chinese Academy of Sciences, China; 5 Key laboratory of Systems Biology, Innovation Center for Cell Signalling Network, Institute of Biochemistry and Cell Biology, Shanghai Institute of Biological Sciences, Chinese Academy of Sciences, China; 6 Department of Pathology, University of Washington, Seattle; 7 Department of Biology, University of Washington, Seattle

**Keywords:** Biology of aging, Caloric restriction, Genetics

## Abstract

Calorie restriction (CR) leads to a remarkable decrease in adipose tissue mass and increases longevity in many taxa. Since the discovery of leptin, the secretory abilities of adipose tissue have gained prominence in the responses to CR. We quantified transcripts of epididymal white adipose tissue of male C57BL/6 mice exposed to graded levels of CR (0–40% CR) for 3 months. The numbers of differentially expressed genes (DEGs) involved in NF-κB, HIF1-α, and p53 signaling increased with increasing levels of CR. These pathways were all significantly downregulated at 40% CR relative to 12 h *ad libitum* feeding. In addition, graded CR had a substantial impact on DEGs associated with pathways involved in angiogenesis. Of the 497 genes differentially expressed with graded CR, 155 of these genes included a signal peptide motif. These putative signaling proteins were involved in the response to ketones, TGF-β signaling, negative regulation of insulin secretion, and inflammation. This accords with the previously established effects of graded CR on glucose homeostasis in the same mice. Overall these data suggest reduced levels of adipose tissue under CR may contribute to the protective impact of CR in multiple ways linked to changes in a large population of secreted proteins.

## Introduction

Calorie restriction (CR) without malnutrition leads to a large decrease in adipose tissue mass compared to controls (1) and increases longevity in many taxa (2). CR induces an important shift towards β-oxidation of fatty acids (3) and as the main energy storage, adipose tissue is preferentially utilized during CR compared to the other organs (4). In addition to its energy storage function, adipose tissue produces many cytokines (also known as adipokines) such as leptin, tumor necrosis factor-alpha (TNF-α), and interleukin 6 (IL6) (5). An increase in adipose tissue mass is observed during aging, in both rats and humans, where it is associated with the metabolic syndrome (6). Increased adiposity is linked to chronic low-grade inflammation and circulating pro-inflammatory cytokines are increased in the obese state (7). CR results in both an improved inflammatory phenotype and improved adipokine signature related to metabolic disorders (8). In addition, circulating levels of several important inflammatory adipokines are decreased under CR (9). Hence decreased fat mass may influence health span and contribute to longevity by reducing adipokine production and improving inflammatory status.

Supporting this viewpoint, mice lacking the insulin receptor in fat tissue (FIRKO) were leaner, protected from age-related obesity and lived longer (10). Furthermore, surgical removal of visceral fat in long-lived growth hormone receptor knockout (GHRKO) mice showed that the secretory activity was necessary to promote longevity by decreased levels of inflammatory cytokines and increased insulin sensitivity (11). Interestingly, removal of visceral adipose tissue in rats led to an increase in mean and maximum lifespan, which was even more pronounced in CR exposed rats compared to *ad libitum* (AL) fed rats (12).

Other data, however, does not support the hypothesis that reduced adiposity is causally linked to elevated longevity. For example, long lived dwarf Ames and Snell mice have a relatively high percentage of fat (13,14). Inbred male DBA mice under CR have only a modest increase in lifespan yet these mice lost more mass and fat mass under CR compared to C57BL/6 mice (15). Other studies have also shown the greater weight loss of DBA mice under CR compared to C57BL/6 mice but longevity was not measured (16,17). Contradictory results come from a lifelong 40% CR study where CR restricted DBA mice had a higher survival rate under CR compared to DBA mice fed AL but compared to the restricted C57BL/6 mice they lived shorter lives (18). When 41 recombinant inbred strains of mice, were exposed to lifelong 40% CR, a reversed correlation was observed between fat reduction and CR-increased lifespan (19,20). However, it should be noted that the adverse relationship between fat loss and life expectancy might be due to the extreme depletion of subcutaneous fat stores in some strains. The visceral fats of dwarf models have been shown to adopt the characteristics of subcutaneous fat. Inhibited expansion of this type of adipose tissue leads to normalization of metabolic parameters despite morbid obesity (21). Hence it is believed that subcutaneous fat is able to expand without inducing hypoxia and inflammation, thus playing a more protective role when excess nutrients are available (21).

The extent to which calorie intake is restricted affects the extent to which lifespan is increased (22). The use of graded levels of CR as a research tool has gained much prominence in recent years (23–25) and we previously subjected individual mice at different levels of CR to an unprecedented level of phenotyping, summarized in a series of previous papers (4,9,26–30). Here we analyze the transcriptomic response of epididymal white adipose tissue to increasing levels of CR of the same individual mice. Adipose tissue secretes many signaling proteins, which may play important roles in mediating the impacts of CR both centrally and peripherally. We therefore used the transcriptomic data to identify the profile of potential secreted signaling proteins. This allowed us to tie together the changes in adipose tissue transcriptome and the phenotype associated with graded CR. 

## Results

### Identifying Differentially Expressed Genes and Enriched Pathways

**Figure 1.**
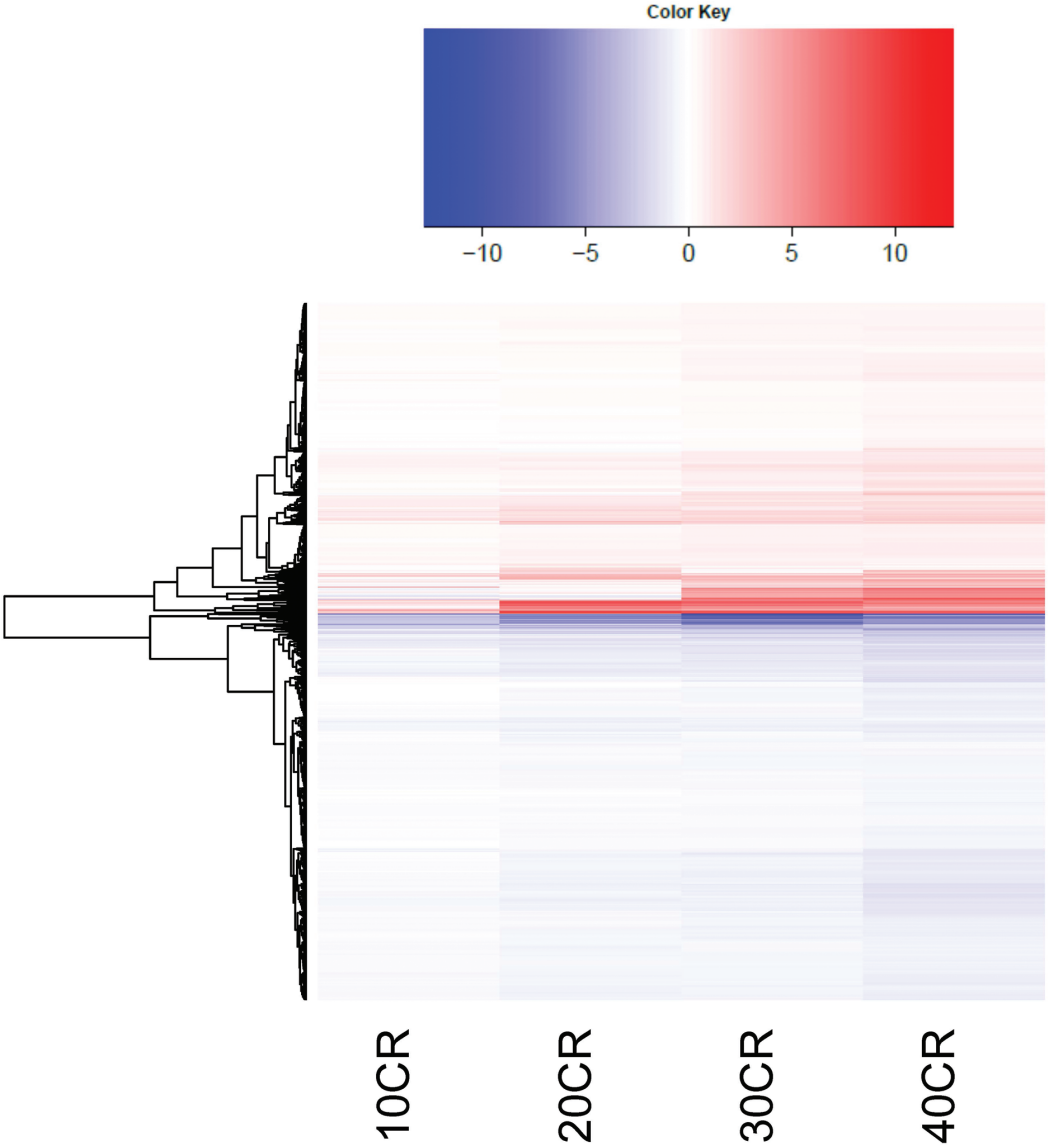
Differentially expressed genes (DEGs) at each level of restriction relative to 12 h *ad libitum* (AL). The heat map represents the 3435 genes that were significantly expressed relative to 12AL. Red indicates a positive log_2_ fold change (log FC) and blue a negative log FC. 10CR, 20CR, 30CR, and 40CR refer to 10, 20, 30, and 40% restriction.

We performed a 3-month graded CR study with four levels of increasing restriction (10%, 20%, 30%, 40%). These treatment groups are referred to as 10CR, 20CR, 30CR, and 40CR, respectively. In addition, we fed animals *ad libitum* for either 24 (24AL) or 12 h per day (12AL)—see methods for rationale of this design. The primary point of reference for the present analysis was 12AL. We have included in supplementary results how selection of 12AL or 24AL as the reference group affected the outcomes (Supplementary Figures 1 and 2). Differential gene expression analysis relative to 12AL was performed based on the Benjamini–Hochberg adjusted *p*-value (FDR < 0.05) and in total 3435 genes were significantly differently expressed in at least one of the CR levels. The number of DEGs relative to 12AL in adipose tissue increased in relation to CR level (i.e., for 10CR, 20CR, 30CR, and 40CR were 19, 733, 1323, and 3234 DEGs, respectively). The expression levels of individual genes did generally show a graded response to the increasing levels of CR (Figure 1).

**Figure 2.**
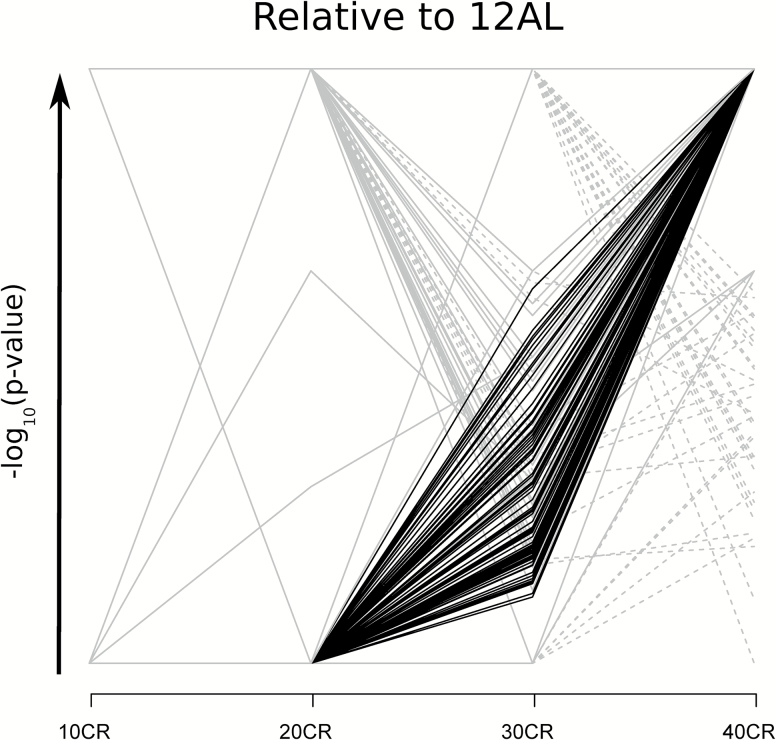
Pathways recruited with increasing CR level identified by Ingenuity Pathway Analysis (IPA, www.qiagen.com/ingenuity). Plot visualizing the increase in significance of pathways with increasing CR level relative to 12AL (*n* = 287). The Y-axis represents the –log10(*p*-value) of the pathway, with a maximum value of less than 0.05. A solid line indicates pathways that were significantly altered at 40CR (*p* < .05) and a dashed line represents pathways that were not significantly altered at 40CR (*p* > .05) relative to 12AL. The 88 pathways whose significance increased with graded CR by the color black and those who did not with the color grey. 10CR, 20CR, 30CR, and 40CR refer to 10, 20, 30, and 40% restriction.

**Figure 3.**
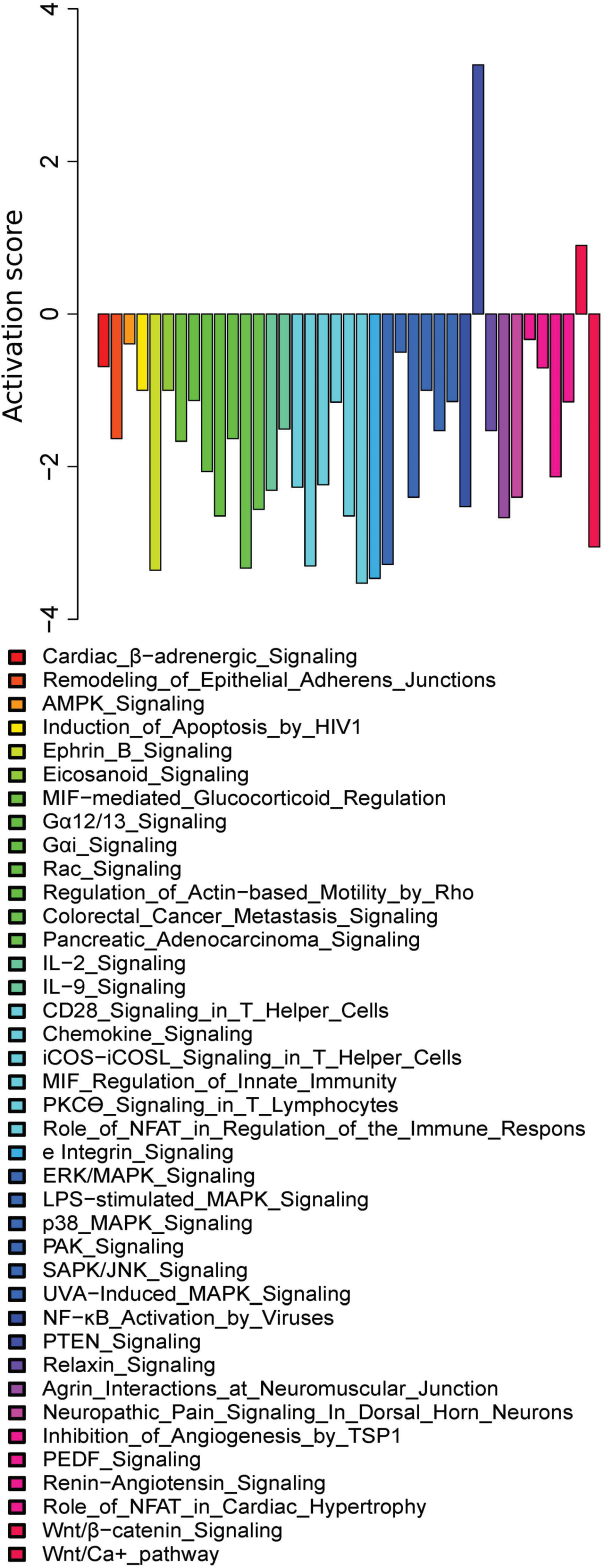
Pathways that increased their significance with increasing CR and were significant at 40CR relative to 12AL (*n* = 88). Bars represent the predicted activation scores identified by Ingenuity Pathway Analysis (IPA, www.qiagen.com/ingenuity).

Based on the DEGs relative to 12AL, pathways were identified at each level of CR by Ingenuity Pathway Analysis (IPA) analysis. There was an increase in the number of significantly altered pathways with the level of CR (i.e., for 10CR, 20CR, 30CR, and 40CR: 4, 90, 106, and 239 pathways were altered, respectively). Some genes were involved in multiple pathways (Supplementary Tables 1–4). If the number of DEGs within a pathway increases, the significance of that pathway in the IPA analysis increases. We observed an increase in pathway significance with increasing CR level which was most prominent between 20CR and 40CR (Figure 2). A total of 88 pathways were significantly altered at 40CR compared to 12AL, and progressively recruited more and more DEGs with increasing CR. In contrast, 54 pathways that were significantly altered at either 20CR or 30CR, had a reduced number of DEGs at 40CR (summarized in Supplementary Table 5, classification of pathways in Supplementary Table 6). To further elaborate on this “pathway recruitment” (i.e., increase in DEGs within a pathway) with increasing CR, we found that a total of 37 pathways were downregulated at 40CR and 2 were upregulated based on the gene expression relative to 12AL (Figure 3). Nine of the 54 pathways that were either significantly altered at 20CR or 30CR were downregulated relative to 12AL (Supplementary Figure 3).

### Identifying the Similarities Between the Different Levels of CR Relative to 12AL

**Figure 4.**
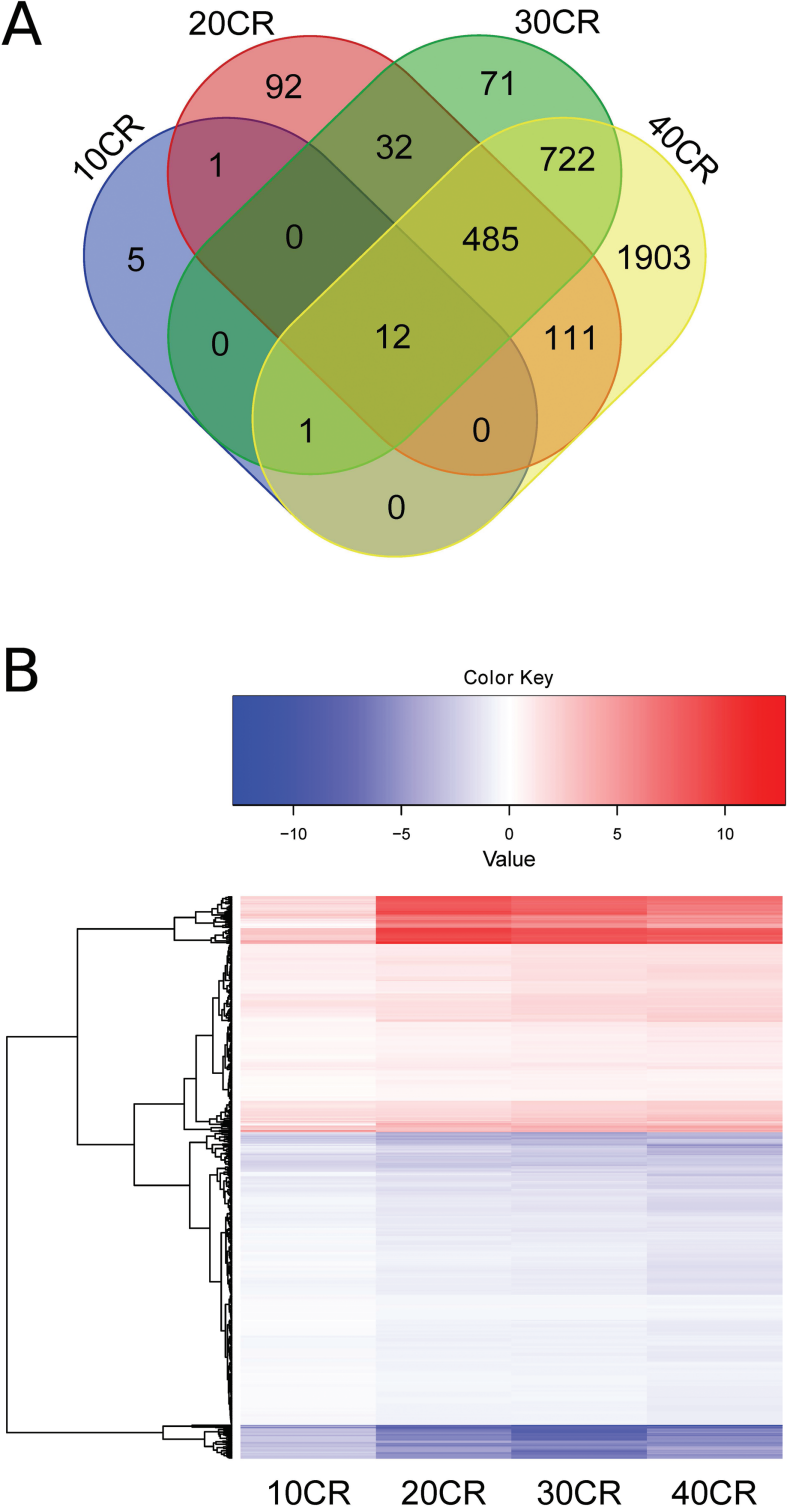
Differentially expressed genes (DEGs) in relation to the level of CR. (**A**) Venn diagram to represent DEGs mutually expressed at the different CR levels relative to 12 hours *ad libitum* feeding (12AL). (**B**) DEGs for each level of CR relative to 12AL. (*n* = 497). The heat map represents the log_2_ fold changes (log FC) at each CR level relative to 12AL. Red indicates a positive log FC and blue a negative log FC. 10CR, 20CR, 30CR, and 40CR refer to 10, 20, 30, and 40% restriction.

Different pathways were altered at the different CR levels, but for other genes CR induced the same DEGs and same enriched pathways across all CR levels relative to 12AL. We further characterized these similarities and found 12 genes differentially expressed at all CR levels (Figure 4A), and 485 DEGs at 20CR, 30CR, and 40CR (Figure 4B, Supplementary Table 7) resulting in a total of the same 497 DEGs altered across the levels of CR. At a pathway level, we found 36 pathways significantly enriched at 20CR, 30CR, and 40CR (Supplementary Table 8). We therefore extended our analysis and correlated the expression level of genes involved in these 36 pathways with the extent of restriction. Genes involved in the acute phase response signaling pathway correlated negatively with the increase in CR (Supplementary Figure 4). The pathways associated with inflammation and oxidative stress (Supplementary Figure 5), vascular biology (Supplementary Figure 6) and retinoid X receptor (RXR) associated pathways (Supplementary Figure 7) were also negatively correlated with the increase of CR.

### Identifying Potentially Encoded Proteins with a Signal Peptide

**Figure 5.**
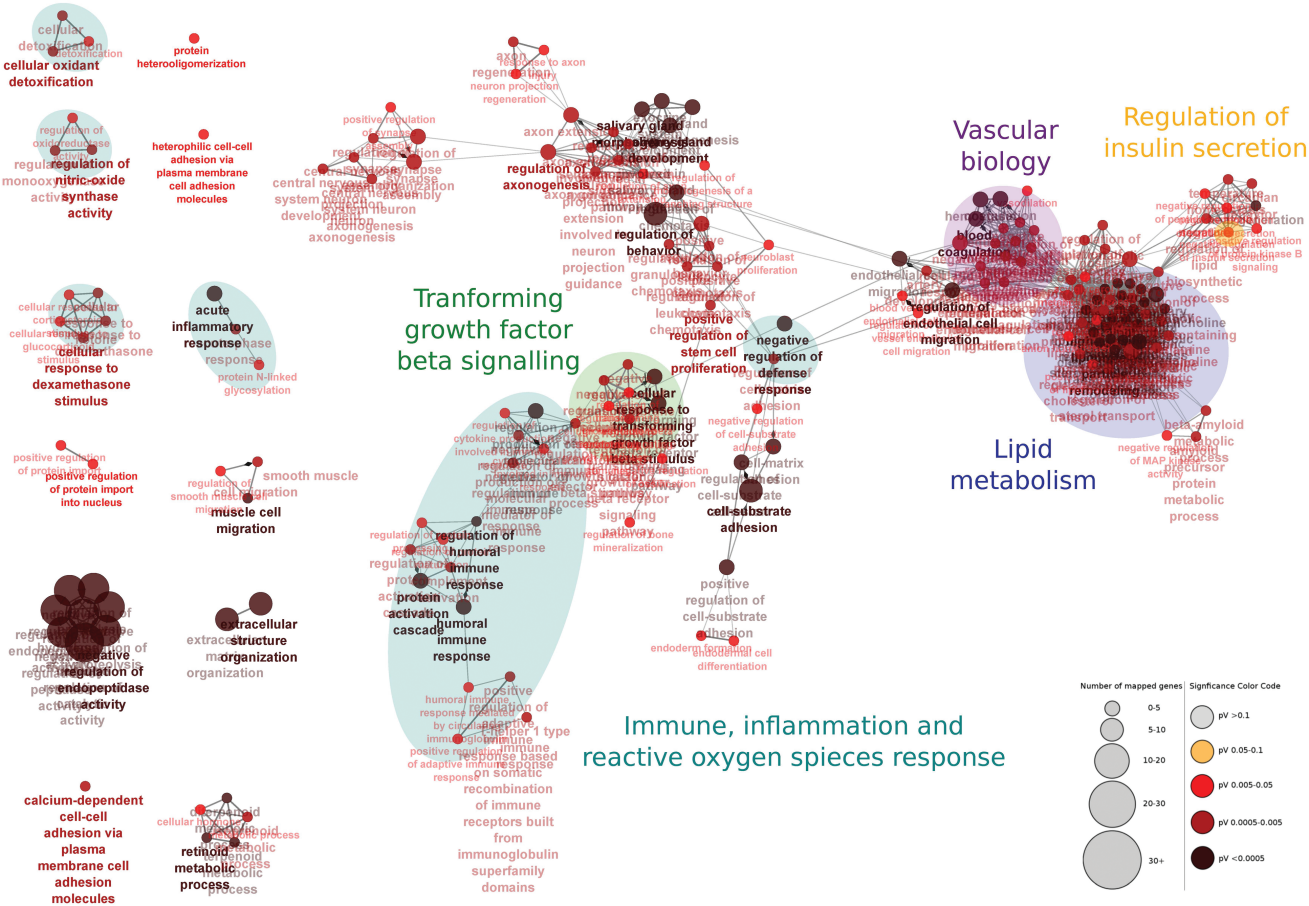
Statistically overrepresented Gene Ontology GO (GO) biological processes identified for the 155 encoded proteins with a signal peptide using ClueGO v2.2.3 in Cytoscape v3.2.1 (32). The network is comprised of nodes presenting significantly overrepresented GO biological processes (*p* < .05) connected via edges representing overlap between target genes in the overrepresented processes. The colors of the nodes represent the significance of the overrepresentation based on the *p*‐value and the size of the nodes represents the number of target genes. To aid visual interpretation, related biological processes were manually grouped in clusters indicated by the colored circles. These included vascular biology (purple), regulation of insulin secretion (orange), lipid metabolism (dark blue), transforming growth factor-beta signaling (green) and immune, inflammation, and reactive oxygen species response (light blue).

Adipose tissue is a secretory organ and therefore we assessed whether the 497 significantly expressed genes relative to 12AL with graded CR could potentially encode proteins with a signal peptide by using their predicted amino acid sequence identified using SignalP v4.1 (31). We identified 155 of the 497 genes that could encode signal proteins (Supplementary Table 9). To gain further insight, statistically overrepresented Gene Ontology (GO) biological processes were identified for these 155 genes using the tool ClueGO v2.2.3 in the Cytoscape program v3.2.1 (32). The resulting network is comprised of nodes presenting GO biological processes connected via edges representing overlap between target genes in the overrepresented processes (Figure 5, Supplementary Table 10). To aid visual interpretation, related biological processes were manually grouped in clusters.

### Identifying the Association Between Encoded Proteins with a Signal Peptide and Physiological Measurements

**Figure 6.**
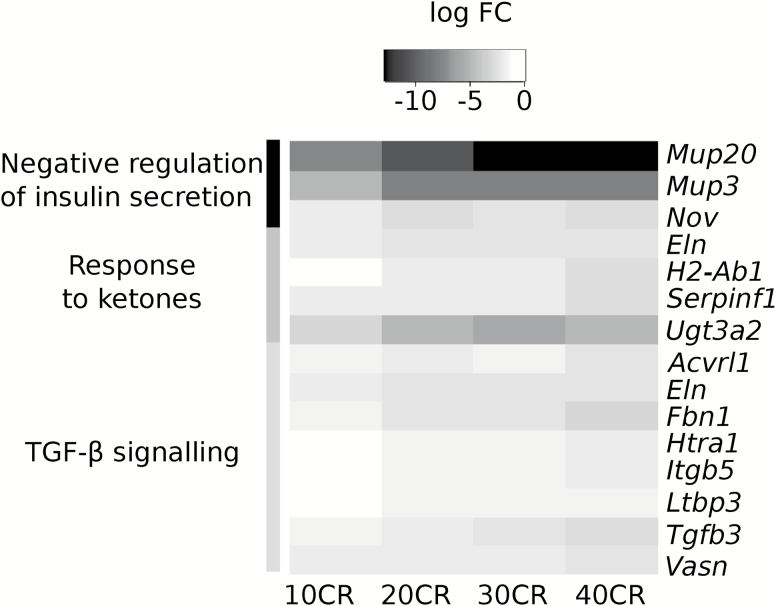
Expression levels of genes involved in insulin secretion, response to ketones, and TGF-β signaling relative to 12 h *ad libitum* intake (12AL). The heat map represents the log_2_ fold changes (log FC) at each CR level relative to 12AL. The intensity of the color black is related to the strength of the negative log FC. 10CR, 20CR, 30CR, and 40CR refer to 10, 20, 30, and 40% restriction.

**Figure 7.**
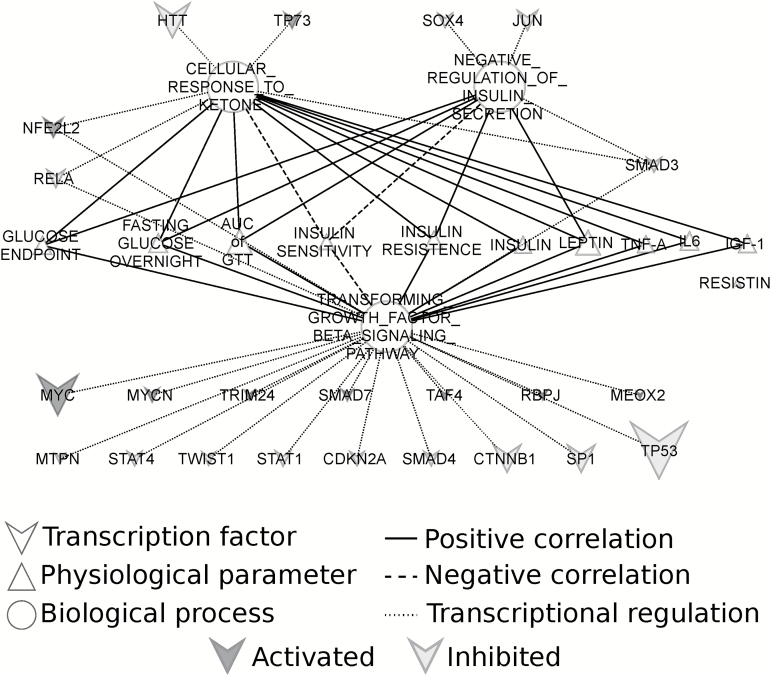
Overview of transcription factors, glucose homeostasis measurements and circulating hormones associated with signaling genes involved in insulin secretion, response to ketones and TGF-β signaling. The full lines represent a positive correlation between expression levels of genes involved in those pathways and physiological measurements. Negative correlations are represented by a dashed line and transcriptional regulation by a dotted line. The activation of transcription factors is represented by grey and the inhibition by black. The node size represents the number of target genes.

We previously measured several hormone levels and glucose homeostasis parameters in the same individual mice characterized here (9). Many of these hormones and parameters were involved in hunger regulation in the hypothalamus and insulin sensitivity (9,29). We therefore further elaborated on the signaling genes involved in insulin secretion, response to ketones, and TGF-β signaling. Genes in these processes showed a significant downregulation under CR relative to 12AL and this most prominent at 40CR (Figure 6). We correlated gene expression levels of each mouse with their corresponding circulating hormone levels and glucose homeostasis parameters (Figure 7). Genes involved in the negative regulation of insulin secretion correlated positively with glucose homeostasis measurements, with the exception of insulin sensitivity, which correlated negatively (Supplementary Table 11). Circulating levels of leptin correlated positively with the expression levels of genes involved in the negative regulation of insulin secretion (Supplementary Table 12). Genes involved in TGF-β signaling and response to ketones correlated with most of the circulating hormones we measured and glucose homeostasis measurements. None of the genes involved in insulin secretion, response to ketones and TGF-β signaling correlated with circulating levels of resistin.

We also identified transcription factors upstream from the genes containing the signal protein motif involved in these three biological processes (identified by IPA). The activation/inhibition state of the transcription factors was based on the expression of their downstream target genes. Of the 23 transcription factors, 19 were involved in regulating TGF-β signaling gene expression, 5 in regulating the response to ketones gene expression, and 3 in regulating expression levels of genes involved in the negative regulation of insulin secretion.

## Discussion

**Figure 8.**
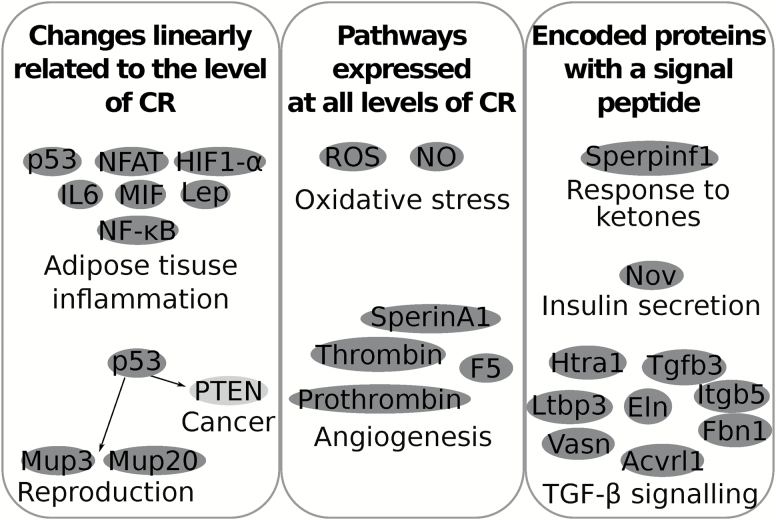
An integrated overview of the effects of graded calorie restriction on the adipose tissue transcriptome. The dark grey circles represent genes that were downregulated relative to 12AL and light grey represent an upregulation relative to 12AL. The genes are grouped according to their biological function. The three panels represent the results discussed in the three paragraphs included in the discussion.

In the same individual mice used here, we previously reported that CR induced a significant reduction in body mass and in particular adipose tissue mass (4), improved glucose homeostasis, reduced levels of several circulating hormones (9), reduced body temperature (26), a shift in behavior (27), an effect on physical activity (28), increased hunger signaling in the hypothalamus (29), lowered basal metabolic rate (33), and altered liver metabolome (34). Epididymal white adipose tissue was preferentially utilized during graded CR compared to the other organs (4) and provided 55.8–60.9% of the total released energy as the mice sought to mitigate the shortfall in their intake (4), consistent with other studies (35,36). Here we focused on the transcriptomic profile of epididymal white adipose tissue and we provided an integrated overview of the effects of graded CR (Figure 8).

Adipose tissue plays a central role in metabolism and secretion of hormones. During over-nutrition, rapid adipose tissue expansion induces a local hypoxia response with elevated ROS production and coincident inflammatory responses (7). This results in a reduction in the health of adipose tissue and consequently adversely affects metabolic processes, triggering metabolic dysfunctions at a physiological level (e.g., insulin resistance) (6). Adipose tissue levels also tend to increase with age and ROS production and chronic low-grade inflammation both contribute to dysfunctional adipose tissue associated with aging and may contribute to molecular differences between normal aging and age-related diseases (6). An important observation we made here is that many genes showed a non-graded transcriptional response to the CR treatment. That is a gene or pathway might be activated at 20CR or 30CR, but was not activated at 40CR. Because in this strain graded increases in CR leads to a graded increase in lifespan (37), such non-linear changes in gene expression with the level of restriction are unlikely to be key components driving the longevity response. Yet such changes would be identified as potentially important in protocols using only a single level of restriction. Importantly we did find several pathways where gene expression profiles were linearly related to the level of restriction, and we suggest these are more likely candidates contributing to the CR longevity response. In particular, we found that pathways involved in the molecular signaling of hypoxia such as activated nuclear factor-kappa B (NF-ĸB) and hypoxia-inducible factor-alpha (HIF1-α), were progressively repressed with increasing CR level and were significantly downregulated at 40CR relatively to 12AL, which has been observed previously (38). Furthermore, signaling elements associated with hypoxia such as leptin (*Lep*), interleukin 6 (IL6), macrophage migration inhibitory factor (MIF), tumor suppressor protein p53, and nuclear factor of activated T-cells (NFAT) were also downregulated at 40CR relative to 12AL. Together our results are consistent with a declining level of adipose tissue inflammation as CR increased potentially negating the ageing-associated decline in adipose tissue health, and associated age-related diseases. Recent work suggested that CR enhances a type 2 immune response, which leads to browning of adipose tissue (39). Fabbanio *et al.* hypothesized that beige fat was a common feature of a negative energy balance. However, we did not find a similar gene expression regulation of browning markers.

The gene p53 was first identified as a tumor suppressor and was found to be the most commonly mutated gene in cancers (40). In p53 knockout mice, 40% CR delayed spontaneous tumorigenesis compared to their wild-type siblings (41) suggesting that the protective role for CR against cancer is independent of p53. In addition, the PTEN signaling pathway was significantly increased at 40CR compared to 12AL. The PTEN gene is a tumor suppressor and can inhibit cellular proliferation (42). The anti-cancer effect of CR has been widely documented and is believed to reduce the initiation and progression of spontaneous tumors in several tissue (43). There is a significant association between excess body fat (i.e., obesity) and the risk of cancer occurrence. One of the mechanisms contributing to the increased risk of cancer in obese individuals is elevated inflammation (44). Hence the reduced adiposity with reduced inflammation as direct effect of CR could mediate some of its beneficial effects. Short-lived organisms such as *Caenorhabditis elegans* and *Drosophila melanogaster* do not develop adult cancers but still exhibit expression levels of p53 which suggest additional functions for this gene (45). Increased gene expression of p53 in adipose tissue inflammation can regulate insulin resistance (46) and downregulation of p53 at 40CR is concordant with the improved insulin sensitivity in these mice (9).

The p53 signaling pathway has also been implicated in playing a role in reproduction (47). During CR, a reallocation of energy investment between somatic maintenance, growth and reproduction has been proposed (48). This reallocation of energy investment has been hypothesized as an integral component of the increased lifespan under CR although others have reasoned against this (49). We found that 40CR significantly altered pathways related to germ cell development. Gene expression levels of major urinary proteins (MUPs) (*Mup20* and *Mup3*) were reduced in the adipose tissue. MUPs are used in scent marking for mate attraction and the decreased production would be consistent with a trade-off between reproductive investment and maintenance of the soma under CR (50). MUPs are primarily found in liver but mRNAs related to hepatic MUPs are also present in other secretory tissues (51). In urine we observed a reduction in MUPs of these mice as CR increased (9). In summary, the pathways that were progressively repressed with increasing CR levels such as NF-κB, HIF1-α and p53 serve important signaling functions in lifespan-associated pathways, and their reduced signaling as CR level increased may contribute to the CR mediated increase in lifespan.

A total of 36 pathways were significantly expressed at all levels of CR relative to 12AL and the gene expression levels changed in a graded manner in response to graded CR. These pathways were involved in ROS production, inflammation, and angiogenesis. Genes involved in the production of nitric oxide (NO) were negatively correlated with the extent of CR and downregulated relative to 12AL. NO is generated by various components of the immune system and is believed to be protective against cellular damage (52). Reduced oxidative stress mediated by CR can be achieved by several mechanisms including a decrease in the rate at which ROS are generated, an increase in the rate at which ROS are detoxified and an upregulation of degradation and repair processes (22). A possible interpretation of lower NO could be that less ROS were produced in the adipose tissue under CR compared to 12AL. Although ROS are necessary for cellular activities (53) and the immune system (54), they may induce cellular damage leading to diseases (55). ROS-induced inflammation can stimulate adipose tissue expansion and is associated with angiogenesis (56). The angiogenic activity of adipose tissue is a crucial step to provide oxygen, nutrition, and hormones (57) and effective development of intrinsic vascular biology is a rate-limiting step in adipose tissue expansion (58). During CR, adipose tissue contracts (1,36) and hence we might anticipate a corresponding reversal of angiogenic processes. This was consistent with the downregulated pathways involved in thrombin signaling under all levels of CR. Thrombin may play an important role in both normal and pathological vascular biology (59) and it stimulates secretion of inflammatory cytokines such as IL6 and TNF-α in human adipose tissue (60). In a study of intermittent fasting in humans, proteomic analysis of plasma after four weeks showed downregulation of prothrombin in the treatment cohort compared to controls (61). We also found a downregulation of coagulation factor V (*F5*), which participates as a cofactor in the conversion of prothrombin to thrombin (62). Furthermore, the gene *SerpinA1* or α1-antitrypsin was downregulated under CR and is a marker for inflammatory processes usually associated with atherogenesis and cardiovascular diseases (63). Interestingly α1-antitrypsin is also downregulated in long-lived Snell mice (64). Our results suggest that graded CR had a beneficial effect in adipose tissue by reducing intrinsic inflammation, ROS production and angiogenesis, leading to an overall “healthier” adipose tissue profile compared to mice fed AL and this in a graded fashion.

Fatty acids in the form of triglycerides are released by adipose tissue and can be converted into ketones in the liver to create an energy source via ketogenesis (65). In addition to an alternative energy source during fasting, ketone bodies can also serve as signaling molecules (66). Early work has shown that murine adipose tissue can utilize ketone bodies (67) and hence they may serve as a signaling metabolite between liver and adipose tissue. We found decreased transcription of genes encoding signaling proteins known to be responsive to ketones in adipose tissue (e.g., *Serpinf1*). Elevated levels of *Serpinf1* may serve as a regulator of metabolic syndrome (68) and prolonged administration leads to adipose tissue lipolysis and reduced insulin sensitivity. Expression levels of *Serpinf1* is transcriptionally regulated by huntingtin (*Htt*) (69) and there was inhibition of *Htt* in our CR study. This may have potentially contributed to the observed improved insulin sensitivity at higher levels of CR (9). Indeed, reduced expression levels of *Serpinf1* correlated with improved insulin sensitivity and reduced insulin resistance. In concordance with this result, we found several signaling proteins, glucose homeostasis measurements and circulating hormones associated with the negative regulation of insulin secretion. These included the gene *Nov* or also known as CCN3, circulating levels of leptin and plasma glucose levels. In mice, plasma levels of *Nov* are related to *Nov* gene expression in adipose tissue, and it is believed to contribute to obesity-related inflammation (70). CCN3 is a part of the CNN family and these proteins contain distinct structural modules resembling IGF binding proteins. CCN3 shows a weak affinity for IGF-binding and by specific interactions with this domain, it may be involved in similar signaling cascades (71). Expression of CCN3 was associated with a significant higher risk of developing lung and bone metastasis in Ewing’s sarcoma (72). In transfected CCN3-negative Edwing’s sarcoma cell line with CCN3, the cell proliferation was reduced but migration and invasion was increased (73). Hence, CCN3 plays an important role in cancer. Its downregulation under CR may contribute to the well-known anti-cancer effect of CR (43). In pancreatic β-cells, *Nov* was found to be a transcriptional target of Forkhead box protein O1 (FoxO1) and *Nov* inhibited glucose-stimulated Ca^2+^ entry and insulin secretion (74). Pancreatic β-cells are also sensitive to plasma glucose and leptin concentrations and secrete insulin accordingly (75,76). Plasma glucose levels and insulin at the end of study were both reduced under CR (9). Downregulation of the evolutionary conserved IGF-1/insulin signaling pathway is associated with increase in lifespan in worms, flies, and rodents (77–79). In *C. elegans*, TGF-β may be tightly linked to the insulin/IGF-1 pathway and hence play a role in regulating longevity (80). Expression profiles of adult long-lived TGF-β mutants overlapped significantly with insulin/IGF-1 pathway profiles, including those genes that regulate life span (81). TGF-β is released by adipocytes and both the mRNA and protein levels of TGF-β are increased in adipose tissue of genetically obese (ob/ob) mice compared to lean mice (82). In addition TNF-α is known to promote insulin resistance and stimulate TGF-β gene expression (82,83). In our study, signaling proteins involved in the TGF-β signaling pathway were downregulated under CR. Furthermore, decreased TGF-β signaling was correlated with measures of improved insulin action under CR. This is in agreement with reduced levels of circulating TNF-α under CR and its correlation with expression levels of genes involved in TGF-β signaling (9). As both of these pathways are evolutionarily conserved, the reduced signaling by TGF-β and insulin may possibly play a role in the longevity of higher organisms, such as mammals.

In most rats and mice increasing levels of CR in both sexes are linearly related to the increase in lifespan. Hence the use of graded levels of CR as a research tool has gained much prominence in recent years (23–25). Recently, 41 strains of recombinant inbred mice were exposed to two levels of CR: 20% and 40% CR. The responses of these strains to CR elucidated a clear role of sex, strain and the level of CR with decreased lifespan at 40% CR for some strains (84). It was proposed that the CR responses inducing longevity may vary according to genotype. Hence our study has some limitations as we only studied one strains. However, C57BL/6 mice are known responders to CR, are a well-studied strain and were the subject of the mouse genome project. Hence there are abundant data for this strain in various other fields and hence the changes we found in the transcriptome and other responses of these mice can be more easily set into a wider context. A recent review suggested that there was no strong sex effect on the impact of CR on lifespan (37). However by using male mice were able to measure their reproductive investment by determining production of urinary MUPs (9). In addition, there is a possibility that CR may change the relative proportions of cell types in the adipose tissue. Although we measured fat mass, we did not perform any histology of the tissue. Hence some of the changes we observed may not reflect changes in gene expression of adipocytes, but instead the changing proportions of different cell types in the tissue. Overall our data suggest reduced levels of adipose tissue under CR may contribute to the protective impact of CR in multiple ways linked to changes in tissue inflammation, oxidative stress and vascular biology.

## Methods

### Animals and Experimental Manipulations

All procedures were approved by the University of Aberdeen ethical approval committee and carried out under the Animals (Scientific Procedures) Act 1986 Home Office license (PPL 60/4366 held by JRS). Forty-nine male C57BL/6 mice (*Mus musculus*) purchased from Charles River (Ormiston, UK) were individually housed and free access to water was provided. Mice were exposed to 12 h dark/light cycle (lights on at 06:30 h) and body mass and food intake were recorded daily, immediately prior to nocturnal feeding. At 20 weeks of age (resembling early adulthood in humans), mice were randomly allocated into 6 different treatment groups: 24 h *ad libitum* intake (24AL) (*n* = 8), 12AL intake (*n* = 8), 10 CR (*n* = 8), 20CR (*n* = 8), 30CR (*n* = 8), and 40CR (*n* = 9). Mice in the 12AL group were fed *ad libitum* for 12 h during the dark period. 40CR indicates 40% lower calories than their own individual intakes measured over a baseline period of 14 days prior to introducing CR. This is a caloric restriction protocol rather than a caloric dilution experiment (37).

CR-restricted mice generally consume their entire daily ration of food during the first few hours after the food is provided. The 24AL animals can by definition eat at any time throughout a 24 h period. Hence, when CR-restricted mice were culled they may have been starving for 10–16 h while 24AL may have eaten an hour prior to culling. To address this issue, 12AL was set as a reference to avoid the “time since last meal effect” and graded levels of CR were introduced to investigate a potential graded response. Information on overall study design, diet composition, and detailed rationale are described elsewhere (4).

### RNA Isolation, RNA Sequencing, Alignment and Analytical Procedure

After culling by a terminal CO_2_ overdose, epididymal white adipose tissue was removed, weighed and frozen in liquid nitrogen and stored at −80°C until RNA extraction. RNA was isolated by homogenizing in Tri-Reagent (Sigma Aldrich, UK) according to the manufacturer’s instructions. Prior to RNA quantification, using the Agilent RNA 6000 Nano Kit, samples were denatured at 65°C. In total, the RNA of 43 individual mice (12 h AL *n* = 7, 24 h AL *n* = 7, 10% CR *n* = 7, 20% CR *n* = 8, 30% CR *n* = 7, 40% CR *n* = 7) was successfully isolated and sent to Beijing Genomic Institute (BGI, Hong Kong) for RNA sequencing. Library preparation was done according to standard protocol of BGI and the library products were sequenced using an Illumina Hi-seq 2000, resulting in 50 bp single end reads. Standard primers and barcodes developed by BGI were used. Detailed information on library preparation and alignment has been described previously (29). Differential gene expression was modeled using the edgeR package (85) in R (version 3.1.2) (86) and pairwise comparisons were conducted between 12AL or 24AL and each level of CR. To control for type I errors, the Benjamini Hochberg adjusted *p*-value was used (5% FDR) (87).

### Biological Interpretation

Enriched pathways were identified based on DEGs using the option core analysis in the IPA program (version 2000–2016, Ingenuity Systems, www.ingenuity.com).

DEGs at all levels of CR (*n* = 12) and at 20CR, 30CR, and 40CR (*n* = 486) relative to 12AL were further analyzed to determine if they encoded a signal protein. Their amino acid sequence was obtained from the National Center for Biotechnology Information website (NCBI, http://www.ncbi.nlm.nih.gov/) and potential signal peptides were identified by using the Center for Biological Sequence analysis (CBS) prediction server SignalP 4.1 (http://www.cbs.dtu.dk/services/SignalP/) (31). Only genes identified to encode a signal protein were considered for further analysis (*n* = 155). The ClueGO v2.2.3 plugin within Cytoscape v3.2.1 (32) was used to determine which biological processes (Gene Ontology biological processes v29.01.2016) were overrepresented based on these 155 genes. The overrepresentation was based on a two-sided hypergeometric test with a Benjamini–Hochberg FDR cut-off of 0.05 and a minimum of 3 genes in the pathway selection. Normalized counts of these genes were correlated with physiological measurements [obtained from (9)] and transcription factors were obtained from the upstream analysis in the IPA program.

## Supplementary Material

Supplementary data are available at *The Journals of Gerontology Series A: Biological Sciences and Medical Sciences* online.

## Funding

The work was supported by the UK Biotechnology and Biological Sciences Research Council BBSRC (grants BB/G009953/1 and BB/J020028/1 to JRS and SEM) and a studentship of DD supported by the Centre for Genome Enabled Biology and Medicine, Aberdeen, UK. DL was supported by Office of Naval Research (ONR) grant N000141512377.

## Conflict of Interest

None declared.

## Supplementary Material

Supplementary_Information

## References

[1] Gelegen C, Collier DA, Campbell IC, Oppelaar H, Kas MJ Behavioral, physiological, and molecular differences in response to dietary restriction in three inbred mouse strains. Am J Physiol Endocrinol Metab. 2006;291:E574–E581. doi:10.1152/ajpendo.00068.2006 16670152 10.1152/ajpendo.00068.2006

[2] Fontana L, Klein S, Holloszy JO Effects of long-term calorie restriction and endurance exercise on glucose tolerance, insulin action, and adipokine production. Age (Dordr). 2010;32:97–108. doi:10.1007/s11357-009-9118-z 19904628 10.1007/s11357-009-9118-z PMC2829643

[3] Hagopian K, Ramsey JJ, Weindruch R Krebs cycle enzymes from livers of old mice are differentially regulated by caloric restriction. Exp Gerontol. 2004;39:1145–1154. doi:10.1016/j.exger.2004.04.009 15288689 10.1016/j.exger.2004.04.009

[4] Mitchell SE, Tang Z, Kerbois C The effects of graded levels of calorie restriction: I. impact of short term calorie and protein restriction on body composition in the C57BL/6 mouse. Oncotarget. 2015;6:15902–15930. doi:10.18632/oncotarget.4142 26079539 10.18632/oncotarget.4142 PMC4599246

[5] Trayhurn P, Beattie JH Physiological role of adipose tissue: white adipose tissue as an endocrine and secretory organ. Proc Nutr Soc. 2007;60:329–339. doi:10.1079/PNS200194 10.1079/pns200194 11681807

[6] Pérez LM, Pareja-Galeano H, Sanchis-Gomar F, Emanuele E, Lucia A, Gálvez BG “Adipaging”: ageing and obesity share biological hallmarks related to a dysfunctional adipose tissue. J Physiol. 2016;594:3187–3207. doi:10.1113/JP271691 26926488 10.1113/JP271691 PMC4908019

[7] Trayhurn P, Wood IS Signalling role of adipose tissue: adipokines and inflammation in obesity. Biochem Soc Trans. 2005;33(Pt 5):1078–1081. doi:10.1042/BST20051078 16246049 10.1042/BST0331078

[8] Renes J, Rosenow A, Roumans N, Noben JP, Mariman EC Calorie restriction-induced changes in the secretome of human adipocytes, comparison with resveratrol-induced secretome effects. Biochim Biophys Acta. 2014;1844:1511–1522. doi:10.1016/j.bbapap.2014.04.023 24802182 10.1016/j.bbapap.2014.04.023

[9] Mitchell SE, Delville C, Konstantopedos P The effects of graded levels of calorie restriction: II. Impact of short term calorie and protein restriction on circulating hormone levels, glucose homeostasis and oxidative stress in male C57BL/6 mice. Oncotarget. 2015;6:23213–23237. doi:10.18632/oncotarget.4003 26061745 10.18632/oncotarget.4003 PMC4695113

[10] Blüher M, Kahn BB, Kahn CR Extended longevity in mice lacking the insulin receptor in adipose tissue. Science. 2003;299:572–574. doi:10.1126/science.1078223 12543978 10.1126/science.1078223

[11] Masternak MM, Bartke A, Wang F Metabolic effects of intra-abdominal fat in GHRKO mice. Aging Cell. 2012;11:73–81. doi:10.1111/j.1474-9726.2011.00763.x 22040032 10.1111/j.1474-9726.2011.00763.x PMC3257405

[12] Muzumdar R, Allison DB, Huffman DM Visceral adipose tissue modulates mammalian longevity. Aging Cell. 2008;7:438–440. doi:10.1111/j.1474-9726.2008.00391.x 18363902 10.1111/j.1474-9726.2008.00391.x PMC2504027

[13] Heiman ML, Tinsley FC, Mattison JA, Hauck S, Bartke A Body composition of prolactin-, growth hormone, and thyrotropin-deficient Ames dwarf mice. Endocrine. 2003;20:149–154. doi:10.1385/ENDO:20:1-2:149 12668880 10.1385/ENDO:20:1-2:149

[14] Berryman DE, List EO, Palmer AJ Two-year body composition analyses of long-lived GHR null mice. J Gerontol A Biol Sci Med Sci. 2010;65:31–40. doi:10.1093/gerona/glp175 19901018 10.1093/gerona/glp175 PMC2796884

[15] Swindell WR Dietary restriction in rats and mice: a meta-analysis and review of the evidence for genotype-dependent effects on lifespan. Ageing Res Rev. 2012;11:254–270. doi:10.1016/j.arr.2011.12.006 22210149 10.1016/j.arr.2011.12.006 PMC3299887

[16] Ferguson M, Sohal BH, Forster MJ, Sohal RS Effect of long-term caloric restriction on oxygen consumption and body temperature in two different strains of mice. Mech Ageing Dev. 2007;128:539–545. doi:10.1016/j.mad.2007.07.005 17822741 10.1016/j.mad.2007.07.005 PMC2048713

[17] Hempenstall S, Picchio L, Mitchell SE, Speakman JR, Selman C The impact of acute caloric restriction on the metabolic phenotype in male C57BL/6 and DBA/2 mice. Mech Ageing Dev. 2010;131:111–118. doi:10.1016/j.mad.2009.12.008 20064544 10.1016/j.mad.2009.12.008

[18] Turturro A, Witt WW, Lewis S, Hass BS, Lipman RD, Hart RW Growth curves and survival characteristics of the animals used in the biomarkers of aging Program. J Gerontol A Biol Sci Med Sci. 1999;54:B492–B501. doi:10.1093/gerona/54.11.B492 10619312 10.1093/gerona/54.11.b492

[19] Liao CY, Rikke BA, Johnson TE, Diaz V, Nelson JF Genetic variation in the murine lifespan response to dietary restriction: from life extension to life shortening. Aging Cell. 2010;9:92–95. doi:10.1111/j.1474-9726.2009.00533.x 19878144 10.1111/j.1474-9726.2009.00533.x PMC3476836

[20] Liao CY, Rikke BA, Johnson TE, Gelfond JA, Diaz V, Nelson JF Fat maintenance is a predictor of the murine lifespan response to dietary restriction. Aging Cell. 2011;10:629–639. doi:10.1111/j.1474-9726.2011.00702.x 21388497 10.1111/j.1474-9726.2011.00702.x PMC3685291

[21] Kim JY, van de Wall E, Laplante M Obesity-associated improvements in metabolic profile through expansion of adipose tissue. J Clin Invest. 2007;117:2621–2637. doi:10.1172/JCI31021 17717599 10.1172/JCI31021 PMC1950456

[22] Speakman JR, Mitchell SE Caloric restriction. Mol Aspects Med. 2011;32:159–221. doi:10.1016/j.mam.2011.07.001 21840335 10.1016/j.mam.2011.07.001

[23] Duffy PH, Lewis SM, Mayhugh MA The effects of different levels of dietary restriction on neoplastic pathology in the male Sprague-Dawley rat. Aging Clin Exp Res. 2004;16:448–456. doi:10.1007/BF03353422 15739595 10.1007/BF03327400

[24] Nogueira LM, Lavigne JA, Chandramouli GV, Lui H, Barrett JC, Hursting SD Dose-dependent effects of calorie restriction on gene expression, metabolism, and tumor progression are partially mediated by insulin-like growth factor-1. Cancer Med. 2012;1:275–288. doi:10.1002/cam4.23 23342276 10.1002/cam4.23 PMC3544443

[25] Kim SS, Choi KM, Kim S Whole-transcriptome analysis of mouse adipose tissue in response to short-term caloric restriction. Mol Genet Genomics. 2016;291:831–847. doi:10.1007/s00438-015-1150-3 26606930 10.1007/s00438-015-1150-3

[26] Mitchell SE, Delville C, Konstantopedos P The effects of graded levels of calorie restriction: III. Impact of short term calorie and protein restriction on mean daily body temperature and torpor use in the C57BL/6 mouse. Oncotarget. 2015;6:18314–18337. doi:10.18632/oncotarget.4506 26286956 10.18632/oncotarget.4506 PMC4621893

[27] Lusseau D, Mitchell SE, Barros C The effects of graded levels of calorie restriction: IV. Non-linear change in behavioural phenotype of mice in response to short-term calorie restriction. Sci Rep. 2015;5:13198. doi:10.1038/srep13198 26306002 10.1038/srep13198 PMC4548231

[28] Mitchell SE, Delville C, Konstantopedos P The effects of graded levels of calorie restriction: V. Impact of short term calorie and protein restriction on physical activity in the C57BL/6 mouse. Oncotarget. 2016;7:19147–19170. doi:10.18632/oncotarget.8158 27007156 10.18632/oncotarget.8158 PMC4991372

[29] Derous D, Mitchell SE, Green CL The effects of graded levels of calorie restriction: VI. Impact of short-term graded calorie restriction on transcriptomic responses of the hypothalamic hunger and circadian signaling pathways. Aging (Albany NY). 2016;8:642–663. doi:10.18632/aging.100895 26945906 10.18632/aging.100895 PMC4925820

[30] Derous D, Mitchell SE, Green CL The effects of graded levels of calorie restriction: VII. Topological rearrangement of hypothalamic aging networks. Aging (Albany NY). 2016;8:917–932. doi:10.18632/aging.100944 27115072 10.18632/aging.100944 PMC4931844

[31] Petersen TN, Brunak S, von Heijne G, Nielsen H SignalP 4.0: discriminating signal peptides from transmembrane regions. Nat Methods. 2011;8:785–786. doi:10.1038/nmeth.1701 21959131 10.1038/nmeth.1701

[32] Shannon P, Markiel A, Ozier O Cytoscape: a software environment for integrated models of biomolecular interaction networks. Genome Res. 2003;13:2498–2504. doi:10.1101/gr.1239303 14597658 10.1101/gr.1239303 PMC403769

[33] Mitchell SE, Tang Z, Kerbois C The effects of graded levels of calorie restriction: VIII. Impact of short term calorie and protein restriction on basal metabolic rate in the C57BL/6 mouse. Oncotarget. 2017;8:17453–17474. doi:10.18632/oncotarget.15294 28193912 10.18632/oncotarget.15294 PMC5392262

[34] Green CL, Mitchell SE, Derous D The effects of graded levels of calorie restriction: IX. Global metabolomic screen reveals modulation of carnitines, sphingolipids and bile acids in the liver of C57BL/6 mice. Aging Cell. 2017;16:529–540. doi:10.1111/acel.12570 28139067 10.1111/acel.12570 PMC5418186

[35] Bagga D, Byerley LO, Koziol BJ, Glick Z, Ashley JM, Heber D Adipose tissue and the effects of fat and calories on breast tumorigenesis in rats. J Nutr Biochem. 1995;6:667–672. doi:10.1016/0955-2863(95)00144-1

[36] Barzilai N, Banerjee S, Hawkins M, Chen W, Rossetti L Caloric restriction reverses hepatic insulin resistance in aging rats by decreasing visceral fat. J Clin Invest. 1998;101:1353–1361. doi:10.1172/JCI485 9525977 10.1172/JCI485 PMC508712

[37] Speakman JR, Mitchell SE, Mazidi M Calories or protein? The effect of dietary restriction on lifespan in rodents is explained by calories alone. Exp Gerontol. 2016;86:28–38. doi:10.1016/j.exger.2016.03.011 27006163 10.1016/j.exger.2016.03.011

[38] Kim HJ, Jung KJ, Yu BP, Cho CG, Choi JS, Chung HY Modulation of redox-sensitive transcription factors by calorie restriction during aging. Mech Ageing Dev. 2002;123:1589–1595. doi:10.1016/S0047-6374(02)00094-5 12470896 10.1016/s0047-6374(02)00094-5

[39] Fabbiano S, Suárez-Zamorano N, Rigo D Caloric restriction leads to browning of white adipose tissue through type 2 immune signaling. Cell Metab. 2016;24:434–446. doi:10.1016/j.cmet.2016.07.023 27568549 10.1016/j.cmet.2016.07.023

[40] Muller PAJ, Vousden KH Mutant p53 in cancer: new functions and therapeutic opportunities. Cancer Cell. 2014;25:304–317. doi:10.1016/j.ccr.2014.01.021 24651012 10.1016/j.ccr.2014.01.021 PMC3970583

[41] Hursting SD, Perkins SN, Phang JM Calorie restriction delays spontaneous tumorigenesis in p53-knockout transgenic mice. Proc Natl Acad Sci USA. 1994;91:7036–7040. doi:10.1073/pnas.91.15.7036 8041741 10.1073/pnas.91.15.7036 PMC44333

[42] Ortega-Molina A, Serrano M PTEN in cancer, metabolism, and aging. Trends Endocrinol Metab. 2013;24:184–189. doi:10.1016/j.tem.2012.11.002 23245767 10.1016/j.tem.2012.11.002 PMC3836169

[43] Longo VD, Fontana L Calorie restriction and cancer prevention: metabolic and molecular mechanisms. Trends Pharmacol Sci. 2010;31:89–98. doi:10.1016/j.tips.2009.11.004 20097433 10.1016/j.tips.2009.11.004 PMC2829867

[44] Calle EE, Kaaks R Overweight, obesity and cancer: epidemiological evidence and proposed mechanisms. Nat Rev Cancer. 2004;4:579–591. doi:10.1038/nrc1408 15286738 10.1038/nrc1408

[45] Tucci P Caloric restriction: is mammalian life extension linked to p53?Aging (Albany NY). 2012;4:525–534. doi:10.18632/aging.100481 22983298 10.18632/aging.100481 PMC3461340

[46] Minamino T, Orimo M, Shimizu I A crucial role for adipose tissue p53 in the regulation of insulin resistance. Nat Med. 2009;15:1082–1087. doi:10.1038/nm.2014 19718037 10.1038/nm.2014

[47] Hu W, Feng Z, Teresky AK, Levine AJ p53 regulates maternal reproduction through LIF. Nature. 2007;450:721–724. doi:10.1038/nature05993 18046411 10.1038/nature05993

[48] Kowald A, Kirkwood TB Evolutionary significance of ageing in the wild. Exp Gerontol. 2015;71:89–94. doi:10.1016/j.exger.2015.08.006 26292149 10.1016/j.exger.2015.08.006

[49] Speakman JR, Król E The heat dissipation limit theory and evolution of life histories in endotherms–time to dispose of the disposable soma theory?Integr Comp Biol. 2010;50:793–807. doi:10.1093/icb/icq049 21558242 10.1093/icb/icq049

[50] Beynon RJ, Hurst JL Urinary proteins and the modulation of chemical scents in mice and rats. Peptides. 2004;25:1553–1563. doi:10.1016/j.peptides.2003.12.025 15374657 10.1016/j.peptides.2003.12.025

[51] van Schothorst EM, Keijer J, Pennings JL Adipose gene expression response of lean and obese mice to short-term dietary restriction. Obesity (Silver Spring). 2006;14:974–979. doi:10.1038/oby.2006.111 16861601 10.1038/oby.2006.111

[52] Wink DA, Hines HB, Cheng RY Nitric oxide and redox mechanisms in the immune response. J Leukoc Biol. 2011;89:873–891. doi:10.1189/jlb.1010550 21233414 10.1189/jlb.1010550 PMC3100761

[53] Banerjee Mustafi S, Chakraborty PK, Dey RS, Raha S Heat stress upregulates chaperone heat shock protein 70 and antioxidant manganese superoxide dismutase through reactive oxygen species (ROS), p38MAPK, and Akt. Cell Stress Chaperones. 2009;14:579–589. doi:10.1007/s12192-009-0109-x 19291423 10.1007/s12192-009-0109-x PMC2866949

[54] Belikov AV, Schraven B, Simeoni L T cells and reactive oxygen species. J Biomed Sci. 2015;22:85. doi:10.1186/s12929-015-0194-3 26471060 10.1186/s12929-015-0194-3 PMC4608155

[55] Zuo L, Rose BA, Roberts WJ, He F, Banes-Berceli AK Molecular characterization of reactive oxygen species in systemic and pulmonary hypertension. Am J Hypertens. 2014;27:643–650. doi:10.1093/ajh/hpt292 24552887 10.1093/ajh/hpt292

[56] Rutkowski JM, Stern JH, Scherer PE The cell biology of fat expansion. J Cell Biol. 2015;208:501–512. doi:10.1083/jcb.201409063 25733711 10.1083/jcb.201409063 PMC4347644

[57] Cao Y Adipose tissue angiogenesis as a therapeutic target for obesity and metabolic diseases. Nat Rev Drug Discov. 2010;9:107–115. doi:10.1038/nrd3055 20118961 10.1038/nrd3055

[58] Rupnick MA, Panigrahy D, Zhang CY Adipose tissue mass can be regulated through the vasculature. Proc Natl Acad Sci USA. 2002;99:10730–10735. doi:10.1073/pnas.162349799 12149466 10.1073/pnas.162349799 PMC125027

[59] Tracy RP Thrombin, inflammation, and cardiovascular disease: an epidemiologic perspective. Chest. 2003;124(3 suppl):49S–57S. doi:10.1378/chest.124.3_suppl.49S 12970124 10.1378/chest.124.3_suppl.49s

[60] Strande JL, Phillips SA Thrombin increases inflammatory cytokine and angiogenic growth factor secretion in human adipose cells in vitro. J Inflamm (Lond). 2009;6:4. doi:10.1186/1476-9255-6-4 19267924 10.1186/1476-9255-6-4 PMC2661073

[61] Mascaro A, D’Antona G, D’Antona G Proteomic analysis of plasma after 4 weeks of intermittent fasting in mice. Med J Nutrition Metab. 2013;6:227–232. doi:10.1007/s12349-013-0136-0

[62] Mann KG, Kalafatis M Factor V: a combination of Dr Jekyll and Mr Hyde. Blood. 2003;101:20–30. doi:10.1182/blood-2002-01-0290 12393635 10.1182/blood-2002-01-0290

[63] Barbier O, Torra IP, Duguay Y Pleiotropic actions of peroxisome proliferator-activated receptors in lipid metabolism and atherosclerosis. Arterioscler Thromb Vasc Biol. 2002;22:717–726. doi:10.1161/01.ATV.0000015598.86369.04 12006382 10.1161/01.atv.0000015598.86369.04

[64] Stauber AJ, Brown-Borg H, Liu J Constitutive expression of peroxisome proliferator-activated receptor alpha-regulated genes in dwarf mice. Mol Pharmacol. 2005;67:681–694. doi:10.1124/mol.104.007278 15576629 10.1124/mol.104.007278

[65] Laffel L Ketone bodies: a review of physiology, pathophysiology and application of monitoring to diabetes. Diabetes Metab Res Rev. 1999;15:412–426. doi:10.1002/(SICI)1520–7560(199911/12)15:6<412:: AID-DMRR72>3.0.CO;2–8 10634967 10.1002/(sici)1520-7560(199911/12)15:6<412::aid-dmrr72>3.0.co;2-8

[66] Newman JC, Verdin E Ketone bodies as signaling metabolites. Trends Endocrinol Metab. 2014;25:42–52. doi:10.1016/j.tem.2013.09.002 24140022 10.1016/j.tem.2013.09.002 PMC4176946

[67] Hanson RW, Ziporin ZZ Factors influencing the utilization of ketone bodies by mouse adipose tissue. J Lipid Res. 1966;7:56–61. 4221104

[68] Gattu AK, Birkenfeld AL, Iwakiri Y Pigment epithelium-derived factor (PEDF) suppresses IL-1β-mediated c-Jun N-terminal kinase (JNK) activation to improve hepatocyte insulin signaling. Endocrinology. 2014;155:1373–1385. doi:10.1210/en.2013-1785 24456163 10.1210/en.2013-1785 PMC5393334

[69] Strehlow AN, Li JZ, Myers RM Wild-type huntingtin participates in protein trafficking between the Golgi and the extracellular space. Hum Mol Genet. 2007;16:391–409. doi:10.1093/hmg/ddl467 17189290 10.1093/hmg/ddl467

[70] Pakradouni J, Le Goff W, Calmel C Plasma NOV/CCN3 levels are closely associated with obesity in patients with metabolic disorders. PLoS One. 2013;8:e66788. doi:10.1371/journal.pone.0066788 23785511 10.1371/journal.pone.0066788 PMC3681908

[71] Kim HS, Nagalla SR, Oh Y, Wilson E, Roberts CT Jr, Rosenfeld RG Identification of a family of low-affinity insulin-like growth factor binding proteins (IGFBPs): characterization of connective tissue growth factor as a member of the IGFBP superfamily. Proc Natl Acad Sci USA. 1997;94:12981–12986. doi:10.1073/pnas.94.24.12981 9371786 10.1073/pnas.94.24.12981 PMC24249

[72] Manara MC, Perbal B, Benini S The expression of ccn3(nov) gene in musculoskeletal tumors. Am J Pathol. 2002;160:849–859. doi:10.1016/S0002-9440(10)64908-5 11891184 10.1016/S0002-9440(10)64908-5 PMC1867180

[73] Benini S, Perbal B, Zambelli D In Ewing’s sarcoma CCN3(NOV) inhibits proliferation while promoting migration and invasion of the same cell type. Oncogene. 2005;24:4349–4361. doi:10.1038/sj.onc.1208620 15824736 10.1038/sj.onc.1208620

[74] Paradis R, Lazar N, Antinozzi P, Perbal B, Buteau J Nov/Ccn3, a novel transcriptional target of FoxO1, impairs pancreatic β-cell function. PLoS One. 2013;8:e64957. doi:10.1371/journal.pone.0064957 23705021 10.1371/journal.pone.0064957 PMC3660386

[75] Schmitz O, Rungby J, Edge L, Juhl CB On high-frequency insulin oscillations. Ageing Res Rev. 2008;7:301–305. doi:10.1016/j.arr.2008.04.002 18583199 10.1016/j.arr.2008.04.002

[76] Fehmann HC, Peiser C, Bode HP Leptin: a potent inhibitor of insulin secretion. Peptides. 1997;18:1267–1273. doi:10.1016/S0196-9781(97)00135-6 9396072 10.1016/s0196-9781(97)00135-6

[77] Wolkow CA, Kimura KD, Lee MS, Ruvkun G Regulation of *C. elegans* life-span by insulinlike signaling in the nervous system. Science. 2000;290:147–150. doi:10.1126/science.290.5489.147 11021802 10.1126/science.290.5489.147

[78] Kimura KD, Tissenbaum HA, Liu Y, Ruvkun G daf-2, an insulin receptor-like gene that regulates longevity and diapause in *Caenorhabditis elegans*. Science. 1997;277:942–946. doi:10.1126/science.277.5328.942 9252323 10.1126/science.277.5328.942

[79] Blüher M, Michael MD, Peroni OD Adipose tissue selective insulin receptor knockout protects against obesity and obesity-related glucose intolerance. Dev Cell. 2002;3:25–38. doi:10.1016/S1534-5807(02)00199-5 12110165 10.1016/s1534-5807(02)00199-5

[80] Shaw WM, Luo S, Landis J, Ashraf J, Murphy CT The *C. elegans* TGF-beta Dauer pathway regulates longevity via insulin signaling. Curr Biol. 2007;17:1635–1645. doi:10.1016/j.cub.2007.08.058 17900898 10.1016/j.cub.2007.08.058 PMC3124252

[81] Murphy CT, McCarroll SA, Bargmann CI Genes that act downstream of DAF-16 to influence the lifespan of *Caenorhabditis elegans*. Nature. 2003;424:277–283. doi:10.1038/nature01789 12845331 10.1038/nature01789

[82] Samad F, Yamamoto K, Pandey M, Loskutoff DJ Elevated expression of transforming growth factor-beta in adipose tissue from obese mice. Mol Med. 1997;3:37–48. 9132278 PMC2230108

[83] Chiba T, Yamaza H, Higami Y, Shimokawa I Anti-aging effects of caloric restriction: involvement of neuroendocrine adaptation by peripheral signaling. Microsc Res Tech. 2002;59:317–324. doi:10.1002/jemt.10211 12424795 10.1002/jemt.10211

[84] Mitchell SJ, Madrigal-Matute J, Scheibye-Knudsen M Effects of sex, strain, and energy intake on hallmarks of aging in mice. Cell Metab. 2016;23:1093–1112. doi:10.1016/j.cmet.2016.05.027 27304509 10.1016/j.cmet.2016.05.027 PMC4911707

[85] Robinson MD, McCarthy DJ, Smyth GK edgeR: a Bioconductor package for differential expression analysis of digital gene expression data. Bioinformatics. 2010;26:139–140. doi:10.1093/bioinformatics/btp616 19910308 10.1093/bioinformatics/btp616 PMC2796818

[86] R Core Team. R: A Language and Environment for Statistical Computing. Vienna, Austria; 2014 http://www.r-project.org/.

[87] Benjamini Y, Hochberg Y Controlling the false discovery rate: a practical and powerful approach to multiple testing. J R Stat Soc Ser B. 1995;57:289–300. doi:10.2307/2346101

