# Supplementary material for: The Effects of Graded Levels of Calorie Restriction: X. Transcriptomic Responses of Epididymal Adipose Tissue
Source: J Gerontol A Biol Sci Med Sci. 2017 May 27;73(3):279–88. doi: 10.1093/gerona/glx101 (PMC5861923; doi:10.1093/gerona/glx101)
Supplement: Supplementary_Information [file glx101_suppl_supplementary_information.docx]

**Supplementary Results**

*Differential gene expression analysis relative to 24h ad libitum intake.*

Differential gene expression analysis relative to 24AL (FDR < 0.05) showed that a total 3375 genes were significantly differently expressed in at least one of the CR levels. The DEGs list relative to 12AL and to 24AL had 2316 genes in common while 1119 of the 3435 DEGs relative to 12AL became non-significant when the CR levels were compared to 24AL.


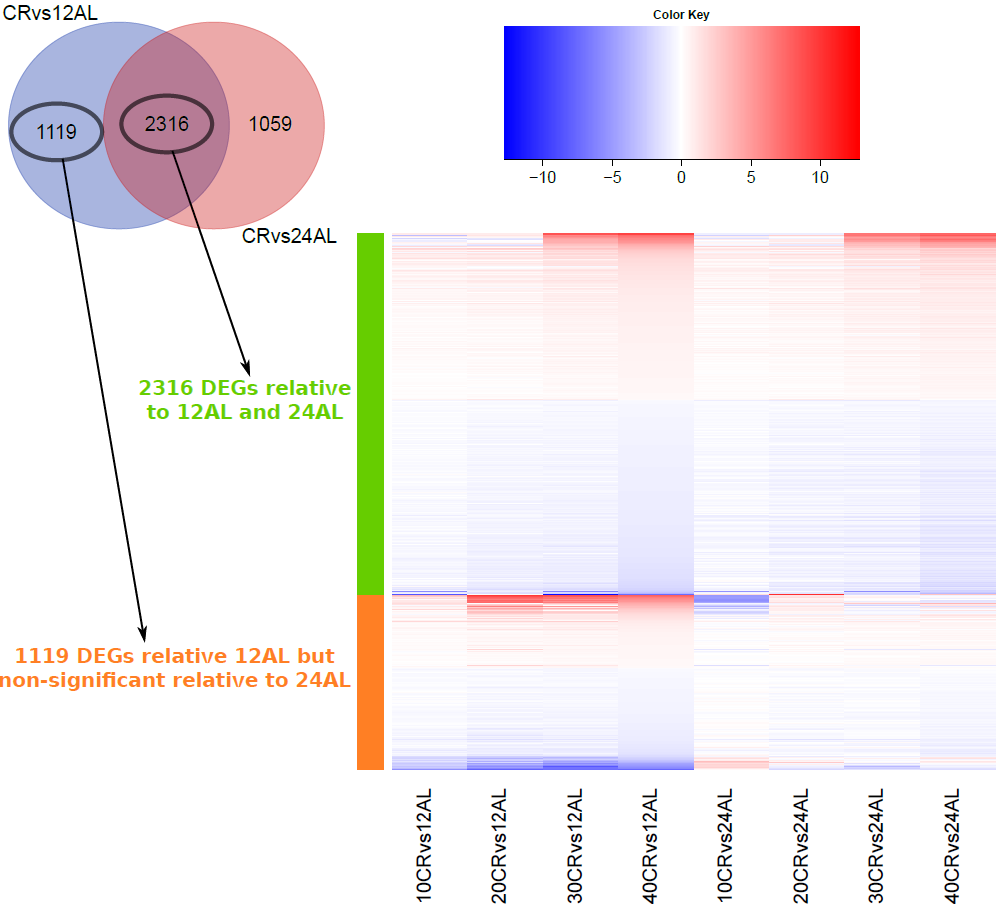


Figure S1. Differentially expressed genes (DEGs) at each level of restriction relative to 12h *ad libitum* (AL) and 24AL. The Venn diagram represent the overlap in DEGs when the level of CR is compared to 12AL or 24AL. The heat map represents the 2316 genes that were significantly expressed both relative to 12AL and 24AL (green column annotation) and those DEGs relative to 12AL that become non-significant when CR was compared to 24AL (orange column annotation). In the heat map, red indicates a positive log2 fold change (log FC) and blue a negative log FC. 10CR, 20CR, 30CR and 40CR refer to 10 %, 20 %, 30 % and 40 % restriction.

*Pathway identification relative to 24h ad libitum intake.*

We also identified pathways based on the DEGs relative to 24AL. We observed an increase in pathway significance with increasing CR level but the pattern did not follow a graded increase in significance. Of the 92 significant pathways relative to 24AL, only 27 are similar to those significant relative to 12AL (n=88) (Figure S2).


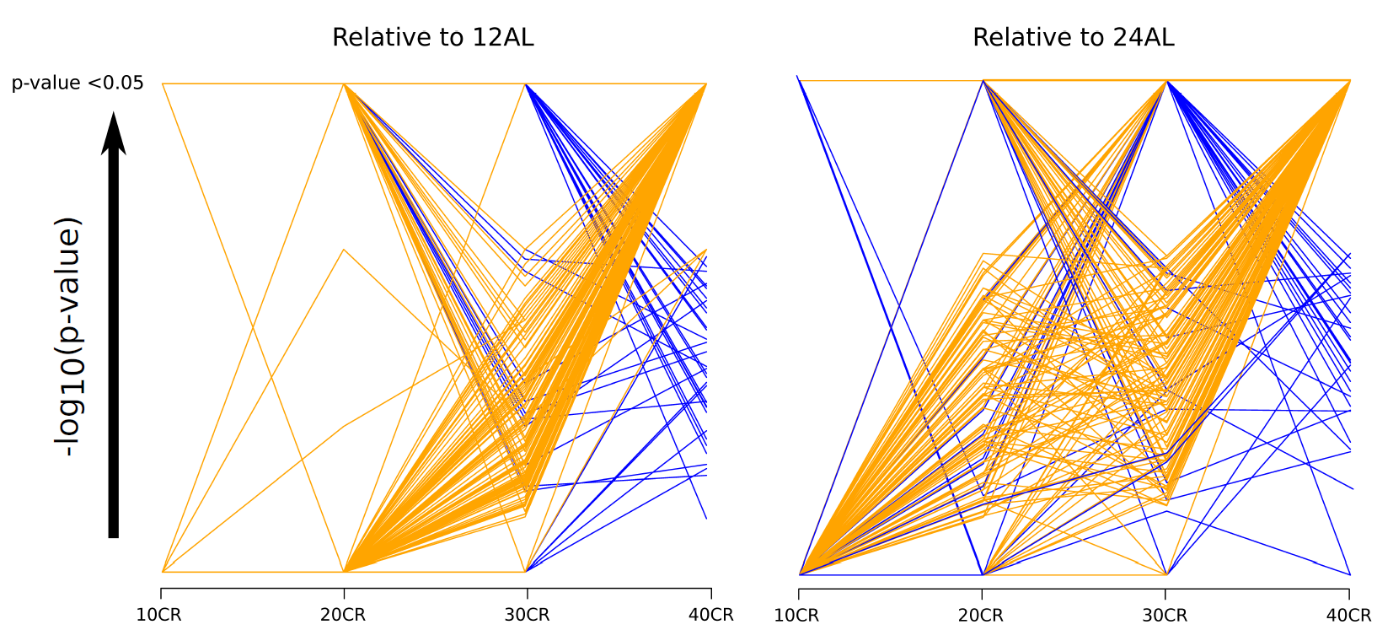


Figure 2. Pathways recruited with increasing CR level identified by Ingenuity Pathway Analysis (IPA, www.qiagen.com/ingenuity). Plot visualizing the increase in significance of pathways with increasing CR level relative to 12AL (n=287) and to 24AL (n=254). The Y-axis represents the –log10(p-value) of the pathway, with a maximum value of less than 0.05. Yellow indicates pathways that were significantly altered at 40CR (p-value < 0.05) and blue represents pathways that were not significantly altered at 40CR (p-value > 0.05) relative to 12AL or 24AL. 10CR, 20CR, 30CR and 40CR refer to 10 %, 20 %, 30 % and 40 % restriction.

**Supplemental figures**


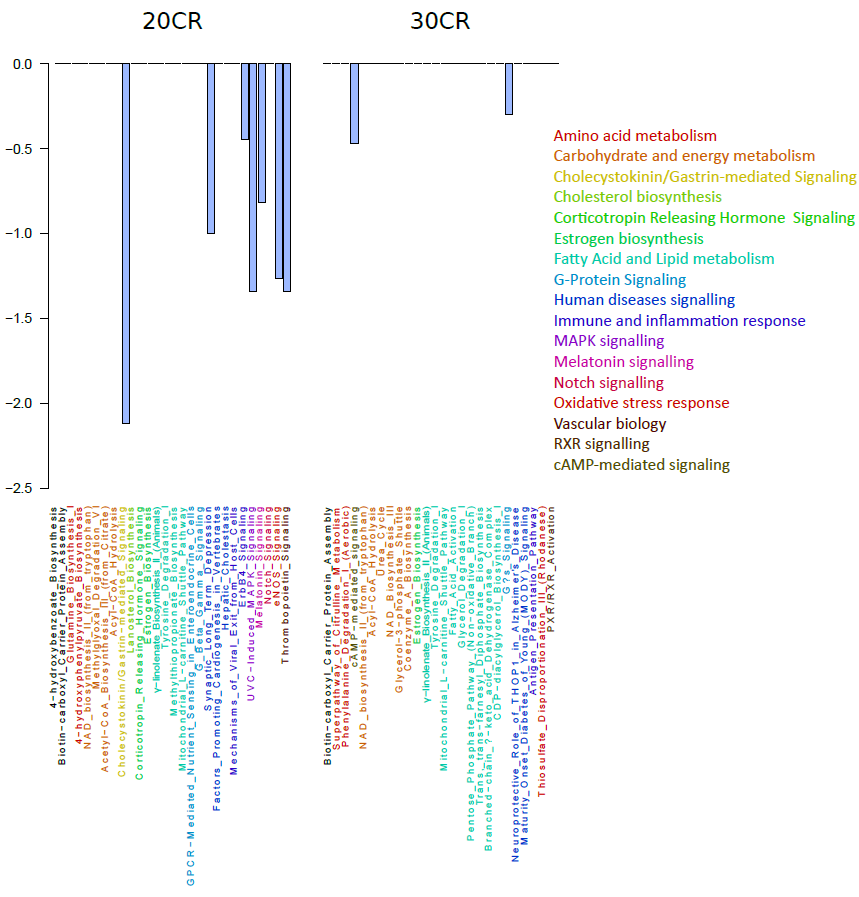
Figure S3. Pathways that were “switched-off” at 40CR (n = 54). Plot represent the enrichment score identified by Ingenuity Pathway Analysis (IPA, www.qiagen.com/ingenuity). Blue indicates an inhibition and red indicates an activation relative to 12h *ad libitum* (12AL). Pathways are coloured according to their summarised biological function. 20CR and 30CR refer to 20 % and 30 % restriction.


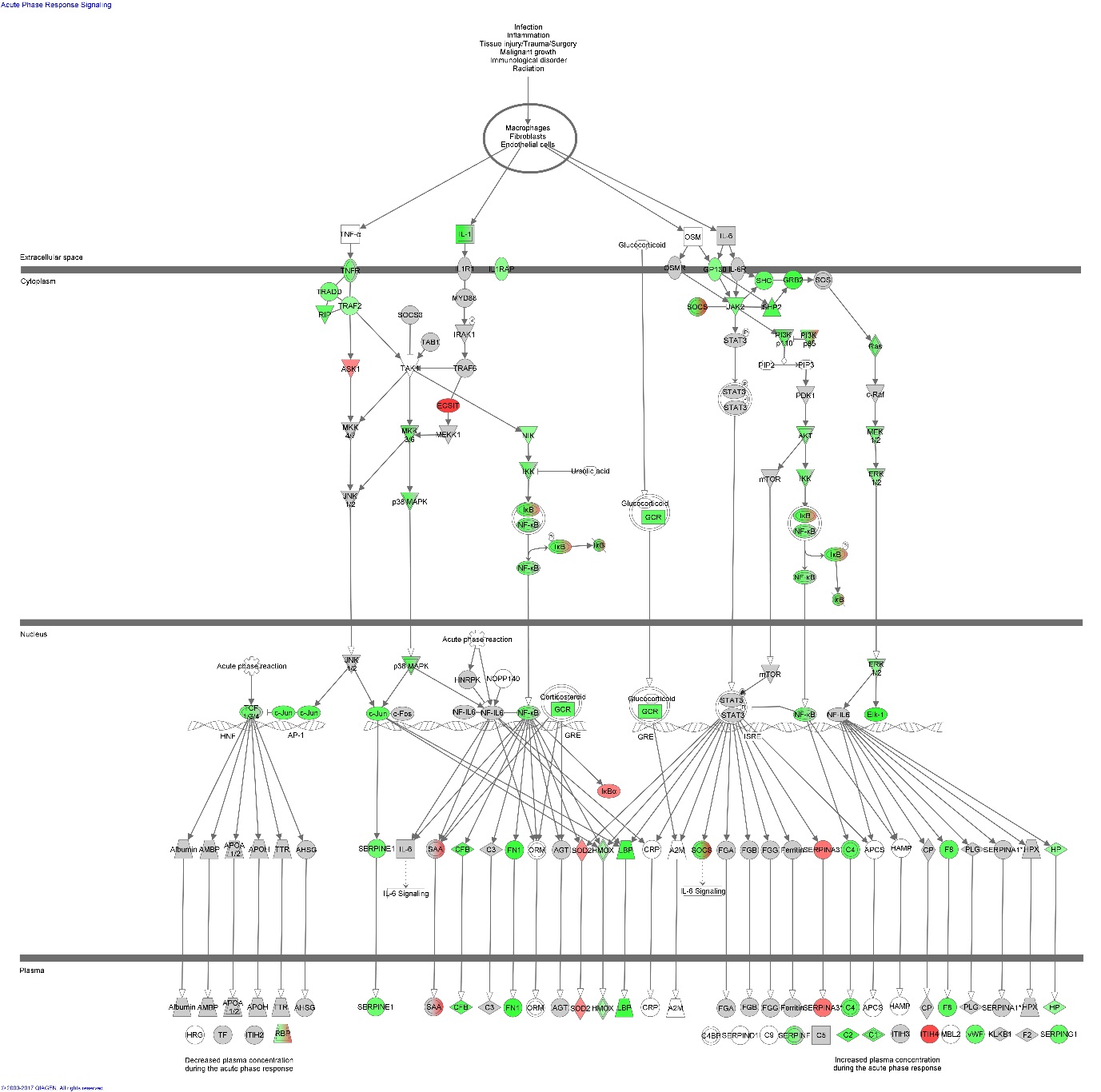


Figure S4. The acute phase response signalling pathway obtained from Ingenuity Pathway Analysis program (IPA, www.qiagen.com/ingenuity) coloured according to genes correlating with increase in CR levels. Red indicates a positive correlation coefficient and green indicates a negative correlation coefficient. Intensity of the colour is related to the strength of the correlation.


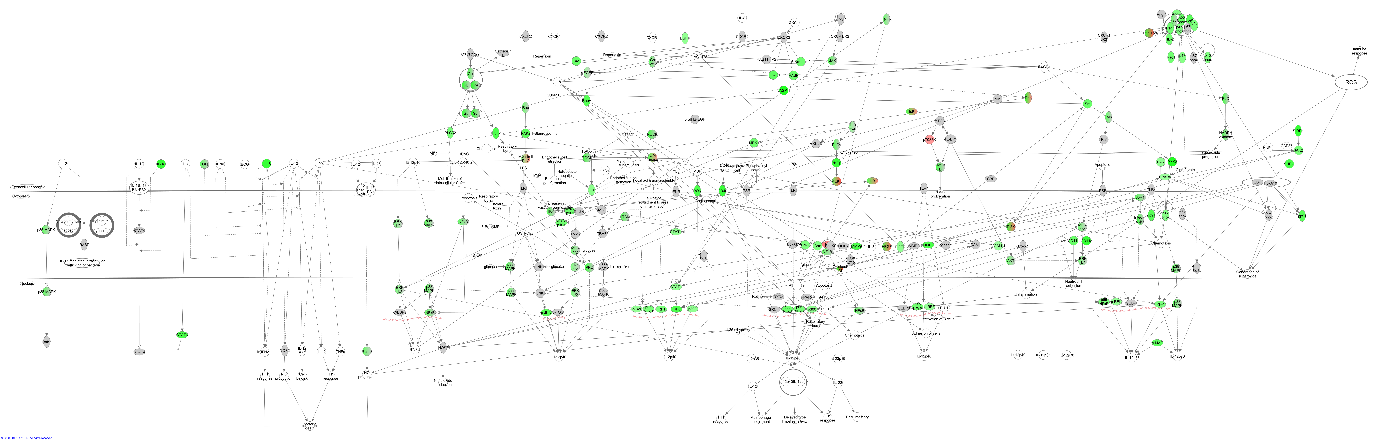


Figure S5. The inflammation and oxidative stress pathway constructed in the Ingenuity Pathway Analysis (IPA, www.qiagen.com/ingenuity) program by merging the pathways IL-12 Signalling and Production in Macrophages, IL-8 Signalling and Production of Nitric Oxide and Reactive Oxygen Species in Macrophages. Pathway is coloured according to genes correlating with increase in CR levels. Red indicates a positive correlation coefficient and green indicates a negative correlation coefficient. Intensity of the colour is related to the strength of the correlation.


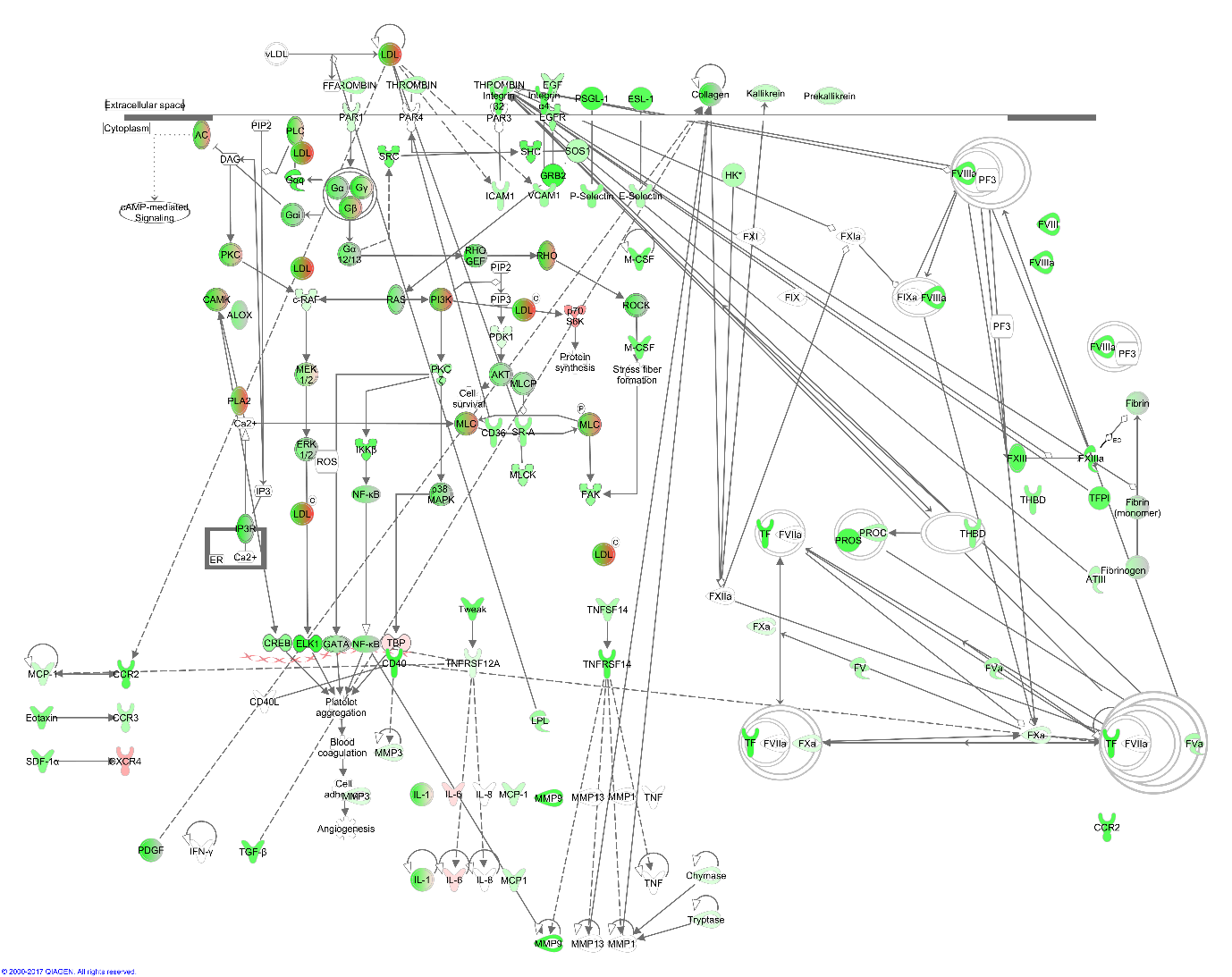


Figure S6. The vascular biology pathway constructed in the Ingenuity Pathway Analysis (IPA, www.qiagen.com/ingenuity) program by merging the pathways Extrinsic Prothrombin Activation Pathway, Intrinsic Prothrombin Activation Pathway, Thrombin Signalling and Atherosclerosis Signalling. Pathway is coloured according to genes correlating with increase in CR levels. Red indicates a positive correlation coefficient and green indicates a negative correlation coefficient. Intensity of the colour is related to the strength of the correlation.


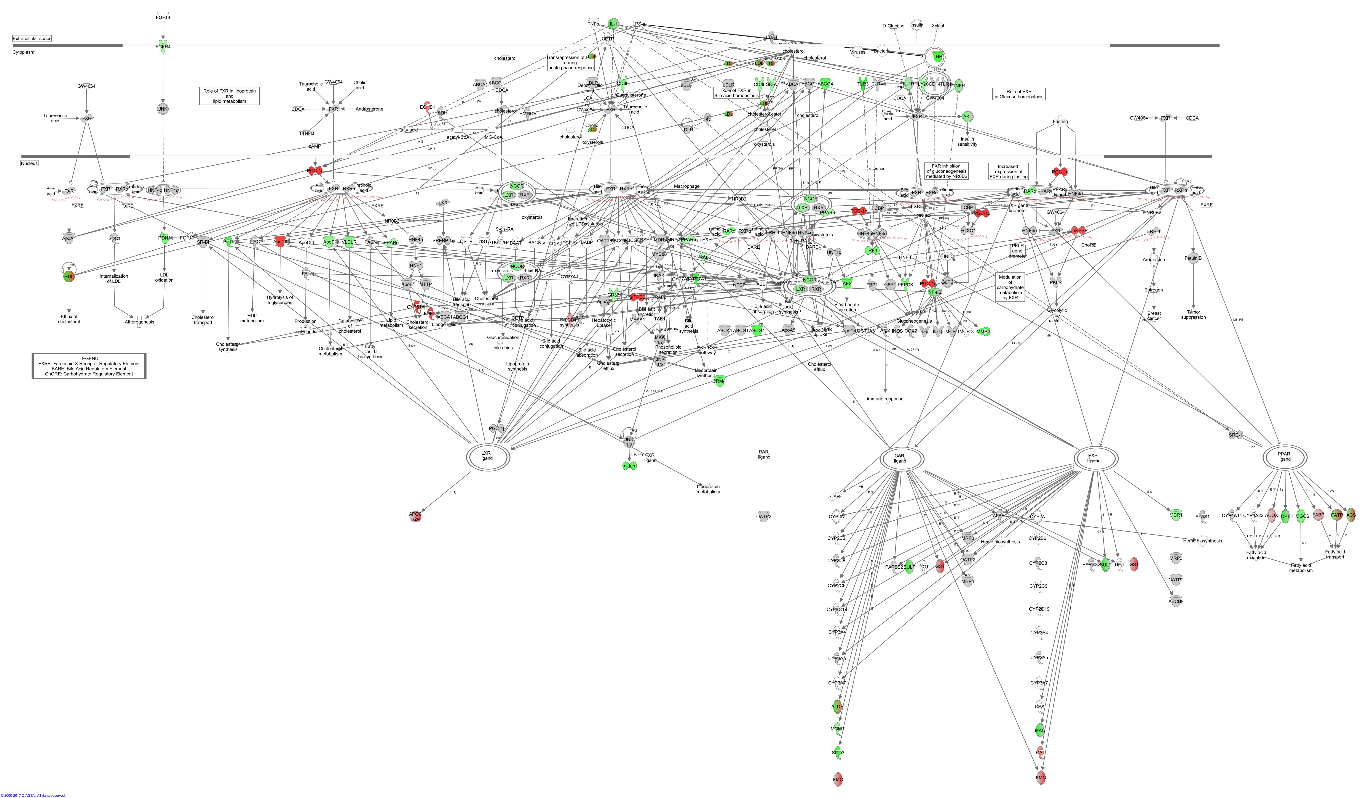


Figure S7. The RXR signalling pathway constructed in the Ingenuity Pathway Analysis (IPA, www.qiagen.com/ingenuity) program by merging the pathways LPS/IL-1 Mediated Inhibition of RXR Function, LXR/RXR Activation and FXR/RXR Activation. Pathway is coloured according to genes correlating with increase in CR levels. Red indicates a positive correlation coefficient and green indicates a negative correlation coefficient. Intensity of the colour is related to the strength of the correlation.

**Supplemental Tables**

Supplementary Table 1. Significantly altered pathways at 10 % restriction relative to 12h *ad libitum* intake identified by Ingenuity Pathway Analysis (IPA, www.qiagen.com/ingenuity).

| **Ingenuity Canonical Pathways** | **-log(p-value)** | **Molecules** |
| --- | --- | --- |
| Stearate Biosynthesis I (Animals) | 3.45E00 | ACOT1,ELOVL6 |
| Acyl-CoA Hydrolysis | 2.03E00 | ACOT1 |
| Coagulation System | 1.57E00 | SERPINA1 |
| Triacylglycerol Biosynthesis | 1.57E00 | ELOVL6 |

Supplementary Table 2. Significantly altered pathways at 20 % restriction relative to 12h *ad libitum* intake identified by Ingenuity Pathway Analysis (IPA, www.qiagen.com/ingenuity).

| **Ingenuity Canonical Pathways** | **-log(p-value)** | **Molecules** |
| --- | --- | --- |
| LXR/RXR Activation | 1.4E01 | KNG1, APOE, APOB, APOH, ABCG4, NR1H4, APOA2, APOC4, VTN, AMBP, C4A/C4B, FDFT1, PON1, LYZ, LCAT, SAA1, SERPINA1, LBP, IL1RAP, CYP51A1, AGT, HPX, AHSG, SERPINF1, ALB, ACACA |
| Superpathway of Cholesterol Biosynthesis | 9.34E00 | MVD, FDPS, SQLE, FDFT1, NSDHL, PMVK, ACAT2, LSS, HMGCS2, TM7SF2, CYP51A1 |
| FXR/RXR Activation | 9.17E00 | KNG1, APOE, HPX, APOB, SLC10A1, APOH, NR1H4, VTN, APOC4, APOA2, AHSG, AMBP, SERPINF1, C4A/C4B, PON1, ALB, LCAT, SAA1, RARA, SERPINA1, AGT |
| Cholesterol Biosynthesis I | 5.82E00 | SQLE, FDFT1, NSDHL, LSS, TM7SF2, CYP51A1 |
| Cholesterol Biosynthesis II (via 24, 25-dihydrolanosterol) | 5.82E00 | SQLE, FDFT1, NSDHL, LSS, TM7SF2, CYP51A1 |
| Cholesterol Biosynthesis III (via Desmosterol) | 5.82E00 | SQLE, FDFT1, NSDHL, LSS, TM7SF2, CYP51A1 |
| Hepatic Fibrosis / Hepatic Stellate Cell Activation | 5.72E00 | CTGF, FN1, COL6A2, FGFR1, FLT4, VEGFC, COL8A1, COL15A1, COL6A6, COL6A1, COL6A3, CSF1, TGFB3, COL6A5, LBP, COL18A1, IL1RAP, EGFR, AGT, PDGFRB |
| Acute Phase Response Signaling | 5.05E00 | ITIH3, HPX, FN1, APOH, APOA2, AHSG, AMBP, SERPINF1, SERPINA3, C5, C4A/C4B, ALB, ITIH2, SAA1, SERPINA1, LBP, IL1RAP, AGT |
| Coagulation System | 4.93E00 | KNG1, F10, SERPINC1, F5, SERPINA1, TFPI, THBD, PLAT |
| IL-8 Signaling | 4.55E00 | ANGPT2, PTK2B, RHOC, FLT4, VEGFC, RHOJ, PRKCG, GNB4, GNG11, PRKCE, PRKCH, PRKD3, TEK, LASP1, VASP, ITGB5, MYL12B, EGFR |
| Sperm Motility | 4.46E00 | CACNA1G, PTK2B, ABHD3, PDE4B, PRKCG, PLCE1, LCAT, PRKCE, PRKCH, CATSPER2, ADCY10, PRKD3, GUCY1B3, PLA2G12A |
| LPS/IL-1 Mediated Inhibition of RXR Function | 4.05E00 | GSTM1, APOE, CPT1A, SLC10A1, NR1H4, APOC4, HMGCS2, CHST15, SOD3, HS3ST3B1, MGST2, RARA, Gm4846, SLC27A1, FABP5, LBP, IL1RAP, ACSL1, CYP2C8 |
| Extrinsic Prothrombin Activation Pathway | 3.96E00 | F10, SERPINC1, F5, TFPI, THBD |
| IL-12 Signaling and Production in Macrophages | 3.94E00 | STAT6, APOE, APOB, APOC4, APOA2, PRKCG, PON1, ALB, LYZ, TGFB3, PRKCE, SERPINA1, PRKCH, PRKD3 |
| Superpathway of Geranylgeranyldiphosphate Biosynthesis I (via Mevalonate) | 3.82E00 | MVD, FDPS, PMVK, ACAT2, HMGCS2 |
| Axonal Guidance Signaling | 3.76E00 | UNC5A, EPHB2, SEMA4C, GNB4, MICAL1, GNG11, PLCE1, ADAM28, EFNB1, SMO, PRKCE, SEMA3F, PRKD3, ADAMTS5, MYL12B, VASP, PLXNA1, EPHB4, ADAMTS1, ADAM15, TUBB2A, VEGFC, L1CAM, PLXND1, PRKCG, TUBA1A, NTRK2, LINGO1, PRKCH |
| Atherosclerosis Signaling | 3.74E00 | PON1, APOE, LYZ, ALB, APOB, LCAT, CSF1, ABHD3, APOA2, APOC4, SERPINA1, COL18A1, PLA2G12A |
| Production of Nitric Oxide and Reactive Oxygen Species in Macrophages | 3.62E00 | MAP3K15, APOE, APOB, RHOC, APOC4, APOA2, RHOJ, PRKCG, PON1, ALB, LYZ, PPP1R3D, PRKCE, SERPINA1, PRKCH, PRKD3 |
| Intrinsic Prothrombin Activation Pathway | 3.57E00 | KNG1, F10, SERPINC1, F5, THBD, COL18A1 |
| Mevalonate Pathway I | 3.22E00 | MVD, PMVK, ACAT2, HMGCS2 |
| Zymosterol Biosynthesis | 3.21E00 | NSDHL, TM7SF2, CYP51A1 |
| Phospholipase C Signaling | 3.12E00 | PEBP1, RHOC, RPS6KA3, CD79A, RHOJ, ARHGEF17, PRKCG, GNB4, GNG11, PLCE1, PRKCE, ARHGEF2, PRKCH, ADCY10, ARHGEF10, PRKD3, MYL12B, PLA2G12A |
| Fcγ Receptor-mediated Phagocytosis in Macrophages and Monocytes | 3.1E00 | GAB2, TLN2, PTK2B, DGKB, PRKCE, PRKCH, ACTG2, PRKD3, VASP, PRKCG |
| Stearate Biosynthesis I (Animals) | 3.04E00 | ACOT2, ACOT1, ACOT4, SLC27A1, ACSL1, ELOVL6 |
| Epoxysqualene Biosynthesis | 2.99E00 | SQLE, FDFT1 |
| Acetyl-CoA Biosynthesis I (Pyruvate Dehydrogenase Complex) | 2.98E00 | PDHA1, DLAT, PDHB |
| Gap Junction Signaling | 2.82E00 | DBN1, TUBB2A, PRKCG, PLCE1, NOV, TUBA1A, PRKCE, PRKCH, ACTG2, ADCY10, PRKD3, GUCY1B3, EGFR |
| Tryptophan Degradation to 2-amino-3-carboxymuconate Semialdehyde | 2.79E00 | TDO2, HAAO, IDO1 |
| Hepatic Cholestasis | 2.65E00 | SLC10A1, NR1H4, PRKCG, PPRC1, RARA, TGFB3, PRKCE, PRKCH, LBP, ADCY10, ESR1, PRKD3, IL1RAP |
| Thrombin Signaling | 2.45E00 | RHOC, RHOJ, PRKCG, GNB4, GNG11, PLCE1, PRKCE, ARHGEF2, PRKCH, ADCY10, PRKD3, ARHGEF10, MYL12B, EGFR |
| Tryptophan Degradation III (Eukaryotic) | 2.39E00 | ACAT2, TDO2, HAAO, IDO1 |
| Acyl-CoA Hydrolysis | 2.23E00 | ACOT2, ACOT1, ACOT4 |
| Biotin-carboxyl Carrier Protein Assembly | 2.23E00 | ACACB, ACACA |
| CCR5 Signaling in Macrophages | 2.18E00 | GNB4, GNG11, PTK2B, PRKCE, PRKCH, PRKD3, PRKCG |
| UVB-Induced MAPK Signaling | 2.16E00 | RPS6KA3, PRKCE, PRKCH, PRKD3, PRKCG, EGFR |
| Clathrin-mediated Endocytosis Signaling | 2.16E00 | PON1, APOE, LYZ, ALB, ARRB1, APOB, EPHB2, APOA2, APOC4, VEGFC, SERPINA1, ACTG2, ITGB5 |
| Breast Cancer Regulation by Stathmin1 | 2.05E00 | TUBB2A, ARHGEF17, PRKCG, GNB4, PPP1R3D, GNG11, TUBA1A, PRKCE, ARHGEF2, PRKCH, ADCY10, ARHGEF10, PRKD3 |
| Role of Tissue Factor in Cancer | 2.04E00 | F10, CTGF, ARRB1, PTK2B, CSF1, RPS6KA3, VEGFC, ITGB5, EGFR |
| Androgen Signaling | 2.02E00 | GNB4, TGFB1I1, GNG11, POLR2A, PRKCE, PRKCH, PRKD3, PRKCG, GTF2H3 |
| phagosome formation | 2.02E00 | FN1, PLCE1, RHOC, VTN, PRKCE, RHOJ, PRKCH, PRKD3, PRKCG |
| Tyrosine Degradation I | 2.01E00 | HPD, TAT |
| Acetate Conversion to Acetyl-CoA | 2.01E00 | ACSS2, ACSL1 |
| Mechanisms of Viral Exit from Host Cells | 2.01E00 | PRKCE, PRKCH, ACTG2, PRKD3, PRKCG |
| RhoGDI Signaling | 1.99E00 | GNB4, CDH2, GNG11, RHOC, RHOJ, ARHGEF17, ARHGEF2, ACTG2, ARHGEF10, ESR1, CDH11, MYL12B |
| UVC-Induced MAPK Signaling | 1.97E00 | PRKCE, PRKCH, PRKD3, PRKCG, EGFR |
| VEGF Family Ligand-Receptor Interactions | 1.96E00 | FLT4, PRKCE, VEGFC, PRKCH, PRKD3, PRKCG, PLA2G12A |
| NAD biosynthesis II (from tryptophan) | 1.95E00 | TDO2, HAAO, IDO1 |
| Tec Kinase Signaling | 1.88E00 | STAT6, GNB4, GNG11, PTK2B, RHOC, PRKCE, RHOJ, PRKCH, ACTG2, PRKD3, PRKCG |
| p70S6K Signaling | 1.84E00 | IL2RG, PLCE1, PRKCE, CD79A, PRKCH, PRKD3, AGT, PRKCG, EGFR |
| Nitric Oxide Signaling in the Cardiovascular System | 1.82E00 | KNG1, FLT4, PRKCE, VEGFC, PRKCH, PRKD3, GUCY1B3, PRKCG |
| γ-linolenate Biosynthesis II (Animals) | 1.79E00 | SLC27A1, ACSL1, FADS1 |
| Mitochondrial L-carnitine Shuttle Pathway | 1.79E00 | CPT1A, SLC27A1, ACSL1 |
| Cholecystokinin/Gastrin-mediated Signaling | 1.79E00 | PTK2B, RHOC, PRKCE, RHOJ, PRKCH, PRKD3, PRKCG, EGFR |
| eNOS Signaling | 1.78E00 | KNG1, FLT4, PRKCE, VEGFC, PRKCH, ADCY10, PRKD3, ESR1, GUCY1B3, PRKCG |
| GPCR-Mediated Nutrient Sensing in Enteroendocrine Cells | 1.74E00 | GNG11, PLCE1, PRKCE, PRKCH, ADCY10, PRKD3, PRKCG |
| Glutathione Redox Reactions I | 1.66E00 | MGST2, GPX1, GPX5 |
| α-Adrenergic Signaling | 1.66E00 | GNB4, GNG11, PRKCE, PRKCH, ADCY10, PRKD3, PRKCG |
| Macropinocytosis Signaling | 1.66E00 | CSF1, PRKCE, PRKCH, PRKD3, ITGB5, PRKCG |
| G Beta Gamma Signaling | 1.64E00 | GNB4, GNG11, PRKCE, PRKCH, PRKD3, PRKCG, EGFR |
| Sphingosine-1-phosphate Signaling | 1.61E00 | S1PR4, S1PR3, PLCE1, PTK2B, RHOC, RHOJ, ADCY10, PDGFRB |
| Melatonin Signaling | 1.6E00 | PLCE1, PRKCE, PRKCH, RORC, PRKD3, PRKCG |
| CXCR4 Signaling | 1.6E00 | GNB4, GNG11, RHOC, PRKCE, RHOJ, PRKCH, ADCY10, PRKD3, MYL12B, PRKCG |
| Protein Kinase A Signaling | 1.6E00 | AKAP12, PTK2B, PDE4B, PRKCG, AKAP2, GNB4, PPP1R3D, GNG11, DUSP3, PLCE1, DUSP10, TGFB3, SMO, PRKCE, DUSP18, PRKCH, ADCY10, PRKD3, VASP, MYL12B |
| IL-3 Signaling | 1.58E00 | GAB2, STAT6, PRKCE, PRKCH, PRKD3, PRKCG |
| Corticotropin Releasing Hormone Signaling | 1.57E00 | CNR1, SMO, PRKCE, PRKCH, ADCY10, PRKD3, GUCY1B3, PRKCG |
| Factors Promoting Cardiogenesis in Vertebrates | 1.54E00 | TGFB3, SMO, PRKCE, PRKCH, PRKD3, LRP1, PRKCG |
| Complement System | 1.52E00 | C4A/C4B, C7, C6, C5 |
| Thrombopoietin Signaling | 1.5E00 | GAB2, PRKCE, PRKCH, PRKD3, PRKCG |
| Role of Macrophages, Fibroblasts and Endothelial Cells in Rheumatoid Arthritis | 1.49E00 | FN1, DAAM1, VEGFC, IL17RA, C5, PRKCG, IL18R1, PLCE1, CSF1, SMO, SFRP5, PRKCE, PRKCH, IL1RAP, PRKD3, LRP1 |
| Methylthiopropionate Biosynthesis | 1.49E00 | ADI1 |
| Acetyl-CoA Biosynthesis III (from Citrate) | 1.49E00 | ACLY |
| Glutamine Biosynthesis I | 1.49E00 | GLUL |
| Methylglyoxal Degradation VI | 1.49E00 | LDHD |
| Lanosterol Biosynthesis | 1.49E00 | LSS |
| 4-hydroxybenzoate Biosynthesis | 1.49E00 | TAT |
| 4-hydroxyphenylpyruvate Biosynthesis | 1.49E00 | TAT |
| Estrogen Biosynthesis | 1.48E00 | HSD17B13, AKR1C4, CYP51A1, CYP2C8 |
| Notch Signaling | 1.48E00 | NOTCH3, LFNG, DTX2, NUMBL |
| HER-2 Signaling in Breast Cancer | 1.45E00 | PRKCE, PRKCH, PRKD3, ITGB5, PRKCG, EGFR |
| Inhibition of Matrix Metalloproteases | 1.45E00 | HSPG2, TIMP4, MMP12, LRP1 |
| Glioma Invasiveness Signaling | 1.45E00 | TIMP4, RHOC, VTN, RHOJ, ITGB5 |
| P2Y Purigenic Receptor Signaling Pathway | 1.42E00 | GNB4, GNG11, PLCE1, PRKCE, PRKCH, ADCY10, PRKD3, PRKCG |
| Phospholipases | 1.42E00 | PLCE1, LCAT, ABHD3, LIPG, PLA2G12A |
| Ketogenesis | 1.41E00 | ACAT2, HMGCS2 |
| Signaling by Rho Family GTPases | 1.4E00 | PTK2B, RHOC, RHOJ, ARHGEF17, CDH11, GNB4, CDH2, GNG11, CDC42EP1, ARHGEF2, ACTG2, ARHGEF10, MYL12B |
| Synaptic Long Term Depression | 1.4E00 | PLCE1, LCAT, ABHD3, PRKCE, PRKCH, PRKD3, GUCY1B3, PRKCG, PLA2G12A |
| ErbB4 Signaling | 1.36E00 | PRKCE, NRG4, PRKCH, PRKD3, PRKCG |
| RAR Activation | 1.36E00 | Aldh1a7, RDH11, RARA, TGFB3, PRKCE, Rdh7, PRKCH, ADCY10, PRKD3, PRKCG, GTF2H3 |
| Epithelial Adherens Junction Signaling | 1.33E00 | CDH2, NOTCH3, MAGI1, TUBA1A, FGFR1, TUBB2A, JUP, ACTG2, EGFR |
| Gαq Signaling | 1.32E00 | GNB4, GNG11, PTK2B, RHOC, PRKCE, RHOJ, PRKCH, PRKD3, PRKCG |

Supplementary Table 3. Significantly altered pathways at 30 % restriction relative to 12h *ad libitum* intake identified by Ingenuity Pathway Analysis (IPA, www.qiagen.com/ingenuity).

| **Ingenuity Canonical Pathways** | **-log(p-value)** | **Molecules** |
| --- | --- | --- |
| Oxidative Phosphorylation | 4.44E01 | NDUFA9, ATP5D, COX8A, NDUFB5, ATP5L, NDUFB8, NDUFA1, NDUFB10, COX6A2, NDUFA5, UQCRFS1, NDUFA10, ATP5G3, NDUFA8, ATP5J, ATP5G2, NDUFS7, ATP5A1, ATP5O, SDHC, UQCR11, NDUFB11, ATP5B, NDUFA6, UQCRC2, NDUFB7, NDUFA12, NDUFS3, NDUFB2, NDUFA4, COX7B, SDHB, ATP5H, Cox6c, COX6A1, Atp5e, NDUFB9, ATP5J2, NDUFS2, NDUFB6, NDUFAB1, COX4I1, NDUFS4, SDHA, NDUFV1, COX6B1, NDUFV3, UQCRB, Cox8b, NDUFS8, NDUFV2, NDUFA11, UQCR10, CYC1, COX5A, COX7A2, SDHD, CYCS, UQCRC1, UQCRQ |
| Mitochondrial Dysfunction | 4.39E01 | NDUFA9, ATP5D, COX8A, NDUFB5, ACO2, ATP5L, NDUFB8, NDUFA1, COX6A2, VDAC2, NDUFB10, PDHA1, NDUFA5, SOD2, GPD2, NDUFA10, UQCRFS1, GPX4, ATP5G3, NDUFA8, ATP5J, ATP5G2, CPT1A, NDUFS7, ATP5A1, ATP5O, UQCR11, SDHC, PRDX3, NDUFB11, ATP5B, TXN2, NDUFA6, UQCRC2, NDUFB7, NDUFA12, BACE2, NDUFS3, VDAC1, NDUFB2, NDUFA4, HSD17B10, COX7B, SDHB, ATP5H, Cox6c, COX6A1, Atp5e, BCL2, NDUFB9, ATP5J2, NDUFB6, NDUFS2, NDUFAB1, COX4I1, NDUFS4, SDHA, NDUFV1, COX6B1, NDUFV3, UQCRB, Cox8b, NDUFS8, NDUFV2, NDUFA11, UQCR10, CYC1, COX5A, COX7A2, SDHD, CYCS, UQCRC1, UQCRQ |
| LXR/RXR Activation | 1.58E01 | KNG1, APOE, SCD, APOA4, APOB, APOH, ABCG4, APOA2, APOC4, VTN, AMBP, ABCG1, C4A/C4B, PON1, FDFT1, LYZ, LCAT, FASN, SERPINA1, LBP, IL1RAP, CYP51A1, HPX, MLXIPL, TTR, ECHS1, AHSG, SERPINF1, APOA5, ALB, APOA1, SREBF1, ACACA, FGA, MMP9, HADH |
| Coagulation System | 1.06E01 | KNG1, PROC, THBD, FGG, F2, PLG, KLKB1, F10, SERPINC1, F8, F5, FGB, SERPINA1, PLAU, FGA, TFPI |
| Intrinsic Prothrombin Activation Pathway | 9.77E00 | KNG1, PROC, THBD, FGG, F2, KLKB1, F10, SERPINC1, COL5A3, F8, F5, FGB, FGA, COL18A1 |
| FXR/RXR Activation | 9.07E00 | KNG1, APOE, APOA4, APOB, SLC10A1, APOH, APOA2, APOC4, VTN, AMBP, C4A/C4B, PON1, LCAT, RARA, FASN, SERPINA1, SLCO1B3, HPX, TTR, MLXIPL, CYP27A1, AHSG, SERPINF1, ALB, APOA1, SREBF1, G6PC, FGA |
| Extrinsic Prothrombin Activation Pathway | 8.58E00 | F10, SERPINC1, F5, PROC, FGB, TFPI, THBD, FGA, FGG, F2 |
| TCA Cycle II (Eukaryotic) | 7.74E00 | SDHA, SDHB, CS, ACO2, DLD, SDHD, SDHC, IDH3A, MDH1, MDH2, IDH3B |
| Superpathway of Cholesterol Biosynthesis | 7.72E00 | MVD, FDPS, SQLE, FDFT1, NSDHL, PMVK, ACAT2, IDI1, LSS, HMGCS2, TM7SF2, CYP51A1 |
| Acute Phase Response Signaling | 7.35E00 | ECSIT, ITIH3, FN1, APOH, APOA2, AMBP, SERPINA3, F2, FGG, C4A/C4B, SOD2, JUN, F8, ITIH2, PIK3CG, SERPINA1, FGB, LBP, IL1RAP, HPX, TTR, AHSG, SERPINF1, C5, PLG, KLKB1, ALB, APOA1, MAP2K3, FGA |
| Hepatic Fibrosis / Hepatic Stellate Cell Activation | 5.55E00 | MYH10, CCR5, FN1, LEP, COL4A5, MYL2, COL8A1, COL6A6, COL15A1, BCL2, COL5A1, LBP, COL18A1, IL1RAP, MYL3, EGFR, MYH1, COL6A2, FGFR1, VEGFC, MYH7, COL5A3, MYH2, COL6A3, CSF1, TGFB3, COL6A5, MMP9 |
| Valine Degradation I | 5.46E00 | HIBCH, ECHS1, ABAT, HIBADH, BCKDHA, DLD, ACADSB, EHHADH |
| Glycolysis I | 5.17E00 | PGK1, ENO1, ALDOB, ENO3, PKM, ALDOA, GAPDH, PFKL, PFKM |
| Gluconeogenesis I | 5.17E00 | PGK1, ENO1, ALDOB, ENO3, ALDOA, GAPDH, ME1, MDH1, MDH2 |
| Tryptophan Degradation III (Eukaryotic) | 4.86E00 | HSD17B10, KMO, ACAT2, TDO2, HAAO, IDO1, EHHADH, HADH |
| Cholesterol Biosynthesis I | 4.32E00 | SQLE, FDFT1, NSDHL, LSS, TM7SF2, CYP51A1 |
| Cholesterol Biosynthesis II (via 24, 25-dihydrolanosterol) | 4.32E00 | SQLE, FDFT1, NSDHL, LSS, TM7SF2, CYP51A1 |
| Cholesterol Biosynthesis III (via Desmosterol) | 4.32E00 | SQLE, FDFT1, NSDHL, LSS, TM7SF2, CYP51A1 |
| Isoleucine Degradation I | 4.1E00 | HSD17B10, ECHS1, ACAT2, DLD, ACADSB, EHHADH |
| Protein Kinase A Signaling | 4.09E00 | AKAP12, FLNB, MYH10, DUSP8, TNNI2, MYL2, PTK2B, MYLK2, TIMM50, ROCK2, PTPRC, AKAP2, GNB4, GNG11, DUSP3, PLCE1, DUSP10, H1FX, SMO, PRKCE, RYR1, VASP, MYL3, PTPRG, MYLPF, CHP1, PYGL, PYGB, CREB5, TTN, ROCK1, PPP1R3D, Hist1h1e, MYH2, PYGM, PRKACA, TGFB3, DUSP18, TNNI1, ADCY10, ADCY7, DUSP16 |
| LPS/IL-1 Mediated Inhibition of RXR Function | 4.08E00 | ECSIT, APOE, ALDH4A1, SLC10A1, APOC4, ABCG1, HMGCS2, GSTT2/GSTT2B, JUN, CYP3A5, RARA, Gm4846, FABP1, FMO1, FABP5, LBP, IL1RAP, SLCO1B3, ACSL3, CPT1A, ALDH9A1, HS3ST3B1, SULT1E1, MGST2, SREBF1, SLC27A1, ACSL1, CYP2C8 |
| Calcium Signaling | 3.95E00 | MYH10, CAMK1, TP63, MYL2, CAMK1D, TNNI2, TNNT3, TNNC2, TNNC1, CHP1, TRDN, MYH7, CREB5, ATP2A1, MYH2, CHRNB2, CASQ1, PRKACA, RYR1, TNNI1, MYL3, ACTA1, MYH1, GRIA3 |
| Pentose Phosphate Pathway | 3.88E00 | PGD, TKT, PGLS, TALDO1, G6PD |
| Superpathway of Geranylgeranyldiphosphate Biosynthesis I (via Mevalonate) | 3.55E00 | MVD, FDPS, PMVK, ACAT2, IDI1, HMGCS2 |
| Fatty Acid β-oxidation I | 3.5E00 | HSD17B10, ACSL3, ECHS1, SLC27A1, EHHADH, ECI1, ACSL1, HADH |
| Atherosclerosis Signaling | 3.5E00 | APOE, APOA4, APOB, APOC4, APOA2, SELPLG, PON1, COL5A3, ALB, LYZ, APOA1, LCAT, CSF1, SERPINA1, COL18A1, MMP9, ITGA4, PLA2G12A |
| Acetyl-CoA Biosynthesis I (Pyruvate Dehydrogenase Complex) | 3.45E00 | PDHA1, DLAT, DLD, PDHB |
| Actin Cytoskeleton Signaling | 3.39E00 | KNG1, MYH10, FN1, MYL2, MYLK2, Actn3, PIP4K2B, TLN1, F2, ROCK2, PIK3CG, LBP, MYL3, ACTA1, ITGA4, MYH1, TIAM1, MYLPF, ACTN2, FGF14, MYH7, TTN, ROCK1, MYH2, TLN2, PIP4K2A |
| Mevalonate Pathway I | 3.23E00 | MVD, PMVK, ACAT2, IDI1, HMGCS2 |
| Tryptophan Degradation to 2-amino-3-carboxymuconate Semialdehyde | 3.17E00 | KMO, TDO2, HAAO, IDO1 |
| Pentose Phosphate Pathway (Oxidative Branch) | 3.12E00 | PGD, PGLS, G6PD |
| Stearate Biosynthesis I (Animals) | 3.03E00 | ACSL3, ACOT2, FASN, ACOT1, ACOT4, SLC27A1, ACSL1, ELOVL6 |
| TR/RXR Activation | 2.9E00 | F10, ENO1, COL6A3, SREBF1, FASN, PIK3CG, APOA5, G6PC, ACACA, ME1, FGA, THRSP, ATP2A1 |
| ILK Signaling | 2.88E00 | FLNB, MYH10, SNAI2, FN1, MYL2, RHOC, ACTN2, PPP2R5B, Actn3, VEGFC, MYH7, CREB5, TGFB1I1, MYH2, JUN, PIK3CG, SH2B2, ACTA1, MYL3, MMP9, ITGB5, MYH1 |
| Oxidative Ethanol Degradation III | 2.76E00 | ALDH4A1, ACSL3, ACSS2, ALDH9A1, ACSL1 |
| Acetate Conversion to Acetyl-CoA | 2.74E00 | ACSL3, ACSS2, ACSL1 |
| Glycogen Degradation II | 2.74E00 | PYGM, PYGB, PYGL, AGL |
| Epithelial Adherens Junction Signaling | 2.66E00 | MYH10, SNAI2, MYL2, ACTN2, FGFR1, Actn3, TUBA4A, MYH7, CDH2, TUBA1A, MYH2, MAGI1, TUBA8, JUP, ACTA1, MYL3, MYH1, EGFR |
| Clathrin-mediated Endocytosis Signaling | 2.56E00 | APOE, APOA4, APOB, EPHB2, APOC4, APOA2, CHP1, FGF14, VEGFC, HIP1, F2, PON1, LYZ, ALB, ARRB1, APOA1, PIK3CG, TFRC, SERPINA1, ITGB5, ACTA1 |
| Epoxysqualene Biosynthesis | 2.47E00 | SQLE, FDFT1 |
| Zymosterol Biosynthesis | 2.46E00 | NSDHL, TM7SF2, CYP51A1 |
| Triacylglycerol Biosynthesis | 2.44E00 | GPAM, ABHD5, AGPAT2, PPAP2B, AGPAT1, DGAT1, ELOVL6 |
| Glutaryl-CoA Degradation | 2.4E00 | HSD17B10, ACAT2, EHHADH, HADH |
| Glycogen Degradation III | 2.4E00 | PYGM, PYGB, PYGL, AGL |
| Ethanol Degradation IV | 2.3E00 | ALDH4A1, ACSL3, ACSS2, ALDH9A1, ACSL1 |
| Estrogen Biosynthesis | 2.23E00 | HSD17B10, HSD17B13, CYP3A5, HSD17B12, AKR1C4, CYP51A1, CYP2C8 |
| Thrombin Signaling | 2.08E00 | CAMK1, MYL2, CAMK1D, RHOC, MYLPF, F2, ROCK2, ROCK1, GNB4, GNG11, PLCE1, PIK3CG, GNAO1, PRKCE, ARHGEF2, ADCY10, ADCY7, ARHGEF10, MYL3, EGFR |
| NAD biosynthesis II (from tryptophan) | 2.03E00 | KMO, TDO2, HAAO, IDO1 |
| Coenzyme A Biosynthesis | 2.01E00 | PPCS, COASY |
| Thiosulfate Disproportionation III (Rhodanese) | 2.01E00 | MPST, TST |
| Glycerol-3-phosphate Shuttle | 2.01E00 | GPD1, GPD2 |
| Fatty Acid β-oxidation III (Unsaturated, Odd Number) | 2.01E00 | EHHADH, ECI1 |
| Estrogen-Dependent Breast Cancer Signaling | 1.97E00 | HSD17B10, HSD17B13, JUN, PIK3CG, HSD17B12, AKR1C4, CREB5, ESR1, EGFR |
| Ethanol Degradation II | 1.95E00 | HSD17B10, ALDH4A1, ACSL3, ACSS2, ALDH9A1, ACSL1 |
| Cardiac Hypertrophy Signaling | 1.94E00 | MAP3K15, CACNA1S, MYL2, RHOC, MYLPF, CHP1, ROCK2, ROCK1, GNB4, GNG11, JUN, PLCE1, PIK3CG, GNAO1, EIF2B1, PRKACA, TGFB3, MAP2K3, ADCY10, ADCY7, MYL3, ADRB2 |
| Role of Tissue Factor in Cancer | 1.92E00 | F10, ARRB1, PTK2B, CSF1, PIK3CG, RPS6KA3, VEGFC, FGB, FGA, ITGB5, F2, FGG, EGFR |
| CDP-diacylglycerol Biosynthesis I | 1.92E00 | GPAM, ABHD5, AGPAT2, AGPAT1 |
| RhoGDI Signaling | 1.9E00 | MYL2, RHOC, MYLPF, PIP4K2B, CDH11, ROCK2, ROCK1, GNB4, CDH2, GNG11, GNAO1, ARHGEF2, PIP4K2A, ARHGEF10, ESR1, ACTA1, MYL3, ITGA4 |
| B Cell Development | 1.89E00 | PTPRC, HLA-DQA1, HLA-DOB, CD79A, HLA-DQB1, HLA-DRB5 |
| Axonal Guidance Signaling | 1.87E00 | ADAM17, RGS3, UNC5A, MYL2, EPHB2, ADAMTS2, SEMA4C, ROCK2, GNB4, PLCE1, GNG11, TUBA8, ADAM28, PIK3CG, SMO, PRKCE, SRGAP2, ADAMTS5, VASP, MYL3, ITGA4, PLXNA1, PLXNC1, ADAM15, SEMA5A, MYLPF, CHP1, TUBA4A, VEGFC, PLXND1, ROCK1, NTRK2, TUBA1A, FZD4, GNAO1, PRKACA, MMP9 |
| γ-linolenate Biosynthesis II (Animals) | 1.82E00 | ACSL3, SLC27A1, ACSL1, FADS1 |
| Mitochondrial L-carnitine Shuttle Pathway | 1.82E00 | ACSL3, CPT1A, SLC27A1, ACSL1 |
| PXR/RXR Activation | 1.81E00 | SCD, Aldh1a7, CPT1A, CYP3A5, PRKACA, G6PC, HMGCS2, SLCO1B3, CYP2C8 |
| Reelin Signaling in Neurons | 1.78E00 | APOE, PIK3CG, CNR1, MAP4K1, ITGA1, ARHGEF2, MAPK8IP1, ARHGEF10, ITGAL, ITGA4 |
| Breast Cancer Regulation by Stathmin1 | 1.78E00 | CAMK1, CAMK1D, PPP2R5B, TUBA4A, ROCK2, ROCK1, GNB4, CCNE1, PPP1R3D, TUBA1A, GNG11, TUBA8, PIK3CG, PRKACA, PRKCE, ARHGEF2, ADCY10, ARHGEF10, ADCY7 |
| Phosphatidylglycerol Biosynthesis II (Non-plastidic) | 1.74E00 | GPAM, ABHD5, AGPAT2, AGPAT1 |
| Tight Junction Signaling | 1.73E00 | MYH10, TIAM1, MYL2, PPP2R5B, MYH7, MPDZ, JUN, MYH2, TGFB3, PRKACA, ARHGEF2, NAPA, ACTA1, MYL3, VASP, CLDN22, MYH1 |
| Branched-chain α-keto acid Dehydrogenase Complex | 1.72E00 | BCKDHA, DLD |
| Biotin-carboxyl Carrier Protein Assembly | 1.72E00 | ACACB, ACACA |
| Phenylalanine Degradation I (Aerobic) | 1.72E00 | PAH, QDPR |
| Signaling by Rho Family GTPases | 1.72E00 | NOX4, PTK2B, MYL2, RHOC, MYLPF, PIP4K2B, CDH11, ROCK2, ROCK1, GNB4, CDH2, JUN, GNG11, PIK3CG, GNAO1, CDC42EP1, ARHGEF2, PIP4K2A, ARHGEF10, MYL3, ACTA1, ITGA4 |
| Antigen Presentation Pathway | 1.71E00 | PSMB5, HLA-DQA1, CIITA, HLA-DOB, CD74, HLA-DRB5 |
| Complement System | 1.71E00 | C4A/C4B, CD55, C7, C1QBP, C6, C5 |
| Caveolar-mediated Endocytosis Signaling | 1.66E00 | FLNB, ALB, CD55, ITGA1, ITGAL, ITGB5, ACTA1, ITGA4, EGFR |
| Glutathione Redox Reactions I | 1.65E00 | GSTT2/GSTT2B, MGST2, GPX5, GPX4 |
| IL-8 Signaling | 1.65E00 | NOX4, PTK2B, MYL2, RHOC, VEGFC, BCL2, ROCK2, ROCK1, GNB4, GNG11, JUN, PIK3CG, PRKCE, MMP9, VASP, ITGB5, LASP1, EGFR |
| Gαs Signaling | 1.6E00 | GNB4, RGS2, GNG11, CNR1, PTH1R, PRKACA, RYR1, RAPGEF3, ADCY10, CREB5, ADCY7, ADRB2 |
| RhoA Signaling | 1.58E00 | PLXNA1, MYL2, PTK2B, MYLPF, MYLK2, PIP4K2B, TTN, ROCK2, ROCK1, CDC42EP1, PIP4K2A, ACTA1, MYL3 |
| IL-12 Signaling and Production in Macrophages | 1.58E00 | PON1, APOE, LYZ, ALB, APOA1, JUN, APOB, APOA4, PIK3CG, APOA2, APOC4, TGFB3, PRKCE, SERPINA1 |
| Neuroprotective Role of THOP1 in Alzheimer's Disease | 1.56E00 | KNG1, PLG, PRKACA, SERPINA3, IDE, MMP9 |
| Acyl-CoA Hydrolysis | 1.53E00 | ACOT2, ACOT1, ACOT4 |
| Serine Biosynthesis | 1.52E00 | PSPH, PHGDH |
| Trans, trans-farnesyl Diphosphate Biosynthesis | 1.52E00 | FDPS, IDI1 |
| Tyrosine Degradation I | 1.52E00 | HPD, TAT |
| cAMP-mediated signaling | 1.49E00 | AKAP12, GRK4, RGS2, CAMK1, RGS18, CAMK1D, PTGER3, CNR1, RAPGEF3, CREB5, S1PR3, AKAP2, NPR3, GNAO1, PTH1R, PRKACA, ADORA1, ADCY10, ADCY7, ADRB2 |
| PPARα/RXRα Activation | 1.48E00 | GPD1, APOA2, Cyp2c44, JUN, PLCE1, APOA1, GPD2, FASN, TGFB3, PRKACA, MAP2K3, SLC27A1, ADCY10, IL1RAP, ADCY7, ITGB5, CYP2C8 |
| Paxillin Signaling | 1.47E00 | TLN2, PTK2B, PIK3CG, ACTN2, Actn3, TLN1, ITGA1, ITGAL, ITGB5, ACTA1, ITGA4 |
| Virus Entry via Endocytic Pathways | 1.46E00 | FLNB, CD55, PIK3CG, PRKCE, TFRC, ITGA1, ITGAL, ITGB5, ACTA1, ITGA4 |
| NRF2-mediated Oxidative Stress Response | 1.46E00 | SOD1, DNAJC15, CLPP, GSTT2/GSTT2B, SOD2, JUN, MGST2, PIK3CG, PRKCE, DNAJA3, MAP2K3, FMO1, CDC34, GCLM, FKBP5, ACTA1, CBR1 |
| Production of Nitric Oxide and Reactive Oxygen Species in Macrophages | 1.46E00 | MAP3K15, APOE, APOA4, APOB, RHOC, APOC4, APOA2, PPP2R5B, PON1, ALB, LYZ, PPP1R3D, JUN, APOA1, PIK3CG, PRKCE, SERPINA1 |
| Maturity Onset Diabetes of Young (MODY) Signaling | 1.44E00 | CACNA1S, ALDOB, GAPDH, FABP1 |
| Fatty Acid Activation | 1.44E00 | ACSL3, SLC27A1, ACSL1 |
| Cellular Effects of Sildenafil (Viagra) | 1.41E00 | MYH10, CACNA1S, MYH2, PLCE1, MYL2, MYLPF, PRKACA, MYH7, ADCY10, ADCY7, ACTA1, MYL3, MYH1 |
| IL-1 Signaling | 1.4E00 | ECSIT, GNB4, GNG11, JUN, GNAO1, PRKACA, MAP2K3, ADCY10, ADCY7, IL1RAP |
| Phospholipase C Signaling | 1.39E00 | MYL2, RHOC, MYLPF, CHP1, RPS6KA3, RAPGEF3, CD79A, CREB5, GNB4, GNG11, PLCE1, AHNAK, PRKCE, ARHGEF2, ADCY10, ARHGEF10, ADCY7, LCP2, MYL3, ITGA4, PLA2G12A |
| VEGF Signaling | 1.38E00 | ROCK2, ROCK1, PTK2B, PIK3CG, ACTN2, EIF2B1, Actn3, VEGFC, ACTA1, BCL2 |
| Leukocyte Extravasation Signaling | 1.38E00 | PTK2B, ACTN2, Actn3, THY1, RAPGEF3, ITGAL, SELPLG, ROCK2, ROCK1, PIK3CG, SIPA1, PRKCE, ITGA1, ACTA1, MMP9, VASP, CLDN22, ITGA4 |
| Urea Cycle | 1.36E00 | ASS1, CPS1 |
| Pentose Phosphate Pathway (Non-oxidative Branch) | 1.36E00 | TKT, TALDO1 |
| Glycerol Degradation I | 1.36E00 | GPD1, GPD2 |
| Glycogen Biosynthesis II (from UDP-D-Glucose) | 1.36E00 | GYG1, GBE1 |
| NAD Biosynthesis III | 1.36E00 | NAMPT, Nmnat3 |
| Superpathway of Citrulline Metabolism | 1.35E00 | ASS1, GLS2, CPS1 |
| Glioma Invasiveness Signaling | 1.32E00 | PLG, RHOC, PIK3CG, VTN, PLAU, ITGB5, MMP9 |
| Nur77 Signaling in T Lymphocytes | 1.32E00 | CHP1, HLA-DQA1, HLA-DOB, CYCS, HLA-DQB1, HLA-DRB5, BCL2 |
| Regulation of Cellular Mechanics by Calpain Protease | 1.32E00 | CCNE1, TLN2, ACTN2, Actn3, TLN1, ITGA4, EGFR |

Supplementary Table 4. Significantly altered pathways at 40 % restriction relative to 12h *ad libitum* intake identified by Ingenuity Pathway Analysis (IPA, www.qiagen.com/ingenuity).

| **Ingenuity Canonical Pathways** | **-log(p-value)** | **Molecules** |
| --- | --- | --- |
| Mitochondrial Dysfunction | 3.21E01 | MAP2K4, NDUFA9, ATP5D, COX8A, NDUFB5, ACO2, ATP5L, NDUFB8, NDUFA4L2, NDUFA1, VDAC2, COX6A2, NDUFB10, PDHA1, NDUFA5, NDUFS1, SOD2, GPD2, NDUFS6, UQCRFS1, NDUFA10, GPX4, ATP5F1, ATP5G3, NDUFA8, ATP5J, ATP5G2, CPT1A, CASP3, NDUFS7, ATP5A1, ATP5O, BACE1, UQCR11, SDHC, GPX7, GSR, ATP5C1, PRDX3, NDUFB11, ATP5B, TXN2, NDUFA6, UQCRC2, NDUFB7, NDUFA12, BACE2, VDAC1, NDUFS3, NDUFB2, HSD17B10, NDUFA4, SDHB, COX7B, ATP5G1, ATP5H, COX6A1, Cox6c, NDUFA7, COX10, Atp5e, NDUFB9, PARK7, ATP5J2, NDUFB6, NDUFS2, CPT1C, NDUFAB1, COX4I1, AIFM1, NDUFS4, SDHA, NDUFV1, COX6B1, NDUFV3, VDAC3, APP, UQCRB, Cox8b, NDUFS8, NDUFV2, NDUFA11, UQCR10, CYC1, COX5A, COX7A2, SDHD, CYCS, UQCRC1, UQCRQ, MAOA |
| Oxidative Phosphorylation | 2.89E01 | NDUFA9, ATP5D, COX8A, NDUFB5, ATP5L, NDUFB8, NDUFA1, COX6A2, NDUFB10, NDUFS1, NDUFA5, NDUFS6, NDUFA10, UQCRFS1, ATP5F1, ATP5G3, NDUFA8, ATP5J, ATP5G2, NDUFS7, ATP5A1, ATP5O, SDHC, UQCR11, ATP5C1, NDUFB11, ATP5B, NDUFA6, UQCRC2, NDUFB7, NDUFA12, NDUFS3, NDUFB2, NDUFA4, ATP5G1, COX7B, SDHB, ATP5H, NDUFA7, Cox6c, COX6A1, COX10, Atp5e, NDUFB9, ATP5J2, NDUFS2, NDUFB6, NDUFAB1, COX4I1, NDUFS4, SDHA, NDUFV1, COX6B1, NDUFV3, UQCRB, Cox8b, NDUFS8, NDUFV2, NDUFA11, UQCR10, CYC1, COX5A, COX7A2, SDHD, CYCS, UQCRC1, UQCRQ |
| Hepatic Fibrosis / Hepatic Stellate Cell Activation | 1.43E01 | MYH4, MYH10, CCR5, MYH9, MYL2, SMAD3, COL8A1, MYL6B, VEGFA, TGFB1, CYP2E1, FIGF, SERPINE1, IL1RAP, MYL3, PDGFRB, TIMP2, COL4A1, FGFR1, VEGFC, MYH7, MMP2, COL6A3, CD40, TGFB3, MYH3, IL10RA, IGFBP3, COL6A5, PDGFD, IFNAR1, COL3A1, COL8A2, RELA, COL4A5, FN1, LEP, COL4A6, MYH8, COL4A2, CCL5, NFKB1, PDGFC, COL15A1, COL6A6, COL1A2, COL16A1, COL5A1, COL6A1, CCL2, IGF1, NGFR, LBP, COL18A1, MYH1, EGFR, COL5A2, COL6A2, TNFRSF1A, FLT4, SMAD7, IL1R1, MYL1, COL1A1, COL5A3, MYH2, CSF1, COL24A1, MMP9 |
| Axonal Guidance Signaling | 1.25E01 | SLIT3, DPYSL2, RAC2, GLI2, ITSN1, PIK3R1, BRCC3, ADAMTS2, VEGFA, GNB4, PAK1, GNA15, PIK3CG, FIGF, SRGAP2, PLXNB2, MYL3, PTCH2, ITGA4, COPS5, SEMA5A, TUBB2A, ITGA5, MMP2, L1CAM, DOCK1, PLCG2, PRKACA, PIK3CD, PDGFD, NRP1, FYN, LRRC4C, ADAM17, RGS3, PLXNA3, UNC5A, FZD1, GNG7, PLCD1, EFNB2, TUBA8, IGF1, EFNA5, NGFR, PRKCE, PSMD14, RASSF5, MYL12B, BMP1, ITGB1, SEMA3G, PLXNA1, EPHB4, NRP2, MYLPF, SLIT2, MYL1, PRKCG, WIPF1, TUBA1A, KIF7, GLIS2, WAS, SEMA4G, BMP6, PFN1, MYL2, EPHB2, FZD3, UNC5B, GNB5, MYL6B, LIMK2, PIK3R4, LIMK1, ROCK2, MICAL1, PLCE1, SEMA3D, CFL2, SUFU, ADAM28, FZD2, ADAMTS5, PAPPA, FES, ADAMTS1, VEGFC, ADAM12, GNAO1, PLCB3, PDGFC, BCAR1, ROBO1, PRKAG1, SEMA4C, GNG11, GLI3, PPP3CB, EFNB1, SMO, SEMA3B, VASP, PLXNC1, ARPC5L, ADAM15, CHP1, TUBG1, TUBA4A, NFATC4, PLXND1, GNAI2, NTRK2, FZD4, EPHB3, PIK3CB, ADAM9, MMP9, WNT5A |
| Actin Cytoskeleton Signaling | 1.12E01 | KNG1, MYH4, MYH10, RAC2, MYH9, PFN1, MYL2, F2R, PIK3R1, MYLK2, PIP4K2B, TLN1, LIMK2, MYL6B, PIK3R4, SLC9A1, F2, LIMK1, ROCK2, PAK1, CFL2, PIK3CG, ACTA1, MYL3, IQGAP3, ITGA4, TIAM1, ITGA5, MYH7, FGD1, GSN, TTN, DOCK1, TLN2, PIP5K1C, MYH3, PIK3CD, VAV1, PDGFD, ACTG1, PIP4K2A, FGD3, FN1, MYH8, Actn3, IQGAP1, PDGFC, BCAR1, FGF13, DIAPH1, FLNA, LBP, VCL, MYL12B, MYH1, ITGB1, ARPC5L, ACTN2, CSK, MYLPF, ACTB, FGF14, MYL1, MYH2, APC2, WAS, VAV3, FGF11, PIK3CB, NCKAP1L, MSN |
| Coagulation System | 1.02E01 | KNG1, F2R, PROC, VWF, THBD, F3, F2, FGG, PLG, KLKB1, F10, SERPINC1, F8, PROS1, F5, SERPINA1, FGB, PLAU, FGA, SERPINE1, TFPI, PLAT |
| ILK Signaling | 9.28E00 | MAP2K4, MYH4, MYH10, MYH9, MYL2, PIK3R1, PPP2R5B, MYL6B, PIK3R4, CCND1, VEGFA, CFL2, PIK3CG, FIGF, ACTA1, ITGB5, MYL3, CASP3, VEGFC, MYH7, DOCK1, RND3, PPP2R3A, IRS1, MYH3, PIK3CD, RSU1, ACTG1, FLNB, RELA, FN1, MYH8, Actn3, HIF1A, NFKB1, PDGFC, ITGB7, TGFB1I1, JUN, RHOT1, FLNA, VCL, NACA, MYH1, ITGB1, PARVA, FBLIM1, LIMS2, TNFRSF1A, RHOC, ACTN2, ACTB, VIM, MYL1, ITGB2, MYH2, SH2B2, PIK3CB, RPS6KA4, MMP9 |
| Leukocyte Extravasation Signaling | 8.98E00 | MAP2K4, RAC2, PIK3R1, MLLT4, PIK3R4, ROCK2, CYBA, PIK3CG, ACTA1, ITGA4, TIMP2, ITGA5, THY1, MMP2, RAPGEF3, NCF4, TIMP4, ITGAM, MMP23B, CDH5, JAM3, PLCG2, PECAM1, PIK3CD, VAV1, ITGA1, ACTG1, ARHGAP1, SPN, PTK2B, MMP15, Actn3, BCAR1, SIPA1, PRKCE, RASSF5, VCL, MMP12, VASP, ITGB1, TIMP3, SRC, ARHGAP6, ACTB, ACTN2, ARHGAP4, ITGAL, SELPLG, PRKCG, GNAI2, ITGB2, F11R, WIPF1, CLDN5, WAS, VAV3, CD44, PIK3CB, CLDN22, MMP9, MSN, CTNND1 |
| Intrinsic Prothrombin Activation Pathway | 8.31E00 | KNG1, PROC, THBD, F2, FGG, COL1A2, KLKB1, COL1A1, F10, SERPINC1, COL5A3, F8, PROS1, F5, FGB, FGA, COL18A1, COL3A1 |
| Acute Phase Response Signaling | 8.26E00 | MAP2K4, ITIH3, APOA2, PIK3R1, SOCS6, FGG, F2, SOD2, ITIH2, PIK3CG, FGB, SERPINE1, IL1RAP, TTR, TCF3, C5, IL33, KLKB1, IL18, PIK3CD, FGA, SOCS5, C2, ECSIT, RELA, SERPING1, FN1, APOH, NFKBIE, AMBP, NFKB1, C1R, C4A/C4B, TRADD, NFKBIA, JUN, F8, NGFR, CFB, SOCS2, SERPINA1, LBP, HPX, TNFRSF1A, AHSG, SERPINF1, VWF, IL1R1, PLG, ALB, APOA1, RBP7, PIK3CB, ELK1 |
| LXR/RXR Activation | 8.25E00 | KNG1, APOE, SCD, RELA, APOA4, APOB, APOH, ABCG4, APOA2, VTN, AMBP, NFKB1, C4A/C4B, PON1, FDFT1, LCAT, CCL2, FASN, NGFR, SERPINA1, TLR3, LBP, IL1RAP, CYP51A1, HPX, TTR, MLXIPL, ECHS1, TNFRSF1A, AHSG, SERPINF1, IL1R1, IL33, ALB, IL18, APOA1, SREBF1, ACACA, PLTP, NCOR2, FGA, MMP9, HADH |
| Superpathway of Cholesterol Biosynthesis | 7.67E00 | MVD, SQLE, NSDHL, PMVK, ACAT2, IDI1, HSD17B7, HMGCS2, TM7SF2, SC5D, FDPS, FDFT1, DHCR7, HADHB, LSS, HADHA, CYP51A1 |
| Protein Kinase A Signaling | 7.47E00 | MYH4, MYH10, DUSP8, MYL2, PTPN23, SMAD3, MYLK2, TIMM50, GNB5, MYL6B, PPP1R3A, PTPN5, ROCK2, PTPRC, GNB4, PLCE1, DUSP3, TGFB1, PGP, PDE11A, PPP1CA, APEX1, MYL3, PTCH2, CDC25A, PTPN6, PTPRE, PTPRG, ITPR2, HIST1H3C, PTPN18, ANAPC7, PDE4B, TCF3, TTN, PYGM, DUSP9, PLCG2, TGFB3, PRKACA, PTPRS, PLCB3, ADCY10, EBI3, PTPRA, SIRPA, ANAPC2, AKAP12, FLNB, RELA, Calm1 (includes others), TNNI2, PTK2B, PTPN9, NFKBIE, NFKB1, GNG7, PRKAG1, PLCD1, AKAP2, PTPN4, GNG11, NFKBIA, PPP3CB, GLI3, PTPRJ, FLNA, NGFR, DUSP10, SMO, PRKCE, RYR1, VASP, MYL12B, PTPN7, PTPRK, ATF1, PDE9A, MYLPF, CHP1, PYGL, AKAP6, PYGB, NFATC4, MYL1, PRKCG, GNAI2, PPP1R3D, Hist1h1e, MYH2, ADD1, DUSP18, TNNI1, ELK1, ADCY7, DUSP16 |
| Extrinsic Prothrombin Activation Pathway | 7.08E00 | F10, SERPINC1, PROS1, F5, PROC, FGB, TFPI, THBD, FGA, F3, FGG, F2 |
| Atherosclerosis Signaling | 6.89E00 | APOE, RELA, APOB, APOA4, APOA2, CCR2, NFKB1, PDGFC, COL1A2, PON1, LCAT, CCL2, TGFB1, TNFSF12, SERPINA1, COL18A1, PLA2G12A, ITGA4, PNPLA8, ABHD3, CCL11, F3, TNFRSF14, SELPLG, GLG1, IL33, PLA2G4A, COL1A1, ITGB2, COL5A3, ALB, IL18, APOA1, PLA2G2E, PLA2G2D, SELP, CD40, CSF1, PDGFD, MMP9, COL3A1 |
| Agranulocyte Adhesion and Diapedesis | 6.84E00 | MYH4, MYH10, MYH9, MYL2, MYL6B, Cxcl9, CXCL10, CXCL13, CCL25, MYL3, ACTA1, ITGA4, PF4, ITGA5, MYH7, MMP2, C5, IL33, IL18, MMP23B, CDH5, SELP, JAM3, MYH3, PECAM1, ITGA1, ACTG1, CD34, AOC3, FN1, MYH8, MMP15, CCL22, CCL5, ITGB7, ICAM2, CCL2, MMP12, MYH1, ITGB1, TNFRSF1A, ACTB, IL1R1, CCL11, MYL1, GLG1, SELPLG, GNAI2, CXCL16, ITGB2, CLDN5, MYH2, CLDN22, MMP9, MSN |
| CD28 Signaling in T Helper Cells | 6.59E00 | MAP2K4, FYN, RELA, HLA-DOA, Calm1 (includes others), PIK3R1, NFKBIE, HLA-DQA1, HLA-DQB1, NFKB1, PIK3R4, PTPRC, PAK1, NFKBIA, JUN, PPP3CB, HLA-DMA, PIK3CG, HLA-DMB, PTPN6, ARPC5L, ITPR2, CSK, CHP1, NFATC4, MALT1, CD3G, CD80, CARD11, WAS, SYK, FCER1G, HLA-DOB, CD86, PIK3CB, VAV1, PIK3CD, LCP2, HLA-DRB5 |
| Clathrin-mediated Endocytosis Signaling | 6.38E00 | APOB, EPS15, F2R, EPHB2, APOA2, PIK3R1, CLTB, Ubb, SH3GLB1, PIK3R4, F2, VEGFA, PON1, PIK3CG, AMPH, FIGF, ACTA1, ITGB5, VEGFC, DNM3, ITGA5, SH3GL1, PIP5K1C, TFRC, PIK3CD, PDGFD, ACTG1, APOE, APOA4, PDGFC, ITGB7, FGF13, ARRB1, PPP3CB, IGF1, DAB2, SERPINA1, CSNK2B, ITGB1, SRC, ARPC5L, ACTB, CHP1, FGF14, HIP1, DNM1, ITGB2, CSNK2A2, ALB, APOA1, PIK3CB, FGF11, MYO1E |
| Caveolar-mediated Endocytosis Signaling | 6.1E00 | FLNB, FYN, ITSN1, ITGB7, CD55, ITGA11, FLNA, ITGA9, CAV1, ACTA1, ITGB5, ITGA4, EGFR, ITGB1, SRC, ACTB, CD48, ITGA5, FLOT1, ITGAL, ITGB2, ALB, ITGAM, ITGA1, PTRF, ACTG1, ITGAX |
| Paxillin Signaling | 6.04E00 | MAP2K4, PTK2B, PIK3R1, Actn3, TLN1, PIK3R4, BCAR1, ITGB7, PAK1, ITGA9, ITGA11, PIK3CG, VCL, ITGB5, ACTA1, ITGA4, ITGB1, SRC, PARVA, ACTB, CSK, ACTN2, ITGA5, ITGAL, GIT2, DOCK1, ITGB2, ITGAM, TLN2, PIK3CB, ITGA1, PIK3CD, ACTG1, ITGAX |
| Signaling by Rho Family GTPases | 5.7E00 | MAP2K4, MYL2, PIK3R1, GNB5, PIP4K2B, MYL6B, LIMK2, PIK3R4, SLC9A1, LIMK1, ROCK2, GNB4, PAK1, CFL2, GNA15, PIK3CG, CDC42EP1, ACTA1, MYL3, ITGA4, NOX4, ITGA5, ARHGEF17, DES, PKN1, MAP3K12, CDH2, CDH5, RND3, PIP5K1C, GNAO1, PIK3CD, PARD3, ACTG1, PIP4K2A, ARHGEF10, RELA, PTK2B, IQGAP1, NFKB1, CDH11, GNG7, JUN, GNG11, RHOT1, ARHGEF2, ARHGEF3, MYL12B, ITGB1, ARPC5L, RHOC, MYLPF, ACTB, VIM, MYL1, GNAI2, WIPF1, WAS, PIK3CB, ELK1, MSN |
| Epithelial Adherens Junction Signaling | 5.65E00 | MYH10, MYH4, MYH9, MYL2, MYH8, MYO7A, TGFBR3, Actn3, MLLT4, MYL6B, IQGAP1, TUBA8, PVRL1, JUP, VCL, ACTA1, MYL3, EGFR, MYH1, SRC, DLL1, NOTCH3, ARPC5L, ACTN2, FGFR1, ACTB, TUBB2A, TUBG1, TUBA4A, MYH7, TCF3, MYL1, CDH2, MAGI1, MYH2, TUBA1A, WAS, MYH3, ZYX, PARD3, ACTG1, PVRL2, CTNND1 |
| TCA Cycle II (Eukaryotic) | 5.5E00 | SDHA, SDHB, CS, SUCLG1, DLST, ACO2, DLD, SDHD, SDHC, IDH3A, MDH2, MDH1, IDH3B |
| Virus Entry via Endocytic Pathways | 5.42E00 | RAC2, FYN, FLNB, ITSN1, PIK3R1, CLTB, PIK3R4, ITGB7, CD55, FLNA, PIK3CG, CAV1, PRKCE, ITGB5, ACTA1, ITGA4, ITGB1, SRC, ACTB, ITGA5, ITGAL, PRKCG, DNM1, ITGB2, PLCG2, TFRC, PIK3CB, ITGA1, PIK3CD, ACTG1 |
| Complement System | 5.36E00 | SERPING1, C1QA, C1QB, C5, C4A/C4B, C1R, ITGB2, CD55, ITGAM, C7, CFB, CFH, C6, C1QBP, C8G, C2, ITGAX |
| Valine Degradation I | 5.18E00 | HIBCH, ECHS1, BCAT2, ABAT, HIBADH, BCKDHA, DLD, HADHB, ACADSB, EHHADH, HADHA |
| PI3K Signaling in B Lymphocytes | 5.16E00 | CD81, FYN, RELA, Calm1 (includes others), ATF5, PIK3R1, NFKBIE, NFKB1, PTPRC, PLCD1, PLCE1, NFKBIA, JUN, PPP3CB, CARD10, PIK3CG, CD79B, ATF1, ITPR2, CHP1, CD79A, MALT1, NFATC4, CD180, CD40, VAV3, SYK, IRS1, PLCG2, SH2B2, LYN, PLCB3, PIK3CB, VAV1, PIK3AP1, PIK3CD, PLEKHA2, ELK1 |
| Fcγ Receptor-mediated Phagocytosis in Macrophages and Monocytes | 4.98E00 | RAC2, GAB2, FYN, PLD2, PTK2B, PIK3R1, TLN1, FCGR1A, PAK1, PIK3CG, PRKCE, VASP, FGR, ACTA1, RPS6KB1, SRC, ARPC5L, ACTB, FYB, PRKCG, PLD4, DOCK1, TLN2, WAS, VAV3, SYK, LYN, VAV1, ACTG1, LCP2 |
| IL-8 Signaling | 4.98E00 | MAP2K4, RELA, RAC2, ANGPT2, PLD2, PTK2B, MYL2, PIK3R1, GNB5, LIMK2, IQGAP1, NFKB1, PIK3R4, PDGFC, CCND1, GNG7, LIMK1, VEGFA, ROCK2, GNB4, GNG11, JUN, RHOT1, PIK3CG, PRKCE, FIGF, MYL12B, LASP1, VASP, ITGB5, TEK, EGFR, SRC, RPS6KB1, NOX4, RHOC, FLT4, VEGFC, MMP2, PRKCG, GNAI2, PLD4, ITGB2, ITGAM, RND3, PIK3CB, PIK3CD, MMP9, ITGAX |
| Gluconeogenesis I | 4.96E00 | PGK1, ENO3, PGAM2, ME1, MDH1, ENO1, GPI, ALDOB, ME2, ALDOA, GAPDH, MDH2, GAPDHS |
| Cholesterol Biosynthesis I | 4.95E00 | SQLE, FDFT1, NSDHL, DHCR7, HSD17B7, LSS, TM7SF2, SC5D, CYP51A1 |
| Cholesterol Biosynthesis II (via 24, 25-dihydrolanosterol) | 4.95E00 | SQLE, FDFT1, NSDHL, DHCR7, HSD17B7, LSS, TM7SF2, SC5D, CYP51A1 |
| Cholesterol Biosynthesis III (via Desmosterol) | 4.95E00 | SQLE, FDFT1, NSDHL, DHCR7, HSD17B7, LSS, TM7SF2, SC5D, CYP51A1 |
| iCOS-iCOSL Signaling in T Helper Cells | 4.85E00 | RELA, GAB2, HLA-DOA, Calm1 (includes others), PIK3R1, NFKBIE, HLA-DQA1, HLA-DQB1, PIK3R4, NFKB1, PTPRC, NFKBIA, PPP3CB, HLA-DMA, PIK3CG, HLA-DMB, IL2RB, IL2RG, ITPR2, CSK, CHP1, NFATC4, CD3G, CD80, CD40, FCER1G, HLA-DOB, PIK3CB, VAV1, PIK3CD, PLEKHA2, LCP2, HLA-DRB5 |
| PKCθ Signaling in T Lymphocytes | 4.8E00 | MAP2K4, RAC2, MAP3K15, FYN, RELA, HLA-DOA, PIK3R1, NFKBIE, HLA-DQA1, HLA-DQB1, NFKB1, PIK3R4, NFKBIA, JUN, PPP3CB, HLA-DMA, PIK3CG, HLA-DMB, CHP1, MALT1, NFATC4, MAP3K12, CD3G, CD80, CARD11, VAV3, PLCG2, FCER1G, HLA-DOB, CD86, PIK3CB, VAV1, PIK3CD, LCP2, HLA-DRB5 |
| Role of Macrophages, Fibroblasts and Endothelial Cells in Rheumatoid Arthritis | 4.72E00 | MAP2K4, TRAF3, FZD3, PIK3R1, TLR8, IL17RC, PIK3R4, CCND1, FCGR1A, IL18R1, VEGFA, ROCK2, PLCE1, TGFB1, PIK3CG, TRAF4, TLR1, FIGF, IL1RAP, FZD2, TNFSF13B, SFRP4, VEGFC, TLR9, TCF3, C5, IL33, IL18, PLCG2, GNAO1, PLCB3, PIK3CD, SFRP1, PDGFD, RELA, FN1, Calm1 (includes others), SFRP2, NFKBIE, FZD1, CCL5, NFKB1, IL17RA, PDGFC, PLCD1, TRADD, NFKBIA, JUN, PPP3CB, CCL2, NGFR, SMO, PRKCE, SFRP5, TLR3, TRAF1, SRC, TNFRSF1A, DAAM1, CHP1, NFATC4, IL1R1, PRKCG, IL16, FZD4, CSF1, APC2, PIK3CB, LRP1, WNT5A |
| Calcium Signaling | 4.7E00 | MYH10, MYH4, TNNT1, Calm1 (includes others), CAMK1, MYH9, CAMK1D, TNNI2, MYL2, MYH8, TNNT3, TNNC1, MYL6B, Tpm2, PRKAG1, ATP2A1, CABIN1, PPP3CB, CASQ1, HDAC7, RYR1, ACTA1, MYL3, MYH1, HDAC9, LETM1, TP63, TNNC2, ITPR2, TRDN, HDAC1, CHP1, SLC8A3, ATP2A3, MYH7, NFATC4, TRPC6, MYL1, MYH2, Tpm4, CHRNB2, MYH3, PRKACA, TNNI1, SLC8A1, ATP2B4, GRIA3 |
| Tec Kinase Signaling | 4.7E00 | MAP2K4, FYN, RELA, JAK1, PTK2B, PIK3R1, GNB5, NFKB1, PIK3R4, GNG7, GNB4, PAK1, GNG11, GNA15, TNFSF12, RHOT1, PIK3CG, PRKCE, ACTA1, FGR, ITGA4, ITGB1, TNFRSF21, SRC, STAT6, RHOC, ACTB, ITGA5, PRKCG, GNAI2, RND3, WAS, VAV3, PLCG2, GNAO1, FCER1G, LYN, VAV1, STAT2, PIK3CB, PIK3CD, JAK3, ACTG1 |
| RhoGDI Signaling | 4.7E00 | MYL2, GNB5, PIP4K2B, LIMK2, MYL6B, CDH11, GNG7, LIMK1, ROCK2, GNB4, PAK1, GNG11, GNA15, CFL2, RHOT1, ARHGEF2, ARHGEF3, MYL12B, ACTA1, MYL3, ITGA4, ITGB1, SRC, ARHGAP6, ARPC5L, RHOC, MYLPF, ACTB, ARHGAP4, ITGA5, ARHGEF17, MYL1, ARHGDIB, GNAI2, CDH2, CDH5, RND3, PIP5K1C, GNAO1, CD44, ARHGAP1, PIP4K2A, ARHGEF10, ACTG1, ESR1, MSN |
| Role of Tissue Factor in Cancer | 4.67E00 | FYN, PTK2B, PIK3R1, RPS6KA3, LIMK2, PIK3R4, F2, FGG, LIMK1, VEGFA, PAK1, ARRB1, GNA15, CFL2, PIK3CG, FGB, FGR, ITGB5, EGFR, TP53, ITGB1, RPS6KB1, SRC, CASP3, VEGFC, F3, F10, CSF1, LYN, PIK3CB, RPS6KA4, PIK3CD, FGA |
| Altered T Cell and B Cell Signaling in Rheumatoid Arthritis | 4.57E00 | RELA, HLA-DOA, TRAF3, HLA-DQA1, TLR8, HLA-DQB1, NFKB1, CXCL13, TGFB1, HLA-DMA, TLR1, HLA-DMB, TLR3, TNFSF13B, CD79B, RELB, CD79A, TLR9, IL33, IL18, CD80, CD40, PRTN3, CSF1, FCER1G, CD86, HLA-DOB, HLA-DRB5 |
| Isoleucine Degradation I | 4.57E00 | HSD17B10, ECHS1, ACAT2, BCAT2, DLD, HADHB, ACADSB, EHHADH, HADHA |
| Germ Cell-Sertoli Cell Junction Signaling | 4.55E00 | MAP2K4, RAC2, MAP3K15, PIK3R1, MYO7A, Actn3, MLLT4, LIMK2, PIK3R4, IQGAP1, BCAR1, LIMK1, PAK1, TUBA8, CFL2, TGFB1, RHOT1, PIK3CG, JUP, VCL, ACTA1, ITGB1, SRC, TJP1, TNFRSF1A, RHOC, ACTN2, ACTB, TUBB2A, TUBG1, TUBA4A, GSN, MAP3K12, CDH2, TUBA1A, RND3, TGFB3, ZYX, PIK3CB, PIK3CD, ACTG1, CTNND1, PVRL2 |
| Tryptophan Degradation III (Eukaryotic) | 4.32E00 | HSD17B10, KMO, ACAT2, TDO2, HAAO, HADHB, IDO1, EHHADH, CA1, HADHA, HADH |
| Integrin Signaling | 4.22E00 | MAP2K4, RAC2, MYL2, PIK3R1, MYLK2, TLN1, PIK3R4, PAK1, ITGA9, ITGA11, PIK3CG, CAV1, ACTA1, ITGB5, ITGA4, RALB, ITGA5, BCAR3, TTN, DOCK1, TLN2, ITGAM, RND3, PLCG2, ZYX, PIK3CD, ITGA1, ACTG1, FYN, Actn3, BCAR1, ITGB7, RHOT1, VCL, VASP, MYL12B, ITGB1, SRC, PARVA, ARPC5L, RHOC, ACTN2, ACTB, ITGAL, ITGB2, WIPF1, WAS, PIK3CB, NEDD9, ITGAX |
| Glycolysis I | 4.18E00 | PGK1, ENO1, GPI, ALDOB, ENO3, PKM, PGAM2, ALDOA, GAPDH, PFKL, GAPDHS, PFKM |
| Reelin Signaling in Neurons | 4.1E00 | MAP2K4, ITGB1, SRC, FYN, APOE, CNR1, PIK3R1, ITGA5, PIK3R4, ITGAL, MAPK8IP1, APP, ITGB2, APBB1, PIK3CG, LYN, PIK3CD, PIK3CB, ITGA1, MAP4K1, ARHGEF2, ARHGEF3, ARHGEF10, FGR, ITGA4 |
| Nur77 Signaling in T Lymphocytes | 4.1E00 | HDAC9, HLA-DOA, Calm1 (includes others), CASP3, CHP1, HDAC1, HLA-DQA1, HLA-DQB1, CABIN1, CD3G, PPP3CB, CD80, HLA-DMA, HLA-DMB, FCER1G, HLA-DOB, CD86, CYCS, MAPK7, HLA-DRB5 |
| Role of Osteoblasts, Osteoclasts and Chondrocytes in Rheumatoid Arthritis | 4.09E00 | MAP2K4, FZD3, PIK3R1, PIK3R4, IL18R1, TGFB1, PIK3CG, IL1RAP, ADAMTS5, FZD2, SFRP4, ITGA5, GSN, TCF3, IL33, IL18, PIK3CD, SFRP1, RELA, ADAM17, Calm1 (includes others), SFRP2, PTK2B, NFKBIE, FZD1, NFKB1, TRADD, NFKBIA, JUN, IGF1, PPP3CB, NGFR, SMO, SFRP5, BMP1, ITGB1, SRC, CTSK, TNFRSF1A, CHP1, SMAD6, NFATC4, IL1R1, TNFRSF11A, CSF1R, COL1A1, FZD4, CSF1, APC2, PIK3CB, BMP6, LRP1, WNT5A |
| Tight Junction Signaling | 4.08E00 | MYH10, MYH4, RELA, MYH9, MYL2, MYH8, MARK2, PPP2R5B, MLLT4, MYL6B, NFKB1, PRKAG1, MPDZ, JUN, TGFB1, NGFR, PVRL1, ARHGEF2, VCL, NAPA, VASP, MYL3, ACTA1, MYH1, TIAM1, TJP1, TNFRSF1A, RAB13, ACTB, MYH7, MYL1, EPB41, F11R, CLDN5, MYH2, JAM3, PPP2R3A, MYH3, PRKACA, TGFB3, ACTG1, CLDN22, PVRL2 |
| Phospholipase C Signaling | 3.88E00 | PLD2, MYL2, GNB5, MYL6B, GNB4, PLCE1, HDAC7, MYL3, ITGA4, PLA2G12A, ITPR2, RALB, ITGA5, ARHGEF17, CD79A, RAPGEF3, CD3G, RND3, PLA2G2D, SYK, PLCG2, PLCB3, ADCY10, ARHGEF10, LCP2, FYN, RELA, Calm1 (includes others), RPS6KA3, NFKB1, GNG7, GNG11, PPP3CB, AHNAK, RHOT1, PRKCE, ARHGEF2, ARHGEF3, MYL12B, ITGB1, HDAC9, SRC, CD79B, RHOC, MYLPF, CHP1, HDAC1, NFATC4, MYL1, PRKCG, PLD4, PLA2G4A, PLA2G2E, LYN, FCER1G, ADCY7 |
| B Cell Development | 3.88E00 | HLA-DOA, SPN, CD79B, HLA-DQA1, CD79A, HLA-DQB1, PTPRC, CD80, CD40, HLA-DMA, HLA-DMB, HLA-DOB, CD86, HLA-DRB5 |
| Role of NFAT in Regulation of the Immune Response | 3.83E00 | FYN, RELA, HLA-DOA, Calm1 (includes others), PIK3R1, NFKBIE, HLA-DQA1, GNB5, HLA-DQB1, NFKB1, PIK3R4, FCGR1A, GNG7, CABIN1, GNB4, NFKBIA, JUN, GNG11, GNA15, PPP3CB, HLA-DMA, PIK3CG, HLA-DMB, CD79B, ITPR2, CHP1, CD79A, NFATC4, GNAI2, CD3G, CD80, SYK, PLCG2, GNAO1, FCER1G, LYN, HLA-DOB, CD86, PLCB3, PIK3CB, PIK3CD, HLA-DRB5, LCP2 |
| Colorectal Cancer Metastasis Signaling | 3.76E00 | MAP2K4, JAK1, FZD3, PIK3R1, SMAD3, GNB5, TLR8, PIK3R4, CCND1, VEGFA, GNB4, TGFB1, PIK3CG, TLR1, FIGF, FZD2, TP53, CASP3, VEGFC, MMP2, TLR9, TCF3, MMP23B, RND3, PRKACA, TGFB3, PIK3CD, ADCY10, RELA, PTGER3, MMP15, FZD1, NFKB1, PDGFC, GNG7, PRKAG1, JUN, GNG11, ARRB1, RHOT1, SMO, TLR3, MMP12, PTGER4, EGFR, SRC, RHOC, TNFRSF1A, FZD4, PIK3CB, JAK3, ADCY7, LRP1, MMP9, WNT5A |
| Ethanol Degradation IV | 3.75E00 | ALDH4A1, ACSL3, ACSS3, ALDH1A3, ACSS2, CYGB, GPX7, ALDH9A1, ACSL1, ALDH7A1 |
| HIF1α Signaling | 3.73E00 | PIK3R1, MMP15, HIF1A, PIK3R4, NAA10, PDGFC, VEGFA, JUN, PIK3CG, EGLN3, FIGF, MMP12, APEX1, TP53, SLC2A5, EGLN2, COPS5, MAPK6, VEGFC, MMP2, SLC2A4, MMP23B, CUL2, PIK3CB, PIK3CD, MAPK7, LDHA, VHL, MMP9 |
| Sertoli Cell-Sertoli Cell Junction Signaling | 3.72E00 | MAP2K4, SPTBN1, MAP3K15, MYO7A, TGFBR3, Actn3, MLLT4, BCAR1, PRKAG1, MPP6, JUN, TUBA8, SPTB, PVRL1, JUP, VCL, GUCY1B3, ACTA1, ITGA4, ITGB1, SRC, TNFRSF1A, TJP1, ACTN2, ACTB, TUBB2A, TUBG1, TUBA4A, ITGA5, MAP3K12, F11R, EPB41, CLDN5, TUBA1A, JAM3, WAS, GUCY1A2, PRKACA, TGFB3, ADCY10, ELK1, ACTG1, CLDN22, PVRL2 |
| Dendritic Cell Maturation | 3.66E00 | MAP2K4, RELA, HLA-DOA, LEP, PIK3R1, NFKBIE, HLA-DQA1, CD83, HLA-DQB1, NFKB1, PIK3R4, FCGR1A, COL1A2, PLCD1, NFKBIA, PLCE1, HLA-DMA, NGFR, PIK3CG, HLA-DMB, TLR3, COL18A1, TNFRSF1A, RELB, TLR9, IL33, COL1A1, COL5A3, IL18, CD80, CD40, PLCG2, FSCN1, FCER1G, HLA-DOB, CD86, PLCB3, STAT2, PIK3CB, PIK3CD, IRF8, HLA-DRB5, IFNAR1, COL3A1 |
| Superpathway of Geranylgeranyldiphosphate Biosynthesis I (via Mevalonate) | 3.66E00 | MVD, FDPS, PMVK, ACAT2, IDI1, COX10, HADHB, HMGCS2, HADHA |
| PAK Signaling | 3.61E00 | MAP2K4, PTK2B, MYL2, PIK3R1, PAK1IP1, MYL6B, LIMK2, PIK3R4, PDGFC, LIMK1, PAK1, CFL2, PIK3CG, MYL3, MYL12B, ITGA4, PDGFRB, ITGB1, CASP3, MYLPF, ITGA5, MYL1, EPHB3, PIK3CB, PIK3CD, PDGFD |
| NRF2-mediated Oxidative Stress Response | 3.61E00 | MAP2K4, PIK3R1, NQO2, MAF, DNAJC15, PIK3R4, CLPP, SOD3, CUL3, JUN, SOD2, PIK3CG, UBE2K, DNAJA3, PRKCE, DNAJA2, FMO1, GCLM, TXN, FKBP5, CBR1, ACTA1, GSTA3, DNAJB8, MGST1, ACTB, SOD1, DNAJB9, GSTO1, PRKCG, GSR, DNAJC21, DNAJC5, MGST2, DNAJC18, PIK3CB, PIK3CD, CDC34, DNAJB6, MAPK7, ENC1, ACTG1, DNAJC7, EPHX1 |
| Glioma Invasiveness Signaling | 3.59E00 | TIMP3, F2R, RHOC, VTN, PIK3R1, MMP2, PIK3R4, PLG, TIMP4, RND3, RHOT1, PIK3CG, CD44, PIK3CB, PIK3CD, PLAU, MMP9, ITGB5, TIMP2 |
| Rac Signaling | 3.57E00 | MAP2K4, RELA, PTK2B, PIK3R1, PIP4K2B, LIMK2, PIK3R4, NFKB1, IQGAP1, LIMK1, PAK1, JUN, CFL2, PIK3CG, ITGA4, IQGAP3, ITGB1, RPS6KB1, NOX4, TIAM1, ARPC5L, ITGA5, PIP5K1C, CD44, PIK3CB, PIK3CD, PARD3, ELK1, PIP4K2A |
| Cellular Effects of Sildenafil (Viagra) | 3.56E00 | MYH10, MYH4, CACNA1S, Calm1 (includes others), MYH9, MYL2, MYH8, MYL6B, PRKAG1, PLCD1, CACNA1E, PLCE1, CACNG7, MYL12B, MYL3, ACTA1, GUCY1B3, MYH1, ITPR2, ACTB, MYLPF, MYH7, PDE4B, MYL1, CACNA1A, MYH2, PLCG2, GUCY1A2, MYH3, PRKACA, PLCB3, ADCY10, ADCY7, ACTG1 |
| phagosome formation | 3.41E00 | MRC1, FN1, VTN, PIK3R1, TLR8, PIK3R4, FCGR1A, PLCD1, PLCE1, RHOT1, PIK3CG, TLR1, PRKCE, TLR3, ITGA4, ITGB1, RHOC, MRC2, ITGA5, TLR9, PRKCG, Fcrls, SCARA3, RND3, SYK, PLCG2, FCER1G, PLCB3, PIK3CB, PIK3CD |
| CXCR4 Signaling | 3.4E00 | MAP2K4, MYL2, PIK3R1, GNB5, MYL6B, PIK3R4, BCAR1, GNG7, ROCK2, ELMO3, GNB4, PAK1, JUN, GNG11, GNA15, RHOT1, PIK3CG, PRKCE, MYL12B, MYL3, SRC, RHOC, ITPR2, MYLPF, MYL1, PRKCG, GNAI2, DOCK1, RND3, GNAO1, LYN, PLCB3, PIK3CB, PIK3CD, ADCY10, ELK1, ELMO1, ADCY7 |
| Gαq Signaling | 3.39E00 | RELA, PLD2, RGS18, Calm1 (includes others), PTK2B, PIK3R1, NFKBIE, GNB5, NFKB1, PIK3R4, AVPR1A, GNG7, ROCK2, GNB4, NFKBIA, GNG11, GNA15, PPP3CB, RHOT1, PIK3CG, PRKCE, ARHGEF25, RGS2, ITPR2, RHOC, CSK, RGS7, CHP1, NFATC4, PRKCG, PLD4, RND3, PLCG2, PLCB3, PIK3CB, PIK3CD, ELK1 |
| Ephrin B Signaling | 3.35E00 | RAC2, EPHB4, RGS3, ITSN1, EPHB2, GNB5, GNG7, LIMK1, GNAI2, ROCK2, GNB4, EFNB2, PAK1, GNG11, CFL2, GNA15, VAV3, EFNB1, GNAO1, CAP1, EPHB3, VAV1 |
| RAR Activation | 3.35E00 | MAP2K4, RELA, ARID1A, PIK3R1, SMAD3, Rdh7, NFKB1, PRKAG1, VEGFA, JUN, ALDH1A3, TGFB1, RARA, PIK3CG, PRKCE, GTF2H5, CSNK2B, NT5C1B, SRC, Aldh1a7, SRA1, DHRS3, RDH11, NRIP2, CSK, ACTB, RELB, SMAD7, SMAD6, PRKCG, CSF2RB, CSNK2A2, RBP7, SMARCA2, IGFBP3, PRKACA, TGFB3, PIK3CB, PIK3CD, NCOR2, PML, ADCY10, ADCY7, HLTF, PPARGC1A |
| MSP-RON Signaling Pathway | 3.34E00 | PIK3R1, ACTB, CCR2, PIK3R4, CSF2RB, ITGB2, KLKB1, ITGAM, CCL2, CSF1, PIK3CG, PIK3CB, PIK3CD, MST1R, ACTG1, ACTA1 |
| Thrombin Signaling | 3.3E00 | RELA, CAMK1, F2R, CAMK1D, MYL2, PIK3R1, GNB5, MYL6B, NFKB1, PIK3R4, GNG7, F2, PLCD1, ROCK2, GNB4, GNG11, PLCE1, GNA15, RHOT1, PIK3CG, GATA6, PRKCE, ARHGEF2, ARHGEF3, MYL3, MYL12B, EGFR, SRC, RPS6KB1, ITPR2, RHOC, MYLPF, MYL1, PRKCG, GNAI2, RND3, PLCG2, GNAO1, PLCB3, PIK3CB, PIK3CD, ADCY10, ELK1, ARHGEF10, ADCY7 |
| Glutaryl-CoA Degradation | 3.28E00 | HSD17B10, ACAT2, HADHB, EHHADH, CA1, HADHA, HADH |
| Regulation of IL-2 Expression in Activated and Anergic T Lymphocytes | 3.24E00 | MAP2K4, RELA, FYN, Calm1 (includes others), NFKBIE, SMAD3, CHP1, TOB1, NFATC4, MALT1, NFKB1, CD3G, JUN, NFKBIA, CD80, PPP3CB, TGFB1, CARD11, VAV3, PLCG2, TGFB3, VAV1, ELK1 |
| Granulocyte Adhesion and Diapedesis | 3.18E00 | MMP15, CCL22, SDC3, CCL5, Cxcl9, CXCL10, ICAM2, CCL2, CXCL13, NGFR, CCL25, MMP12, IL1RAP, ITGA4, ITGB1, TNFRSF1A, PF4, THY1, ITGA5, MMP2, IL1R1, CCL11, ITGAL, SELPLG, GLG1, C5, GNAI2, IL33, ITGB2, CXCL16, IL18, CLDN5, ITGAM, MMP23B, SELP, CDH5, JAM3, PECAM1, ITGA1, MMP9, CLDN22, MSN |
| Inhibition of Matrix Metalloproteases | 3.15E00 | HSPG2, TIMP3, ADAM17, RECK, MMP15, MMP2, TIMP4, MMP23B, ADAM12, THBS2, MMP12, LRP1, MMP9, TIMP2 |
| NF-κB Signaling | 3.11E00 | RELA, TRAF3, PIK3R1, TGFBR3, NFKBIE, TLR8, TNFAIP3, NFKB1, PIK3R4, TANK, TRADD, NFKBIA, CARD10, PIK3CG, NGFR, TLR1, TLR3, CSNK2B, TNFSF13B, PDGFRB, EGFR, TNFRSF1A, RELB, FGFR1, FLT4, HDAC1, IL1R1, MALT1, TNFRSF11A, TLR9, IL33, CSNK2A2, IL18, NTRK2, CD40, CARD11, PLCG2, FCER1G, PRKACA, PIK3CB, PIK3CD |
| Fatty Acid β-oxidation I | 3.11E00 | HSD17B10, ACSL3, ECHS1, ECI2, HADHB, SLC27A1, EHHADH, ACADM, ECI1, ACSL1, HADHA, HADH |
| T Helper Cell Differentiation | 3.11E00 | STAT6, HLA-DOA, IL2RG, TNFRSF1A, HLA-DQA1, HLA-DQB1, RORC, BCL6, IL18R1, IL18, CD40, CD80, TGFB1, HLA-DMA, NGFR, HLA-DMB, IL10RA, FCER1G, HLA-DOB, CD86, HLA-DRB5 |
| Oxidative Ethanol Degradation III | 3.09E00 | ALDH4A1, ACSL3, ACSS3, ALDH1A3, ACSS2, ALDH9A1, ACSL1, ALDH7A1 |
| Cardiac Hypertrophy Signaling | 3.07E00 | MAP2K4, MAP3K15, CACNA1S, MYL2, PIK3R1, GNB5, MYL6B, PIK3R4, ADRB3, ROCK2, GNB4, CACNA1E, PLCE1, GNA15, TGFB1, PIK3CG, MYL3, RPS6KB1, CACNA1A, MAP3K12, RND3, PLCG2, IRS1, GNAO1, PRKACA, TGFB3, PLCB3, PIK3CD, ADCY10, Calm1 (includes others), GNG7, PRKAG1, PLCD1, JUN, GNG11, PPP3CB, IGF1, RHOT1, MYL12B, ADRB2, RHOC, MYLPF, CHP1, NFATC4, MYL1, GNAI2, EIF2B1, PIK3CB, ELK1, ADCY7 |
| Pancreatic Adenocarcinoma Signaling | 3.06E00 | MAP2K4, RELA, PLD2, JAK1, SMAD3, PIK3R1, PIK3R4, NFKB1, CCND1, PDGFC, VEGFA, CYP2E1, TGFB1, PIK3CG, FIGF, EGFR, TP53, TFDP1, VEGFC, PLD4, CCNE1, E2F1, TGFB3, PIK3CB, PIK3CD, JAK3, ELK1, MMP9 |
| Regulation of Actin-based Motility by Rho | 3.06E00 | ITGB1, RAC2, PFN1, MYL2, RHOC, ARPC5L, ACTB, MYLPF, ITGA5, PIP4K2B, MYL6B, GSN, MYL1, LIMK1, WIPF1, PAK1, RND3, WAS, PIP5K1C, RHOT1, PIP4K2A, MYL3, ACTA1, MYL12B, ITGA4 |
| RhoA Signaling | 3.01E00 | PFN1, MYL2, PTK2B, MYLK2, PIP4K2B, LIMK2, MYL6B, LIMK1, ROCK2, CFL2, IGF1, CDC42EP1, MYL12B, MYL3, ACTA1, PLXNA1, ARHGAP6, NRP2, ARPC5L, ACTB, MYLPF, ARHGAP4, TTN, PKN1, MYL1, RND3, PIP5K1C, ARHGAP1, PIP4K2A, ACTG1, MSN |
| Gα12/13 Signaling | 3E00 | MAP2K4, RELA, PTK2B, MYL2, F2R, NFKBIE, PIK3R1, TBXA2R, MYL6B, PIK3R4, NFKB1, CDH11, F2, ROCK2, NFKBIA, JUN, PIK3CG, MYL12B, MYL3, SRC, MYLPF, MYL1, CDH2, CDH5, VAV3, PIK3CB, VAV1, PIK3CD, MAPK7, ELK1 |
| Ubiquinol-10 Biosynthesis (Eukaryotic) | 3E00 | CYP7B1, MICAL1, MICAL2, CYP2E1, BCKDHA, COQ3, ECHDC1 |
| Mevalonate Pathway I | 3E00 | MVD, PMVK, ACAT2, IDI1, HADHB, HMGCS2, HADHA |
| FAK Signaling | 2.98E00 | ITGB1, SRC, FYN, ACTB, CSK, PIK3R1, ITGA5, TLN1, PIK3R4, BCAR1, GIT2, DOCK1, PAK1, TLN2, WAS, PIK3CG, PLCG2, PIK3CB, PIK3CD, VCL, ACTG1, ACTA1, EGFR, ITGA4 |
| VEGF Signaling | 2.98E00 | SRC, PTPN6, PTK2B, ACTB, ACTN2, PIK3R1, FLT4, Actn3, VEGFC, HIF1A, PIK3R4, PDGFC, EIF2S2, VEGFA, ROCK2, PLCG2, PIK3CG, EIF2B1, FIGF, PIK3CD, PIK3CB, VCL, ACTG1, EIF1AY, ACTA1 |
| Estrogen-Dependent Breast Cancer Signaling | 2.98E00 | HSD17B10, RELA, SRC, HSD17B13, PIK3R1, HSD17B7, PIK3R4, NFKB1, CCND1, JUN, IGF1, PIK3CG, HSD17B12, PIK3CB, PIK3CD, AKR1C4, ELK1, ESR1, EGFR |
| B Cell Receptor Signaling | 2.96E00 | MAP2K4, RAC2, MAP3K15, GAB2, RELA, Calm1 (includes others), PTK2B, POU2F2, PIK3R1, NFKBIE, NFKB1, PIK3R4, BCL6, PTPRC, NFKBIA, JUN, PPP3CB, CFL2, CARD10, PIK3CG, CD22, RASSF5, RPS6KB1, PTPN6, CD79B, CSK, CD79A, NFATC4, MALT1, TCF3, MAP3K12, EBF1, VAV3, SYK, PLCG2, LYN, VAV1, PIK3AP1, PIK3CB, PIK3CD, ELK1 |
| Macropinocytosis Signaling | 2.95E00 | MRC1, ITGB1, SRC, PIK3R1, ITGA5, PIK3R4, PDGFC, CSF1R, ITGB7, PRKCG, ITGB2, PAK1, CSF1, PIK3CG, PLCG2, PRKCE, PIK3CB, PIK3CD, PDGFD, ITGB5 |
| Calcium-induced T Lymphocyte Apoptosis | 2.89E00 | HLA-DOA, Calm1 (includes others), ITPR2, CHP1, HDAC1, HLA-DQA1, ATP2A3, HLA-DQB1, CABIN1, PRKCG, ATP2A1, CD3G, PPP3CB, HLA-DMA, HLA-DMB, FCER1G, HLA-DOB, PRKCE, HLA-DRB5 |
| Agrin Interactions at Neuromuscular Junction | 2.87E00 | MAP2K4, ITGB1, RAC2, SRC, ACTB, LAMA2, ITGA5, NRG4, ITGAL, LAMC1, ITGB2, PAK1, JUN, UTRN, ITGA1, AGRN, ACTG1, ACTA1, EGFR, ITGA4 |
| Growth Hormone Signaling | 2.87E00 | RPS6KB1, PTPN6, PIK3R1, SOCS6, RPS6KA3, PIK3R4, SLC2A4, PRKCG, IGF1, IRS1, PLCG2, PIK3CG, IGFBP3, PRKCE, SOCS2, RPS6KA4, PIK3CB, PIK3CD, ELK1, SOCS5 |
| Cdc42 Signaling | 2.86E00 | MAP2K4, FGD3, HLA-DOA, MYL2, HLA-DQA1, LIMK2, MYL6B, HLA-DQB1, IQGAP1, LIMK1, DIAPH1, PAK1, JUN, CFL2, HLA-DMA, HLA-DMB, MYL12B, MYL3, IQGAP3, ITGA4, ITGB1, SRC, ARPC5L, MYLPF, ITGA5, FGD1, MYL1, CD3G, WIPF1, CDC42BPA, EXOC4, APC2, WAS, FCER1G, HLA-DOB, VAV1, CDC42SE1, PARD3, HLA-DRB5 |
| Superpathway of Inositol Phosphate Compounds | 2.83E00 | DUSP8, PTPN23, PPP1R1A, PIK3R1, NUDT12, PPFIA3, PPP2R5B, PIP4K2B, IGBP1, STYX, PIK3R4, UBLCP1, SACM1L, PTPRC, PLCD1, PLCE1, ITPKB, PTPRJ, DUSP10, PIK3CG, PPP1CA, NUDT4, PMPCA, CDC25A, PTPN7, PTPN6, TMEM55B, DUSP27, PPTC7, ITPKA, PLD4, ITPK1, TNS3, PI4K2A, PIP5K1C, PPP2R3A, PLCG2, CILP, PLCB3, PIK3CB, PIK3CD, PIP4K2A, SIRPA, DUSP16 |
| Nitric Oxide Signaling in the Cardiovascular System | 2.77E00 | KNG1, CACNA1S, Calm1 (includes others), PIK3R1, PIK3R4, PDGFC, PRKAG1, ADRB3, ATP2A1, VEGFA, CACNA1E, PIK3CG, CAV1, PRKCE, FIGF, GUCY1B3, ITPR2, FLT4, VEGFC, ATP2A3, CACNA1A, PRKCG, GUCY1A2, PRKACA, PIK3CB, PIK3CD |
| Wnt/β-catenin Signaling | 2.76E00 | SFRP2, FZD3, TGFBR3, MARK2, PPP2R5B, Ubb, BCL9, FZD1, CCND1, JUN, TGFB1, RARA, SMO, SFRP5, MAP4K1, CSNK2B, SOX18, FZD2, SOX5, TP53, SOX7, SFRP4, SRC, GJA1, HDAC1, TCF3, CSNK2A2, CDH2, FZD4, CDH5, APC2, PPP2R3A, GNAO1, CD44, TLE3, TGFB3, SFRP1, LRP1, WNT5A |
| Production of Nitric Oxide and Reactive Oxygen Species in Macrophages | 2.76E00 | MAP2K4, APOE, MAP3K15, RELA, APOA4, JAK1, APOB, PIK3R1, APOA2, NFKBIE, PPP2R5B, PPP1R3A, PIK3R4, NFKB1, PON1, NFKBIA, JUN, RHOT1, CYBA, NGFR, PIK3CG, PRKCE, SERPINA1, PPP1CA, PTPN6, TNFRSF1A, RHOC, NCF4, PRKCG, MAP3K12, PPP1R3D, ALB, APOA1, RND3, PPP2R3A, PLCG2, PIK3CB, PIK3CD, IRF8, JAK3, SIRPA |
| Acetate Conversion to Acetyl-CoA | 2.72E00 | ACSL3, ACSS3, ACSS2, ACSL1 |
| FXR/RXR Activation | 2.71E00 | MAP2K4, KNG1, APOE, SLC10A1, APOA4, APOB, SLC4A2, APOH, APOA2, VTN, AMBP, C4A/C4B, PON1, LCAT, RARA, FASN, SERPINA1, MLXIPL, HPX, TTR, CYP27A1, AHSG, SERPINF1, IL33, IL18, ALB, APOA1, SREBF1, PLTP, FGA, PPARGC1A |
| Role of Pattern Recognition Receptors in Recognition of Bacteria and Viruses | 2.71E00 | MAP2K4, RELA, LIF, PIK3R1, TLR8, C1QA, CCL5, C1QB, NFKB1, PIK3R4, RNASEL, TICAM1, TGFB1, PIK3CG, TLR1, NOD1, PRKCE, TLR3, NLRC4, OAS1, TLR9, C5, PRKCG, IL18, NOD2, PLCG2, SYK, DDX58, TGFB3, PIK3CB, PIK3CD |
| IL-9 Signaling | 2.7E00 | RELA, IL2RG, JAK1, IRS1, PIK3CG, PIK3R1, SOCS2, PIK3CB, PIK3CD, NFKB1, PIK3R4, JAK3 |
| Inhibition of Angiogenesis by TSP1 | 2.7E00 | MAP2K4, VEGFA, HSPG2, TP53, FYN, JUN, CASP3, TGFB1, THBS1, GUCY1A2, MMP9, GUCY1B3 |
| IL-4 Signaling | 2.69E00 | STAT6, RPS6KB1, HLA-DOA, PTPN6, IL2RG, JAK1, IRF4, PIK3R1, HLA-DQA1, NFATC4, HLA-DQB1, PIK3R4, HLA-DMA, PIK3CG, IRS1, HLA-DMB, HLA-DOB, PIK3CB, PIK3CD, JAK3, HLA-DRB5 |
| Type II Diabetes Mellitus Signaling | 2.68E00 | MAP2K4, RELA, PIK3R1, NFKBIE, PKM, SOCS6, PIK3R4, NFKB1, PRKAG1, KCNJ11, TRADD, NFKBIA, PIK3CG, NGFR, PRKAA2, SOCS2, PRKCE, ACSL3, TNFRSF1A, SLC2A4, PRKCG, IRS1, SH2B2, SLC27A1, PIK3CB, PIK3CD, ADIPOR2, SOCS5, ACSL1 |
| Molecular Mechanisms of Cancer | 2.67E00 | MAP2K4, GAB2, RAC2, JAK1, FZD3, PIK3R1, SMAD3, PIK3R4, CCND1, PAK1, BBC3, GNA15, TGFB1, SUFU, PIK3CG, FZD2, PTCH2, CDC25A, ITGA4, TP53, CASP3, TFDP1, RALB, ITGA5, RAPGEF3, ARHGEF17, TCF3, DAXX, RASGRF2, RND3, IRS1, E2F1, GNAO1, TGFB3, PRKACA, PLCB3, PIK3CD, ADCY10, ARHGEF10, RELA, FYN, NFKBIE, HIF1A, FZD1, NFKB1, PRKAG1, NFKBIA, JUN, RHOT1, SMO, PRKCE, ARHGEF2, ARHGEF3, BMP1, ITGB1, SRC, RHOC, SMAD7, SMAD6, PRKCG, GNAI2, CCNE1, FZD4, PIK3CB, RBPJ, CYCS, BMP6, ELK1, JAK3, ADCY7, LRP1, CTNND1, WNT5A |
| T Cell Receptor Signaling | 2.63E00 | MAP2K4, FYN, RELA, PTPN7, Calm1 (includes others), CSK, PIK3R1, NFATC4, MALT1, CD8A, NFKB1, PIK3R4, PTPRC, CD3G, JUN, NFKBIA, PPP3CB, CARD11, VAV3, PIK3CG, PIK3CD, PIK3CB, VAV1, ELK1, LCP2 |
| Ephrin A Signaling | 2.63E00 | FYN, PIK3R1, PIK3R4, BCAR1, LIMK1, ROCK2, PAK1, CFL2, EFNA5, NGFR, PIK3CG, VAV3, PIK3CB, VAV1, PIK3CD |
| Superoxide Radicals Degradation | 2.62E00 | SOD2, CYGB, SOD1, GPX7, SOD3 |
| Semaphorin Signaling in Neurons | 2.6E00 | DPYSL2, ITGB1, FYN, PLXNA1, FES, RHOC, DPYSL3, LIMK2, LIMK1, ROCK2, PAK1, RND3, CFL2, RHOT1, ARHGAP1, NRP1 |
| Remodeling of Epithelial Adherens Junctions | 2.55E00 | SRC, NME1, ARPC5L, ACTB, ACTN2, TUBG1, TUBB2A, TUBA4A, Actn3, DNM3, IQGAP1, DNM1, TUBA1A, TUBA8, ZYX, VCL, ACTG1, ACTA1, CTNND1 |
| NF-κB Activation by Viruses | 2.54E00 | ITGB1, RELA, CCR5, NFKBIE, PIK3R1, ITGA5, PIK3R4, NFKB1, ITGAL, TNFRSF14, PRKCG, ITGB2, NFKBIA, PIK3CG, PRKCE, PIK3CB, ITGA1, PIK3CD, ITGB5, ITGA4 |
| Fatty Acid β-oxidation III (Unsaturated, Odd Number) | 2.52E00 | ECI2, EHHADH, ECI1 |
| Breast Cancer Regulation by Stathmin1 | 2.51E00 | Calm1 (includes others), CAMK1, CAMK1D, PIK3R1, PPP2R5B, GNB5, PPP1R3A, LIMK2, PIK3R4, PRKAG1, GNG7, LIMK1, ROCK2, GNB4, PAK1, GNG11, TUBA8, PIK3CG, PRKCE, ARHGEF2, ARHGEF3, PPP1CA, TP53, ITPR2, TUBB2A, TUBG1, TUBA4A, ARHGEF17, PRKCG, GNAI2, PPP1R3D, CCNE1, TUBA1A, PPP2R3A, E2F1, PRKACA, PLCB3, PIK3CB, PIK3CD, ADCY10, ADCY7, ARHGEF10 |
| Antioxidant Action of Vitamin C | 2.5E00 | SLC2A5, PNPLA8, MAP2K4, RELA, PLD2, ABHD3, NFKBIE, NFKB1, SLC2A4, GLRX, GSTO1, PLD4, PLCD1, CSF2RB, PLA2G4A, PLA2G2E, PLCE1, NFKBIA, PLA2G2D, LCAT, CSF2RA, PLCG2, PLCB3, TXN, PLA2G12A |
| Glutathione Redox Reactions I | 2.5E00 | GSR, MGST1, MGST2, GPX1, GPX5, GPX4, GPX7, Gstt1 |
| Acute Myeloid Leukemia Signaling | 2.47E00 | MAP2K4, RUNX1, RELA, RPS6KB1, PIK3R1, FLT3, PIK3R4, NFKB1, TCF3, CCND1, CSF1R, CSF2RB, PIM1, CSF2RA, FLT3LG, PIK3CG, RARA, PIK3CB, PIK3CD, JUP, PML |
| Type I Diabetes Mellitus Signaling | 2.47E00 | MAP2K4, RELA, HLA-DOA, JAK1, NFKBIE, SOCS6, HLA-DQA1, HLA-DQB1, NFKB1, TRADD, NFKBIA, HLA-DMA, NGFR, HLA-DMB, SOCS2, IL1RAP, CASP3, TNFRSF1A, IL1R1, CD3G, CD80, FCER1G, CD86, HLA-DOB, CYCS, SOCS5, HLA-DRB5 |
| Protein Ubiquitination Pathway | 2.45E00 | UBE2A, USP20, Ubb, DNAJC15, SKP1, UBE2D4, BAG1, UCHL5, PSMA2, PSMA6, PSMB5, HSPA9, PSMD5, PSMC4, HSPB9, PSMD6, PSMD3, UCHL3, PSMD11, PSMB7, DNAJC5, PSMD12, DNAJC18, DNAJB6, PSMD4, VHL, PSMB3, ANAPC2, PSMD7, USP35, USP2, HSPA1L, UBE2F, USP3, HSPA12B, USP13, PSMC6, HSPE1, PSMA3, PSMD14, HSPB6, HSPA4L, DNAJB8, USP15, DNAJB9, USP33, HSPA12A, DNAJC21, PSMC1, CUL2, CDC34, UBE2D3, DNAJC7 |
| ERK/MAPK Signaling | 2.44E00 | RAC2, FYN, PTK2B, PIK3R1, PPP2R5B, PPP1R3A, TLN1, PIK3R4, BCAR1, PRKAG1, ELF4, PAK1, ETS2, PIK3CG, PRKCE, PPP1CA, PLA2G12A, ITGA4, ITGB1, SRC, ATF1, HIST1H3C, ITGA5, RAPGEF3, PRKCG, PLA2G4A, DOCK1, PPP1R3D, TLN2, DUSP9, PLA2G2E, PLA2G2D, PPP2R3A, PLCG2, PRKACA, PIK3CB, RPS6KA4, PIK3CD, ELK1, ELK3, ESR1 |
| CD40 Signaling | 2.4E00 | MAP2K4, RELA, TRAF3, ATF1, NFKBIE, PIK3R1, TNFAIP3, NFKB1, PIK3R4, TANK, NFKBIA, JUN, CD40, PIK3CG, PIK3CB, PIK3CD, JAK3, TRAF1 |
| Small Cell Lung Cancer Signaling | 2.32E00 | TP53, RELA, TRAF3, TFDP1, NFKBIE, PIK3R1, NFKB1, PIK3R4, CCND1, PIAS3, CCNE1, NFKBIA, PIK3CG, TRAF4, E2F1, PIK3CB, PIK3CD, CYCS, TRAF1 |
| Role of NFAT in Cardiac Hypertrophy | 2.3E00 | MAP2K4, Calm1 (includes others), LIF, CAMK1, CAMK1D, PIK3R1, GNB5, PIK3R4, GNG7, PRKAG1, CABIN1, PLCD1, GNB4, GNG11, PLCE1, PPP3CB, IGF1, TGFB1, PIK3CG, HDAC7, PRKCE, HDAC9, SRC, ITPR2, HDAC1, CHP1, SLC8A3, NFATC4, PRKCG, GNAI2, PLCG2, PRKACA, TGFB3, PLCB3, PIK3CB, PIK3CD, ADCY10, SLC8A1, ADCY7 |
| Ethanol Degradation II | 2.3E00 | ADH5, HSD17B10, ALDH4A1, ACSL3, ACSS3, ALDH1A3, ACSS2, PECR, ALDH9A1, ACSL1, ALDH7A1 |
| Zymosterol Biosynthesis | 2.29E00 | NSDHL, HSD17B7, TM7SF2, CYP51A1 |
| fMLP Signaling in Neutrophils | 2.28E00 | RELA, Calm1 (includes others), NFKBIE, PIK3R1, GNB5, PIK3R4, NFKB1, GNG7, GNB4, NFKBIA, GNG11, PPP3CB, PIK3CG, PRKCE, NOX4, ARPC5L, ITPR2, CHP1, NFATC4, PRKCG, GNAI2, WAS, PLCB3, PIK3CB, PIK3CD, ELK1 |
| D-myo-inositol-5-phosphate Metabolism | 2.28E00 | DUSP8, PTPN23, PPP1R1A, NUDT12, PPFIA3, PPP2R5B, IGBP1, STYX, UBLCP1, SACM1L, PTPRC, PLCD1, PLCE1, PTPRJ, DUSP10, PPP1CA, NUDT4, CDC25A, PTPN6, PTPN7, TMEM55B, DUSP27, PPTC7, PLD4, ITPK1, TNS3, PPP2R3A, PLCG2, PLCB3, CILP, PIP4K2A, SIRPA, DUSP16 |
| Erythropoietin Signaling | 2.25E00 | RPS6KB1, SRC, RELA, PTPN6, EPOR, NFKBIE, PIK3R1, NFKB1, PIK3R4, PRKCG, NFKBIA, JUN, PIK3CG, PLCG2, PRKCE, PIK3CB, PIK3CD, ELK1 |
| 3-phosphoinositide Biosynthesis | 2.24E00 | DUSP8, PTPN23, PPP1R1A, PIK3R1, NUDT12, PPFIA3, PPP2R5B, PIP4K2B, IGBP1, STYX, PIK3R4, UBLCP1, SACM1L, PTPRC, PTPRJ, DUSP10, PIK3CG, PPP1CA, NUDT4, CDC25A, PTPN6, PTPN7, DUSP27, PPTC7, ITPK1, TNS3, PI4K2A, PIP5K1C, PPP2R3A, CILP, PIK3CB, PIK3CD, PIP4K2A, SIRPA, DUSP16 |
| Sphingosine-1-phosphate Signaling | 2.23E00 | PTK2B, PIK3R1, ACER2, PIK3R4, PDGFC, PLCD1, NAAA, PLCE1, RHOT1, PIK3CG, CASQ1, PDGFRB, S1PR2, CASP3, RHOC, ASAH2, GNAI2, S1PR3, RND3, PLCG2, PLCB3, PIK3CD, PIK3CB, PDGFD, ADCY10, ADCY7 |
| RANK Signaling in Osteoclasts | 2.22E00 | MAP2K4, RELA, MAP3K15, SRC, Calm1 (includes others), PTK2B, MITF, NFKBIE, PIK3R1, CHP1, TNFRSF11A, GSN, PIK3R4, NFKB1, MAP3K12, JUN, NFKBIA, PPP3CB, PIK3CG, PIK3CB, PIK3CD, ELK1 |
| IL-17A Signaling in Gastric Cells | 2.21E00 | MAP2K4, CXCL10, RELA, JUN, IL17RC, CCL5, IL17RA, NFKB1, EGFR |
| Ovarian Cancer Signaling | 2.21E00 | FZD3, PIK3R1, FZD1, PIK3R4, CCND1, PDGFC, PRKAG1, VEGFA, ARRB1, PIK3CG, SMO, FIGF, FZD2, EGFR, TP53, RPS6KB1, SRC, GJA1, TFDP1, VEGFC, MMP2, TCF3, FZD4, E2F1, PRKACA, CD44, PIK3CB, PIK3CD, MMP9, WNT5A |
| TWEAK Signaling | 2.19E00 | TRADD, RELA, TRAF3, NFKBIA, CASP3, TNFSF12, NFKBIE, CYCS, NFKB1, BAG4, TRAF1 |
| IL-2 Signaling | 2.18E00 | IL2RG, JAK1, PTK2B, PIK3R1, PIK3R4, CSNK2A2, JUN, PIK3CG, SYK, PIK3CD, PIK3CB, CSNK2B, ELK1, JAK3, IL2RB |
| Glucocorticoid Receptor Signaling | 2.16E00 | MAP2K4, JAK1, SGK1, PIK3R1, SMAD3, PBX1, PIK3R4, FCGR1A, FGG, TSC22D3, BAG1, TGFB1, PIK3CG, SERPINE1, HSPA9, TAT, TAF4B, CD3G, TAF5, POLR2E, SMARCA2, TGFB3, PRKACA, PIK3CD, ESR1, RELA, ARID1A, POU2F2, NFKBIE, CCL5, NFKB1, HSPA1L, PRKAG1, JUN, POLR2A, NFKBIA, CCL2, PPP3CB, ANXA1, PRKAA2, GTF2H5, FKBP5, ADRB2, SRA1, ACTB, CHP1, NFATC4, CCL11, CSN2, PIK3CB, NCOR2, PLAU, ELK1, JAK3, HLTF |
| Crosstalk between Dendritic Cells and Natural Killer Cells | 2.15E00 | RELA, IL2RG, ACTB, TLN1, CD83, NFKB1, TLR9, ITGAL, CSF2RB, IL18, TLN2, MICB, CD80, CD40, FSCN1, CD86, TLR3, ACTG1, ACTA1, HLA-DRB5, PVRL2, IL2RB |
| Adipogenesis pathway | 2.14E00 | LEP, FZD3, SMAD3, FZD1, HIF1A, LPIN1, TGFB1, AGPAT2, HDAC7, SMO, GTF2H5, FZD2, TP53, HDAC9, RPS6KB1, TNFRSF1A, FGFR1, HDAC1, NFATC4, BSCL2, SLC2A4, ZNF423, EBF1, FZD4, SREBF1, KLF3, PER2, RPS6KC1, WNT5A |
| Gap Junction Signaling | 2.12E00 | DBN1, PIK3R1, PIK3R4, PRKAG1, PLCD1, PLCE1, PPP3CB, TUBA8, PIK3CG, CAV1, PRKCE, ACTA1, GUCY1B3, EGFR, SRC, ITPR2, ACTB, TUBG1, TUBB2A, TUBA4A, PRKCG, GNAI2, TUBA1A, NOV, PLCG2, GUCY1A2, PRKACA, PLCB3, PIK3CB, PIK3CD, MAPK7, ADCY10, ADCY7, ACTG1 |
| Toll-like Receptor Signaling | 2.11E00 | MAP2K4, ECSIT, RELA, TLR8, TNFAIP3, Ubb, NFKB1, TLR9, IL33, IL18, NFKBIA, JUN, TICAM1, TLR1, TRAF4, LBP, TLR3, ELK1, TRAF1 |
| Eicosanoid Signaling | 2.1E00 | PNPLA8, PLA2G4A, PTGES, LTA4H, PLA2G2E, PTGES2, PTGIR, LCAT, PLA2G2D, ABHD3, PTGER3, TBXA2R, CYSLTR1, HPGDS, TBXAS1, PTGER4, PLA2G12A |
| Triacylglycerol Biosynthesis | 2.08E00 | GPAM, ABHD5, LPIN1, PPP2R2D, AGPAT2, PPAP2B, LPIN2, DGAT1, AGPAT3, AGPAT6, ELOVL6 |
| Ketogenesis | 2.08E00 | ACAT2, HADHB, HMGCL, HMGCS2, HADHA |
| Pentose Phosphate Pathway | 2.08E00 | PGD, TKT, PGLS, TALDO1, G6PD |
| Glycogen Degradation II | 2.08E00 | PYGM, PGM1, PYGB, PYGL, AGL |
| Histidine Degradation VI | 2.08E00 | CYP7B1, MICAL1, HAL, MICAL2, CYP2E1 |
| TR/RXR Activation | 2.08E00 | PIK3R1, ME1, HIF1A, PIK3R4, DIO2, THRSP, NCOA4, ATP2A1, ENO1, F10, COL6A3, SREBF1, PIK3CG, FASN, ACACA, STRBP, PIK3CB, PIK3CD, NCOR2, FGA, PPARGC1A |
| Role of PKR in Interferon Induction and Antiviral Response | 2.05E00 | TP53, RELA, TRAF3, NFKBIA, CASP3, TNFRSF1A, NFKBIE, CYCS, TLR3, NFKB1, RNASEL, FCGR1A |
| TREM1 Signaling | 2.05E00 | ITGB1, RELA, TLR8, CIITA, ITGA5, CD83, NFKB1, TLR9, NOD2, IL18, CD40, CCL2, PLCG2, TLR1, CD86, NOD1, TLR3, NLRC4, ITGAX |
| Induction of Apoptosis by HIV1 | 2.02E00 | TP53, MAP2K4, RELA, CASP3, TNFRSF1A, NFKBIE, SLC25A3, NFKB1, TRADD, DAXX, BBC3, NFKBIA, NGFR, CYCS, SLC25A5, TRAF1 |
| Relaxin Signaling | 2.02E00 | RELA, NFKBIE, PIK3R1, GNB5, PIK3R4, NFKB1, PRKAG1, GNG7, VEGFA, GNB4, JUN, NFKBIA, GNG11, GNA15, PIK3CG, PDE11A, APEX1, GUCY1B3, PDE9A, PDE4B, GNAI2, GNAO1, GUCY1A2, PRKACA, PIK3CB, PIK3CD, ADCY10, ELK1, ADCY7, MMP9 |
| Aldosterone Signaling in Epithelial Cells | 2.01E00 | SGK1, PIK3R1, PIP4K2B, DNAJC15, PIK3R4, SLC9A1, HSPA1L, PLCD1, PLCE1, HSPA12B, PIK3CG, HSPE1, PRKCE, HSPB6, HSPA4L, DNAJB8, ITPR2, HSPA9, HSPB9, DNAJB9, HSPA12A, PRKCG, DNAJC21, DNAJC5, PIP5K1C, PLCG2, DNAJC18, PLCB3, PIK3CB, PIK3CD, DNAJB6, PIP4K2A, DNAJC7 |
| Stearate Biosynthesis I (Animals) | 1.99E00 | ACSL3, ACOT2, FAM213B, CYP2E1, PPT1, FASN, ACOT1, ACOT4, SLC27A1, ACSL1, ELOVL6 |
| Acetyl-CoA Biosynthesis I (Pyruvate Dehydrogenase Complex) | 1.98E00 | PDHA1, DLAT, DLD, PDHB |
| Pregnenolone Biosynthesis | 1.98E00 | CYP7B1, MICAL1, MICAL2, CYP2E1 |
| Chemokine Signaling | 1.97E00 | SRC, CCR5, CAMK1, Calm1 (includes others), PTK2B, CAMK1D, MYL2, LIMK2, CCL5, CCL11, LIMK1, GNAI2, ROCK2, JUN, CCL2, PIK3CG, PLCG2, PLCB3 |
| Uracil Degradation II (Reductive) | 1.97E00 | DPYSL2, DPYD, UPB1 |
| Pentose Phosphate Pathway (Oxidative Branch) | 1.97E00 | PGD, PGLS, G6PD |
| Thymine Degradation | 1.97E00 | DPYSL2, DPYD, UPB1 |
| D-myo-inositol (1, 4, 5)-Trisphosphate Biosynthesis | 1.97E00 | PLD4, PLCD1, PLCE1, PI4K2A, PIP5K1C, PLCG2, PIP4K2B, PLCB3, PIP4K2A |
| MIF Regulation of Innate Immunity | 1.96E00 | MAP2K4, TP53, PLA2G4A, RELA, JUN, PLA2G2E, NFKBIA, PLA2G2D, NFKBIE, CD74, NFKB1, PLA2G12A |
| Angiopoietin Signaling | 1.96E00 | RELA, ANGPT2, GRB14, DOK2, NFKBIE, PIK3R1, NFKB1, PIK3R4, TIE1, ANGPTL1, PAK1, NFKBIA, PIK3CG, ANGPT4, PIK3CB, PIK3CD, TEK |
| EGF Signaling | 1.95E00 | MAP2K4, RPS6KB1, SRC, JAK1, ITPR2, PIK3R1, PIK3R4, CSNK2A2, JUN, PIK3CG, PIK3CD, PIK3CB, CSNK2B, ELK1, EGFR |
| JAK/Stat Signaling | 1.91E00 | RELA, STAT6, PTPN6, JAK1, PIAS2, PIK3R1, SOCS6, PIK3R4, NFKB1, PIAS3, JUN, PIK3CG, SOCS2, PIK3CB, STAT2, PIK3CD, JAK3, SOCS5 |
| UVA-Induced MAPK Signaling | 1.9E00 | TP53, MAP2K4, RPS6KB1, PARP6, CASP3, PIK3R1, ART1, RPS6KA3, ZC3HAV1, PIK3R4, PLCD1, PLCE1, JUN, PIK3CG, PLCG2, PLCB3, RPS6KA4, PIK3CB, PIK3CD, CYCS, EGFR |
| Regulation of the Epithelial-Mesenchymal Transition Pathway | 1.87E00 | MAP2K4, RELA, ADAM17, JAK1, FZD3, PIK3R1, SMAD3, BCL9, FZD1, HIF1A, NFKB1, PIK3R4, FGF13, TGFB1, PIK3CG, SMO, FZD2, PDGFRB, EGFR, NOTCH3, FGFR1, FGF14, MMP2, ZEB1, TCF3, PYGO2, CDH2, FZD4, ZEB2, TGFB3, PIK3CB, FGF11, RBPJ, PIK3CD, PDGFD, JAK3, MMP9, WNT5A |
| LPS/IL-1 Mediated Inhibition of RXR Function | 1.87E00 | MAP2K4, ECSIT, ALDH4A1, APOE, SLC10A1, CHST7, HS2ST1, HMGCS2, SOD3, CHST15, JUN, UST, ALDH1A3, RARA, NGFR, Gm4846, FABP5, FMO1, CPT1C, LBP, IL1RAP, FMO4, ALDH7A1, GSTA3, MGST1, ACSL3, CPT1A, TNFRSF1A, IL1R1, ALDH9A1, GSTO1, IL33, HS3ST3B1, IL18, SULT1E1, MGST2, SREBF1, ALDH3B1, SLC27A1, PLTP, NDST1, ACSL1, PPARGC1A, MAOA |
| SAPK/JNK Signaling | 1.87E00 | TP53, MAP2K4, RAC2, DUSP8, PIK3R1, PIK3R4, MAPK8IP1, GNG7, DAXX, TRADD, MINK1, MAP3K12, JUN, GNG11, PIK3CG, DUSP10, IRS1, FCER1G, MAP4K1, PIK3CB, PIK3CD, ELK1 |
| STAT3 Pathway | 1.85E00 | MAP2K4, SRC, PTPN6, TGFBR3, FGFR1, FLT4, SOCS6, TNFRSF11A, MAP3K12, PIAS3, NTRK2, PIM1, NGFR, SOCS2, SOCS5, EGFR, CDC25A, PDGFRB |
| G-Protein Coupled Receptor Signaling | 1.83E00 | PIK3R1, PIK3R4, ADRB3, GNA15, PIK3CG, RGS10, ADORA2B, PDE11A, APEX1, APLNR, CNR1, RAPGEF3, PDE4B, S1PR3, DUSP9, NPR3, GNAO1, PRKACA, PLCB3, PIK3CD, ADCY10, RELA, FYN, GPR17, RGS18, PTK2B, PTGER3, NFKBIE, TBXA2R, NFKB1, AVPR1A, PRKAG1, NFKBIA, PRKCE, ADORA1, PTGER4, ADRB2, SRC, RGS2, PTGIR, PDE9A, RGS7, XCR1, PRKCG, GNAI2, P2RY13, PTH1R, P2RY12, PIK3CB, ADCY7 |
| Role of JAK1 and JAK3 in γc Cytokine Signaling | 1.82E00 | STAT6, IL2RG, JAK1, PTK2B, FES, PIK3R1, PIK3R4, TSLP, IRS1, PIK3CG, SYK, SH2B2, PIK3CB, PIK3CD, JAK3, IL2RB |
| Ephrin Receptor Signaling | 1.82E00 | RAC2, FYN, RGS3, ITSN1, EPHB2, GNB5, LIMK2, BCAR1, PDGFC, GNG7, LIMK1, VEGFA, ROCK2, GNB4, EFNB2, PAK1, GNG11, GNA15, CFL2, EFNA5, PIK3CG, EFNB1, FIGF, ITGA4, ITGB1, SRC, EPHB4, ARPC5L, VEGFC, ITGA5, GNAI2, WIPF1, WAS, GNAO1, EPHB3, PDGFD |
| Phospholipases | 1.81E00 | PLCD1, PLD4, PNPLA8, PLA2G4A, PLD2, PLA2G2E, PLCE1, LCAT, PLA2G2D, ABHD3, PLCG2, PLCB3, LIPG, PLA1A, PLA2G12A |
| UVB-Induced MAPK Signaling | 1.8E00 | MAP2K4, TP53, RPS6KB1, PIK3R1, HIST1H3C, RPS6KA3, PIK3R4, PRKCG, JUN, PIK3CG, PRKCE, PIK3CB, PIK3CD, EGFR |
| Glioblastoma Multiforme Signaling | 1.79E00 | FZD3, PIK3R1, FZD1, PIK3R4, CCND1, PDGFC, PLCD1, PLCE1, IGF1, RHOT1, PIK3CG, SMO, FZD2, EGFR, PDGFRB, TP53, RPS6KB1, SRC, RHOC, ITPR2, TCF3, CCNE1, FZD4, RND3, PLCG2, E2F1, PLCB3, PIK3CB, PIK3CD, PDGFD, WNT5A |
| TNFR2 Signaling | 1.75E00 | MAP2K4, TANK, RELA, JUN, NFKBIA, NFKBIE, TNFAIP3, NFKB1, TRAF1 |
| IL-17A Signaling in Airway Cells | 1.75E00 | MAP2K4, RELA, JAK1, PIK3R1, NFKBIE, IL17RC, IL17RA, NFKB1, PIK3R4, CCL11, NFKBIA, PIK3CG, PIK3CB, MUC5B, PIK3CD, JAK3 |
| Huntington's Disease Signaling | 1.74E00 | MAP2K4, SDHB, SGK1, PIK3R1, CLTB, GNB5, Ubb, RCOR2, PIK3R4, HSPA1L, GNG7, GNB4, GNG11, POLR2A, JUN, GNA15, IGF1, PIK3CG, CASQ1, HDAC7, DLG4, PRKCE, NAPA, EGFR, SDHA, ATP5J, TP53, HDAC9, CASP3, HSPA9, HDAC1, DNM3, HIP1, PRKCG, DNM1, DNAJC5, ATP5B, POLR2E, PENK, PSME4, PLCB3, PIK3CB, PIK3CD, CYCS, NCOR2 |
| p70S6K Signaling | 1.73E00 | JAK1, F2R, PIK3R1, PPP2R5B, PIK3R4, F2, PLCD1, PLCE1, PIK3CG, PRKCE, EGFR, RPS6KB1, SRC, IL2RG, CD79B, CD79A, PRKCG, GNAI2, PPP2R3A, PLCG2, IRS1, SYK, LYN, PLCB3, PIK3CB, PIK3CD |
| Tryptophan Degradation to 2-amino-3-carboxymuconate Semialdehyde | 1.73E00 | KMO, TDO2, HAAO, IDO1 |
| Lymphotoxin β Receptor Signaling | 1.73E00 | RELA, TRAF3, CASP3, PIK3R1, RELB, NFKB1, PIK3R4, NFKBIA, PIK3CG, TRAF4, PIK3CD, CYCS, PIK3CB, TRAF1 |
| Role of IL-17A in Arthritis | 1.73E00 | MAP2K4, RELA, PIK3R1, NFKBIE, IL17RC, CCL5, IL17RA, NFKB1, PIK3R4, NFKBIA, CCL2, PIK3CG, PIK3CB, PIK3CD |
| TNFR1 Signaling | 1.72E00 | TANK, MAP2K4, TRADD, RELA, PAK1, NFKBIA, JUN, CASP3, TNFRSF1A, NFKBIE, TNFAIP3, CYCS, NFKB1 |
| Death Receptor Signaling | 1.69E00 | MAP2K4, TNFRSF21, PARP6, RELA, CASP3, TNFRSF1A, NFKBIE, ACTB, ART1, ZC3HAV1, NFKB1, ARHGDIB, LIMK1, TANK, TRADD, DAXX, NFKBIA, TNFSF12, CYCS, ACTG1, ACTA1 |
| Glycogen Degradation III | 1.69E00 | PYGM, PGM1, PYGB, PYGL, AGL |
| Epoxysqualene Biosynthesis | 1.68E00 | SQLE, FDFT1 |
| Endothelin-1 Signaling | 1.68E00 | PLD2, PIK3R1, PIK3R4, PLCD1, PLCE1, JUN, LCAT, GNA15, PIK3CG, CASQ1, PRKCE, GUCY1B3, PLA2G12A, PNPLA8, SRC, CASP3, ABHD3, ITPR2, MAPK6, PRKCG, PLD4, GNAI2, PLA2G4A, PLA2G2E, PLA2G2D, PLCG2, GNAO1, SHC2, GUCY1A2, PLCB3, PIK3CB, PIK3CD, MAPK7, ADCY10, ADCY7 |
| Renin-Angiotensin Signaling | 1.67E00 | MAP2K4, RELA, PTPN6, PTK2B, ITPR2, PIK3R1, CCL5, PIK3R4, NFKB1, PRKAG1, PRKCG, PAK1, JUN, CCL2, PIK3CG, PLCG2, SHC2, PRKACA, PRKCE, PIK3CB, PIK3CD, ADCY10, ELK1, ADCY7 |
| VEGF Family Ligand-Receptor Interactions | 1.67E00 | NRP2, PIK3R1, FLT4, VEGFC, PIK3R4, PRKCG, VEGFA, PLA2G4A, PLA2G2E, PLA2G2D, PLCG2, PIK3CG, PRKCE, FIGF, PIK3CB, PIK3CD, NRP1, PLA2G12A |
| Amyotrophic Lateral Sclerosis Signaling | 1.67E00 | TP53, CACNA1S, CASP3, PIK3R1, GPX1, VEGFC, SOD1, PIK3R4, PDGFC, CACNA1A, VEGFA, PAK1, CACNA1E, IGF1, PIK3CG, SLC1A2, GLUL, FIGF, PIK3CB, PIK3CD, CYCS, GRIA3 |
| Sonic Hedgehog Signaling | 1.66E00 | DYRK1B, GLI2, GLIS2, GLI3, SUFU, PRKACA, SMO, PRKAG1, PTCH2 |
| IL-3 Signaling | 1.65E00 | GAB2, STAT6, PTPN6, JAK1, PIK3R1, CHP1, PIK3R4, PRKCG, CSF2RB, PAK1, JUN, PPP3CB, PIK3CG, PRKCE, PIK3CB, PIK3CD, ELK1 |
| PEDF Signaling | 1.65E00 | TP53, RELA, ARHGAP22, PIK3R1, NFKBIE, SERPINF1, ZEB1, NFKB1, PIK3R4, PNPLA2, ROCK2, SOD2, NFKBIA, PIK3CG, PIK3CB, PIK3CD, ELK1 |
| Role of JAK2 in Hormone-like Cytokine Signaling | 1.64E00 | PTPN6, JAK1, EPOR, IRS1, SOCS6, SH2B2, SOCS2, HLTF, SOCS5, SIRPA |
| B Cell Activating Factor Signaling | 1.64E00 | MAP2K4, RELA, TRAF3, JUN, NFKBIA, NFKBIE, NFATC4, NFKB1, ELK1, TNFSF13B, TRAF1 |
| Serine Biosynthesis | 1.62E00 | PSAT1, PSPH, PHGDH |
| Cardiac β-adrenergic Signaling | 1.62E00 | AKAP12, CACNA1S, PPP1R1A, PPP2R5B, GNB5, PPP1R3A, PRKAG1, GNG7, ATP2A1, AKAP2, GNB4, GNG11, CACNA1E, PDE11A, PPP1CA, APEX1, PDE9A, SLC8A3, ATP2A3, AKAP6, PDE4B, CACNA1A, PPP1R3D, PPP2R3A, PRKACA, SLC8A1, ADCY10, ADCY7 |
| PDGF Signaling | 1.62E00 | MAP2K4, SRC, JAK1, PIK3R1, PIK3R4, PDGFC, CSNK2A2, JUN, PLCG2, PIK3CG, CAV1, PIK3CB, PIK3CD, CSNK2B, PDGFD, ELK1, JAK3, PDGFRB |
| IL-17 Signaling | 1.59E00 | MAP2K4, RELA, JAK1, PIK3R1, IL17RC, CCL11, IL17RA, NFKB1, PIK3R4, CXCL10, JUN, CCL2, PIK3CG, PIK3CB, MUC5B, PIK3CD, ELK1 |
| Basal Cell Carcinoma Signaling | 1.59E00 | TP53, GLI2, FZD3, FZD1, TCF3, FZD4, KIF7, GLIS2, GLI3, APC2, SUFU, SMO, BMP6, FZD2, PTCH2, BMP1, WNT5A |
| Wnt/Ca+ pathway | 1.59E00 | PLCD1, RELA, FZD4, PLCE1, FZD3, PLCG2, SMO, PLCB3, FZD1, NFATC4, NFKB1, ROR1, FZD2, WNT5A |
| Human Embryonic Stem Cell Pluripotency | 1.58E00 | FZD3, SMAD3, PIK3R1, FZD1, PIK3R4, PDGFC, NOG, TGFB1, PIK3CG, SMO, FZD2, PDGFRB, BMP1, S1PR2, FGFR1, SMAD6, SMAD7, TCF3, INHBA, S1PR3, NTRK2, FZD4, TGFB3, PIK3CB, PIK3CD, BMP6, PDGFD, WNT5A |
| CCR3 Signaling in Eosinophils | 1.57E00 | Calm1 (includes others), PIK3R1, GNB5, LIMK2, PIK3R4, GNG7, LIMK1, ROCK2, GNB4, PAK1, GNG11, CFL2, PIK3CG, PRKCE, PLA2G12A, ITPR2, CCL11, PRKCG, GNAI2, PLA2G4A, PLA2G2E, PLA2G2D, PLCB3, PIK3CD, PIK3CB |
| FcγRIIB Signaling in B Lymphocytes | 1.56E00 | MAP2K4, CD79B, PLCG2, PIK3CG, SYK, PIK3R1, LYN, PIK3CB, CD79A, PIK3CD, PIK3R4 |
| LPS-stimulated MAPK Signaling | 1.54E00 | MAP2K4, RELA, ATF1, NFKBIE, PIK3R1, NFKB1, PIK3R4, PRKCG, PAK1, NFKBIA, JUN, PIK3CG, PRKCE, PIK3CB, PIK3CD, LBP, ELK1 |
| PTEN Signaling | 1.53E00 | RELA, RAC2, TGFBR3, PIK3R1, NFKB1, CCND1, BCAR1, NGFR, PIK3CG, CSNK2B, ITGA4, EGFR, PDGFRB, ITGB1, RPS6KB1, CASP3, FGFR1, FLT4, ITGA5, TNFRSF11A, CSNK2A2, NTRK2, MAGI1, PIK3CD, PIK3CB |
| Prostanoid Biosynthesis | 1.53E00 | PTGES, PTGES2, HPGDS, TBXAS1 |
| Ketolysis | 1.53E00 | ACAT2, HADHB, OXCT1, HADHA |
| P2Y Purigenic Receptor Signaling Pathway | 1.49E00 | RELA, PIK3R1, GNB5, NFKB1, PIK3R4, GNG7, PRKAG1, PRKCG, GNAI2, PLCD1, GNB4, PLCE1, JUN, GNG11, P2RY6, PLCG2, PIK3CG, PRKACA, P2RY12, PLCB3, PRKCE, PIK3CD, PIK3CB, ADCY10, ADCY7 |
| IL-1 Signaling | 1.47E00 | MAP2K4, ECSIT, RELA, NFKBIE, GNB5, IL1R1, NFKB1, GNG7, PRKAG1, GNAI2, GNB4, JUN, GNG11, NFKBIA, GNA15, GNAO1, PRKACA, ADCY10, IL1RAP, ADCY7 |
| CCR5 Signaling in Macrophages | 1.46E00 | MAP2K4, CCR5, Calm1 (includes others), PTK2B, GNB5, CCL5, GNG7, PRKCG, GNAI2, GNB4, CD3G, JUN, GNG11, PLCG2, FCER1G, PRKCE |
| Phosphatidylglycerol Biosynthesis II (Non-plastidic) | 1.45E00 | GPAM, ABHD5, AGPAT2, PGS1, AGPAT3, AGPAT6 |
| Gαi Signaling | 1.45E00 | GPR17, SRC, APLNR, PTGER3, CNR1, RGS7, RALB, TBXA2R, GNB5, GNG7, PRKAG1, XCR1, S1PR3, GNAI2, P2RY13, GNB4, GNG11, NPR3, RGS10, PRKACA, CAV1, P2RY12, ADCY10, ADORA1, ADCY7 |
| PPARα/RXRα Activation | 1.44E00 | MAP2K4, RELA, GPD1, SMAD3, APOA2, TGFBR3, NFKBIE, Cyp2c44, NFKB1, PRKAG1, PLCD1, NFKBIA, PLCE1, JUN, GNA15, GPD2, TGFB1, FASN, PRKAA2, IL1RAP, ITGB5, IL1R1, ACADL, APOA1, IRS1, PLCG2, PRKACA, TGFB3, PLCB3, SLC27A1, NCOR2, ADIPOR2, ADCY10, ADCY7, PPARGC1A |
| AMPK Signaling | 1.44E00 | CAB39, LEP, ARID1A, PIK3R1, PPP2R5B, PFKL, PIK3R4, CCND1, PRKAG1, ADRB3, FASN, PIK3CG, PRKAA2, CPT1C, ADRB2, PFKFB3, RPS6KB1, SRC, CPT1A, ACACB, CKM, ACTB, SLC2A4, PFKM, SMARCA2, PPP2R3A, IRS1, PRKACA, PIK3CB, ACACA, PIK3CD, MLYCD, AK2, HLTF, PPARGC1A |
| Graft-versus-Host Disease Signaling | 1.43E00 | IL33, IL18, HLA-DOA, CD80, HLA-DMA, HLA-DMB, HLA-DQA1, FCER1G, CD86, HLA-DOB, HLA-DQB1, HLA-DRB5 |
| p53 Signaling | 1.41E00 | TP53, HDAC9, TP53INP1, TP63, PLAGL1, PIK3R1, HDAC1, HIF1A, PIK3R4, CCND1, SERPINE2, CCNG1, JUN, BBC3, THBS1, PIK3CG, E2F1, ADCK3, PIK3CB, PIK3CD, PML |
| Sperm Motility | 1.41E00 | PNPLA8, SLC9A5, Calm1 (includes others), PTK2B, ITPR2, ABHD3, PDE4B, PRKAG1, PRKCG, PLCD1, PLA2G4A, PLA2G2E, PLCE1, PLA2G2D, LCAT, PLCG2, PRKACA, GUCY1A2, PLCB3, PRKCE, MST1R, CATSPER2, ADCY10, GUCY1B3, PLA2G12A |
| April Mediated Signaling | 1.41E00 | MAP2K4, RELA, TRAF3, JUN, NFKBIA, NFKBIE, NFATC4, NFKB1, ELK1, TRAF1 |
| MIF-mediated Glucocorticoid Regulation | 1.4E00 | PLA2G4A, RELA, PLA2G2E, NFKBIA, PLA2G2D, NFKBIE, CD74, NFKB1, PLA2G12A |
| α-Adrenergic Signaling | 1.4E00 | Calm1 (includes others), ITPR2, SLC8A3, GNB5, PYGB, PYGL, PRKAG1, GNG7, PRKCG, GNAI2, GNB4, GNG11, PYGM, PLCG2, PRKACA, PRKCE, ADCY10, SLC8A1, ADCY7 |
| Bladder Cancer Signaling | 1.4E00 | TP53, TFDP1, FGF14, MMP15, VEGFC, MMP2, PDGFC, CCND1, FGF13, VEGFA, MMP23B, THBS1, E2F1, FIGF, FGF11, MMP12, MMP9, EGFR, RASSF1 |
| Colanic Acid Building Blocks Biosynthesis | 1.39E00 | GPI, UGP2, PMM1, GALE, MPI |
| HER-2 Signaling in Breast Cancer | 1.39E00 | TP53, ITGB1, PIK3R1, MMP2, PIK3R4, CCND1, ITGB7, PRKCG, ITGB2, CCNE1, PIK3CG, PRKCE, PIK3CB, PIK3CD, PARD3, ITGB5, EGFR |
| Hypoxia Signaling in the Cardiovascular System | 1.38E00 | TP53, UBE2A, COPS5, NFKBIE, HIF1A, SLC2A4, UBE2F, VEGFA, UBE2D4, JUN, NFKBIA, CDC34, LDHA, VHL, UBE2D3 |
| Chondroitin and Dermatan Biosynthesis | 1.37E00 | CHPF, CSGALNACT1, CHPF2 |
| Ceramide Degradation | 1.37E00 | NAAA, ACER2, ASAH2 |
| Glycogen Biosynthesis II (from UDP-D-Glucose) | 1.37E00 | UGP2, GYG1, GBE1 |
| GDP-mannose Biosynthesis | 1.37E00 | GPI, PMM1, MPI |
| HGF Signaling | 1.36E00 | MAP2K4, ITGB1, MAP3K15, PIK3R1, ITGA5, PIK3R4, CCND1, PRKCG, ELF4, DOCK1, MAP3K12, PAK1, JUN, ETS2, PIK3CG, PLCG2, PRKCE, PIK3CB, PIK3CD, ELK1, ELK3, ITGA4 |
| Androgen Signaling | 1.35E00 | RELA, SRC, Calm1 (includes others), SMAD3, GNB5, NFKB1, CCND1, PRKAG1, GNG7, NCOA4, PRKCG, GNAI2, GNB4, TGFB1I1, JUN, POLR2A, GNG11, GNA15, POLR2E, GNAO1, PRKACA, PRKCE, GTF2H5 |
| p38 MAPK Signaling | 1.34E00 | TP53, MAP2K4, RPS6KB1, ATF1, TNFRSF1A, HIST1H3C, HMGN1, RPS6KA3, IL1R1, IL33, TRADD, DAXX, PLA2G4A, IL18, PLA2G2E, PLA2G2D, TGFB1, DUSP10, TGFB3, RPS6KA4, MAP4K1, ELK1, IL1RAP, PLA2G12A |
| Docosahexaenoic Acid (DHA) Signaling | 1.33E00 | CASP3, PIK3CG, PIK3R1, SERPINF1, PIK3CB, PIK3CD, CYCS, PIK3R4, PNPLA2, APP |
| Neuropathic Pain Signaling In Dorsal Horn Neurons | 1.33E00 | SRC, CAMK1, CAMK1D, ITPR2, PIK3R1, PIK3R4, PRKAG1, PRKCG, TACR1, PLCD1, PLCE1, NTRK2, PIK3CG, PLCG2, PRKACA, PLCB3, PRKCE, PIK3CB, PIK3CD, ELK1, GRIA3 |
| IL-12 Signaling and Production in Macrophages | 1.33E00 | MAP2K4, RELA, APOE, APOA4, APOB, APOA2, PIK3R1, MAF, PIK3R4, NFKB1, PON1, JUN, TGFB1, PIK3CG, PRKCE, SERPINA1, STAT6, PRKCG, ALB, IL18, APOA1, CD40, TGFB3, PIK3CB, PIK3CD, IRF8, MST1R |
| 3-phosphoinositide Degradation | 1.32E00 | DUSP8, PTPN23, PPP1R1A, NUDT12, PPFIA3, PPP2R5B, MTMR1, IGBP1, STYX, UBLCP1, SACM1L, PTPRC, PTPRJ, DUSP10, NUDT4, PPP1CA, CDC25A, PTPN7, PTPN6, TMEM55B, MTMR14, DUSP27, PPTC7, ITPK1, TNS3, PPP2R3A, CILP, SIRPA, DUSP16 |
| Dopamine Degradation | 1.32E00 | ALDH4A1, ALDH1A3, COMT, Sult1a1, ALDH9A1, ALDH7A1, MAOA |
| Glutathione-mediated Detoxification | 1.32E00 | GSTA3, MGST1, MGST2, HPGDS, Gsta4, ANPEP, Gstt1, GSTO1 |

Supplementary Table 5. Overview of pathways clustered according to biological function. Pathway recruitment referred to those pathways that increased their significance with increasing CR level (identified by IPA based on amount of DEGs and expression levels). Deregulated refers to those pathways that decreased their significance with increasing CR level and were non-significant at 40CR relative to 12AL function recruited with increasing CR. The summarized pathways contain multiple gene sets. Hence, if one gene set is increased in expression while the other is decreased, this would result in a summarised pathway to be both recruited and deregulated with CR.

| **Summarized pathways** | **Recruited with CR** | **Deregulated with CR** |
| --- | --- | --- |
| Adherens Junctions | X |  |
| Adipogenesis | X |  |
| Amino acid metabolism |  | X |
| AMPK signalling | X |  |
| Apoptosis | X |  |
| cAMP-mediated signalling |  | X |
| Carbohydrate and energy metabolism |  | X |
| Cholecystokinin/Gastrin-mediated Signalling |  | X |
| Cholesterol biosynthesis |  | X |
| Corticotropin Releasing Hormone Signalling |  | X |
| Electrolyte transport | X |  |
| Ephrin signalling | X |  |
| Estrogen biosynthesis |  | X |
| Fatty Acid and Lipid metabolism | X | X |
| Germ cell development | X |  |
| Glucocorticoid signalling | X |  |
| G-Protein Signalling | X | X |
| Hedgehog signalling | X |  |
| HIF1a/hypoxia signalling | X |  |
| Human diseases signalling | X | X |
| Interleukin signalling | X |  |
| Immune and inflammation response | X | X |
| Inositol phosphate metabolism | X |  |
| Integrin signalling | X |  |
| MAPK signalling | X | X |
| Melatonin signalling |  | X |
| NF-ĸB signalling | X |  |
| Notch signalling |  | X |
| Oncogenic signalling pathway | X |  |
| Oxidative stress response | X | X |
| p53 signalling | X |  |
| Protein Ubiquitination Pathway | X |  |
| Relaxin Signalling | X |  |
| RXR signalling |  | X |
| Sphingolipid Synthesis and Metabolism | X |  |
| Synaptogenesis | X |  |
| Vascular biology | X | X |
| Wnt signalling | X |  |
| β-adrenergic signalling | X |  |

Supplementary Table 6. Classification of pathways recruited with increasing CR.

| **pathway** | **clustered** | **Recruited** | **Non-recruited** |
| --- | --- | --- | --- |
| Colanic_Acid_Building_Blocks_Biosynthesis | ? | X |  |
| GDP-mannose_Biosynthesis | ? | X |  |
| Neuropathic_Pain_Signaling_In_Dorsal_Horn_Neurons | ? | X |  |
| Regulation_of_the_Epithelial-Mesenchymal_Transition_Pathway | ? | X |  |
| 4-hydroxybenzoate_Biosynthesis | ? |  | X |
| Biotin-carboxyl_Carrier_Protein_Assembly | ? |  | X |
| Remodeling_of_Epithelial_Adherens_Junctions | Adherens Junctions | X |  |
| Adipogenesis_pathway | Adipogenesis | X |  |
| Glutamine_Biosynthesis_I | Amino acid metabolism |  | X |
| 4-hydroxyphenylpyruvate_Biosynthesis | Amino acid metabolism |  | X |
| Superpathway_of_Citrulline_Metabolism | Amino acid metabolism |  | X |
| Phenylalanine_Degradation_I_(Aerobic) | Amino acid metabolism |  | X |
| AMPK_Signaling | AMPK signalling | X |  |
| Calcium-induced_T_Lymphocyte_Apoptosis | Apoptosis | X |  |
| Induction_of_Apoptosis_by_HIV1 | Apoptosis | X |  |
| Nur77_Signaling_in_T_Lymphocytes | Apoptosis | X |  |
| cAMP-mediated_signaling | cAMP-mediated signaling |  | X |
| Acetyl-CoA_Biosynthesis_III_(from_Citrate) | carbohydrate and energy metabolism |  | X |
| Acyl-CoA_Hydrolysis | carbohydrate and energy metabolism |  | X |
| Urea_Cycle | carbohydrate and energy metabolism |  | X |
| Coenzyme_A_Biosynthesis | carbohydrate and energy metabolism |  | X |
| NAD_biosynthesis_II_(from_tryptophan) | carbohydrate and energy metabolism |  | X |
| NAD_Biosynthesis_III | carbohydrate and energy metabolism |  | X |
| Glycerol-3-phosphate_Shuttle | carbohydrate and energy metabolism |  | X |
| Methylglyoxal_Degradation_VI | carbohydrate and energy metabolism |  | X |
| Cholecystokinin/Gastrin-mediated_Signaling | Cholecystokinin/Gastrin-mediated Signaling |  | X |
| Lanosterol_Biosynthesis | Cholesterol biosynthesis |  | X |
| Corticotropin_Releasing_Hormone_Signaling | Corticotropin Releasing Hormone Signaling |  | X |
| Aldosterone_Signaling_in_Epithelial_Cells | electrolyte transport | X |  |
| Ephrin_A_Signaling | Ephrin signalling | X |  |
| Ephrin_B_Signaling | Ephrin signalling | X |  |
| Ephrin_Receptor_Signaling | Ephrin signalling | X |  |
| Estrogen_Biosynthesis | Estrogen biosynthesis |  | X |
| 3-phosphoinositide_Biosynthesis | Fatty Acid and Lipid metabolism | X |  |
| Docosahexaenoic_Acid_(DHA)_Signaling | Fatty Acid and Lipid metabolism | X |  |
| Eicosanoid_Signaling | Fatty Acid and Lipid metabolism | X |  |
| Fatty_Acid_Activation | Fatty Acid and Lipid metabolism |  | X |
| γ-linolenate_Biosynthesis_II_(Animals) | Fatty Acid and Lipid metabolism |  | X |
| Tyrosine_Degradation_I | Fatty Acid and Lipid metabolism |  | X |
| Branched-chain_α-keto_acid_Dehydrogenase_Complex | Fatty Acid and Lipid metabolism |  | X |
| Methylthiopropionate_Biosynthesis | Fatty Acid and Lipid metabolism |  | X |
| Mitochondrial_L-carnitine_Shuttle_Pathway | Fatty Acid and Lipid metabolism |  | X |
| Glycerol_Degradation_I | Fatty Acid and Lipid metabolism |  | X |
| Pentose_Phosphate_Pathway_(Non-oxidative_Branch) | Fatty Acid and Lipid metabolism |  | X |
| Trans,_trans-farnesyl_Diphosphate_Biosynthesis | Fatty Acid and Lipid metabolism |  | X |
| CDP-diacylglycerol_Biosynthesis_I | Fatty Acid and Lipid metabolism |  | X |
| Germ_Cell-Sertoli_Cell_Junction_Signaling | germ cell development | X |  |
| Sertoli_Cell-Sertoli_Cell_Junction_Signaling | germ cell development | X |  |
| Glucocorticoid_Receptor_Signaling | Glucocorticoid signalling | X |  |
| MIF-mediated_Glucocorticoid_Regulation | Glucocorticoid signalling | X |  |
| G-Protein_Coupled_Receptor_Signaling | G-Protein Signaling | X |  |
| Gα12/13_Signaling | G-Protein Signaling | X |  |
| Gαi_Signaling | G-Protein Signaling | X |  |
| Rac_Signaling | G-Protein Signaling | X |  |
| Regulation_of_Actin-based_Motility_by_Rho | G-Protein Signaling | X |  |
| Gαs_Signaling | G-Protein Signaling |  | X |
| GPCR-Mediated_Nutrient_Sensing_in_Enteroendocrine_Cells | G-Protein Signaling |  | X |
| G_Beta_Gamma_Signaling | G-Protein Signaling |  | X |
| Sonic_Hedgehog_Signaling | Hedgehog signalling | X |  |
| HIF1α_Signaling | HIF1a/hypoxia signalling | X |  |
| Hypoxia_Signaling_in_the_Cardiovascular_System | HIF1a/hypoxia signalling | X |  |
| Graft-versus-Host_Disease_Signaling | human diseases signalling | X |  |
| Huntington's_Disease_Signaling | human diseases signalling | X |  |
| Ovarian_Cancer_Signaling | human diseases signalling | X |  |
| Pancreatic_Adenocarcinoma_Signaling | human diseases signalling | X |  |
| Type_I_Diabetes_Mellitus_Signaling | human diseases signalling | X |  |
| Type_II_Diabetes_Mellitus_Signaling | human diseases signalling | X |  |
| Colorectal_Cancer_Metastasis_Signaling | human diseases signalling | X |  |
| Amyotrophic_Lateral_Sclerosis_Signaling | human diseases signalling | X |  |
| Human_Embryonic_Stem_Cell_Pluripotency | human diseases signalling | X |  |
| Role_of_Osteoblasts,_Osteoclasts_and_Chondrocytes_in_Rheumatoid_Arthritis | human diseases signalling | X |  |
| Maturity_Onset_Diabetes_of_Young_(MODY)_Signaling | human diseases signalling |  | X |
| Synaptic_Long_Term_Depression | human diseases signalling |  | X |
| Factors_Promoting_Cardiogenesis_in_Vertebrates | human diseases signalling |  | X |
| Hepatic_Cholestasis | human diseases signalling |  | X |
| Neuroprotective_Role_of_THOP1_in_Alzheimer's_Disease | human diseases signalling |  | X |
| IL-17A_Signaling_in_Gastric_Cells | IL signalling | X |  |
| IL-2_Signaling | IL signalling | X |  |
| IL-4_Signaling | IL signalling | X |  |
| IL-9_Signaling | IL signalling | X |  |
| Regulation_of_IL-2_Expression_in_Activated_and_Anergic_T_Lymphocytes | IL signalling | X |  |
| Crosstalk_between_Dendritic_Cells_and_Natural_Killer_Cells | Immune and inflammation response | X |  |
| MIF_Regulation_of_Innate_Immunity | Immune and inflammation response | X |  |
| MSP-RON_Signaling_Pathway | Immune and inflammation response | X |  |
| PI3K_Signaling_in_B_Lymphocytes | Immune and inflammation response | X |  |
| PKCθ_Signaling_in_T_Lymphocytes | Immune and inflammation response | X |  |
| Agranulocyte_Adhesion_and_Diapedesis | Immune and inflammation response | X |  |
| Altered_T_Cell_and_B_Cell_Signaling_in_Rheumatoid_Arthritis | Immune and inflammation response | X |  |
| B_Cell_Receptor_Signaling | Immune and inflammation response | X |  |
| CCR3_Signaling_in_Eosinophils | Immune and inflammation response | X |  |
| CD28_Signaling_in_T_Helper_Cells | Immune and inflammation response | X |  |
| Chemokine_Signaling | Immune and inflammation response | X |  |
| iCOS-iCOSL_Signaling_in_T_Helper_Cells | Immune and inflammation response | X |  |
| T_Helper_Cell_Differentiation | Immune and inflammation response | X |  |
| Role_of_JAK1_and_JAK3_in_γc_Cytokine_Signaling | Immune and inflammation response | X |  |
| Role_of_NFAT_in_Regulation_of_the_Immune_Response | Immune and inflammation response | X |  |
| Mechanisms_of_Viral_Exit_from_Host_Cells | Immune and inflammation response |  | X |
| Antigen_Presentation_Pathway | Immune and inflammation response |  | X |
| ErbB4_Signaling | Immune and inflammation response |  | X |
| D-myo-inositol_(1,4,5)-Trisphosphate_Biosynthesis | Inositol phosphate metabolism | X |  |
| D-myo-inositol-5-phosphate_Metabolism | Inositol phosphate metabolism | X |  |
| Superpathway_of_Inositol_Phosphate_Compounds | Inositol phosphate metabolism | X |  |
| Integrin_Signaling | Integrin signalling | X |  |
| FAK_Signaling | Integrin signalling | X |  |
| ERK/MAPK_Signaling | MAPK signalling | X |  |
| LPS-stimulated_MAPK_Signaling | MAPK signalling | X |  |
| p38_MAPK_Signaling | MAPK signalling | X |  |
| SAPK/JNK_Signaling | MAPK signalling | X |  |
| PAK_Signaling | MAPK signalling | X |  |
| UVA-Induced_MAPK_Signaling | MAPK signalling | X |  |
| UVC-Induced_MAPK_Signaling | MAPK signalling |  | X |
| Melatonin_Signaling | Melatonin signalling |  | X |
| NF-κB_Activation_by_Viruses | NF-KB signalling | X |  |
| Notch_Signaling | Notch signalling |  | X |
| PTEN_Signaling | oncogenic signaling pathway?? | X |  |
| Superoxide_Radicals_Degradation | oxidative stress response | X |  |
| eNOS_Signaling | oxidative stress response |  | X |
| Thiosulfate_Disproportionation_III_(Rhodanese) | oxidative stress response |  | X |
| p53_Signaling | p53 signalling | X |  |
| Protein_Ubiquitination_Pathway | Protein Ubiquitination Pathway | X |  |
| Ubiquinol-10_Biosynthesis_(Eukaryotic) | Protein Ubiquitination Pathway | X |  |
| Relaxin_Signaling | Relaxin Signaling | X |  |
| PXR/RXR_Activation | RXR signalling |  | X |
| Ceramide_Degradation | Sphingolipid Synthesis and Metabolism | X |  |
| Agrin_Interactions_at_Neuromuscular_Junction | synaptogenesis | X |  |
| Semaphorin_Signaling_in_Neurons | synaptogenesis | X |  |
| Inhibition_of_Angiogenesis_by_TSP1 | Vascular biology | X |  |
| PEDF_Signaling | Vascular biology | X |  |
| Prostanoid_Biosynthesis | Vascular biology | X |  |
| Renin-Angiotensin_Signaling | Vascular biology | X |  |
| Role_of_NFAT_in_Cardiac_Hypertrophy | Vascular biology | X |  |
| Thrombopoietin_Signaling | Vascular biology |  | X |
| Wnt/Ca+_pathway | Wnt signalling | X |  |
| Wnt/β-catenin_Signaling | Wnt signalling | X |  |
| Cardiac_β-adrenergic_Signaling | β-adrenergic signalling | X |  |

Supplementary Table 7. Differentially expressed genes altered by graded CR relative to 12h *ad libitum* intake. First twelve genes represent those differently expressed at all CR levels.

| **Genes** | **CR10** |  | **CR20** |  | **CR30** |  | **CR40** |  |
| --- | --- | --- | --- | --- | --- | --- | --- | --- |
|  | logFC | FDR | logFC | FDR | logFC | FDR | logFC | FDR |
| *Sorl1* | 1.659 | 0.045 | 1.780 | 0.003 | 2.114 | <0.001 | 2.321 | <0.001 |
| *Gm10069* | -2.430 | 0.044 | -2.261 | 0.007 | -2.817 | <0.001 | -2.545 | <0.001 |
| *Atp1a3* | 2.397 | 0.045 | 2.720 | 0.001 | 3.632 | <0.001 | 3.847 | <0.001 |
| *Elovl6* | 3.155 | 0.004 | 3.506 | <0.001 | 4.186 | <0.001 | 4.411 | <0.001 |
| *Pth1r* | 1.420 | 0.045 | 1.406 | 0.006 | 1.982 | <0.001 | 1.888 | <0.001 |
| *Tmem79* | 1.781 | 0.022 | 1.710 | 0.003 | 2.048 | <0.001 | 2.089 | <0.001 |
| *Serpina1e* | -6.030 | 0.045 | -8.554 | <0.001 | -7.941 | <0.001 | -8.268 | <0.001 |
| *Acot3* | 2.608 | 0.045 | 3.273 | <0.001 | 3.972 | <0.001 | 4.494 | <0.001 |
| *Slc2a5* | 2.364 | 0.047 | 2.785 | 0.001 | 3.348 | <0.001 | 3.540 | <0.001 |
| *Pdk1* | 1.066 | 0.047 | 1.516 | <0.001 | 1.941 | <0.001 | 2.241 | <0.001 |
| *Oscp1* | 1.144 | 0.024 | 1.443 | <0.001 | 1.807 | <0.001 | 1.721 | <0.001 |
| *Rorc* | 1.220 | 0.034 | 1.600 | <0.001 | 2.113 | <0.001 | 2.441 | <0.001 |
| *Tgm3* | 5.542 | 0.003 | 2.793 | 0.093 | 3.822 | 0.012 | 4.567 | 0.001 |
| *Npdc1* | -0.272 | 0.970 | -0.600 | 0.012 | -0.521 | 0.026 | -0.969 | <0.001 |
| *Pcdh1* | -0.602 | 0.448 | -0.868 | 0.006 | -0.701 | 0.025 | -1.030 | <0.001 |
| *St5* | -0.281 | 0.969 | -0.496 | 0.044 | -0.603 | 0.009 | -0.772 | <0.001 |
| *Psph* | 0.637 | 0.717 | 1.040 | 0.010 | 1.255 | 0.001 | 1.285 | <0.001 |
| *Ntrk2* | -0.489 | 0.894 | -1.026 | 0.005 | -1.142 | 0.001 | -1.866 | <0.001 |
| *Nek6* | -0.382 | 0.971 | -0.712 | 0.044 | -0.729 | 0.033 | -1.009 | 0.001 |
| *Fads1* | 0.127 | 0.998 | 0.606 | 0.048 | 0.673 | 0.019 | 0.719 | 0.005 |
| *Tmem53* | 0.449 | 0.772 | 0.604 | 0.047 | 0.876 | 0.001 | 0.951 | <0.001 |
| *Usp2* | 0.845 | 0.219 | 1.063 | 0.006 | 1.300 | <0.001 | 1.511 | <0.001 |
| *Pmm1* | 0.583 | 0.738 | 0.836 | 0.029 | 1.336 | <0.001 | 1.672 | <0.001 |
| *Egfr* | -0.834 | 0.116 | -1.094 | 0.001 | -1.032 | 0.001 | -1.165 | <0.001 |
| *Gm15386* | 3.721 | 0.841 | 10.704 | 0.016 | 9.606 | 0.014 | 8.650 | 0.010 |
| *Cox4i1* | 0.451 | 0.709 | 0.702 | 0.014 | 0.865 | 0.001 | 1.019 | <0.001 |
| *Dock11* | -0.668 | 0.398 | -0.877 | 0.011 | -1.060 | 0.001 | -1.655 | <0.001 |
| *Metap1* | 0.336 | 0.954 | 0.661 | 0.017 | 0.646 | 0.014 | 1.032 | <0.001 |
| *Akr1c14* | -1.293 | 0.534 | -1.647 | 0.022 | -1.784 | 0.010 | -1.952 | 0.002 |
| *Cdh11* | -0.574 | 0.966 | -0.971 | 0.045 | -1.101 | 0.017 | -1.228 | 0.002 |
| *Pxdn* | -0.547 | 0.759 | -0.956 | 0.007 | -0.807 | 0.020 | -0.885 | 0.004 |
| *Nid2* | -0.422 | 0.970 | -0.778 | 0.041 | -0.759 | 0.039 | -1.363 | <0.001 |
| *Magi1* | -0.389 | 0.764 | -0.527 | 0.044 | -0.601 | 0.016 | -0.781 | <0.001 |
| *Gbe1* | 0.945 | 0.453 | 1.170 | 0.022 | 1.542 | 0.001 | 1.931 | <0.001 |
| *Itpripl2* | -0.407 | 0.819 | -0.648 | 0.024 | -0.660 | 0.016 | -0.740 | 0.002 |
| *Slc9a2* | 2.704 | 0.229 | 3.156 | 0.013 | 2.921 | 0.014 | 2.318 | 0.031 |
| *Gcnt4* | 0.755 | 0.998 | 4.579 | 0.020 | 3.594 | 0.047 | 3.213 | 0.044 |
| *Plce1* | -0.508 | 0.756 | -0.673 | 0.045 | -0.860 | 0.006 | -1.479 | <0.001 |
| *Bcl6b* | -0.773 | 0.408 | -1.160 | 0.003 | -1.157 | 0.002 | -1.337 | <0.001 |
| *Rassf6* | 0.953 | 0.673 | 1.578 | 0.006 | 2.096 | <0.001 | 2.410 | <0.001 |
| *Zeb2* | -0.452 | 0.819 | -0.757 | 0.017 | -0.921 | 0.002 | -1.169 | <0.001 |
| *Cst11* | 2.678 | 0.998 | 9.661 | 0.028 | 8.812 | 0.026 | 8.041 | 0.019 |
| *Acss2* | 1.471 | 0.247 | 1.511 | 0.032 | 2.036 | 0.001 | 2.126 | <0.001 |
| *Akr1c6* | -2.895 | 0.998 | -7.326 | 0.040 | -8.185 | 0.023 | -6.277 | 0.039 |
| *Tecr* | 0.874 | 0.181 | 1.195 | 0.001 | 1.503 | <0.001 | 1.465 | <0.001 |
| *Lmo2* | -0.132 | 0.998 | -0.779 | 0.008 | -0.842 | 0.002 | -1.165 | <0.001 |
| *Pla2g12a* | 0.430 | 0.894 | 0.700 | 0.033 | 0.888 | 0.003 | 1.116 | <0.001 |
| *Il2rg* | -0.208 | 0.998 | -0.847 | 0.036 | -0.919 | 0.017 | -1.174 | 0.001 |
| *Defb25* | 2.359 | 0.998 | 9.419 | 0.016 | 8.494 | 0.015 | 7.840 | 0.010 |
| *Gclm* | 0.517 | 0.760 | 0.884 | 0.010 | 0.671 | 0.046 | 0.841 | 0.004 |
| *Tfpi* | -0.368 | 0.933 | -0.624 | 0.035 | -0.664 | 0.019 | -0.836 | 0.001 |
| *Plxna1* | -0.254 | 0.976 | -0.538 | 0.022 | -0.502 | 0.027 | -0.586 | 0.003 |
| *Thbd* | -0.584 | 0.970 | -1.182 | 0.021 | -1.030 | 0.040 | -1.095 | 0.013 |
| *Fbn1* | -0.595 | 0.970 | -1.253 | 0.018 | -1.361 | 0.007 | -1.912 | <0.001 |
| *Spink2* | 0.424 | 0.998 | 6.843 | 0.012 | 5.989 | 0.015 | 4.364 | 0.037 |
| *Adamts16* | -1.519 | 0.479 | -1.942 | 0.020 | -1.949 | 0.016 | -2.272 | 0.002 |
| *Apob* | -3.010 | 0.969 | -6.294 | 0.035 | -8.561 | 0.006 | -6.264 | 0.017 |
| *Ccdc18* | 0.753 | 0.998 | 1.516 | 0.045 | 1.496 | 0.039 | 1.575 | 0.013 |
| *Npr3* | -0.638 | 0.969 | -1.416 | 0.010 | -1.822 | <0.001 | -2.876 | <0.001 |
| *Ascl4* | 2.171 | 0.970 | 7.222 | 0.015 | 5.810 | 0.024 | 4.878 | 0.025 |
| *Stard13* | -0.498 | 0.305 | -0.739 | 0.001 | -0.750 | 0.001 | -0.773 | <0.001 |
| *Crispld2* | -0.933 | 0.386 | -1.089 | 0.022 | -1.027 | 0.026 | -1.548 | <0.001 |
| *Bank1* | -0.791 | 0.607 | -1.107 | 0.015 | -1.702 | <0.001 | -2.407 | <0.001 |
| *Gjc1* | -0.539 | 0.617 | -0.654 | 0.038 | -0.759 | 0.011 | -1.033 | <0.001 |
| *Sash1* | -0.495 | 0.663 | -0.740 | 0.012 | -0.616 | 0.032 | -0.637 | 0.012 |
| *Gng11* | -0.436 | 0.717 | -0.684 | 0.013 | -0.661 | 0.013 | -0.850 | <0.001 |
| *Plekha2* | -0.302 | 0.833 | -0.538 | 0.012 | -0.566 | 0.005 | -0.854 | <0.001 |
| *Cpt1a* | -0.221 | 0.989 | -0.473 | 0.033 | -0.561 | 0.007 | -0.880 | <0.001 |
| *Col18a1* | -0.492 | 0.734 | -0.948 | 0.002 | -1.022 | <0.001 | -1.583 | <0.001 |
| *Sipa1* | -0.599 | 0.458 | -0.926 | 0.003 | -0.816 | 0.008 | -1.219 | <0.001 |
| *Asgr2* | -1.592 | 0.663 | -2.527 | 0.010 | -2.413 | 0.010 | -2.730 | 0.001 |
| *Haao* | -1.649 | 0.617 | -2.698 | 0.006 | -2.771 | 0.003 | -3.331 | <0.001 |
| *Hpd* | -2.399 | 0.333 | -4.207 | <0.001 | -3.280 | 0.005 | -2.504 | 0.016 |
| *Pcsk4* | 1.188 | 0.300 | 1.349 | 0.020 | 1.946 | <0.001 | 1.832 | <0.001 |
| *Cst8* | 1.430 | 0.998 | 8.640 | 0.028 | 7.601 | 0.030 | 7.166 | 0.020 |
| *Fam198b* | -0.397 | 0.871 | -0.722 | 0.013 | -0.672 | 0.016 | -0.638 | 0.011 |
| *Boc* | -0.363 | 0.849 | -0.589 | 0.025 | -0.645 | 0.010 | -0.849 | <0.001 |
| *Ndn* | -0.331 | 0.987 | -0.662 | 0.038 | -0.655 | 0.034 | -1.307 | <0.001 |
| *Cdh2* | -0.784 | 0.617 | -0.983 | 0.033 | -0.985 | 0.026 | -1.532 | <0.001 |
| *Acot4* | 1.107 | 0.210 | 1.356 | 0.007 | 1.396 | 0.002 | 1.569 | <0.001 |
| *Nr1d1* | -0.412 | 0.987 | -1.186 | 0.001 | -1.024 | 0.005 | -0.860 | 0.010 |
| *Rnf146* | 0.720 | 0.674 | 0.995 | 0.024 | 1.385 | <0.001 | 1.777 | <0.001 |
| *Nsdhl* | 0.745 | 0.658 | 1.091 | 0.015 | 1.427 | <0.001 | 1.559 | <0.001 |
| *Cacnb4* | 0.418 | 0.987 | 0.804 | 0.043 | 0.890 | 0.017 | 0.756 | 0.027 |
| *Hhex* | -0.444 | 0.967 | -0.911 | 0.015 | -1.247 | <0.001 | -1.371 | <0.001 |
| *Pzp* | -3.734 | 0.759 | -6.016 | 0.023 | -7.887 | 0.004 | -6.661 | 0.005 |
| *Psmd5* | 0.311 | 0.930 | 0.514 | 0.039 | 0.703 | 0.002 | 0.584 | 0.005 |
| *Fbln5* | -0.492 | 0.658 | -0.972 | <0.001 | -0.860 | 0.001 | -1.392 | <0.001 |
| *Itih3* | -2.277 | 0.328 | -3.385 | 0.003 | -3.209 | 0.003 | -2.956 | 0.003 |
| *A430105I19Rik* | -0.247 | 0.998 | -0.711 | 0.038 | -0.940 | 0.003 | -1.504 | <0.001 |
| *Defb48* | 1.719 | 0.998 | 8.645 | 0.021 | 7.573 | 0.024 | 6.521 | 0.023 |
| *Cpne2* | -0.503 | 0.759 | -0.902 | 0.006 | -1.011 | 0.001 | -1.361 | <0.001 |
| *Fdft1* | 0.594 | 0.168 | 0.818 | 0.001 | 1.101 | <0.001 | 1.245 | <0.001 |
| *Acacb* | 1.127 | 0.617 | 1.639 | 0.014 | 2.171 | <0.001 | 2.521 | <0.001 |
| *Vgll3* | -0.858 | 0.116 | -1.127 | 0.001 | -1.674 | <0.001 | -1.997 | <0.001 |
| *Arhgef2* | -0.364 | 0.769 | -0.525 | 0.032 | -0.520 | 0.027 | -0.683 | 0.001 |
| *Hoxc4* | -0.415 | 0.822 | -0.799 | 0.006 | -0.600 | 0.038 | -0.949 | <0.001 |
| *Prkcdbp* | -0.476 | 0.700 | -1.040 | <0.001 | -1.138 | <0.001 | -1.777 | <0.001 |
| *Mpc1* | 1.307 | 0.157 | 1.540 | 0.006 | 2.024 | <0.001 | 2.278 | <0.001 |
| *D730048I06Rik* | 2.368 | 0.998 | 9.225 | 0.027 | 8.633 | 0.023 | 6.729 | 0.033 |
| *Fam26e* | -0.583 | 0.828 | -1.044 | 0.013 | -0.917 | 0.026 | -1.007 | 0.006 |
| *Kng1* | -2.683 | 0.953 | -5.774 | 0.018 | -7.732 | 0.002 | -5.601 | 0.008 |
| *9430020K01Rik* | -0.325 | 0.945 | -0.520 | 0.050 | -0.615 | 0.014 | -0.643 | 0.004 |
| *Smoc2* | -0.799 | 0.302 | -0.863 | 0.026 | -1.005 | 0.006 | -1.602 | <0.001 |
| *Acot1* | 1.030 | 0.709 | 1.699 | 0.008 | 1.535 | 0.012 | 2.420 | <0.001 |
| *Fabp5* | 1.206 | 0.403 | 1.279 | 0.047 | 1.608 | 0.007 | 1.577 | 0.003 |
| *Cdnf* | 0.479 | 0.894 | 0.894 | 0.013 | 0.842 | 0.014 | 0.882 | 0.004 |
| *Serpina1c* | -3.967 | 0.690 | -7.334 | 0.008 | -8.103 | 0.003 | -7.226 | 0.003 |
| *Tmem179* | 1.397 | 0.181 | 1.513 | 0.016 | 2.069 | <0.001 | 1.989 | <0.001 |
| *Ros1* | 2.569 | 0.998 | 7.980 | 0.029 | 7.187 | 0.029 | 7.143 | 0.014 |
| *Rnase13* | 1.665 | 0.998 | 8.261 | 0.033 | 7.556 | 0.032 | 6.241 | 0.036 |
| *Heg1* | -0.463 | 0.966 | -0.799 | 0.041 | -0.771 | 0.042 | -0.948 | 0.004 |
| *Slc2a13* | -0.677 | 0.709 | -0.978 | 0.021 | -1.348 | 0.001 | -2.018 | <0.001 |
| *Grb14* | -1.080 | 0.185 | -1.639 | <0.001 | -1.948 | <0.001 | -3.268 | <0.001 |
| *Slc27a1* | 0.893 | 0.668 | 1.386 | 0.011 | 1.981 | <0.001 | 2.285 | <0.001 |
| *Esyt1* | -0.301 | 0.998 | -0.655 | 0.044 | -0.664 | 0.034 | -0.982 | <0.001 |
| *Slc25a16* | 0.343 | 0.734 | 0.464 | 0.038 | 0.602 | 0.003 | 0.720 | <0.001 |
| *Man1c1* | -0.211 | 0.998 | -0.710 | 0.045 | -0.769 | 0.023 | -1.056 | <0.001 |
| *Ecm2* | -0.478 | 0.781 | -0.727 | 0.023 | -0.659 | 0.035 | -0.966 | <0.001 |
| *Klhl13* | -0.467 | 0.793 | -0.857 | 0.007 | -0.870 | 0.004 | -1.006 | <0.001 |
| *Ankrd27* | -0.322 | 0.623 | -0.433 | 0.022 | -0.457 | 0.011 | -0.381 | 0.020 |
| *Abat* | -0.339 | 0.987 | -0.828 | 0.010 | -0.770 | 0.013 | -1.030 | <0.001 |
| *Sbk1* | 1.049 | 0.168 | 1.226 | 0.007 | 1.567 | <0.001 | 1.309 | 0.001 |
| *Tuba1a* | -0.769 | 0.354 | -1.322 | <0.001 | -1.393 | <0.001 | -2.040 | <0.001 |
| *Col8a1* | -0.606 | 0.982 | -1.132 | 0.048 | -1.388 | 0.010 | -2.028 | <0.001 |
| *Gm17727* | 1.671 | 0.998 | 8.941 | 0.036 | 8.229 | 0.031 | 6.034 | 0.044 |
| *Antxr2* | -0.375 | 0.953 | -0.787 | 0.010 | -0.729 | 0.013 | -1.042 | <0.001 |
| *Palm* | -0.261 | 0.998 | -0.761 | 0.015 | -0.794 | 0.007 | -1.490 | <0.001 |
| *Gm13476* | -0.578 | 0.658 | -0.726 | 0.036 | -0.909 | 0.005 | -0.800 | 0.007 |
| *Ppa2* | 0.241 | 0.970 | 0.462 | 0.033 | 0.495 | 0.015 | 0.625 | <0.001 |
| *Pdha1* | 0.915 | 0.326 | 1.197 | 0.008 | 1.567 | <0.001 | 1.846 | <0.001 |
| *Esr1* | 0.434 | 0.905 | 0.845 | 0.012 | 0.712 | 0.029 | 1.070 | <0.001 |
| *Glul* | 0.619 | 0.684 | 0.977 | 0.011 | 1.326 | <0.001 | 1.647 | <0.001 |
| *Srxn1* | 0.401 | 0.794 | 0.692 | 0.012 | 0.676 | 0.009 | 0.596 | 0.011 |
| *Arhgap23* | -0.532 | 0.415 | -0.860 | 0.001 | -0.792 | 0.002 | -0.868 | <0.001 |
| *Rbms3* | -0.481 | 0.398 | -0.594 | 0.017 | -0.779 | 0.001 | -1.061 | <0.001 |
| *L2hgdh* | 0.505 | 0.527 | 0.603 | 0.033 | 0.752 | 0.003 | 0.848 | <0.001 |
| *Unc79* | -1.065 | 0.878 | -1.647 | 0.046 | -1.604 | 0.047 | -1.939 | 0.007 |
| *Xrra1* | 0.461 | 0.998 | 2.181 | 0.007 | 1.769 | 0.025 | 2.647 | <0.001 |
| *Fn1* | -0.506 | 0.998 | -1.097 | 0.040 | -1.275 | 0.011 | -1.890 | <0.001 |
| *Scd3* | 0.538 | 0.998 | 1.403 | 0.029 | 1.545 | 0.009 | 2.321 | <0.001 |
| *Gm5177* | 0.682 | 0.658 | 0.824 | 0.046 | 1.020 | 0.008 | 1.194 | <0.001 |
| *AI182371* | -2.568 | 0.160 | -3.024 | 0.006 | -3.175 | 0.003 | -2.637 | 0.005 |
| *Syngr1* | 1.149 | 0.061 | 1.000 | 0.022 | 1.128 | 0.005 | 1.275 | <0.001 |
| *C4b* | -0.465 | 0.990 | -0.933 | 0.047 | -1.097 | 0.014 | -1.445 | <0.001 |
| *Sncg* | -0.895 | 0.969 | -1.896 | 0.015 | -2.462 | 0.001 | -4.438 | <0.001 |
| *Vegfc* | -0.561 | 0.794 | -0.937 | 0.016 | -0.911 | 0.015 | -1.402 | <0.001 |
| *Sqle* | 0.896 | 0.623 | 1.146 | 0.032 | 1.451 | 0.003 | 1.945 | <0.001 |
| *2410066E13Rik* | -0.594 | 0.667 | -0.746 | 0.039 | -1.088 | 0.001 | -1.407 | <0.001 |
| *Sh3pxd2a* | -0.967 | 0.074 | -1.410 | <0.001 | -1.405 | <0.001 | -1.390 | <0.001 |
| *Col6a2* | -0.622 | 0.804 | -1.004 | 0.020 | -0.937 | 0.026 | -1.548 | <0.001 |
| *Nxnl1* | 1.236 | 0.398 | 1.533 | 0.018 | 1.748 | 0.003 | 1.881 | <0.001 |
| *Ugt3a2* | -2.026 | 0.617 | -3.297 | 0.006 | -4.031 | 0.001 | -3.423 | 0.001 |
| *Hsd17b13* | -2.715 | 0.789 | -4.486 | 0.021 | -7.515 | <0.001 | -4.780 | 0.005 |
| *Pdhx* | 0.580 | 0.825 | 0.841 | 0.046 | 1.209 | 0.001 | 1.423 | <0.001 |
| *Aldh1a7* | 0.782 | 0.600 | 1.135 | 0.012 | 1.436 | <0.001 | 1.410 | <0.001 |
| *Ldlrad3* | 0.493 | 0.392 | 0.817 | 0.001 | 1.013 | <0.001 | 1.020 | <0.001 |
| *Fgfr1* | -0.419 | 0.793 | -0.754 | 0.007 | -0.659 | 0.016 | -0.928 | <0.001 |
| *Il1rap* | -0.526 | 0.804 | -0.780 | 0.035 | -0.907 | 0.010 | -0.784 | 0.014 |
| *Slc1a2* | -1.770 | 0.658 | -2.112 | 0.047 | -2.727 | 0.009 | -2.762 | 0.003 |
| *Cox5a* | 0.490 | 0.617 | 0.594 | 0.039 | 0.946 | <0.001 | 1.034 | <0.001 |
| *Rtn4rl1* | -0.297 | 0.998 | -0.912 | 0.010 | -1.108 | 0.001 | -1.574 | <0.001 |
| *Rhbdd2* | 0.281 | 0.947 | 0.460 | 0.045 | 0.541 | 0.011 | 0.700 | <0.001 |
| *Mxra8* | -0.480 | 0.849 | -0.839 | 0.015 | -0.858 | 0.009 | -1.196 | <0.001 |
| *Map4* | -0.364 | 0.833 | -0.622 | 0.016 | -0.540 | 0.033 | -0.682 | 0.002 |
| *F10* | -1.906 | 0.247 | -2.527 | 0.004 | -2.227 | 0.009 | -1.948 | 0.011 |
| *St3gal6* | 0.522 | 0.987 | 1.330 | 0.009 | 1.799 | <0.001 | 2.026 | <0.001 |
| *Cox6c* | 0.298 | 0.998 | 0.705 | 0.021 | 0.875 | 0.002 | 0.952 | <0.001 |
| *Gsta4* | 0.531 | 0.600 | 0.619 | 0.045 | 0.708 | 0.014 | 0.556 | 0.037 |
| *Elk3* | -0.346 | 0.936 | -0.700 | 0.011 | -0.590 | 0.028 | -0.868 | <0.001 |
| *Arrb1* | -0.279 | 0.998 | -0.693 | 0.022 | -0.801 | 0.005 | -1.116 | <0.001 |
| *Ass1* | -2.200 | 0.142 | -2.651 | 0.003 | -2.916 | 0.001 | -2.758 | <0.001 |
| *Uox* | -2.567 | 0.833 | -4.062 | 0.036 | -5.400 | 0.006 | -5.229 | 0.003 |
| *Pcdh12* | -0.841 | 0.424 | -1.425 | 0.001 | -1.100 | 0.010 | -1.975 | <0.001 |
| *1700047G03Rik* | -0.780 | 0.953 | -1.578 | 0.015 | -2.425 | <0.001 | -3.599 | <0.001 |
| *Jup* | -0.375 | 0.930 | -0.713 | 0.015 | -0.679 | 0.016 | -1.056 | <0.001 |
| *Slmo1* | 0.913 | 0.894 | 1.531 | 0.028 | 1.475 | 0.026 | 1.535 | 0.008 |
| *Serpina1a* | -2.972 | 0.487 | -5.864 | <0.001 | -5.963 | <0.001 | -5.566 | <0.001 |
| *Gde1* | 0.655 | 0.333 | 0.710 | 0.033 | 1.276 | <0.001 | 1.524 | <0.001 |
| *Aacs* | 0.955 | 0.344 | 1.000 | 0.042 | 1.578 | <0.001 | 1.477 | <0.001 |
| *Lfng* | -0.245 | 0.998 | -0.779 | 0.018 | -0.677 | 0.035 | -1.139 | <0.001 |
| *Spock1* | 2.406 | 0.987 | 5.872 | 0.037 | 5.192 | 0.043 | 4.972 | 0.028 |
| *Vtn* | -1.582 | 0.089 | -2.098 | <0.001 | -1.523 | 0.009 | -1.925 | <0.001 |
| *Lcn10* | 2.338 | 0.998 | 8.886 | 0.027 | 8.168 | 0.025 | 7.857 | 0.014 |
| *Tat* | -2.719 | 0.864 | -4.504 | 0.034 | -6.004 | 0.006 | -3.743 | 0.041 |
| *Ms4a1* | -1.707 | 0.108 | -1.719 | 0.011 | -2.026 | 0.002 | -1.739 | 0.003 |
| *Ovch2* | 4.075 | 0.828 | 10.880 | 0.008 | 9.590 | 0.008 | 9.330 | 0.004 |
| *Gm4846* | 2.278 | 0.998 | 9.600 | 0.015 | 8.108 | 0.019 | 5.868 | 0.041 |
| *9230104L09Rik* | 2.098 | 0.998 | 9.039 | 0.010 | 8.022 | 0.010 | 7.480 | 0.006 |
| *Muc5b* | 0.648 | 0.998 | 7.365 | 0.028 | 6.492 | 0.032 | 5.187 | 0.044 |
| *Lyplal1* | 0.349 | 0.988 | 0.834 | 0.015 | 0.789 | 0.015 | 0.600 | 0.047 |
| *Mpc2* | 0.953 | 0.285 | 1.012 | 0.029 | 1.507 | <0.001 | 1.615 | <0.001 |
| *Serpinf1* | -0.730 | 0.663 | -0.954 | 0.030 | -0.938 | 0.026 | -1.619 | <0.001 |
| *Cnksr2* | 0.915 | 0.998 | 6.197 | 0.022 | 5.506 | 0.026 | 5.377 | 0.014 |
| *Amotl2* | -0.531 | 0.697 | -0.812 | 0.013 | -0.850 | 0.006 | -1.194 | <0.001 |
| *Pprc1* | -0.458 | 0.759 | -0.753 | 0.013 | -0.634 | 0.032 | -0.661 | 0.012 |
| *Spag11b* | 2.321 | 0.998 | 8.909 | 0.019 | 8.543 | 0.013 | 8.420 | 0.006 |
| *Ephb2* | -0.342 | 0.998 | -0.858 | 0.018 | -0.976 | 0.004 | -1.463 | <0.001 |
| *Pvr* | -0.512 | 0.225 | -0.496 | 0.036 | -0.542 | 0.016 | -0.649 | 0.001 |
| *Tgfb1i1* | -0.245 | 0.998 | -0.575 | 0.041 | -0.580 | 0.032 | -0.753 | 0.001 |
| *Il18r1* | -0.305 | 0.998 | -1.024 | 0.045 | -0.994 | 0.046 | -1.274 | 0.003 |
| *Trpv2* | -0.745 | 0.403 | -1.170 | 0.002 | -1.060 | 0.004 | -1.275 | <0.001 |
| *Sc4mol* | 0.439 | 0.967 | 0.927 | 0.012 | 1.040 | 0.002 | 1.087 | <0.001 |
| *Hpx* | -2.795 | 0.760 | -5.116 | 0.010 | -6.654 | 0.001 | -5.067 | 0.003 |
| *Disp2* | -1.187 | 0.397 | -1.460 | 0.017 | -2.370 | <0.001 | -3.770 | <0.001 |
| *Atp6v1g1* | 0.247 | 0.998 | 0.755 | 0.046 | 0.726 | 0.047 | 0.650 | 0.048 |
| *Arxes2* | 0.617 | 0.894 | 1.016 | 0.033 | 1.368 | 0.001 | 1.379 | <0.001 |
| *Sepn1* | -0.293 | 0.994 | -0.709 | 0.016 | -0.648 | 0.024 | -0.983 | <0.001 |
| *Slc10a1* | -2.416 | 0.663 | -4.494 | 0.003 | -5.448 | <0.001 | -4.317 | 0.001 |
| *Bhmt* | -3.024 | 0.781 | -4.473 | 0.037 | -6.836 | 0.002 | -5.061 | 0.008 |
| *F5* | -0.999 | 0.683 | -1.334 | 0.031 | -1.366 | 0.022 | -1.231 | 0.021 |
| *Prelp* | -0.405 | 0.998 | -1.095 | 0.023 | -1.553 | <0.001 | -2.521 | <0.001 |
| *Myo1c* | -0.392 | 0.966 | -0.754 | 0.020 | -0.716 | 0.023 | -0.938 | 0.001 |
| *Gpd2* | 0.886 | 0.527 | 1.178 | 0.018 | 1.631 | <0.001 | 1.819 | <0.001 |
| *Cyp2c44* | -3.056 | 0.181 | -4.371 | 0.001 | -3.630 | 0.005 | -3.770 | 0.001 |
| *Itgbl1* | -1.205 | 0.356 | -1.269 | 0.038 | -1.386 | 0.018 | -2.083 | <0.001 |
| *Tdo2* | -2.249 | 0.285 | -3.379 | 0.001 | -3.323 | 0.001 | -2.719 | 0.003 |
| *Echdc1* | 0.847 | 0.617 | 1.117 | 0.025 | 1.527 | 0.001 | 1.927 | <0.001 |
| *Akap12* | -0.544 | 0.420 | -0.802 | 0.004 | -1.017 | <0.001 | -0.963 | <0.001 |
| *Sec16b* | -0.376 | 0.970 | -0.757 | 0.022 | -0.706 | 0.029 | -0.899 | 0.001 |
| *9230110F15Rik* | 2.653 | 0.998 | 9.762 | 0.017 | 8.902 | 0.015 | 8.321 | 0.009 |
| *Rps6ka3* | -0.645 | 0.168 | -0.728 | 0.009 | -0.933 | <0.001 | -0.959 | <0.001 |
| *Acvrl1* | -0.367 | 0.894 | -0.750 | 0.006 | -0.647 | 0.015 | -1.090 | <0.001 |
| *Dera* | 0.274 | 0.998 | 0.647 | 0.028 | 0.775 | 0.004 | 0.797 | 0.001 |
| *Cyp2d9* | -3.741 | 0.436 | -4.159 | 0.038 | -5.237 | 0.010 | -6.024 | 0.001 |
| *Srpx2* | -0.766 | 0.449 | -1.201 | 0.003 | -1.372 | <0.001 | -1.878 | <0.001 |
| *Acat2* | 1.177 | 0.142 | 1.574 | 0.001 | 2.006 | <0.001 | 2.007 | <0.001 |
| *Tkt* | 1.494 | 0.144 | 1.723 | 0.007 | 2.204 | <0.001 | 2.196 | <0.001 |
| *Egln3* | 0.881 | 0.323 | 1.078 | 0.013 | 1.590 | <0.001 | 1.811 | <0.001 |
| *S1pr3* | -0.313 | 0.998 | -0.757 | 0.033 | -0.729 | 0.035 | -1.322 | <0.001 |
| *Atox1* | 0.257 | 0.976 | 0.539 | 0.022 | 0.753 | <0.001 | 0.693 | <0.001 |
| *Apoa2* | -2.668 | 0.953 | -6.200 | 0.012 | -7.829 | 0.002 | -5.269 | 0.012 |
| *Map3k15* | 0.990 | 0.139 | 1.250 | 0.002 | 1.611 | <0.001 | 1.915 | <0.001 |
| *Clip2* | -0.339 | 0.910 | -0.581 | 0.030 | -0.550 | 0.034 | -1.063 | <0.001 |
| *Fndc3b* | -0.430 | 0.663 | -0.563 | 0.030 | -0.736 | 0.002 | -1.059 | <0.001 |
| *Lcat* | -2.107 | 0.326 | -2.788 | 0.008 | -3.167 | 0.002 | -2.490 | 0.006 |
| *Acaca* | 1.416 | 0.326 | 1.629 | 0.022 | 2.019 | 0.002 | 2.015 | 0.001 |
| *Cisd1* | 0.578 | 0.617 | 0.858 | 0.011 | 1.223 | <0.001 | 1.260 | <0.001 |
| *Cadps2* | 0.746 | 0.759 | 1.465 | 0.002 | 1.636 | <0.001 | 2.445 | <0.001 |
| *Bace2* | -0.191 | 0.998 | -0.682 | 0.042 | -0.786 | 0.014 | -0.984 | <0.001 |
| *Serpina1d* | -2.923 | 0.894 | -6.318 | 0.012 | -7.516 | 0.003 | -6.190 | 0.005 |
| *Dbn1* | -0.560 | 0.706 | -0.754 | 0.033 | -0.730 | 0.032 | -1.342 | <0.001 |
| *Ly6g5c* | 1.281 | 0.998 | 8.303 | 0.033 | 7.645 | 0.030 | 6.352 | 0.034 |
| *2010003K11Rik* | 1.203 | 0.398 | 1.305 | 0.042 | 1.799 | 0.002 | 1.970 | <0.001 |
| *Smo* | -0.184 | 0.998 | -0.418 | 0.040 | -0.415 | 0.035 | -0.494 | 0.004 |
| *Mat1a* | -2.088 | 0.998 | -5.737 | 0.030 | -6.759 | 0.011 | -4.904 | 0.030 |
| *Wdfy4* | -0.508 | 0.953 | -0.852 | 0.044 | -1.030 | 0.010 | -1.738 | <0.001 |
| *Col6a5* | -0.576 | 0.969 | -1.119 | 0.026 | -1.247 | 0.009 | -2.011 | <0.001 |
| *Arhgap20* | -0.452 | 0.953 | -0.753 | 0.045 | -0.768 | 0.034 | -0.648 | 0.049 |
| *Hs3st3b1* | 0.258 | 0.998 | 2.399 | 0.021 | 2.092 | 0.034 | 2.313 | 0.008 |
| *Pmvk* | 0.801 | 0.683 | 1.006 | 0.046 | 1.603 | <0.001 | 1.829 | <0.001 |
| *Ttc25* | 1.797 | 0.108 | 1.998 | 0.006 | 2.107 | 0.002 | 2.514 | <0.001 |
| *Sypl* | 0.386 | 0.683 | 0.479 | 0.048 | 0.627 | 0.005 | 0.803 | <0.001 |
| *Acot13* | 0.488 | 0.484 | 0.673 | 0.011 | 0.615 | 0.015 | 0.487 | 0.037 |
| *Cd79a* | -1.208 | 0.142 | -1.413 | 0.005 | -1.574 | 0.001 | -1.751 | <0.001 |
| *Ubxn2a* | 0.155 | 0.998 | 0.499 | 0.013 | 0.468 | 0.015 | 0.775 | <0.001 |
| *Daxx* | -0.433 | 0.326 | -0.539 | 0.011 | -0.509 | 0.013 | -0.540 | 0.003 |
| *Defb47* | 2.903 | 0.987 | 9.185 | 0.020 | 8.762 | 0.015 | 8.806 | 0.006 |
| *Slc9a6* | 0.344 | 0.987 | 0.777 | 0.020 | 0.670 | 0.039 | 1.132 | <0.001 |
| *Hmgcs2* | -0.506 | 0.970 | -1.190 | 0.010 | -1.145 | 0.009 | -0.971 | 0.015 |
| *Col15a1* | -0.492 | 0.953 | -1.027 | 0.010 | -1.253 | 0.001 | -2.021 | <0.001 |
| *Vkorc1l1* | 0.275 | 0.967 | 0.464 | 0.048 | 0.466 | 0.039 | 0.542 | 0.006 |
| *Tmem120b* | 1.054 | 0.398 | 1.197 | 0.032 | 1.697 | 0.001 | 1.773 | <0.001 |
| *Vasp* | -0.226 | 0.998 | -0.545 | 0.038 | -0.526 | 0.039 | -0.592 | 0.008 |
| *Nrg4* | 0.855 | 0.617 | 1.104 | 0.029 | 1.412 | 0.002 | 1.620 | <0.001 |
| *Lrrc27* | 0.955 | 0.074 | 1.066 | 0.003 | 1.364 | <0.001 | 1.378 | <0.001 |
| *Mgl2* | -0.566 | 0.889 | -0.976 | 0.022 | -1.041 | 0.010 | -1.490 | <0.001 |
| *Wdr81* | -0.136 | 0.998 | -0.522 | 0.031 | -0.534 | 0.021 | -0.613 | 0.003 |
| *Mcam* | -0.488 | 0.889 | -0.871 | 0.016 | -1.018 | 0.003 | -1.522 | <0.001 |
| *Pkp2* | -0.442 | 0.755 | -0.718 | 0.012 | -0.935 | <0.001 | -1.150 | <0.001 |
| *Hspa12a* | -0.277 | 0.998 | -0.792 | 0.037 | -0.909 | 0.011 | -1.614 | <0.001 |
| *Gm4788* | -1.676 | 0.326 | -2.428 | 0.003 | -2.272 | 0.004 | -2.367 | 0.001 |
| *Dlat* | 0.911 | 0.415 | 1.149 | 0.018 | 1.519 | <0.001 | 1.769 | <0.001 |
| *Adam28* | 1.357 | 0.998 | 7.643 | 0.008 | 6.507 | 0.010 | 5.384 | 0.014 |
| *Kirrel* | -0.387 | 0.953 | -0.910 | 0.003 | -0.995 | <0.001 | -1.192 | <0.001 |
| *H2-Ab1* | -0.250 | 0.998 | -0.758 | 0.048 | -0.912 | 0.011 | -1.560 | <0.001 |
| *Cdc42ep1* | -0.447 | 0.960 | -1.020 | 0.005 | -0.894 | 0.011 | -1.284 | <0.001 |
| *Mgst2* | 1.171 | 0.347 | 1.489 | 0.012 | 1.984 | <0.001 | 2.139 | <0.001 |
| *Gnmt* | -2.984 | 0.415 | -3.806 | 0.017 | -4.446 | 0.005 | -3.844 | 0.005 |
| *2310061I04Rik* | 0.447 | 0.695 | 0.608 | 0.030 | 0.821 | 0.001 | 0.860 | <0.001 |
| *Ddx58* | -0.270 | 0.933 | -0.441 | 0.044 | -0.539 | 0.008 | -0.663 | <0.001 |
| *Lrtm1* | 1.016 | 0.369 | 1.175 | 0.024 | 1.888 | <0.001 | 2.016 | <0.001 |
| *Igsf10* | -0.339 | 0.976 | -0.641 | 0.042 | -0.752 | 0.011 | -0.964 | <0.001 |
| *Atp6v1d* | 0.172 | 0.998 | 0.496 | 0.042 | 0.496 | 0.033 | 0.649 | 0.001 |
| *Itih2* | -2.497 | 0.238 | -3.506 | 0.002 | -3.953 | <0.001 | -4.125 | <0.001 |
| *Chchd10* | 1.275 | 0.285 | 1.651 | 0.007 | 2.024 | <0.001 | 2.532 | <0.001 |
| *Alb* | -2.461 | 0.722 | -5.174 | 0.002 | -6.467 | <0.001 | -5.500 | <0.001 |
| *Pdhb* | 0.862 | 0.617 | 1.117 | 0.029 | 1.641 | <0.001 | 1.875 | <0.001 |
| *Nmnat3* | 0.396 | 0.970 | 0.839 | 0.019 | 1.266 | <0.001 | 1.362 | <0.001 |
| *Pah* | -2.848 | 0.858 | -5.688 | 0.013 | -6.783 | 0.003 | -4.494 | 0.020 |
| *C2cd2l* | 0.633 | 0.789 | 0.900 | 0.041 | 1.192 | 0.003 | 1.416 | <0.001 |
| *Hpca* | -1.021 | 0.796 | -1.571 | 0.030 | -1.730 | 0.013 | -1.960 | 0.002 |
| *Mxra7* | -0.368 | 0.986 | -0.702 | 0.045 | -0.678 | 0.047 | -1.040 | <0.001 |
| *Ubl3* | 0.321 | 0.735 | 0.482 | 0.020 | 0.483 | 0.014 | 0.567 | 0.001 |
| *Ppap2b* | -0.321 | 0.970 | -0.661 | 0.023 | -0.662 | 0.018 | -0.874 | <0.001 |
| *Gpx5* | 2.805 | 0.998 | 9.455 | 0.027 | 9.049 | 0.020 | 8.611 | 0.012 |
| *Nov* | -1.048 | 0.155 | -1.624 | <0.001 | -1.083 | 0.010 | -1.573 | <0.001 |
| *Dchs1* | -0.467 | 0.967 | -1.060 | 0.008 | -0.912 | 0.019 | -1.149 | 0.001 |
| *Tiam1* | -0.404 | 0.970 | -0.785 | 0.033 | -1.132 | 0.001 | -1.477 | <0.001 |
| *Prtn3* | -0.880 | 0.808 | -1.429 | 0.023 | -1.566 | 0.010 | -1.668 | 0.002 |
| *Ecscr* | -0.384 | 0.875 | -0.678 | 0.018 | -0.591 | 0.034 | -0.845 | <0.001 |
| *Ambp* | -2.802 | 0.970 | -6.498 | 0.028 | -8.493 | 0.006 | -5.349 | 0.032 |
| *Rdh7* | -2.625 | 0.987 | -5.654 | 0.047 | -7.250 | 0.014 | -5.347 | 0.032 |
| *Slc7a2* | -0.860 | 0.398 | -1.086 | 0.015 | -1.033 | 0.016 | -0.994 | 0.010 |
| *Marcks* | -0.400 | 0.932 | -0.781 | 0.013 | -0.959 | 0.001 | -1.461 | <0.001 |
| *Pdzk1ip1* | 1.574 | 0.804 | 2.775 | 0.016 | 2.423 | 0.025 | 1.999 | 0.039 |
| *Rasgrf2* | -0.427 | 0.827 | -0.805 | 0.007 | -0.959 | 0.001 | -1.505 | <0.001 |
| *Defb20* | 2.260 | 0.998 | 9.058 | 0.024 | 8.463 | 0.021 | 7.717 | 0.015 |
| *Tenm4* | -0.788 | 0.440 | -1.002 | 0.016 | -1.379 | <0.001 | -2.023 | <0.001 |
| *Azgp1* | -1.395 | 0.998 | -4.345 | 0.030 | -5.007 | 0.012 | -3.525 | 0.040 |
| *Cnr1* | -0.622 | 0.717 | -0.912 | 0.022 | -1.033 | 0.006 | -1.313 | <0.001 |
| *Cercam* | -0.245 | 0.998 | -0.765 | 0.046 | -0.803 | 0.030 | -1.133 | <0.001 |
| *Cyp51* | 1.313 | 0.247 | 1.778 | 0.004 | 2.523 | <0.001 | 3.702 | <0.001 |
| *Myo1d* | -0.328 | 0.849 | -0.579 | 0.015 | -0.561 | 0.014 | -0.949 | <0.001 |
| *Vasn* | -0.763 | 0.086 | -1.003 | <0.001 | -1.035 | <0.001 | -1.181 | <0.001 |
| *2310044G17Rik* | 0.452 | 0.225 | 0.465 | 0.026 | 0.599 | 0.001 | 0.765 | <0.001 |
| *Tril* | -0.496 | 0.683 | -0.689 | 0.024 | -0.657 | 0.026 | -0.925 | <0.001 |
| *C230081A13Rik* | -0.398 | 0.759 | -0.662 | 0.011 | -0.712 | 0.003 | -0.877 | <0.001 |
| *Ces2g* | -0.146 | 0.998 | -0.771 | 0.015 | -0.919 | 0.002 | -1.068 | <0.001 |
| *Wee1* | 0.666 | 0.246 | 0.939 | 0.001 | 0.790 | 0.007 | 0.763 | 0.003 |
| *Cry1* | 0.520 | 0.759 | 0.733 | 0.033 | 1.148 | <0.001 | 1.295 | <0.001 |
| *Teddm1* | 1.798 | 0.998 | 8.750 | 0.022 | 7.836 | 0.023 | 7.185 | 0.017 |
| *5033411D12Rik* | 0.771 | 0.755 | 1.014 | 0.048 | 1.637 | <0.001 | 1.440 | 0.001 |
| *Tpmt* | 0.248 | 0.998 | 0.958 | 0.035 | 0.906 | 0.037 | 0.949 | 0.014 |
| *Slc38a3* | -2.156 | 0.663 | -2.779 | 0.033 | -2.749 | 0.032 | -2.277 | 0.046 |
| *Dusp10* | -0.936 | 0.285 | -1.260 | 0.004 | -1.347 | 0.001 | -1.484 | <0.001 |
| *Setd1b* | -0.251 | 0.970 | -0.456 | 0.047 | -0.547 | 0.012 | -0.402 | 0.046 |
| *Tgfb3* | -0.649 | 0.789 | -1.040 | 0.018 | -1.125 | 0.007 | -1.445 | <0.001 |
| *Bcl2l15* | 0.642 | 0.998 | 5.756 | 0.022 | 5.124 | 0.026 | 4.057 | 0.042 |
| *Daam1* | -0.247 | 0.970 | -0.649 | 0.003 | -0.696 | 0.001 | -0.824 | <0.001 |
| *Apln* | -0.731 | 0.822 | -1.349 | 0.010 | -1.059 | 0.038 | -1.628 | <0.001 |
| *Lcn5* | 1.444 | 0.998 | 8.594 | 0.026 | 8.020 | 0.023 | 6.479 | 0.030 |
| *Snx33* | -0.501 | 0.440 | -0.896 | <0.001 | -0.973 | <0.001 | -0.928 | <0.001 |
| *D430019H16Rik* | -0.751 | 0.998 | -1.854 | 0.031 | -2.443 | 0.003 | -4.074 | <0.001 |
| *Chrnb2* | 0.809 | 0.763 | 1.111 | 0.039 | 1.555 | 0.001 | 1.900 | <0.001 |
| *Adam15* | -0.276 | 0.987 | -0.605 | 0.023 | -0.551 | 0.034 | -0.920 | <0.001 |
| *Rarres2* | -0.734 | 0.078 | -1.264 | <0.001 | -1.378 | <0.001 | -1.603 | <0.001 |
| *Csf1* | -0.561 | 0.683 | -0.910 | 0.007 | -0.803 | 0.015 | -1.212 | <0.001 |
| *Insig1* | 1.402 | 0.060 | 1.690 | 0.001 | 1.762 | <0.001 | 1.557 | <0.001 |
| *Cyp4v3* | -0.527 | 0.733 | -0.899 | 0.007 | -0.962 | 0.002 | -1.573 | <0.001 |
| *Pon1* | -1.552 | 0.671 | -2.644 | 0.005 | -3.023 | 0.001 | -2.580 | 0.002 |
| *Arl2* | 0.247 | 0.998 | 0.503 | 0.049 | 0.758 | 0.001 | 0.614 | 0.004 |
| *Rnase10* | 2.276 | 0.998 | 8.749 | 0.007 | 7.805 | 0.006 | 7.157 | 0.004 |
| *Ntn4* | -0.310 | 0.987 | -0.704 | 0.016 | -0.635 | 0.026 | -0.894 | <0.001 |
| *Ebf1* | -0.512 | 0.685 | -0.751 | 0.017 | -0.751 | 0.013 | -0.805 | 0.003 |
| *Htra1* | -0.153 | 0.998 | -0.678 | 0.024 | -0.590 | 0.046 | -1.034 | <0.001 |
| *Tmem147* | 0.405 | 0.828 | 0.628 | 0.031 | 0.784 | 0.003 | 0.831 | <0.001 |
| *Emp1* | -0.564 | 0.953 | -1.258 | 0.006 | -1.435 | 0.001 | -2.104 | <0.001 |
| *2900026A02Rik* | -0.451 | 0.658 | -0.587 | 0.030 | -0.565 | 0.030 | -0.616 | 0.007 |
| *Mzt1* | 0.446 | 0.822 | 0.795 | 0.012 | 0.665 | 0.029 | 0.636 | 0.020 |
| *Fam43a* | -0.376 | 0.804 | -0.617 | 0.018 | -0.568 | 0.025 | -0.691 | 0.002 |
| *Ahsg* | -2.804 | 0.398 | -5.214 | <0.001 | -6.078 | <0.001 | -5.188 | <0.001 |
| *Dock8* | -0.303 | 0.998 | -0.785 | 0.012 | -1.035 | <0.001 | -1.002 | <0.001 |
| *Plxnd1* | -0.416 | 0.953 | -1.009 | 0.002 | -0.864 | 0.007 | -1.240 | <0.001 |
| *Gm7120* | 0.893 | 0.326 | 0.971 | 0.030 | 0.867 | 0.046 | 1.368 | <0.001 |
| *Lyrm5* | 0.273 | 0.987 | 0.530 | 0.044 | 0.593 | 0.016 | 0.554 | 0.012 |
| *Mvd* | 0.975 | 0.412 | 1.046 | 0.045 | 1.500 | 0.001 | 1.780 | <0.001 |
| *Frmd6* | -0.263 | 0.987 | -0.581 | 0.021 | -0.563 | 0.021 | -0.812 | <0.001 |
| *Ankrd44* | -0.230 | 0.998 | -0.577 | 0.046 | -0.613 | 0.027 | -0.736 | 0.002 |
| *Ccdc57* | 0.721 | 0.642 | 1.093 | 0.010 | 1.048 | 0.008 | 1.375 | <0.001 |
| *Cml2* | -3.019 | 0.825 | -6.321 | 0.009 | -6.362 | 0.007 | -5.636 | 0.006 |
| *Gnb4* | -0.669 | 0.181 | -1.111 | <0.001 | -0.996 | <0.001 | -1.391 | <0.001 |
| *Sema4c* | -0.338 | 0.936 | -0.557 | 0.044 | -0.563 | 0.035 | -0.680 | 0.004 |
| *Apoh* | -2.593 | 0.970 | -5.849 | 0.030 | -7.485 | 0.007 | -5.360 | 0.021 |
| *Lamb2* | -0.397 | 0.889 | -0.691 | 0.019 | -0.656 | 0.022 | -1.020 | <0.001 |
| *Me1* | 1.840 | 0.170 | 2.032 | 0.013 | 2.489 | 0.001 | 2.834 | <0.001 |
| *Col6a6* | -0.535 | 0.988 | -1.086 | 0.044 | -1.080 | 0.039 | -1.841 | <0.001 |
| *Mup3* | -3.442 | 0.479 | -5.975 | 0.003 | -5.740 | 0.003 | -5.946 | 0.001 |
| *Lrrc61* | -0.536 | 0.210 | -0.974 | <0.001 | -1.048 | <0.001 | -1.274 | <0.001 |
| *Adcy10* | 2.119 | 0.108 | 2.347 | 0.006 | 2.720 | <0.001 | 3.077 | <0.001 |
| *Slc44a2* | -0.505 | 0.709 | -0.802 | 0.011 | -0.685 | 0.026 | -1.045 | <0.001 |
| *Dusp3* | -0.250 | 0.987 | -0.518 | 0.036 | -0.538 | 0.023 | -0.690 | 0.001 |
| *Adamtsl4* | -0.255 | 0.998 | -0.731 | 0.045 | -0.751 | 0.033 | -0.799 | 0.010 |
| *C7* | -0.942 | 0.617 | -1.171 | 0.032 | -1.355 | 0.009 | -2.234 | <0.001 |
| *Elovl4* | 4.175 | 0.159 | 3.914 | 0.033 | 3.569 | 0.036 | 3.637 | 0.016 |
| *Gm6792* | 1.602 | 0.998 | 8.292 | 0.010 | 7.587 | 0.008 | 6.383 | 0.010 |
| *Lcn9* | 2.496 | 0.998 | 9.414 | 0.033 | 8.717 | 0.029 | 8.076 | 0.020 |
| *Cryba4* | 1.008 | 0.998 | 7.067 | 0.030 | 6.394 | 0.030 | 6.054 | 0.019 |
| *Lasp1* | -0.420 | 0.683 | -0.702 | 0.006 | -0.766 | 0.001 | -1.093 | <0.001 |
| *Ifi27l2a* | -1.011 | 0.247 | -2.075 | <0.001 | -2.308 | <0.001 | -3.265 | <0.001 |
| *Ldhd* | 0.627 | 0.998 | 1.926 | 0.007 | 1.698 | 0.011 | 1.465 | 0.016 |
| *Chchd4* | 0.328 | 0.987 | 0.640 | 0.042 | 0.701 | 0.017 | 0.738 | 0.005 |
| *Ptk2b* | -0.241 | 0.970 | -0.452 | 0.037 | -0.529 | 0.009 | -0.805 | <0.001 |
| *Apoe* | -0.462 | 0.953 | -0.939 | 0.012 | -0.886 | 0.014 | -0.739 | 0.025 |
| *Ddo* | 0.906 | 0.246 | 1.216 | 0.003 | 1.264 | 0.001 | 1.478 | <0.001 |
| *Ltbp3* | -0.286 | 0.970 | -0.562 | 0.028 | -0.498 | 0.046 | -0.681 | 0.001 |
| *Ly6g5b* | 2.062 | 0.998 | 8.169 | 0.008 | 7.281 | 0.008 | 6.798 | 0.005 |
| *Mup20* | -5.555 | 0.709 | -7.914 | 0.037 | -11.005 | 0.007 | -12.786 | 0.001 |
| *Acly* | 2.066 | 0.076 | 2.302 | 0.004 | 2.901 | <0.001 | 3.264 | <0.001 |
| *Prkce* | 0.963 | 0.155 | 1.036 | 0.012 | 1.064 | 0.006 | 0.720 | 0.046 |
| *Cox7c* | 0.339 | 0.953 | 0.649 | 0.019 | 0.891 | <0.001 | 0.871 | <0.001 |
| *Eln* | -0.737 | 0.759 | -1.237 | 0.011 | -1.170 | 0.012 | -1.229 | 0.003 |
| *Serpina1b* | -2.774 | 0.875 | -5.276 | 0.018 | -7.628 | 0.001 | -6.203 | 0.002 |
| *Akap2* | -0.445 | 0.804 | -0.680 | 0.029 | -0.634 | 0.035 | -0.884 | 0.001 |
| *Daam2* | -0.807 | 0.108 | -0.946 | 0.003 | -0.953 | 0.001 | -1.216 | <0.001 |
| *Ndufb9* | 0.457 | 0.683 | 0.651 | 0.021 | 1.024 | <0.001 | 1.091 | <0.001 |
| *Il27ra* | -0.424 | 0.998 | -1.027 | 0.029 | -1.441 | 0.001 | -1.397 | 0.001 |
| *Abcg4* | -0.207 | 0.998 | -1.242 | 0.032 | -1.258 | 0.026 | -1.137 | 0.024 |
| *Map1s* | -0.331 | 0.970 | -0.604 | 0.047 | -0.623 | 0.034 | -0.876 | 0.001 |
| *Slc6a13* | -0.535 | 0.970 | -0.961 | 0.048 | -1.740 | <0.001 | -2.963 | <0.001 |
| *Supt3* | 0.400 | 0.894 | 0.757 | 0.012 | 0.937 | 0.001 | 0.883 | <0.001 |
| *Anxa1* | -0.368 | 0.965 | -0.712 | 0.020 | -0.630 | 0.034 | -0.939 | <0.001 |
| *Slc38a5* | 0.050 | 0.998 | 5.756 | 0.021 | 5.590 | 0.015 | 3.871 | 0.049 |
| *Dusp18* | 0.478 | 0.659 | 0.803 | 0.003 | 0.823 | 0.001 | 1.127 | <0.001 |
| *Lss* | 1.474 | 0.218 | 1.441 | 0.035 | 2.197 | <0.001 | 2.476 | <0.001 |
| *Rhoc* | -0.209 | 0.998 | -0.535 | 0.024 | -0.519 | 0.024 | -0.821 | <0.001 |
| *Tm7sf2* | 0.885 | 0.759 | 1.420 | 0.016 | 1.629 | 0.002 | 1.452 | 0.003 |
| *Arhgef10* | -0.175 | 0.998 | -0.545 | 0.035 | -0.769 | 0.001 | -0.816 | <0.001 |
| *Sfrp5* | -0.861 | 0.998 | -2.958 | 0.003 | -3.855 | <0.001 | -4.506 | <0.001 |
| *Prr12* | -0.338 | 0.894 | -0.523 | 0.043 | -0.505 | 0.044 | -0.552 | 0.012 |
| *Gls2* | -2.341 | 0.108 | -2.392 | 0.010 | -2.326 | 0.009 | -2.597 | 0.001 |
| *Spink12* | 4.581 | 0.886 | 8.868 | 0.043 | 8.366 | 0.036 | 6.986 | 0.039 |
| *Lbp* | -0.471 | 0.760 | -0.700 | 0.024 | -0.924 | 0.001 | -1.343 | <0.001 |
| *Recql4* | 1.845 | 0.108 | 1.708 | 0.022 | 2.344 | <0.001 | 2.888 | <0.001 |
| *Trp63* | 1.010 | 0.769 | 1.793 | 0.008 | 1.743 | 0.006 | 2.036 | <0.001 |
| *Angptl3* | -1.847 | 0.302 | -2.633 | 0.003 | -1.976 | 0.023 | -2.139 | 0.005 |
| *Mpzl2* | 1.200 | 0.139 | 1.979 | <0.001 | 2.159 | <0.001 | 2.834 | <0.001 |
| *Cst12* | 2.996 | 0.998 | 9.557 | 0.037 | 8.675 | 0.035 | 8.406 | 0.020 |
| *Bag1* | 0.544 | 0.397 | 0.684 | 0.015 | 1.065 | <0.001 | 1.242 | <0.001 |
| *Arhgap18* | -0.206 | 0.998 | -0.485 | 0.042 | -0.700 | 0.001 | -0.770 | <0.001 |
| *Cd248* | -0.260 | 0.998 | -0.942 | 0.030 | -0.977 | 0.018 | -1.615 | <0.001 |
| *Grhpr* | 0.415 | 0.767 | 0.589 | 0.035 | 0.835 | 0.001 | 0.569 | 0.017 |
| *Eri2* | 0.440 | 0.969 | 0.781 | 0.044 | 1.297 | <0.001 | 1.975 | <0.001 |
| *Lgals9* | -0.408 | 0.885 | -0.685 | 0.024 | -0.673 | 0.022 | -0.852 | 0.001 |
| *Ogn* | -0.720 | 0.387 | -0.911 | 0.013 | -1.018 | 0.003 | -1.377 | <0.001 |
| *Rdh11* | 1.006 | 0.478 | 1.215 | 0.029 | 1.913 | <0.001 | 1.898 | <0.001 |
| *Rad50* | -0.424 | 0.302 | -0.483 | 0.018 | -0.652 | <0.001 | -0.869 | <0.001 |
| *4930471I20Rik* | 1.798 | 0.108 | 1.818 | 0.013 | 2.319 | <0.001 | 2.378 | <0.001 |
| *Spink5* | 3.347 | 0.894 | 7.170 | 0.022 | 6.787 | 0.017 | 5.533 | 0.024 |
| *Rbm28* | -0.301 | 0.900 | -0.476 | 0.042 | -0.597 | 0.006 | -0.575 | 0.003 |
| *Ephx3* | -0.614 | 0.894 | -1.091 | 0.022 | -1.355 | 0.003 | -1.880 | <0.001 |
| *Ppp1r18* | -0.286 | 0.998 | -0.615 | 0.045 | -0.622 | 0.035 | -0.958 | <0.001 |
| *Adi1* | 0.326 | 0.933 | 0.522 | 0.046 | 0.646 | 0.008 | 0.592 | 0.007 |
| *Rhcg* | 1.893 | 0.998 | 8.041 | 0.021 | 7.634 | 0.016 | 6.609 | 0.016 |
| *Mrpl30* | 0.469 | 0.499 | 0.667 | 0.010 | 0.806 | <0.001 | 0.976 | <0.001 |
| *Map1a* | -0.297 | 0.998 | -0.849 | 0.018 | -0.864 | 0.012 | -1.012 | 0.001 |
| *Nudt12* | 0.531 | 0.837 | 0.807 | 0.037 | 0.826 | 0.025 | 1.023 | 0.001 |
| *Acsl1* | 0.813 | 0.759 | 1.232 | 0.024 | 1.297 | 0.011 | 1.295 | 0.005 |
| *Heyl* | -0.497 | 0.717 | -0.763 | 0.016 | -0.841 | 0.005 | -0.895 | 0.001 |
| *Parm1* | 1.363 | 0.163 | 1.582 | 0.008 | 2.091 | <0.001 | 1.968 | <0.001 |
| *Rara* | -0.342 | 0.967 | -0.595 | 0.044 | -0.600 | 0.035 | -0.728 | 0.003 |
| *C130026I21Rik* | -0.563 | 0.998 | -1.525 | 0.016 | -2.389 | <0.001 | -2.053 | <0.001 |
| *Zfp532* | -0.255 | 0.981 | -0.545 | 0.022 | -0.557 | 0.015 | -0.713 | <0.001 |
| *Gm1110* | 1.677 | 0.998 | 8.106 | 0.010 | 7.568 | 0.007 | 6.396 | 0.008 |
| *Ndufb6* | 0.575 | 0.455 | 0.649 | 0.039 | 0.937 | 0.001 | 0.859 | 0.001 |
| *Adora1* | 0.897 | 0.247 | 1.066 | 0.011 | 1.262 | 0.001 | 0.992 | 0.005 |
| *Cyp2j5* | -3.334 | 0.889 | -5.934 | 0.032 | -8.923 | 0.002 | -4.958 | 0.036 |
| *Gramd4* | -0.391 | 0.709 | -0.655 | 0.007 | -0.817 | <0.001 | -1.141 | <0.001 |
| *Svip* | 0.396 | 0.894 | 0.597 | 0.050 | 0.709 | 0.012 | 0.654 | 0.011 |
| *Gm7609* | -0.392 | 0.998 | -0.995 | 0.046 | -1.091 | 0.023 | -1.634 | <0.001 |
| *Ndufa10* | 0.480 | 0.709 | 0.639 | 0.038 | 0.962 | <0.001 | 1.011 | <0.001 |
| *Col6a3* | -0.432 | 0.987 | -0.874 | 0.034 | -0.871 | 0.029 | -1.685 | <0.001 |
| *Trim16* | -0.105 | 0.998 | -0.758 | 0.030 | -0.845 | 0.010 | -0.854 | 0.004 |
| *Unc5a* | 0.856 | 0.534 | 1.106 | 0.022 | 1.505 | <0.001 | 1.301 | 0.001 |
| *Hc* | -3.315 | 0.181 | -5.121 | <0.001 | -5.321 | <0.001 | -4.471 | <0.001 |
| *Pcdh19* | -0.394 | 0.967 | -0.734 | 0.029 | -0.734 | 0.023 | -0.934 | 0.001 |
| *Proz* | -1.775 | 0.623 | -2.576 | 0.015 | -2.461 | 0.016 | -2.758 | 0.003 |
| *Acot2* | 0.549 | 0.607 | 0.654 | 0.041 | 0.801 | 0.007 | 0.835 | 0.001 |
| *Chac1* | 0.909 | 0.293 | 1.030 | 0.019 | 1.212 | 0.002 | 1.181 | 0.001 |
| *Ido1* | -0.222 | 0.998 | 6.112 | 0.020 | 5.422 | 0.024 | 4.118 | 0.046 |
| *Slmo2* | 0.410 | 0.828 | 0.601 | 0.044 | 0.806 | 0.003 | 1.061 | <0.001 |
| *Maml2* | -0.404 | 0.746 | -0.629 | 0.016 | -0.702 | 0.004 | -0.720 | 0.001 |
| *Itgb5* | -0.317 | 0.979 | -0.676 | 0.021 | -0.645 | 0.023 | -0.998 | <0.001 |
| *Fam150b* | -1.139 | 0.181 | -1.693 | <0.001 | -1.324 | 0.005 | -1.410 | 0.001 |
| *Zfp648* | 1.924 | 0.998 | 6.387 | 0.037 | 5.957 | 0.034 | 5.976 | 0.016 |
| *Adamts5* | -0.930 | 0.181 | -1.502 | <0.001 | -1.667 | <0.001 | -2.227 | <0.001 |
| *Nes* | -0.697 | 0.479 | -0.833 | 0.029 | -0.858 | 0.018 | -0.798 | 0.015 |
| *Slc25a1* | 1.439 | 0.157 | 1.604 | 0.010 | 2.266 | <0.001 | 2.382 | <0.001 |
| *Tfr2* | -2.390 | 0.157 | -2.474 | 0.015 | -2.445 | 0.013 | -3.926 | <0.001 |
| *Icam2* | -0.379 | 0.970 | -0.810 | 0.020 | -0.766 | 0.023 | -0.969 | 0.001 |
| *Tmem50b* | 0.321 | 0.881 | 0.587 | 0.014 | 0.601 | 0.007 | 0.428 | 0.041 |
| *Thrsp* | 1.302 | 0.305 | 1.549 | 0.016 | 2.123 | <0.001 | 2.285 | <0.001 |
| *Anxa5* | -0.364 | 0.789 | -0.626 | 0.011 | -0.683 | 0.003 | -1.009 | <0.001 |
| *Mybph* | 0.923 | 0.305 | 1.061 | 0.019 | 1.215 | 0.003 | 1.142 | 0.002 |
| *Lcn8* | 2.806 | 0.998 | 9.805 | 0.022 | 8.751 | 0.023 | 8.101 | 0.016 |
| *Serpinc1* | -2.949 | 0.594 | -5.203 | 0.003 | -6.351 | <0.001 | -4.905 | 0.001 |
| *Taok3* | -0.265 | 0.998 | -0.657 | 0.016 | -0.693 | 0.008 | -0.763 | 0.001 |
| *Aldob* | -2.088 | 0.967 | -3.701 | 0.047 | -4.254 | 0.022 | -3.421 | 0.035 |
| *Tnrc18* | -0.370 | 0.769 | -0.610 | 0.013 | -0.721 | 0.001 | -0.737 | <0.001 |
| *Fbxo44* | 0.564 | 0.793 | 0.799 | 0.041 | 1.069 | 0.002 | 1.170 | <0.001 |
| *Sccpdh* | 0.369 | 0.902 | 0.606 | 0.035 | 0.776 | 0.003 | 0.959 | <0.001 |
| *Lipg* | -0.027 | 0.998 | 3.293 | 0.015 | 2.830 | 0.025 | 2.795 | 0.013 |
| *1110059M19Rik* | -1.773 | 0.142 | -2.562 | <0.001 | -3.248 | <0.001 | -5.378 | <0.001 |
| *Ppp1r3d* | -0.613 | 0.594 | -0.755 | 0.033 | -0.827 | 0.014 | -1.162 | <0.001 |
| *2700081O15Rik* | -0.300 | 0.970 | -0.568 | 0.038 | -0.676 | 0.009 | -0.926 | <0.001 |
| *Syp* | -0.995 | 0.157 | -1.488 | <0.001 | -1.728 | <0.001 | -2.211 | <0.001 |
| *Spire1* | 0.442 | 0.796 | 0.627 | 0.042 | 0.838 | 0.003 | 1.371 | <0.001 |
| *Lrrk1* | -0.271 | 0.988 | -0.602 | 0.024 | -0.590 | 0.023 | -0.617 | 0.007 |
| *4930506C21Rik* | 1.150 | 0.210 | 1.419 | 0.006 | 1.872 | <0.001 | 2.179 | <0.001 |
| *Mall* | -0.426 | 0.953 | -0.745 | 0.036 | -0.876 | 0.009 | -1.309 | <0.001 |
| *Meox1* | -0.640 | 0.822 | -0.964 | 0.036 | -1.091 | 0.013 | -1.700 | <0.001 |
| *Uhrf1bp1l* | 0.304 | 0.878 | 0.529 | 0.019 | 0.492 | 0.023 | 0.663 | <0.001 |
| *Cbr1* | 0.246 | 0.998 | 0.581 | 0.039 | 0.812 | 0.001 | 1.056 | <0.001 |
| *C6* | -0.753 | 0.495 | -1.237 | 0.002 | -1.175 | 0.002 | -1.486 | <0.001 |
| *Bahcc1* | -0.439 | 0.760 | -0.730 | 0.012 | -0.651 | 0.020 | -1.133 | <0.001 |
| *Fdps* | 0.895 | 0.717 | 1.344 | 0.020 | 1.652 | 0.002 | 1.812 | <0.001 |
| *Tln2* | -0.510 | 0.683 | -0.625 | 0.049 | -0.867 | 0.003 | -1.264 | <0.001 |
| *Nav1* | -0.513 | 0.709 | -0.857 | 0.007 | -0.831 | 0.006 | -1.289 | <0.001 |

Supplementary Table 8. Significantly altered pathways at all CR levels and their targets genes

| **Ingenuity Canonical Pathways** | **10CR** | **20CR** | **30CR** | **40CR** |
| --- | --- | --- | --- | --- |
| Coagulation System | SERPINA1 | KNG1, F10, SERPINC1, F5, SERPINA1, TFPI, THBD, PLAT | KNG1, PROC, THBD, FGG, F2, PLG, KLKB1, F10, SERPINC1, F8, F5, FGB, SERPINA1, PLAU, FGA, TFPI | KNG1, F2R, PROC, VWF, THBD, F3, F2, FGG, PLG, KLKB1, F10, SERPINC1, F8, PROS1, F5, SERPINA1, FGB, PLAU, FGA, SERPINE1, TFPI, PLAT |
| Stearate Biosynthesis I (Animals) | ACOT1, ELOVL6 | ACOT2, ACOT1, ACOT4, SLC27A1, ACSL1, ELOVL6 | ACSL3, ACOT2, FASN, ACOT1, ACOT4, SLC27A1, ACSL1, ELOVL6 | ACSL3, ACOT2, FAM213B, CYP2E1, PPT1, FASN, ACOT1, ACOT4, SLC27A1, ACSL1, ELOVL6 |
| Acute Phase Response Signaling | N/A | ITIH3, HPX, FN1, APOH, APOA2, AHSG, AMBP, SERPINF1, SERPINA3, C5, C4A/C4B, ALB, ITIH2, SAA1, SERPINA1, LBP, IL1RAP, AGT | ECSIT, ITIH3, FN1, APOH, APOA2, AMBP, SERPINA3, F2, FGG, C4A/C4B, SOD2, JUN, F8, ITIH2, PIK3CG, SERPINA1, FGB, LBP, IL1RAP, HPX, TTR, AHSG, SERPINF1, C5, PLG, KLKB1, ALB, APOA1, MAP2K3, FGA | MAP2K4, ITIH3, APOA2, PIK3R1, SOCS6, FGG, F2, SOD2, ITIH2, PIK3CG, FGB, SERPINE1, IL1RAP, TTR, TCF3, C5, IL33, KLKB1, IL18, PIK3CD, FGA, SOCS5, C2, ECSIT, RELA, SERPING1, FN1, APOH, NFKBIE, AMBP, NFKB1, C1R, C4A/C4B, TRADD, NFKBIA, JUN, F8, NGFR, CFB, SOCS2, SERPINA1, LBP, HPX, TNFRSF1A, AHSG, SERPINF1, VWF, IL1R1, PLG, ALB, APOA1, RBP7, PIK3CB, ELK1 |
| Extrinsic Prothrombin Activation Pathway | N/A | F10, SERPINC1, F5, TFPI, THBD | F10, SERPINC1, F5, PROC, FGB, TFPI, THBD, FGA, FGG, F2 | F10, SERPINC1, PROS1, F5, PROC, FGB, TFPI, THBD, FGA, F3, FGG, F2 |
| Cholesterol Biosynthesis III (via Desmosterol) | N/A | SQLE, FDFT1, NSDHL, LSS, TM7SF2, CYP51A1 | SQLE, FDFT1, NSDHL, LSS, TM7SF2, CYP51A1 | SQLE, FDFT1, NSDHL, DHCR7, HSD17B7, LSS, TM7SF2, SC5D, CYP51A1 |
| Tryptophan Degradation III (Eukaryotic) | N/A | ACAT2, TDO2, HAAO, IDO1 | HSD17B10, KMO, ACAT2, TDO2, HAAO, IDO1, EHHADH, HADH | HSD17B10, KMO, ACAT2, TDO2, HAAO, HADHB, IDO1, EHHADH, CA1, HADHA, HADH |
| Phospholipase C Signaling | N/A | PEBP1, RHOC, RPS6KA3, CD79A, RHOJ, ARHGEF17, PRKCG, GNB4, GNG11, PLCE1, PRKCE, ARHGEF2, PRKCH, ADCY10, ARHGEF10, PRKD3, MYL12B, PLA2G12A | MYL2, RHOC, MYLPF, CHP1, RPS6KA3, RAPGEF3, CD79A, CREB5, GNB4, GNG11, PLCE1, AHNAK, PRKCE, ARHGEF2, ADCY10, ARHGEF10, ADCY7, LCP2, MYL3, ITGA4, PLA2G12A | PLD2, MYL2, GNB5, MYL6B, GNB4, PLCE1, HDAC7, MYL3, ITGA4, PLA2G12A, ITPR2, RALB, ITGA5, ARHGEF17, CD79A, RAPGEF3, CD3G, RND3, PLA2G2D, SYK, PLCG2, PLCB3, ADCY10, ARHGEF10, LCP2, FYN, RELA, Calm1 (includes others), RPS6KA3, NFKB1, GNG7, GNG11, PPP3CB, AHNAK, RHOT1, PRKCE, ARHGEF2, ARHGEF3, MYL12B, ITGB1, HDAC9, SRC, CD79B, RHOC, MYLPF, CHP1, HDAC1, NFATC4, MYL1, PRKCG, PLD4, PLA2G4A, PLA2G2E, LYN, FCER1G, ADCY7 |
| Intrinsic Prothrombin Activation Pathway | N/A | KNG1, F10, SERPINC1, F5, THBD, COL18A1 | KNG1, PROC, THBD, FGG, F2, KLKB1, F10, SERPINC1, COL5A3, F8, F5, FGB, FGA, COL18A1 | KNG1, PROC, THBD, F2, FGG, COL1A2, KLKB1, COL1A1, F10, SERPINC1, COL5A3, F8, PROS1, F5, FGB, FGA, COL18A1, COL3A1 |
| FXR/RXR Activation | N/A | KNG1, APOE, HPX, APOB, SLC10A1, APOH, NR1H4, VTN, APOC4, APOA2, AHSG, AMBP, SERPINF1, C4A/C4B, PON1, ALB, LCAT, SAA1, RARA, SERPINA1, AGT | KNG1, APOE, APOA4, APOB, SLC10A1, APOH, APOA2, APOC4, VTN, AMBP, C4A/C4B, PON1, LCAT, RARA, FASN, SERPINA1, SLCO1B3, HPX, TTR, MLXIPL, CYP27A1, AHSG, SERPINF1, ALB, APOA1, SREBF1, G6PC, FGA | MAP2K4, KNG1, APOE, SLC10A1, APOA4, APOB, SLC4A2, APOH, APOA2, VTN, AMBP, C4A/C4B, PON1, LCAT, RARA, FASN, SERPINA1, MLXIPL, HPX, TTR, CYP27A1, AHSG, SERPINF1, IL33, IL18, ALB, APOA1, SREBF1, PLTP, FGA, PPARGC1A |
| Glioma Invasiveness Signaling | N/A | TIMP4, RHOC, VTN, RHOJ, ITGB5 | PLG, RHOC, PIK3CG, VTN, PLAU, ITGB5, MMP9 | TIMP3, F2R, RHOC, VTN, PIK3R1, MMP2, PIK3R4, PLG, TIMP4, RND3, RHOT1, PIK3CG, CD44, PIK3CB, PIK3CD, PLAU, MMP9, ITGB5, TIMP2 |
| LXR/RXR Activation | N/A | KNG1, APOE, APOB, APOH, ABCG4, NR1H4, APOA2, APOC4, VTN, AMBP, C4A/C4B, FDFT1, PON1, LYZ, LCAT, SAA1, SERPINA1, LBP, IL1RAP, CYP51A1, AGT, HPX, AHSG, SERPINF1, ALB, ACACA | KNG1, APOE, SCD, APOA4, APOB, APOH, ABCG4, APOA2, APOC4, VTN, AMBP, ABCG1, C4A/C4B, PON1, FDFT1, LYZ, LCAT, FASN, SERPINA1, LBP, IL1RAP, CYP51A1, HPX, MLXIPL, TTR, ECHS1, AHSG, SERPINF1, APOA5, ALB, APOA1, SREBF1, ACACA, FGA, MMP9, HADH | KNG1, APOE, SCD, RELA, APOA4, APOB, APOH, ABCG4, APOA2, VTN, AMBP, NFKB1, C4A/C4B, PON1, FDFT1, LCAT, CCL2, FASN, NGFR, SERPINA1, TLR3, LBP, IL1RAP, CYP51A1, HPX, TTR, MLXIPL, ECHS1, TNFRSF1A, AHSG, SERPINF1, IL1R1, IL33, ALB, IL18, APOA1, SREBF1, ACACA, PLTP, NCOR2, FGA, MMP9, HADH |
| Glutathione Redox Reactions I | N/A | MGST2, GPX1, GPX5 | GSTT2/GSTT2B, MGST2, GPX5, GPX4 | GSR, MGST1, MGST2, GPX1, GPX5, GPX4, GPX7, Gstt1 |
| Epoxysqualene Biosynthesis | N/A | SQLE, FDFT1 | SQLE, FDFT1 | SQLE, FDFT1 |
| IL-12 Signaling and Production in Macrophages | N/A | STAT6, APOE, APOB, APOC4, APOA2, PRKCG, PON1, ALB, LYZ, TGFB3, PRKCE, SERPINA1, PRKCH, PRKD3 | PON1, APOE, LYZ, ALB, APOA1, JUN, APOB, APOA4, PIK3CG, APOA2, APOC4, TGFB3, PRKCE, SERPINA1 | MAP2K4, RELA, APOE, APOA4, APOB, APOA2, PIK3R1, MAF, PIK3R4, NFKB1, PON1, JUN, TGFB1, PIK3CG, PRKCE, SERPINA1, STAT6, PRKCG, ALB, IL18, APOA1, CD40, TGFB3, PIK3CB, PIK3CD, IRF8, MST1R |
| Superpathway of Cholesterol Biosynthesis | N/A | MVD, FDPS, SQLE, FDFT1, NSDHL, PMVK, ACAT2, LSS, HMGCS2, TM7SF2, CYP51A1 | MVD, FDPS, SQLE, FDFT1, NSDHL, PMVK, ACAT2, IDI1, LSS, HMGCS2, TM7SF2, CYP51A1 | MVD, SQLE, NSDHL, PMVK, ACAT2, IDI1, HSD17B7, HMGCS2, TM7SF2, SC5D, FDPS, FDFT1, DHCR7, HADHB, LSS, HADHA, CYP51A1 |
| Breast Cancer Regulation by Stathmin1 | N/A | TUBB2A, ARHGEF17, PRKCG, GNB4, PPP1R3D, GNG11, TUBA1A, PRKCE, ARHGEF2, PRKCH, ADCY10, ARHGEF10, PRKD3 | CAMK1, CAMK1D, PPP2R5B, TUBA4A, ROCK2, ROCK1, GNB4, CCNE1, PPP1R3D, TUBA1A, GNG11, TUBA8, PIK3CG, PRKACA, PRKCE, ARHGEF2, ADCY10, ARHGEF10, ADCY7 | Calm1 (includes others), CAMK1, CAMK1D, PIK3R1, PPP2R5B, GNB5, PPP1R3A, LIMK2, PIK3R4, PRKAG1, GNG7, LIMK1, ROCK2, GNB4, PAK1, GNG11, TUBA8, PIK3CG, PRKCE, ARHGEF2, ARHGEF3, PPP1CA, TP53, ITPR2, TUBB2A, TUBG1, TUBA4A, ARHGEF17, PRKCG, GNAI2, PPP1R3D, CCNE1, TUBA1A, PPP2R3A, E2F1, PRKACA, PLCB3, PIK3CB, PIK3CD, ADCY10, ADCY7, ARHGEF10 |
| Complement System | N/A | C4A/C4B, C7, C6, C5 | C4A/C4B, CD55, C7, C1QBP, C6, C5 | SERPING1, C1QA, C1QB, C5, C4A/C4B, C1R, ITGB2, CD55, ITGAM, C7, CFB, CFH, C6, C1QBP, C8G, C2, ITGAX |
| Signaling by Rho Family GTPases | N/A | PTK2B, RHOC, RHOJ, ARHGEF17, CDH11, GNB4, CDH2, GNG11, CDC42EP1, ARHGEF2, ACTG2, ARHGEF10, MYL12B | NOX4, PTK2B, MYL2, RHOC, MYLPF, PIP4K2B, CDH11, ROCK2, ROCK1, GNB4, CDH2, JUN, GNG11, PIK3CG, GNAO1, CDC42EP1, ARHGEF2, PIP4K2A, ARHGEF10, MYL3, ACTA1, ITGA4 | MAP2K4, MYL2, PIK3R1, GNB5, PIP4K2B, MYL6B, LIMK2, PIK3R4, SLC9A1, LIMK1, ROCK2, GNB4, PAK1, CFL2, GNA15, PIK3CG, CDC42EP1, ACTA1, MYL3, ITGA4, NOX4, ITGA5, ARHGEF17, DES, PKN1, MAP3K12, CDH2, CDH5, RND3, PIP5K1C, GNAO1, PIK3CD, PARD3, ACTG1, PIP4K2A, ARHGEF10, RELA, PTK2B, IQGAP1, NFKB1, CDH11, GNG7, JUN, GNG11, RHOT1, ARHGEF2, ARHGEF3, MYL12B, ITGB1, ARPC5L, RHOC, MYLPF, ACTB, VIM, MYL1, GNAI2, WIPF1, WAS, PIK3CB, ELK1, MSN |
| Superpathway of Geranylgeranyldiphosphate Biosynthesis I (via Mevalonate) | N/A | MVD, FDPS, PMVK, ACAT2, HMGCS2 | MVD, FDPS, PMVK, ACAT2, IDI1, HMGCS2 | MVD, FDPS, PMVK, ACAT2, IDI1, COX10, HADHB, HMGCS2, HADHA |
| LPS/IL-1 Mediated Inhibition of RXR Function | N/A | GSTM1, APOE, CPT1A, SLC10A1, NR1H4, APOC4, HMGCS2, CHST15, SOD3, HS3ST3B1, MGST2, RARA, Gm4846, SLC27A1, FABP5, LBP, IL1RAP, ACSL1, CYP2C8 | ECSIT, APOE, ALDH4A1, SLC10A1, APOC4, ABCG1, HMGCS2, GSTT2/GSTT2B, JUN, CYP3A5, RARA, Gm4846, FABP1, FMO1, FABP5, LBP, IL1RAP, SLCO1B3, ACSL3, CPT1A, ALDH9A1, HS3ST3B1, SULT1E1, MGST2, SREBF1, SLC27A1, ACSL1, CYP2C8 | MAP2K4, ECSIT, ALDH4A1, APOE, SLC10A1, CHST7, HS2ST1, HMGCS2, SOD3, CHST15, JUN, UST, ALDH1A3, RARA, NGFR, Gm4846, FABP5, FMO1, CPT1C, LBP, IL1RAP, FMO4, ALDH7A1, GSTA3, MGST1, ACSL3, CPT1A, TNFRSF1A, IL1R1, ALDH9A1, GSTO1, IL33, HS3ST3B1, IL18, SULT1E1, MGST2, SREBF1, ALDH3B1, SLC27A1, PLTP, NDST1, ACSL1, PPARGC1A, MAOA |
| Zymosterol Biosynthesis | N/A | NSDHL, TM7SF2, CYP51A1 | NSDHL, TM7SF2, CYP51A1 | NSDHL, HSD17B7, TM7SF2, CYP51A1 |
| Cholesterol Biosynthesis II (via 24, 25-dihydrolanosterol) | N/A | SQLE, FDFT1, NSDHL, LSS, TM7SF2, CYP51A1 | SQLE, FDFT1, NSDHL, LSS, TM7SF2, CYP51A1 | SQLE, FDFT1, NSDHL, DHCR7, HSD17B7, LSS, TM7SF2, SC5D, CYP51A1 |
| Hepatic Fibrosis / Hepatic Stellate Cell Activation | N/A | CTGF, FN1, COL6A2, FGFR1, FLT4, VEGFC, COL8A1, COL15A1, COL6A6, COL6A1, COL6A3, CSF1, TGFB3, COL6A5, LBP, COL18A1, IL1RAP, EGFR, AGT, PDGFRB | MYH10, CCR5, FN1, LEP, COL4A5, MYL2, COL8A1, COL6A6, COL15A1, BCL2, COL5A1, LBP, COL18A1, IL1RAP, MYL3, EGFR, MYH1, COL6A2, FGFR1, VEGFC, MYH7, COL5A3, MYH2, COL6A3, CSF1, TGFB3, COL6A5, MMP9 | MYH4, MYH10, CCR5, MYH9, MYL2, SMAD3, COL8A1, MYL6B, VEGFA, TGFB1, CYP2E1, FIGF, SERPINE1, IL1RAP, MYL3, PDGFRB, TIMP2, COL4A1, FGFR1, VEGFC, MYH7, MMP2, COL6A3, CD40, TGFB3, MYH3, IL10RA, IGFBP3, COL6A5, PDGFD, IFNAR1, COL3A1, COL8A2, RELA, COL4A5, FN1, LEP, COL4A6, MYH8, COL4A2, CCL5, NFKB1, PDGFC, COL15A1, COL6A6, COL1A2, COL16A1, COL5A1, COL6A1, CCL2, IGF1, NGFR, LBP, COL18A1, MYH1, EGFR, COL5A2, COL6A2, TNFRSF1A, FLT4, SMAD7, IL1R1, MYL1, COL1A1, COL5A3, MYH2, CSF1, COL24A1, MMP9 |
| Mevalonate Pathway I | N/A | MVD, PMVK, ACAT2, HMGCS2 | MVD, PMVK, ACAT2, IDI1, HMGCS2 | MVD, PMVK, ACAT2, IDI1, HADHB, HMGCS2, HADHA |
| Atherosclerosis Signaling | N/A | PON1, APOE, LYZ, ALB, APOB, LCAT, CSF1, ABHD3, APOA2, APOC4, SERPINA1, COL18A1, PLA2G12A | APOE, APOA4, APOB, APOC4, APOA2, SELPLG, PON1, COL5A3, ALB, LYZ, APOA1, LCAT, CSF1, SERPINA1, COL18A1, MMP9, ITGA4, PLA2G12A | APOE, RELA, APOB, APOA4, APOA2, CCR2, NFKB1, PDGFC, COL1A2, PON1, LCAT, CCL2, TGFB1, TNFSF12, SERPINA1, COL18A1, PLA2G12A, ITGA4, PNPLA8, ABHD3, CCL11, F3, TNFRSF14, SELPLG, GLG1, IL33, PLA2G4A, COL1A1, ITGB2, COL5A3, ALB, IL18, APOA1, PLA2G2E, PLA2G2D, SELP, CD40, CSF1, PDGFD, MMP9, COL3A1 |
| Axonal Guidance Signaling | N/A | UNC5A, EPHB2, SEMA4C, GNB4, MICAL1, GNG11, PLCE1, ADAM28, EFNB1, SMO, PRKCE, SEMA3F, PRKD3, ADAMTS5, MYL12B, VASP, PLXNA1, EPHB4, ADAMTS1, ADAM15, TUBB2A, VEGFC, L1CAM, PLXND1, PRKCG, TUBA1A, NTRK2, LINGO1, PRKCH | ADAM17, RGS3, UNC5A, MYL2, EPHB2, ADAMTS2, SEMA4C, ROCK2, GNB4, PLCE1, GNG11, TUBA8, ADAM28, PIK3CG, SMO, PRKCE, SRGAP2, ADAMTS5, VASP, MYL3, ITGA4, PLXNA1, PLXNC1, ADAM15, SEMA5A, MYLPF, CHP1, TUBA4A, VEGFC, PLXND1, ROCK1, NTRK2, TUBA1A, FZD4, GNAO1, PRKACA, MMP9 | SLIT3, DPYSL2, RAC2, GLI2, ITSN1, PIK3R1, BRCC3, ADAMTS2, VEGFA, GNB4, PAK1, GNA15, PIK3CG, FIGF, SRGAP2, PLXNB2, MYL3, PTCH2, ITGA4, COPS5, SEMA5A, TUBB2A, ITGA5, MMP2, L1CAM, DOCK1, PLCG2, PRKACA, PIK3CD, PDGFD, NRP1, FYN, LRRC4C, ADAM17, RGS3, PLXNA3, UNC5A, FZD1, GNG7, PLCD1, EFNB2, TUBA8, IGF1, EFNA5, NGFR, PRKCE, PSMD14, RASSF5, MYL12B, BMP1, ITGB1, SEMA3G, PLXNA1, EPHB4, NRP2, MYLPF, SLIT2, MYL1, PRKCG, WIPF1, TUBA1A, KIF7, GLIS2, WAS, SEMA4G, BMP6, PFN1, MYL2, EPHB2, FZD3, UNC5B, GNB5, MYL6B, LIMK2, PIK3R4, LIMK1, ROCK2, MICAL1, PLCE1, SEMA3D, CFL2, SUFU, ADAM28, FZD2, ADAMTS5, PAPPA, FES, ADAMTS1, VEGFC, ADAM12, GNAO1, PLCB3, PDGFC, BCAR1, ROBO1, PRKAG1, SEMA4C, GNG11, GLI3, PPP3CB, EFNB1, SMO, SEMA3B, VASP, PLXNC1, ARPC5L, ADAM15, CHP1, TUBG1, TUBA4A, NFATC4, PLXND1, GNAI2, NTRK2, FZD4, EPHB3, PIK3CB, ADAM9, MMP9, WNT5A |
| Acetate Conversion to Acetyl-CoA | N/A | ACSS2, ACSL1 | ACSL3, ACSS2, ACSL1 | ACSL3, ACSS3, ACSS2, ACSL1 |
| Role of Tissue Factor in Cancer | N/A | F10, CTGF, ARRB1, PTK2B, CSF1, RPS6KA3, VEGFC, ITGB5, EGFR | F10, ARRB1, PTK2B, CSF1, PIK3CG, RPS6KA3, VEGFC, FGB, FGA, ITGB5, F2, FGG, EGFR | FYN, PTK2B, PIK3R1, RPS6KA3, LIMK2, PIK3R4, F2, FGG, LIMK1, VEGFA, PAK1, ARRB1, GNA15, CFL2, PIK3CG, FGB, FGR, ITGB5, EGFR, TP53, ITGB1, RPS6KB1, SRC, CASP3, VEGFC, F3, F10, CSF1, LYN, PIK3CB, RPS6KA4, PIK3CD, FGA |
| IL-8 Signaling | N/A | ANGPT2, PTK2B, RHOC, FLT4, VEGFC, RHOJ, PRKCG, GNB4, GNG11, PRKCE, PRKCH, PRKD3, TEK, LASP1, VASP, ITGB5, MYL12B, EGFR | NOX4, PTK2B, MYL2, RHOC, VEGFC, BCL2, ROCK2, ROCK1, GNB4, GNG11, JUN, PIK3CG, PRKCE, MMP9, VASP, ITGB5, LASP1, EGFR | MAP2K4, RELA, RAC2, ANGPT2, PLD2, PTK2B, MYL2, PIK3R1, GNB5, LIMK2, IQGAP1, NFKB1, PIK3R4, PDGFC, CCND1, GNG7, LIMK1, VEGFA, ROCK2, GNB4, GNG11, JUN, RHOT1, PIK3CG, PRKCE, FIGF, MYL12B, LASP1, VASP, ITGB5, TEK, EGFR, SRC, RPS6KB1, NOX4, RHOC, FLT4, VEGFC, MMP2, PRKCG, GNAI2, PLD4, ITGB2, ITGAM, RND3, PIK3CB, PIK3CD, MMP9, ITGAX |
| Clathrin-mediated Endocytosis Signaling | N/A | PON1, APOE, LYZ, ALB, ARRB1, APOB, EPHB2, APOA2, APOC4, VEGFC, SERPINA1, ACTG2, ITGB5 | APOE, APOA4, APOB, EPHB2, APOC4, APOA2, CHP1, FGF14, VEGFC, HIP1, F2, PON1, LYZ, ALB, ARRB1, APOA1, PIK3CG, TFRC, SERPINA1, ITGB5, ACTA1 | APOB, EPS15, F2R, EPHB2, APOA2, PIK3R1, CLTB, Ubb, SH3GLB1, PIK3R4, F2, VEGFA, PON1, PIK3CG, AMPH, FIGF, ACTA1, ITGB5, VEGFC, DNM3, ITGA5, SH3GL1, PIP5K1C, TFRC, PIK3CD, PDGFD, ACTG1, APOE, APOA4, PDGFC, ITGB7, FGF13, ARRB1, PPP3CB, IGF1, DAB2, SERPINA1, CSNK2B, ITGB1, SRC, ARPC5L, ACTB, CHP1, FGF14, HIP1, DNM1, ITGB2, CSNK2A2, ALB, APOA1, PIK3CB, FGF11, MYO1E |
| Production of Nitric Oxide and Reactive Oxygen Species in Macrophages | N/A | MAP3K15, APOE, APOB, RHOC, APOC4, APOA2, RHOJ, PRKCG, PON1, ALB, LYZ, PPP1R3D, PRKCE, SERPINA1, PRKCH, PRKD3 | MAP3K15, APOE, APOA4, APOB, RHOC, APOC4, APOA2, PPP2R5B, PON1, ALB, LYZ, PPP1R3D, JUN, APOA1, PIK3CG, PRKCE, SERPINA1 | MAP2K4, APOE, MAP3K15, RELA, APOA4, JAK1, APOB, PIK3R1, APOA2, NFKBIE, PPP2R5B, PPP1R3A, PIK3R4, NFKB1, PON1, NFKBIA, JUN, RHOT1, CYBA, NGFR, PIK3CG, PRKCE, SERPINA1, PPP1CA, PTPN6, TNFRSF1A, RHOC, NCF4, PRKCG, MAP3K12, PPP1R3D, ALB, APOA1, RND3, PPP2R3A, PLCG2, PIK3CB, PIK3CD, IRF8, JAK3, SIRPA |
| Epithelial Adherens Junction Signaling | N/A | CDH2, NOTCH3, MAGI1, TUBA1A, FGFR1, TUBB2A, JUP, ACTG2, EGFR | MYH10, SNAI2, MYL2, ACTN2, FGFR1, Actn3, TUBA4A, MYH7, CDH2, TUBA1A, MYH2, MAGI1, TUBA8, JUP, ACTA1, MYL3, MYH1, EGFR | MYH10, MYH4, MYH9, MYL2, MYH8, MYO7A, TGFBR3, Actn3, MLLT4, MYL6B, IQGAP1, TUBA8, PVRL1, JUP, VCL, ACTA1, MYL3, EGFR, MYH1, SRC, DLL1, NOTCH3, ARPC5L, ACTN2, FGFR1, ACTB, TUBB2A, TUBG1, TUBA4A, MYH7, TCF3, MYL1, CDH2, MAGI1, MYH2, TUBA1A, WAS, MYH3, ZYX, PARD3, ACTG1, PVRL2, CTNND1 |
| Tryptophan Degradation to 2-amino-3-carboxymuconate Semialdehyde | N/A | TDO2, HAAO, IDO1 | KMO, TDO2, HAAO, IDO1 | KMO, TDO2, HAAO, IDO1 |
| Cholesterol Biosynthesis I | N/A | SQLE, FDFT1, NSDHL, LSS, TM7SF2, CYP51A1 | SQLE, FDFT1, NSDHL, LSS, TM7SF2, CYP51A1 | SQLE, FDFT1, NSDHL, DHCR7, HSD17B7, LSS, TM7SF2, SC5D, CYP51A1 |
| RhoGDI Signaling | N/A | GNB4, CDH2, GNG11, RHOC, RHOJ, ARHGEF17, ARHGEF2, ACTG2, ARHGEF10, ESR1, CDH11, MYL12B | MYL2, RHOC, MYLPF, PIP4K2B, CDH11, ROCK2, ROCK1, GNB4, CDH2, GNG11, GNAO1, ARHGEF2, PIP4K2A, ARHGEF10, ESR1, ACTA1, MYL3, ITGA4 | MYL2, GNB5, PIP4K2B, LIMK2, MYL6B, CDH11, GNG7, LIMK1, ROCK2, GNB4, PAK1, GNG11, GNA15, CFL2, RHOT1, ARHGEF2, ARHGEF3, MYL12B, ACTA1, MYL3, ITGA4, ITGB1, SRC, ARHGAP6, ARPC5L, RHOC, MYLPF, ACTB, ARHGAP4, ITGA5, ARHGEF17, MYL1, ARHGDIB, GNAI2, CDH2, CDH5, RND3, PIP5K1C, GNAO1, CD44, ARHGAP1, PIP4K2A, ARHGEF10, ACTG1, ESR1, MSN |
| Thrombin Signaling | N/A | RHOC, RHOJ, PRKCG, GNB4, GNG11, PLCE1, PRKCE, ARHGEF2, PRKCH, ADCY10, PRKD3, ARHGEF10, MYL12B, EGFR | CAMK1, MYL2, CAMK1D, RHOC, MYLPF, F2, ROCK2, ROCK1, GNB4, GNG11, PLCE1, PIK3CG, GNAO1, PRKCE, ARHGEF2, ADCY10, ADCY7, ARHGEF10, MYL3, EGFR | RELA, CAMK1, F2R, CAMK1D, MYL2, PIK3R1, GNB5, MYL6B, NFKB1, PIK3R4, GNG7, F2, PLCD1, ROCK2, GNB4, GNG11, PLCE1, GNA15, RHOT1, PIK3CG, GATA6, PRKCE, ARHGEF2, ARHGEF3, MYL3, MYL12B, EGFR, SRC, RPS6KB1, ITPR2, RHOC, MYLPF, MYL1, PRKCG, GNAI2, RND3, PLCG2, GNAO1, PLCB3, PIK3CB, PIK3CD, ADCY10, ELK1, ARHGEF10, ADCY7 |
| Acetyl-CoA Biosynthesis I (Pyruvate Dehydrogenase Complex) | N/A | PDHA1, DLAT, PDHB | PDHA1, DLAT, DLD, PDHB | PDHA1, DLAT, DLD, PDHB |
| Protein Kinase A Signaling | N/A | AKAP12, PTK2B, PDE4B, PRKCG, AKAP2, GNB4, PPP1R3D, GNG11, DUSP3, PLCE1, DUSP10, TGFB3, SMO, PRKCE, DUSP18, PRKCH, ADCY10, PRKD3, VASP, MYL12B | AKAP12, FLNB, MYH10, DUSP8, TNNI2, MYL2, PTK2B, MYLK2, TIMM50, ROCK2, PTPRC, AKAP2, GNB4, GNG11, DUSP3, PLCE1, DUSP10, H1FX, SMO, PRKCE, RYR1, VASP, MYL3, PTPRG, MYLPF, CHP1, PYGL, PYGB, CREB5, TTN, ROCK1, PPP1R3D, Hist1h1e, MYH2, PYGM, PRKACA, TGFB3, DUSP18, TNNI1, ADCY10, ADCY7, DUSP16 | MYH4, MYH10, DUSP8, MYL2, PTPN23, SMAD3, MYLK2, TIMM50, GNB5, MYL6B, PPP1R3A, PTPN5, ROCK2, PTPRC, GNB4, PLCE1, DUSP3, TGFB1, PGP, PDE11A, PPP1CA, APEX1, MYL3, PTCH2, CDC25A, PTPN6, PTPRE, PTPRG, ITPR2, HIST1H3C, PTPN18, ANAPC7, PDE4B, TCF3, TTN, PYGM, DUSP9, PLCG2, TGFB3, PRKACA, PTPRS, PLCB3, ADCY10, EBI3, PTPRA, SIRPA, ANAPC2, AKAP12, FLNB, RELA, Calm1 (includes others), TNNI2, PTK2B, PTPN9, NFKBIE, NFKB1, GNG7, PRKAG1, PLCD1, AKAP2, PTPN4, GNG11, NFKBIA, PPP3CB, GLI3, PTPRJ, FLNA, NGFR, DUSP10, SMO, PRKCE, RYR1, VASP, MYL12B, PTPN7, PTPRK, ATF1, PDE9A, MYLPF, CHP1, PYGL, AKAP6, PYGB, NFATC4, MYL1, PRKCG, GNAI2, PPP1R3D, Hist1h1e, MYH2, ADD1, DUSP18, TNNI1, ELK1, ADCY7, DUSP16 |

Supplementary Table 9. Positively identified signalling genes based on DEGs altered by graded CR compared to 12h *ad libitum* intake. The D-score is the score that is used to discriminate signal peptides from non-signal peptides. Non-secretory peptides contain a low D-score and cut-off was set at 0.45.

| **Gene** | **Gene name** | **signalP** | **D-value** |
| --- | --- | --- | --- |
| *Lrtm1* | leucine-rich repeats and transmembrane domains 1 | yes | 0.722 |
| *Sorl1* | sortilin-related receptor, LDLR class A repeats-containing | yes | 0.768 |
| *Lbp* | lipopolysaccharide binding protein | yes | 0.848 |
| *Rtn4rl1* | reticulon 4 receptor-like 1 | yes | 0.716 |
| *Adam15* | a disintegrin and metallopeptidase domain 15 (metargidin) | yes | 0.67 |
| *Bace2* | beta-site APP-cleaving enzyme 2 | yes | 0.863 |
| *Kirrel* | kin of IRRE like (Drosophila) | yes | 0.716 |
| *Pvr* | poliovirus receptor | yes | 0.734 |
| *Prelp* | proline arginine-rich end leucine-rich repeat | yes | 0.939 |
| *Slc27a1* | solute carrier family 27 (fatty acid transporter), member 1 | yes | 0.483 |
| *Taok3* | TAO kinase 3 | yes | 0.727 |
| *Npdc1* | neural proliferation, differentiation and control 1 | yes | 0.815 |
| *Lfng* | LFNG O-fucosylpeptide 3-beta-N-acetylglucosaminyltransferase | yes | 0.836 |
| *Ces2g* | carboxylesterase 2G | yes | 0.876 |
| *Rarres2* | retinoic acid receptor responder (tazarotene induced) 2 | yes | 0.833 |
| *Ddo* | D-aspartate oxidase | yes | 0.573 |
| *H2-Ab1* | histocompatibility 2, class II antigen A, beta 1 | yes | 0.771 |
| *Rdh11* | retinol dehydrogenase 11 | yes | 0.686 |
| *Cd248* | CD248 antigen, endosialin | yes | 0.877 |
| *Parm1* | prostate androgen-regulated mucin-like protein 1 | yes | 0.691 |
| *Cercam* | cerebral endothelial cell adhesion molecule | yes | 0.823 |
| *Smo* | smoothened, frizzled class receptor | yes | 0.787 |
| *Mcam* | melanoma cell adhesion molecule | yes | 0.797 |
| *Adamts5* | a disintegrin-like and metallopeptidase (reprolysin type) with thrombospondin type 1 motif, 5 (aggrecanase-2) | yes | 0.926 |
| *Col18a1* | collagen, type XVIII, alpha 1 | yes | 0.891 |
| *Acvrl1* | activin A receptor, type II-like 1 | yes | 0.651 |
| *Pcdh19* | protocadherin 19 | yes | 0.923 |
| *Pcsk4* | proprotein convertase subtilisin/kexin type 4 | yes | 0.87 |
| *Vasn* | vasorin | yes | 0.45 |
| *Vegfc* | vascular endothelial growth factor C | yes | 0.816 |
| *Fbln5* | fibulin 5 | yes | 0.84 |
| *Itgb5* | integrin beta 5 | yes | 0.773 |
| *Npr3* | natriuretic peptide receptor 3 | yes | 0.743 |
| *Unc5a* | unc-5 homolog A (C. elegans) | yes | 0.843 |
| *Htra1* | HtrA serine peptidase 1 | yes | 0.947 |
| *Nid2* | nidogen 2 | yes | 0.862 |
| *Fgfr1* | fibroblast growth factor receptor 1 | yes | 0.912 |
| *Il2rg* | interleukin 2 receptor, gamma chain | yes | 0.755 |
| *C4b* | complement component 4B (Chido blood group) | yes | 0.7 |
| *Csf1* | colony stimulating factor 1 (macrophage) | yes | 0.788 |
| *Mxra8* | matrix-remodelling associated 8 | yes | 0.733 |
| *Sema4c* | sema domain, immunoglobulin domain (Ig), transmembrane domain (TM) and short cytoplasmic domain, (semaphorin) 4C | yes | 0.564 |
| *Col15a1* | collagen, type XV, alpha 1 | yes | 0.773 |
| *Sfrp5* | secreted frizzled-related sequence protein 5 | yes | 0.774 |
| *Antxr2* | anthrax toxin receptor 2 | yes | 0.595 |
| *Fbn1* | fibrillin 1 | yes | 0.632 |
| *Mpzl2* | myelin protein zero-like 2 | yes | 0.802 |
| *Col6a2* | collagen, type VI, alpha 2 | yes | 0.772 |
| *Plxna1* | plexin A1 | yes | 0.852 |
| *Mxra7* | matrix-remodelling associated 7 | yes | 0.551 |
| *Ldlrad3* | low density lipoprotein receptor class A domain containing 3 | yes | 0.785 |
| *Tril* | TLR4 interactor with leucine-rich repeats | yes | 0.749 |
| *Adamtsl4* | ADAMTS-like 4 | yes | 0.838 |
| *Tfpi* | tissue factor pathway inhibitor | yes | 0.763 |
| *Ephb2* | Eph receptor B2 | yes | 0.765 |
| *Plxnd1* | plexin D1 | yes | 0.514 |
| *Il18r1* | interleukin 18 receptor 1 | yes | 0.492 |
| *Pth1r* | parathyroid hormone 1 receptor | yes | 0.777 |
| *Igsf10* | immunoglobulin superfamily, member 10 | yes | 0.639 |
| *Ltbp3* | latent transforming growth factor beta binding protein 3 | yes | 0.822 |
| *Chrnb2* | cholinergic receptor, nicotinic, beta polypeptide 2 (neuronal) | yes | 0.917 |
| *Apoe* | apolipoprotein E | yes | 0.937 |
| *Icam2* | intercellular adhesion molecule 2 | yes | 0.805 |
| *Il27ra* | interleukin 27 receptor, alpha | yes | 0.71 |
| *Ogn* | osteoglycin | yes | 0.753 |
| *Lamb2* | laminin, beta 2 | yes | 0.696 |
| *Serpinf1* | serine (or cysteine) peptidase inhibitor, clade F, member 1 | yes | 0.831 |
| *Col8a1* | collagen, type VIII, alpha 1 | yes | 0.627 |
| *Pxdn* | peroxidasin | yes | 0.885 |
| *Cdh11* | cadherin 11 | yes | 0.455 |
| *Tgfb3* | transforming growth factor, beta 3 | yes | 0.836 |
| *Cdh2* | cadherin 2 | yes | 0.716 |
| *Ecm2* | extracellular matrix protein 2, female organ and adipocyte specific | yes | 0.859 |
| *C6* | complement component 6 | yes | 0.917 |
| *Egfr* | epidermal growth factor receptor | yes | 0.706 |
| *C7* | complement component 7 | yes | 0.822 |
| *Nov* | nephroblastoma overexpressed gene | yes | 0.923 |
| *Adamts16* | a disintegrin-like and metallopeptidase (reprolysin type) with thrombospondin type 1 motif, 16 | yes | 0.693 |
| *Apln* | apelin | yes | 0.888 |
| *Thbd* | thrombomodulin | yes | 0.783 |
| *Slc25a16* | solute carrier family 25 (mitochondrial carrier, Graves disease autoantigen), member 16 | yes | 0.481 |
| *Pla2g12a* | phospholipase A2, group XIIA | yes | 0.836 |
| *Cd79a* | CD79A antigen (immunoglobulin-associated alpha) | yes | 0.804 |
| *Fam150b* | family with sequence similarity 150, member B | yes | 0.779 |
| *Crispld2* | cysteine-rich secretory protein LCCL domain containing 2 | yes | 0.728 |
| *Col6a5* | collagen, type VI, alpha 5 | yes | 0.904 |
| *Smoc2* | SPARC related modular calcium binding 2 | yes | 0.755 |
| *Eln* | elastin | yes | 0.766 |
| *Ntn4* | netrin 4 | yes | 0.78 |
| *Itgbl1* | integrin, beta-like 1 | yes | 0.769 |
| *Pcdh12* | protocadherin 12 | yes | 0.734 |
| *Pcdh1* | protocadherin 1 | yes | 0.777 |
| *Cdnf* | cerebral dopamine neurotrophic factor | yes | 0.605 |
| *Il1rap* | interleukin 1 receptor accessory protein | yes | 0.677 |
| *Gm7120* | predicted gene 7120 | yes | 0.768 |
| *Defb47* | defensin beta 47 | yes | 0.865 |
| *F5* | coagulation factor V | yes | 0.776 |
| *Pon1* | paraoxonase 1 | yes | 0.787 |
| *Muc5b* | mucin 5, subtype B, tracheobronchial | yes | 0.89 |
| *Spag11b* | sperm associated antigen 11B | yes | 0.825 |
| *Lcn9* | lipocalin 9 | yes | 0.918 |
| *Cst8* | cystatin 8 (cystatin-related epididymal spermatogenic) | yes | 0.945 |
| *Defb20* | defensin beta 20 | yes | 0.934 |
| *Rnase13* | ribonuclease, RNase A family, 13 (non-active) | yes | 0.76 |
| *Cst12* | cystatin 12 | yes | 0.575 |
| *9230110F15Rik* | RIKEN cDNA 9230110F15 gene | yes | 0.846 |
| *Cst11* | cystatin 11 | yes | 0.671 |
| *Spink12* | serine peptidase inhibitor, Kazal type 12 | yes | 0.482 |
| *9230104L09Rik* | RIKEN cDNA 9230104L09 gene | yes | 0.837 |
| *Slc9a2* | solute carrier family 9 (sodium/hydrogen exchanger), member 2 | yes | 0.845 |
| *Gpx5* | glutathione peroxidase 5 | yes | 0.63 |
| *F10* | coagulation factor X | yes | 0.812 |
| *Ugt3a2* | UDP glycosyltransferases 3 family, polypeptide A2 | yes | 0.71 |
| *Spink2* | serine peptidase inhibitor, Kazal type 2 | yes | 0.832 |
| *Ros1* | Ros1 proto-oncogene | yes | 0.451 |
| *Defb25* | defensin beta 25 | yes | 0.839 |
| *Spock1* | sparc/osteonectin, cwcv and kazal-like domains proteoglycan 1 | yes | 0.544 |
| *Proz* | protein Z, vitamin K-dependent plasma glycoprotein | yes | 0.811 |
| *Gm4788* | predicted gene 4788 | yes | 0.803 |
| *Gm17727* | predicted gene, 17727 | yes | 0.844 |
| *Itih3* | inter-alpha trypsin inhibitor, heavy chain 3 | yes | 0.659 |
| *Vtn* | vitronectin | yes | 0.852 |
| *Itih2* | inter-alpha trypsin inhibitor, heavy chain 2 | yes | 0.881 |
| *Lcat* | lecithin cholesterol acyltransferase | yes | 0.622 |
| *Lipg* | lipase, endothelial | yes | 0.569 |
| *Rdh7* | retinol dehydrogenase 7 | yes | 0.703 |
| *Alb* | albumin | yes | 0.852 |
| *Kng1* | kininogen 1 | yes | 0.842 |
| *Azgp1* | alpha-2-glycoprotein 1, zinc | yes | 0.619 |
| *Serpina1b* | serine (or cysteine) preptidase inhibitor, clade A, member 1B | yes | 0.903 |
| *Apoh* | apolipoprotein H | yes | 0.835 |
| *Ahsg* | alpha-2-HS-glycoprotein | yes | 0.82 |
| *Serpina1d* | serine (or cysteine) peptidase inhibitor, clade A, member 1D | yes | 0.883 |
| *Serpinc1* | serine (or cysteine) peptidase inhibitor, clade C (antithrombin), member 1 | yes | 0.727 |
| *AI182371* | expressed sequence AI182371 | yes | 0.867 |
| *Hpx* | hemopexin | yes | 0.933 |
| *Apoa2* | apolipoprotein A-II | yes | 0.81 |
| *Ambp* | alpha 1 microglobulin/bikunin | yes | 0.798 |
| *Gm15386* | defensin beta 41 | yes | 0.866 |
| *Serpina1a* | serine (or cysteine) peptidase inhibitor, clade A, member 1A | yes | 0.903 |
| *Pzp* | pregnancy zone protein | yes | 0.786 |
| *Lcn8* | lipocalin 8 | yes | 0.654 |
| *Adam28* | a disintegrin and metallopeptidase domain 28 | yes | 0.808 |
| *Angptl3* | angiopoietin-like 3 | yes | 0.838 |
| *Defb48* | defensin beta 48 | yes | 0.848 |
| *Cyp2c44* | cytochrome P450, family 2, subfamily c, polypeptide 44 | yes | 0.742 |
| *Mup3* | major urinary protein 3 | yes | 0.868 |
| *Serpina1c* | serine (or cysteine) peptidase inhibitor, clade A, member 1C | yes | 0.903 |
| *Mup20* | major urinary protein 20 | yes | 0.931 |
| *Ovch2* | ovochymase 2 | yes | 0.45 |
| *Serpina1e* | serine (or cysteine) peptidase inhibitor, clade A, member 1E | yes | 0.915 |
| *D730048I06Rik* | RIKEN cDNA D730048I06 gene | yes | 0.788 |
| *Lcn5* | lipocalin 5 | yes | 0.811 |
| *Spink5* | serine peptidase inhibitor, Kazal type 5 | yes | 0.707 |
| *Ly6g5b* | lymphocyte antigen 6 complex, locus G5B | yes | 0.658 |

Supplementary Table 10. Statistically overrepresented Gene Ontology biological processes based on positively identified signaling genes

| **GOID** | **GOTerm** | **Nr. Genes** | **FDR** | **Associated Genes Found** |
| --- | --- | --- | --- | --- |
| GO:0001523 | retinoid metabolic process | 5 | <0.001 | *Lcn5, Lcn8, Rarres2, Rdh11, Rdh7* |
| GO:0001659 | temperature homeostasis | 4 | 0.001 | *Apln, Mup20, Mup3, Slc27a1* |
| GO:0001706 | endoderm formation | 3 | 0.008 | *Col8a1, Itgb5, Vtn* |
| GO:0001937 | negative regulation of endothelial cell proliferation | 3 | 0.002 | *Acvrl1, Apoe, Apoh* |
| GO:0001952 | regulation of cell-matrix adhesion | 4 | 0.004 | *Acvrl1, Adam15, Csf1, Vegfc* |
| GO:0002367 | cytokine production involved in immune response | 3 | 0.014 | *Apoa2, Tgfb3, Tril* |
| GO:0002455 | humoral immune response mediated by circulating immunoglobulin | 3 | 0.011 | *C4b, H2-Ab1, Hpx* |
| GO:0002526 | acute inflammatory response | 7 | <0.001 | *Ahsg, Apoa2, C6, Lbp, Pzp, Serpina1a, Serpina1b* |
| GO:0002688 | regulation of leukocyte chemotaxis | 5 | 0.001 | *Csf1, Lbp, Nov, Rarres2, Vegfc* |
| GO:0002690 | positive regulation of leukocyte chemotaxis | 4 | 0.004 | *Csf1, Lbp, Rarres2, Vegfc* |
| GO:0002698 | negative regulation of immune effector process | 5 | 0.002 | *Apoa2, Htra1, Pzp, Spink5, Tgfb3* |
| GO:0002700 | regulation of production of molecular mediator of immune response | 6 | <0.001 | *Apoa2, Hpx, Il27ra, Spink5, Tgfb3, Tril* |
| GO:0002701 | negative regulation of production of molecular mediator of immune response | 3 | 0.002 | *Apoa2, Spink5, Tgfb3* |
| GO:0002718 | regulation of cytokine production involved in immune response | 3 | 0.008 | *Apoa2, Tgfb3, Tril* |
| GO:0002792 | negative regulation of peptide secretion | 3 | 0.013 | *Mup20, Mup3, Nov* |
| GO:0002821 | positive regulation of adaptive immune response | 4 | 0.006 | *H2-Ab1, Hpx, Il27ra, Pvr* |
| GO:0002824 | positive regulation of adaptive immune response based on somatic recombination of immune receptors built from immunoglobulin superfamily domains | 4 | 0.005 | *H2-Ab1, Hpx, Il27ra, Pvr* |
| GO:0002920 | regulation of humoral immune response | 4 | <0.001 | *C6, Hpx, Pzp, Spink5* |
| GO:0006487 | protein N-linked glycosylation | 3 | 0.016 | *D730048I06Rik, Serpina1a, Serpina1b* |
| GO:0006576 | cellular biogenic amine metabolic process | 4 | 0.005 | *Apoa2, Lcat, Pon1, Slc27a1* |
| GO:0006639 | acylglycerol metabolic process | 4 | 0.007 | *Angptl3, Apoa2, Apoe, Apoh* |
| GO:0006656 | phosphatidylcholine biosynthetic process | 3 | 0.001 | *Apoa2, Lcat, Slc27a1* |
| GO:0006720 | isoprenoid metabolic process | 5 | 0.001 | *Lcn5, Lcn8, Rarres2, Rdh11, Rdh7* |
| GO:0006721 | terpenoid metabolic process | 5 | <0.001 | *Lcn5, Lcn8, Rarres2, Rdh11, Rdh7* |
| GO:0006953 | acute-phase response | 4 | 0.001 | *Ahsg, Lbp, Serpina1a, Serpina1b* |
| GO:0006956 | complement activation | 4 | 0.002 | *AI182371, C4b, C6, Pzp* |
| GO:0006959 | humoral immune response | 7 | <0.001 | *AI182371, C4b, C6, H2-Ab1, Hpx, Pzp, Spink5* |
| GO:0007157 | heterophilic cell-cell adhesion via plasma membrane cell adhesion molecules | 3 | 0.006 | *Cdh2, Mcam, Pvr* |
| GO:0007160 | cell-matrix adhesion | 8 | <0.001 | *Acvrl1, Adam15, Angptl3, Csf1, Itgb5, Nid2, Vegfc, Vtn* |
| GO:0007179 | transforming growth factor beta receptor signaling pathway | 7 | <0.001 | *Acvrl1, Fbn1, Htra1, Itgb5, Ltbp3, Tgfb3, Vasn* |
| GO:0007405 | neuroblast proliferation | 3 | 0.012 | *Fgfr1, Smo, Vegfc* |
| GO:0007416 | synapse assembly | 6 | 0.001 | *Cdh2, Chrnb2, Ephb2, Il1rap, Lrtm1, Plxnd1* |
| GO:0007431 | salivary gland development | 6 | <0.001 | *Egfr, Fgfr1, Ntn4, Plxna1, Plxnd1, Tgfb3* |
| GO:0007435 | salivary gland morphogenesis | 6 | <0.001 | *Egfr, Fgfr1, Ntn4, Plxna1, Plxnd1, Tgfb3* |
| GO:0007596 | blood coagulation | 9 | <0.001 | *Apoe, Apoh, F10, F5, Kng1, Proz, Serpinc1, Tfpi, Thbd* |
| GO:0007599 | hemostasis | 9 | <0.001 | *Apoe, Apoh, F10, F5, Kng1, Proz, Serpinc1, Tfpi, Thbd* |
| GO:0008203 | cholesterol metabolic process | 6 | <0.001 | *Angptl3, Apoa2, Apoe, Lcat, Pon1, Sorl1* |
| GO:0010466 | negative regulation of peptidase activity | 26 | <0.001 | *9230104L09Rik, AI182371, Ahsg, Ambp, C4b, Cst11, Cst12, Cst8, Itih2, Itih3, Kng1, Pzp, Serpina1a, Serpina1b, Serpina1c, Serpina1d, Serpina1e, Serpinc1, Serpinf1, Sorl1, Spink12, Spink2, Spink5, Spock1, Tfpi, Vtn* |
| GO:0010594 | regulation of endothelial cell migration | 6 | <0.001 | *Acvrl1, Apoe, Apoh, Fgfr1, Serpinf1, Vegfc* |
| GO:0010596 | negative regulation of endothelial cell migration | 4 | 0.001 | *Acvrl1, Apoe, Apoh, Serpinf1* |
| GO:0010633 | negative regulation of epithelial cell migration | 4 | 0.001 | *Acvrl1, Apoe, Apoh, Serpinf1* |
| GO:0010810 | regulation of cell-substrate adhesion | 9 | <0.001 | *Acvrl1, Adam15, Col8a1, Csf1, Ecm2, Smoc2, Spock1, Vegfc, Vtn* |
| GO:0010811 | positive regulation of cell-substrate adhesion | 6 | <0.001 | *Col8a1, Csf1, Ecm2, Smoc2, Vegfc, Vtn* |
| GO:0010812 | negative regulation of cell-substrate adhesion | 3 | 0.006 | *Acvrl1, Adam15, Spock1* |
| GO:0010951 | negative regulation of endopeptidase activity | 26 | <0.001 | *9230104L09Rik, AI182371, Ahsg, Ambp, C4b, Cst11, Cst12, Cst8, Itih2, Itih3, Kng1, Pzp, Serpina1a, Serpina1b, Serpina1c, Serpina1d, Serpina1e, Serpinc1, Serpinf1, Sorl1, Spink12, Spink2, Spink5, Spock1, Tfpi, Vtn* |
| GO:0014812 | muscle cell migration | 5 | <0.001 | *Nov, Plxna1, Smo, Sorl1, Vtn* |
| GO:0014909 | smooth muscle cell migration | 4 | 0.001 | *Nov, Plxna1, Sorl1, Vtn* |
| GO:0014910 | regulation of smooth muscle cell migration | 3 | 0.006 | *Plxna1, Sorl1, Vtn* |
| GO:0015918 | sterol transport | 5 | <0.001 | *Apoa2, Apoe, Lcat, Lipg, Pon1* |
| GO:0016101 | diterpenoid metabolic process | 5 | <0.001 | *Lcn5, Lcn8, Rarres2, Rdh11, Rdh7* |
| GO:0016125 | sterol metabolic process | 6 | 0.001 | *Angptl3, Apoa2, Apoe, Lcat, Pon1, Sorl1* |
| GO:0016339 | calcium-dependent cell-cell adhesion via plasma membrane cell adhesion molecules | 3 | 0.001 | *Cdh2, Pcdh1, Pcdh12* |
| GO:0017015 | regulation of transforming growth factor beta receptor signaling pathway | 4 | 0.005 | *Fbn1, Htra1, Tgfb3, Vasn* |
| GO:0021952 | central nervous system projection neuron axonogenesis | 3 | 0.001 | *Cdh11, Chrnb2, Ephb2* |
| GO:0021954 | central nervous system neuron development | 4 | 0.005 | *Cdh11, Chrnb2, Ephb2, Fgfr1* |
| GO:0021955 | central nervous system neuron axonogenesis | 3 | 0.002 | *Cdh11, Chrnb2, Ephb2* |
| GO:0022612 | gland morphogenesis | 7 | <0.001 | *Csf1, Egfr, Fgfr1, Ntn4, Plxna1, Plxnd1, Tgfb3* |
| GO:0030162 | regulation of proteolysis | 29 | <0.001 | *9230104L09Rik, AI182371, Ahsg, Ambp, Apoe, C4b, C6, Cst11, Cst12, Cst8, Itih2, Itih3, Kng1, Ldlrad3, Pzp, Serpina1a, Serpina1b, Serpina1c, Serpina1d, Serpina1e, Serpinc1, Serpinf1, Sorl1, Spink12, Spink2, Spink5, Spock1, Tfpi, Vtn* |
| GO:0030193 | regulation of blood coagulation | 5 | 0.001 | *Apoe, Apoh, Kng1, Serpinc1, Thbd* |
| GO:0030195 | negative regulation of blood coagulation | 4 | 0.001 | *Apoe, Apoh, Kng1, Thbd* |
| GO:0030198 | extracellular matrix organization | 11 | <0.001 | *Adamtsl4, Col18a1, Crispld2, Ecm2, Eln, Fbln5, Lamb2, Ltbp3, Pxdn, Smoc2, Vtn* |
| GO:0030279 | negative regulation of ossification | 3 | 0.015 | *Ahsg, Fgfr1, Ltbp3* |
| GO:0030301 | cholesterol transport | 5 | <0.001 | *Apoa2, Apoe, Lcat, Lipg, Pon1* |
| GO:0030500 | regulation of bone mineralization | 3 | 0.016 | *Ahsg, Ltbp3, Tgfb3* |
| GO:0030512 | negative regulation of transforming growth factor beta receptor signaling pathway | 4 | 0.001 | *Fbn1, Htra1, Tgfb3, Vasn* |
| GO:0030516 | regulation of axon extension | 4 | 0.005 | *Apoe, Plxna1, Plxnd1, Sema4c* |
| GO:0031102 | neuron projection regeneration | 3 | 0.007 | *Apoe, Lamb2, Rtn4rl1* |
| GO:0031103 | axon regeneration | 3 | 0.005 | *Apoe, Lamb2, Rtn4rl1* |
| GO:0031348 | negative regulation of defense response | 7 | <0.001 | *Apoe, Htra1, Nov, Pzp, Serpinc1, Serpinf1, Spink5* |
| GO:0031589 | cell-substrate adhesion | 12 | <0.001 | *Acvrl1, Adam15, Angptl3, Col8a1, Csf1, Ecm2, Itgb5, Nid2, Smoc2, Spock1, Vegfc, Vtn* |
| GO:0031649 | heat generation | 4 | <0.001 | *Apln, Mup20, Mup3, Slc27a1* |
| GO:0032368 | regulation of lipid transport | 4 | 0.005 | *Apoa2, Apoe, Lipg, Pon1* |
| GO:0032370 | positive regulation of lipid transport | 3 | 0.008 | *Apoe, Lipg, Pon1* |
| GO:0032371 | regulation of sterol transport | 4 | <0.001 | *Apoa2, Apoe, Lipg, Pon1* |
| GO:0032373 | positive regulation of sterol transport | 3 | 0.001 | *Apoe, Lipg, Pon1* |
| GO:0032374 | regulation of cholesterol transport | 4 | <0.001 | *Apoa2, Apoe, Lipg, Pon1* |
| GO:0032376 | positive regulation of cholesterol transport | 3 | 0.001 | *Apoe, Lipg, Pon1* |
| GO:0032768 | regulation of monooxygenase activity | 3 | 0.004 | *Apoe, Egfr, Npr3* |
| GO:0033344 | cholesterol efflux | 3 | 0.005 | *Apoa2, Apoe, Pon1* |
| GO:0034367 | macromolecular complex remodeling | 4 | <0.001 | *Apoa2, Apoe, Lcat, Lipg* |
| GO:0034368 | protein-lipid complex remodeling | 4 | <0.001 | *Apoa2, Apoe, Lcat, Lipg* |
| GO:0034369 | plasma lipoprotein particle remodeling | 4 | <0.001 | *Apoa2, Apoe, Lcat, Lipg* |
| GO:0034370 | triglyceride-rich lipoprotein particle remodeling | 3 | <0.001 | *Apoa2, Apoe, Lcat* |
| GO:0034372 | very-low-density lipoprotein particle remodeling | 3 | <0.001 | *Apoa2, Apoe, Lcat* |
| GO:0034375 | high-density lipoprotein particle remodeling | 4 | <0.001 | *Apoa2, Apoe, Lcat, Lipg* |
| GO:0034377 | plasma lipoprotein particle assembly | 3 | 0.001 | *Apoa2, Apoe, Lcat* |
| GO:0034380 | high-density lipoprotein particle assembly | 3 | <0.001 | *Apoa2, Apoe, Lcat* |
| GO:0034381 | plasma lipoprotein particle clearance | 3 | 0.002 | *Apoa2, Apoe, Lipg* |
| GO:0034384 | high-density lipoprotein particle clearance | 3 | <0.001 | *Apoa2, Apoe, Lipg* |
| GO:0034433 | steroid esterification | 3 | <0.001 | *Apoa2, Apoe, Lcat* |
| GO:0034434 | sterol esterification | 3 | <0.001 | *Apoa2, Apoe, Lcat* |
| GO:0034435 | cholesterol esterification | 3 | <0.001 | *Apoa2, Apoe, Lcat* |
| GO:0034754 | cellular hormone metabolic process | 4 | 0.006 | *Lcn5, Lcn8, Rdh11, Rdh7* |
| GO:0035272 | exocrine system development | 6 | <0.001 | *Egfr, Fgfr1, Ntn4, Plxna1, Plxnd1, Tgfb3* |
| GO:0035987 | endodermal cell differentiation | 3 | 0.005 | *Col8a1, Itgb5, Vtn* |
| GO:0042088 | T-helper 1 type immune response | 3 | 0.004 | *H2-Ab1, Il18r1, Il27ra* |
| GO:0042307 | positive regulation of protein import into nucleus | 4 | 0.006 | *Egfr, Il18r1, Smo, Tgfb3* |
| GO:0042311 | vasodilation | 3 | 0.014 | *Apln, Apoe, Kng1* |
| GO:0042439 | ethanolamine-containing compound metabolic process | 4 | 0.001 | *Apoa2, Lcat, Pon1, Slc27a1* |
| GO:0042632 | cholesterol homeostasis | 5 | <0.001 | *Angptl3, Apoa2, Apoe, Lcat, Lipg* |
| GO:0042982 | amyloid precursor protein metabolic process | 3 | 0.002 | *Apoe, Bace2, Sorl1* |
| GO:0043062 | extracellular structure organization | 11 | <0.001 | *Adamtsl4, Col18a1, Crispld2, Ecm2, Eln, Fbln5, Lamb2, Ltbp3, Pxdn, Smoc2, Vtn* |
| GO:0043086 | negative regulation of catalytic activity | 33 | <0.001 | *9230104L09Rik, AI182371, Ahsg, Ambp, Angptl3, Apoa2, Apoe, C4b, Cst11, Cst12, Cst8, Itih2, Itih3, Kng1, Lrtm1, Npr3, Pzp, Rtn4rl1, Serpina1a, Serpina1b, Serpina1c, Serpina1d, Serpina1e, Serpinc1, Serpinf1, Sfrp5, Sorl1, Spink12, Spink2, Spink5, Spock1, Tfpi, Vtn* |
| GO:0043407 | negative regulation of MAP kinase activity | 3 | 0.014 | *Apoe, Sfrp5, Sorl1* |
| GO:0043534 | blood vessel endothelial cell migration | 3 | 0.017 | *Acvrl1, Apoe, Vegfc* |
| GO:0043535 | regulation of blood vessel endothelial cell migration | 3 | 0.007 | *Acvrl1, Apoe, Vegfc* |
| GO:0043542 | endothelial cell migration | 8 | <0.001 | *Acvrl1, Apoe, Apoh, Fgfr1, Nov, Plxnd1, Serpinf1, Vegfc* |
| GO:0043691 | reverse cholesterol transport | 4 | <0.001 | *Apoa2, Apoe, Lcat, Lipg* |
| GO:0044106 | cellular amine metabolic process | 5 | 0.003 | *Apoa2, Chrnb2, Lcat, Pon1, Slc27a1* |
| GO:0045833 | negative regulation of lipid metabolic process | 5 | 0.001 | *Apoa2, Apoe, Mup20, Mup3, Slc27a1* |
| GO:0045834 | positive regulation of lipid metabolic process | 6 | 0.001 | *Angptl3, Apoa2, Apoe, Apoh, Mup20, Mup3* |
| GO:0045861 | negative regulation of proteolysis | 26 | <0.001 | *9230104L09Rik, AI182371, Ahsg, Ambp, C4b, Cst11, Cst12, Cst8, Itih2, Itih3, Kng1, Pzp, Serpina1a, Serpina1b, Serpina1c, Serpina1d, Serpina1e, Serpinc1, Serpinf1, Sorl1, Spink12, Spink2, Spink5, Spock1, Tfpi, Vtn* |
| GO:0046461 | neutral lipid catabolic process | 3 | 0.001 | *Apoa2, Apoe, Apoh* |
| GO:0046464 | acylglycerol catabolic process | 3 | 0.001 | *Apoa2, Apoe, Apoh* |
| GO:0046470 | phosphatidylcholine metabolic process | 4 | <0.001 | *Apoa2, Lcat, Pon1, Slc27a1* |
| GO:0046503 | glycerolipid catabolic process | 3 | 0.003 | *Apoa2, Apoe, Apoh* |
| GO:0046676 | negative regulation of insulin secretion | 3 | 0.009 | *Mup20, Mup3, Nov* |
| GO:0048512 | circadian behavior | 4 | 0.001 | *Alb, Chrnb2, Mup20, Mup3* |
| GO:0048520 | positive regulation of behavior | 6 | 0.001 | *Alb, Csf1, Fgfr1, Lbp, Rarres2, Vegfc* |
| GO:0048675 | axon extension | 6 | 0.001 | *Apoe, Lamb2, Ogn, Plxna1, Plxnd1, Sema4c* |
| GO:0048678 | response to axon injury | 3 | 0.016 | *Apoe, Lamb2, Rtn4rl1* |
| GO:0048841 | regulation of axon extension involved in axon guidance | 3 | 0.003 | *Plxna1, Plxnd1, Sema4c* |
| GO:0048846 | axon extension involved in axon guidance | 4 | 0.001 | *Ogn, Plxna1, Plxnd1, Sema4c* |
| GO:0050435 | beta-amyloid metabolic process | 3 | 0.002 | *Apoe, Bace2, Sorl1* |
| GO:0050770 | regulation of axonogenesis | 7 | 0.001 | *Apoe, Cdh2, Ephb2, Ogn, Plxna1, Plxnd1, Sema4c* |
| GO:0050777 | negative regulation of immune response | 5 | 0.003 | *Apoa2, Il27ra, Pzp, Spink5, Tgfb3* |
| GO:0050795 | regulation of behavior | 11 | <0.001 | *Alb, Chrnb2, Csf1, Fgfr1, Lbp, Nov, Plxna1, Plxnd1, Rarres2, Sema4c, Vegfc* |
| GO:0050807 | regulation of synapse organization | 6 | 0.001 | *Apoe, Cdh2, Chrnb2, Ephb2, Il1rap, Lrtm1* |
| GO:0050817 | coagulation | 9 | <0.001 | *Apoe, Apoh, F10, F5, Kng1, Proz, Serpinc1, Tfpi, Thbd* |
| GO:0050818 | regulation of coagulation | 5 | 0.001 | *Apoe, Apoh, Kng1, Serpinc1, Thbd* |
| GO:0050819 | negative regulation of coagulation | 4 | 0.001 | *Apoe, Apoh, Kng1, Thbd* |
| GO:0050920 | regulation of chemotaxis | 9 | <0.001 | *Csf1, Fgfr1, Lbp, Nov, Plxna1, Plxnd1, Rarres2, Sema4c, Vegfc* |
| GO:0050921 | positive regulation of chemotaxis | 5 | 0.003 | *Csf1, Fgfr1, Lbp, Rarres2, Vegfc* |
| GO:0050994 | regulation of lipid catabolic process | 4 | 0.001 | *Angptl3, Apoa2, Apoh, Rarres2* |
| GO:0050996 | positive regulation of lipid catabolic process | 3 | 0.001 | *Angptl3, Apoa2, Apoh* |
| GO:0050999 | regulation of nitric-oxide synthase activity | 3 | 0.002 | *Apoe, Egfr, Npr3* |
| GO:0051055 | negative regulation of lipid biosynthetic process | 4 | 0.001 | *Apoe, Mup20, Mup3, Slc27a1* |
| GO:0051291 | protein heterooligomerization | 4 | 0.006 | *Acvrl1, Cdh2, Chrnb2, Col6a2* |
| GO:0051341 | regulation of oxidoreductase activity | 3 | 0.016 | *Apoe, Egfr, Npr3* |
| GO:0051346 | negative regulation of hydrolase activity | 28 | <0.001 | *9230104L09Rik, AI182371, Ahsg, Ambp, Angptl3, Apoa2, C4b, Cst11, Cst12, Cst8, Itih2, Itih3, Kng1, Pzp, Serpina1a, Serpina1b, Serpina1c, Serpina1d, Serpina1e, Serpinc1, Serpinf1, Sorl1, Spink12, Spink2, Spink5, Spock1, Tfpi, Vtn* |
| GO:0051897 | positive regulation of protein kinase B signaling | 4 | 0.007 | *Egfr, F10, Mup20, Mup3* |
| GO:0051963 | regulation of synapse assembly | 4 | 0.005 | *Chrnb2, Ephb2, Il1rap, Lrtm1* |
| GO:0051965 | positive regulation of synapse assembly | 3 | 0.014 | *Ephb2, Il1rap, Lrtm1* |
| GO:0052547 | regulation of peptidase activity | 26 | <0.001 | *9230104L09Rik, AI182371, Ahsg, Ambp, C4b, Cst11, Cst12, Cst8, Itih2, Itih3, Kng1, Pzp, Serpina1a, Serpina1b, Serpina1c, Serpina1d, Serpina1e, Serpinc1, Serpinf1, Sorl1, Spink12, Spink2, Spink5, Spock1, Tfpi, Vtn* |
| GO:0052548 | regulation of endopeptidase activity | 26 | <0.001 | *9230104L09Rik, AI182371, Ahsg, Ambp, C4b, Cst11, Cst12, Cst8, Itih2, Itih3, Kng1, Pzp, Serpina1a, Serpina1b, Serpina1c, Serpina1d, Serpina1e, Serpinc1, Serpinf1, Sorl1, Spink12, Spink2, Spink5, Spock1, Tfpi, Vtn* |
| GO:0055081 | anion homeostasis | 3 | 0.006 | *Angptl3, Apoe, Lipg* |
| GO:0055088 | lipid homeostasis | 5 | 0.002 | *Angptl3, Apoa2, Apoe, Lcat, Lipg* |
| GO:0055092 | sterol homeostasis | 5 | <0.001 | *Angptl3, Apoa2, Apoe, Lcat, Lipg* |
| GO:0060191 | regulation of lipase activity | 4 | 0.002 | *Angptl3, Apoa2, Apoh, Fgfr1* |
| GO:0060445 | branching involved in salivary gland morphogenesis | 4 | <0.001 | *Fgfr1, Ntn4, Plxna1, Plxnd1* |
| GO:0060688 | regulation of morphogenesis of a branching structure | 3 | 0.011 | *Fgfr1, Ntn4, Smo* |
| GO:0060840 | artery development | 5 | 0.001 | *Acvrl1, Angptl3, Apoe, Eln, Plxnd1* |
| GO:0061041 | regulation of wound healing | 5 | 0.003 | *Apoe, Apoh, Kng1, Serpinc1, Thbd* |
| GO:0061045 | negative regulation of wound healing | 4 | 0.001 | *Apoe, Apoh, Kng1, Thbd* |
| GO:0065005 | protein-lipid complex assembly | 3 | 0.001 | *Apoa2, Apoe, Lcat* |
| GO:0070613 | regulation of protein processing | 3 | 0.014 | *C6, Ldlrad3, Pzp* |
| GO:0071384 | cellular response to corticosteroid stimulus | 3 | 0.012 | *Eln, H2-Ab1, Serpinf1* |
| GO:0071385 | cellular response to glucocorticoid stimulus | 3 | 0.011 | *Eln, H2-Ab1, Serpinf1* |
| GO:0071526 | semaphorin-plexin signaling pathway | 3 | 0.003 | *Plxna1, Plxnd1, Sema4c* |
| GO:0071548 | response to dexamethasone | 3 | 0.003 | *Eln, H2-Ab1, Serpinf1* |
| GO:0071549 | cellular response to dexamethasone stimulus | 3 | 0.002 | *Eln, H2-Ab1, Serpinf1* |
| GO:0071560 | cellular response to transforming growth factor beta stimulus | 8 | <0.001 | *Acvrl1, Eln, Fbn1, Htra1, Itgb5, Ltbp3, Tgfb3, Vasn* |
| GO:0071622 | regulation of granulocyte chemotaxis | 3 | 0.005 | *Csf1, Lbp, Rarres2* |
| GO:0071825 | protein-lipid complex subunit organization | 4 | <0.001 | *Apoa2, Apoe, Lcat, Lipg* |
| GO:0071827 | plasma lipoprotein particle organization | 4 | <0.001 | *Apoa2, Apoe, Lcat, Lipg* |
| GO:0072376 | protein activation cascade | 6 | <0.001 | *AI182371, Apoh, C4b, C6, Pzp, Serpinc1* |
| GO:0090101 | negative regulation of transmembrane receptor protein serine/threonine kinase signaling pathway | 5 | 0.001 | *Fbn1, Htra1, Nov, Tgfb3, Vasn* |
| GO:0090278 | negative regulation of peptide hormone secretion | 3 | 0.012 | *Mup20, Mup3, Nov* |
| GO:0097006 | regulation of plasma lipoprotein particle levels | 4 | 0.001 | *Apoa2, Apoe, Lcat, Lipg* |
| GO:0098754 | detoxification | 4 | 0.005 | *Apoe, Fbln5, Gpx5, Pxdn* |
| GO:0098869 | cellular oxidant detoxification | 4 | 0.004 | *Apoe, Fbln5, Gpx5, Pxdn* |
| GO:1900046 | regulation of hemostasis | 5 | 0.001 | *Apoe, Apoh, Kng1, Serpinc1, Thbd* |
| GO:1900047 | negative regulation of hemostasis | 4 | 0.001 | *Apoe, Apoh, Kng1, Thbd* |
| GO:1901655 | cellular response to ketone | 4 | 0.004 | *Eln, H2-Ab1, Serpinf1, Ugt3a2* |
| GO:1902284 | neuron projection extension involved in neuron projection guidance | 4 | 0.001 | *Ogn, Plxna1, Plxnd1, Sema4c* |
| GO:1902652 | secondary alcohol metabolic process | 7 | <0.001 | *Angptl3, Apoa2, Apoe, Fgfr1, Lcat, Pon1, Sorl1* |
| GO:1902667 | regulation of axon guidance | 3 | 0.004 | *Plxna1, Plxnd1, Sema4c* |
| GO:1903035 | negative regulation of response to wounding | 7 | 0.001 | *Apoe, Apoh, Kng1, Nov, Serpinc1, Serpinf1, Thbd* |
| GO:1903317 | regulation of protein maturation | 3 | 0.014 | *C6, Ldlrad3, Pzp* |
| GO:1903844 | regulation of cellular response to transforming growth factor beta stimulus | 4 | 0.005 | *Fbn1, Htra1, Tgfb3, Vasn* |
| GO:1903845 | negative regulation of cellular response to transforming growth factor beta stimulus | 4 | 0.001 | *Fbn1, Htra1, Tgfb3, Vasn* |
| GO:1904591 | positive regulation of protein import | 4 | 0.006 | *Egfr, Il18r1, Smo, Tgfb3* |
| GO:1990748 | cellular detoxification | 4 | 0.004 | *Apoe, Fbln5, Gpx5, Pxdn* |
| GO:2000257 | regulation of protein activation cascade | 3 | 0.001 | *C6, Pzp, Serpinc1* |
| GO:2000648 | positive regulation of stem cell proliferation | 4 | 0.004 | *Fgfr1, Ltbp3, Smo, Vegfc* |

Supplementary Table 11. Expression levels of signaling genes correlated with levels of circulating hormones

|  | **Leptin** |  | **TNF-a** |  | **Insulin** |  | **IL 6** |  | **Resistin** |  | **IGF-1** |  |
| --- | --- | --- | --- | --- | --- | --- | --- | --- | --- | --- | --- | --- |
|  | **r** | **p-value** | **r** | **p-value** | **r** | **p-value** | **r** | **p-value** | **r** | **p-value** | **r** | **p-value** |
| *9230104L09Rik* | -0.158 | 0.404 | -0.179 | 0.344 | -0.173 | 0.362 | 0.404 | 0.027 | -0.205 | 0.277 | 0.143 | 0.451 |
| *9230110F15Rik* | -0.176 | 0.352 | -0.180 | 0.341 | -0.154 | 0.416 | 0.416 | 0.022 | -0.187 | 0.323 | 0.113 | 0.551 |
| *Acvrl1* | 0.658 | <0.001 | 0.446 | 0.014 | 0.513 | 0.004 | -0.483 | 0.007 | 0.250 | 0.183 | 0.539 | 0.002 |
| *Adam15* | 0.655 | <0.001 | 0.469 | 0.009 | 0.538 | 0.002 | -0.496 | 0.005 | 0.293 | 0.116 | 0.533 | 0.002 |
| *Adam28* | -0.139 | 0.463 | -0.177 | 0.349 | -0.192 | 0.308 | 0.402 | 0.028 | -0.246 | 0.190 | 0.170 | 0.369 |
| *Adamts16* | 0.421 | 0.021 | 0.518 | 0.003 | 0.384 | 0.036 | -0.220 | 0.242 | 0.177 | 0.349 | 0.296 | 0.113 |
| *Adamts5* | 0.644 | <0.001 | 0.546 | 0.002 | 0.535 | 0.002 | -0.445 | 0.014 | 0.118 | 0.536 | 0.466 | 0.010 |
| *Adamtsl4* | 0.611 | <0.001 | 0.389 | 0.034 | 0.392 | 0.032 | -0.540 | 0.002 | 0.202 | 0.285 | 0.503 | 0.005 |
| *Ahsg* | 0.382 | 0.037 | 0.222 | 0.238 | 0.291 | 0.118 | -0.214 | 0.256 | 0.094 | 0.620 | 0.272 | 0.146 |
| *AI182371* | 0.379 | 0.039 | 0.214 | 0.256 | 0.276 | 0.139 | -0.206 | 0.275 | 0.090 | 0.638 | 0.269 | 0.151 |
| *Alb* | 0.385 | 0.036 | 0.223 | 0.235 | 0.294 | 0.114 | -0.216 | 0.253 | 0.091 | 0.632 | 0.275 | 0.141 |
| *Ambp* | 0.377 | 0.040 | 0.221 | 0.241 | 0.290 | 0.120 | -0.212 | 0.262 | 0.091 | 0.633 | 0.270 | 0.149 |
| *Angptl3* | 0.370 | 0.044 | 0.219 | 0.246 | 0.308 | 0.098 | -0.218 | 0.248 | 0.103 | 0.587 | 0.270 | 0.149 |
| *Antxr2* | 0.649 | <0.001 | 0.450 | 0.013 | 0.508 | 0.004 | -0.536 | 0.002 | 0.238 | 0.205 | 0.433 | 0.017 |
| *Apln* | 0.577 | 0.001 | 0.579 | 0.001 | 0.425 | 0.019 | -0.496 | 0.005 | 0.186 | 0.325 | 0.347 | 0.060 |
| *Apoa2* | 0.379 | 0.039 | 0.222 | 0.238 | 0.290 | 0.120 | -0.212 | 0.261 | 0.094 | 0.621 | 0.270 | 0.148 |
| *Apoe* | 0.588 | 0.001 | 0.518 | 0.003 | 0.492 | 0.006 | -0.428 | 0.018 | 0.184 | 0.330 | 0.427 | 0.019 |
| *Apoh* | 0.379 | 0.039 | 0.221 | 0.240 | 0.291 | 0.118 | -0.212 | 0.261 | 0.091 | 0.632 | 0.271 | 0.147 |
| *Azgp1* | 0.392 | 0.032 | 0.198 | 0.294 | 0.303 | 0.103 | -0.254 | 0.176 | 0.094 | 0.621 | 0.265 | 0.157 |
| *Bace2* | 0.716 | <0.001 | 0.426 | 0.019 | 0.397 | 0.030 | -0.543 | 0.002 | 0.140 | 0.461 | 0.546 | 0.002 |
| *C4b* | 0.640 | <0.001 | 0.455 | 0.012 | 0.455 | 0.012 | -0.431 | 0.017 | 0.236 | 0.210 | 0.458 | 0.011 |
| *C6* | 0.627 | <0.001 | 0.336 | 0.070 | 0.431 | 0.017 | -0.390 | 0.033 | 0.110 | 0.562 | 0.453 | 0.012 |
| *C7* | 0.589 | 0.001 | 0.281 | 0.132 | 0.409 | 0.025 | -0.203 | 0.283 | 0.332 | 0.073 | 0.483 | 0.007 |
| *Cd248* | 0.628 | <0.001 | 0.523 | 0.003 | 0.478 | 0.008 | -0.546 | 0.002 | 0.257 | 0.171 | 0.508 | 0.004 |
| *Cd79a* | 0.621 | <0.001 | 0.472 | 0.008 | 0.438 | 0.015 | -0.209 | 0.267 | 0.019 | 0.922 | 0.526 | 0.003 |
| *Cdh11* | 0.466 | 0.009 | 0.332 | 0.073 | 0.209 | 0.269 | -0.257 | 0.171 | 0.065 | 0.732 | 0.385 | 0.036 |
| *Cdh2* | 0.548 | 0.002 | 0.309 | 0.097 | 0.322 | 0.083 | -0.294 | 0.114 | 0.213 | 0.258 | 0.357 | 0.053 |
| *Cdnf* | -0.239 | 0.203 | -0.364 | 0.048 | -0.398 | 0.029 | 0.245 | 0.192 | -0.012 | 0.949 | -0.052 | 0.785 |
| *Cercam* | 0.625 | <0.001 | 0.495 | 0.005 | 0.432 | 0.017 | -0.478 | 0.008 | 0.174 | 0.359 | 0.504 | 0.005 |
| *Ces2g* | 0.680 | <0.001 | 0.552 | 0.002 | 0.502 | 0.005 | -0.491 | 0.006 | 0.155 | 0.413 | 0.513 | 0.004 |
| *Chrnb2* | -0.728 | <0.001 | -0.401 | 0.028 | -0.371 | 0.044 | 0.204 | 0.280 | 0.180 | 0.342 | -0.550 | 0.002 |
| *Col15a1* | 0.728 | <0.001 | 0.550 | 0.002 | 0.593 | 0.001 | -0.468 | 0.009 | 0.181 | 0.338 | 0.467 | 0.009 |
| *Col18a1* | 0.748 | <0.001 | 0.507 | 0.004 | 0.573 | 0.001 | -0.530 | 0.003 | 0.244 | 0.194 | 0.523 | 0.003 |
| *Col6a2* | 0.663 | <0.001 | 0.462 | 0.010 | 0.509 | 0.004 | -0.377 | 0.040 | 0.244 | 0.195 | 0.441 | 0.015 |
| *Col6a5* | 0.621 | <0.001 | 0.282 | 0.131 | 0.412 | 0.024 | -0.362 | 0.050 | 0.274 | 0.143 | 0.482 | 0.007 |
| *Col8a1* | 0.559 | 0.001 | 0.388 | 0.034 | 0.388 | 0.034 | -0.318 | 0.087 | 0.175 | 0.355 | 0.483 | 0.007 |
| *Crispld2* | 0.525 | 0.003 | 0.362 | 0.049 | 0.400 | 0.028 | -0.209 | 0.269 | 0.217 | 0.250 | 0.434 | 0.016 |
| *Csf1* | 0.649 | <0.001 | 0.386 | 0.035 | 0.510 | 0.004 | -0.517 | 0.003 | 0.346 | 0.061 | 0.447 | 0.013 |
| *Cst11* | -0.162 | 0.394 | -0.189 | 0.318 | -0.163 | 0.389 | 0.405 | 0.026 | -0.199 | 0.292 | 0.140 | 0.460 |
| *Cst12* | -0.175 | 0.356 | -0.179 | 0.343 | -0.154 | 0.417 | 0.406 | 0.026 | -0.171 | 0.366 | 0.117 | 0.537 |
| *Cst8* | -0.163 | 0.389 | -0.175 | 0.354 | -0.171 | 0.368 | 0.408 | 0.025 | -0.200 | 0.289 | 0.135 | 0.478 |
| *Cyp2c44* | 0.377 | 0.040 | 0.231 | 0.219 | 0.287 | 0.124 | -0.221 | 0.241 | 0.085 | 0.654 | 0.265 | 0.156 |
| *D730048I06Rik* | -0.113 | 0.553 | -0.223 | 0.236 | -0.179 | 0.345 | 0.364 | 0.048 | -0.211 | 0.264 | 0.216 | 0.252 |
| *Ddo* | -0.441 | 0.015 | -0.281 | 0.133 | -0.358 | 0.052 | 0.231 | 0.220 | -0.013 | 0.944 | -0.491 | 0.006 |
| *Defb20* | -0.177 | 0.348 | -0.197 | 0.296 | -0.138 | 0.467 | 0.407 | 0.026 | -0.166 | 0.381 | 0.115 | 0.547 |
| *Defb25* | -0.160 | 0.399 | -0.185 | 0.327 | -0.168 | 0.374 | 0.404 | 0.027 | -0.201 | 0.286 | 0.143 | 0.451 |
| *Defb47* | -0.247 | 0.189 | -0.151 | 0.425 | -0.064 | 0.735 | 0.426 | 0.019 | -0.056 | 0.770 | -0.037 | 0.846 |
| *Defb48* | -0.136 | 0.472 | -0.187 | 0.323 | -0.191 | 0.313 | 0.394 | 0.031 | -0.237 | 0.207 | 0.180 | 0.342 |
| *Ecm2* | 0.560 | 0.001 | 0.403 | 0.027 | 0.400 | 0.029 | -0.363 | 0.049 | 0.210 | 0.265 | 0.430 | 0.018 |
| *Egfr* | 0.653 | <0.001 | 0.358 | 0.052 | 0.540 | 0.002 | -0.393 | 0.031 | 0.325 | 0.079 | 0.405 | 0.027 |
| *Eln* | 0.476 | 0.008 | 0.384 | 0.036 | 0.448 | 0.013 | -0.351 | 0.057 | 0.351 | 0.057 | 0.354 | 0.055 |
| *Ephb2* | 0.695 | <0.001 | 0.545 | 0.002 | 0.628 | <0.001 | -0.428 | 0.018 | 0.248 | 0.186 | 0.476 | 0.008 |
| *F10* | 0.406 | 0.026 | 0.229 | 0.223 | 0.302 | 0.105 | -0.240 | 0.202 | 0.137 | 0.471 | 0.249 | 0.185 |
| *F5* | 0.487 | 0.006 | 0.240 | 0.201 | 0.314 | 0.091 | -0.265 | 0.158 | 0.149 | 0.432 | 0.338 | 0.068 |
| *Fam150b* | 0.496 | 0.005 | 0.388 | 0.034 | 0.514 | 0.004 | -0.342 | 0.065 | 0.378 | 0.040 | 0.247 | 0.188 |
| *Fbln5* | 0.626 | <0.001 | 0.489 | 0.006 | 0.506 | 0.004 | -0.442 | 0.014 | 0.211 | 0.263 | 0.441 | 0.015 |
| *Fbn1* | 0.622 | <0.001 | 0.465 | 0.010 | 0.514 | 0.004 | -0.398 | 0.029 | 0.323 | 0.082 | 0.418 | 0.021 |
| *Fgfr1* | 0.642 | <0.001 | 0.507 | 0.004 | 0.528 | 0.003 | -0.487 | 0.006 | 0.309 | 0.097 | 0.441 | 0.015 |
| *Gm15386* | -0.132 | 0.487 | -0.187 | 0.323 | -0.194 | 0.303 | 0.391 | 0.033 | -0.232 | 0.218 | 0.186 | 0.326 |
| *Gm17727* | -0.101 | 0.595 | -0.223 | 0.237 | -0.190 | 0.316 | 0.355 | 0.054 | -0.233 | 0.215 | 0.236 | 0.209 |
| *Gm4788* | 0.410 | 0.025 | 0.268 | 0.152 | 0.304 | 0.103 | -0.257 | 0.170 | 0.094 | 0.620 | 0.271 | 0.147 |
| *Gm7120* | -0.540 | 0.002 | -0.391 | 0.033 | -0.437 | 0.016 | 0.435 | 0.016 | -0.288 | 0.122 | -0.207 | 0.272 |
| *Gpx5* | -0.208 | 0.270 | -0.185 | 0.327 | -0.102 | 0.592 | 0.417 | 0.022 | -0.106 | 0.577 | 0.049 | 0.795 |
| *H2-Ab1* | 0.636 | <0.001 | 0.428 | 0.018 | 0.348 | 0.059 | -0.430 | 0.018 | 0.111 | 0.559 | 0.582 | 0.001 |
| *Hpx* | 0.379 | 0.039 | 0.219 | 0.246 | 0.287 | 0.124 | -0.209 | 0.267 | 0.091 | 0.631 | 0.274 | 0.143 |
| *Htra1* | 0.620 | <0.001 | 0.516 | 0.004 | 0.449 | 0.013 | -0.588 | 0.001 | 0.127 | 0.503 | 0.495 | 0.005 |
| *Icam2* | 0.581 | 0.001 | 0.456 | 0.011 | 0.352 | 0.056 | -0.422 | 0.020 | 0.084 | 0.659 | 0.474 | 0.008 |
| *Igsf10* | 0.627 | <0.001 | 0.378 | 0.040 | 0.434 | 0.017 | -0.340 | 0.066 | 0.309 | 0.097 | 0.393 | 0.032 |
| *Il18r1* | 0.472 | 0.008 | 0.286 | 0.126 | 0.228 | 0.226 | -0.254 | 0.176 | -0.014 | 0.942 | 0.456 | 0.011 |
| *Il1rap* | 0.572 | 0.001 | 0.282 | 0.131 | 0.352 | 0.056 | -0.268 | 0.152 | 0.125 | 0.510 | 0.400 | 0.028 |
| *Il27ra* | 0.578 | 0.001 | 0.530 | 0.003 | 0.372 | 0.043 | -0.360 | 0.051 | 0.081 | 0.671 | 0.471 | 0.009 |
| *Il2rg* | 0.643 | <0.001 | 0.569 | 0.001 | 0.422 | 0.020 | -0.513 | 0.004 | 0.183 | 0.332 | 0.481 | 0.007 |
| *Itgb5* | 0.642 | <0.001 | 0.605 | <0.001 | 0.537 | 0.002 | -0.416 | 0.022 | 0.218 | 0.247 | 0.481 | 0.007 |
| *Itgbl1* | 0.447 | 0.013 | 0.305 | 0.101 | 0.256 | 0.172 | -0.085 | 0.656 | 0.038 | 0.844 | 0.450 | 0.013 |
| *Itih2* | 0.383 | 0.037 | 0.219 | 0.246 | 0.291 | 0.119 | -0.207 | 0.273 | 0.097 | 0.612 | 0.288 | 0.122 |
| *Itih3* | 0.386 | 0.035 | 0.213 | 0.259 | 0.302 | 0.105 | -0.220 | 0.243 | 0.099 | 0.603 | 0.278 | 0.136 |
| *Kirrel* | 0.636 | <0.001 | 0.579 | 0.001 | 0.491 | 0.006 | -0.526 | 0.003 | 0.231 | 0.219 | 0.420 | 0.021 |
| *Kng1* | 0.383 | 0.037 | 0.223 | 0.236 | 0.294 | 0.115 | -0.214 | 0.256 | 0.087 | 0.648 | 0.272 | 0.146 |
| *Lamb2* | 0.659 | <0.001 | 0.453 | 0.012 | 0.516 | 0.004 | -0.415 | 0.022 | 0.333 | 0.072 | 0.451 | 0.012 |
| *Lbp* | 0.713 | <0.001 | 0.477 | 0.008 | 0.566 | 0.001 | -0.389 | 0.034 | 0.224 | 0.233 | 0.515 | 0.004 |
| *Lcat* | 0.391 | 0.033 | 0.240 | 0.201 | 0.277 | 0.138 | -0.220 | 0.244 | 0.104 | 0.584 | 0.283 | 0.130 |
| *Lcn5* | -0.114 | 0.547 | -0.197 | 0.296 | -0.168 | 0.374 | 0.378 | 0.039 | -0.140 | 0.461 | 0.166 | 0.380 |
| *Lcn8* | -0.161 | 0.397 | -0.175 | 0.355 | -0.175 | 0.356 | 0.411 | 0.024 | -0.214 | 0.256 | 0.138 | 0.467 |
| *Lcn9* | -0.183 | 0.333 | -0.186 | 0.324 | -0.140 | 0.462 | 0.415 | 0.023 | -0.170 | 0.370 | 0.102 | 0.591 |
| *Ldlrad3* | -0.552 | 0.002 | -0.528 | 0.003 | -0.361 | 0.050 | 0.255 | 0.173 | -0.064 | 0.737 | -0.295 | 0.113 |
| *Lfng* | 0.677 | <0.001 | 0.499 | 0.005 | 0.519 | 0.003 | -0.498 | 0.005 | 0.297 | 0.111 | 0.484 | 0.007 |
| *Lipg* | -0.187 | 0.323 | -0.215 | 0.255 | -0.212 | 0.262 | 0.405 | 0.026 | -0.102 | 0.591 | 0.054 | 0.777 |
| *Lrtm1* | -0.631 | <0.001 | -0.402 | 0.028 | -0.344 | 0.063 | 0.080 | 0.672 | 0.115 | 0.546 | -0.733 | <0.001 |
| *Ltbp3* | 0.655 | <0.001 | 0.554 | 0.001 | 0.552 | 0.002 | -0.423 | 0.020 | 0.308 | 0.097 | 0.484 | 0.007 |
| *Ly6g5b* | -0.173 | 0.359 | -0.179 | 0.344 | -0.156 | 0.410 | 0.412 | 0.024 | -0.184 | 0.329 | 0.117 | 0.539 |
| *Mcam* | 0.626 | <0.001 | 0.491 | 0.006 | 0.434 | 0.017 | -0.430 | 0.018 | 0.156 | 0.410 | 0.442 | 0.014 |
| *Mpzl2* | -0.778 | <0.001 | -0.455 | 0.011 | -0.473 | 0.008 | 0.536 | 0.002 | -0.115 | 0.547 | -0.478 | 0.008 |
| *Muc5b* | -0.082 | 0.668 | -0.140 | 0.461 | -0.181 | 0.337 | 0.360 | 0.051 | -0.091 | 0.631 | 0.135 | 0.477 |
| *Mup20* | 0.379 | 0.039 | 0.221 | 0.240 | 0.290 | 0.120 | -0.213 | 0.259 | 0.093 | 0.624 | 0.271 | 0.148 |
| *Mup3* | 0.378 | 0.039 | 0.220 | 0.242 | 0.289 | 0.121 | -0.214 | 0.255 | 0.094 | 0.621 | 0.270 | 0.150 |
| *Mxra7* | 0.581 | 0.001 | 0.372 | 0.043 | 0.363 | 0.049 | -0.315 | 0.090 | 0.062 | 0.747 | 0.560 | 0.001 |
| *Mxra8* | 0.608 | <0.001 | 0.482 | 0.007 | 0.442 | 0.014 | -0.383 | 0.037 | 0.192 | 0.310 | 0.467 | 0.009 |
| *Nid2* | 0.661 | <0.001 | 0.477 | 0.008 | 0.517 | 0.003 | -0.532 | 0.002 | 0.199 | 0.292 | 0.354 | 0.055 |
| *Nov* | 0.424 | 0.020 | 0.324 | 0.081 | 0.334 | 0.071 | -0.256 | 0.173 | 0.142 | 0.453 | 0.299 | 0.109 |
| *Npdc1* | 0.658 | <0.001 | 0.523 | 0.003 | 0.557 | 0.001 | -0.469 | 0.009 | 0.252 | 0.180 | 0.562 | 0.001 |
| *Npr3* | 0.611 | <0.001 | 0.502 | 0.005 | 0.490 | 0.006 | -0.422 | 0.020 | -0.011 | 0.955 | 0.412 | 0.024 |
| *Ntn4* | 0.588 | 0.001 | 0.398 | 0.029 | 0.452 | 0.012 | -0.448 | 0.013 | 0.275 | 0.141 | 0.494 | 0.006 |
| *Ogn* | 0.500 | 0.005 | 0.345 | 0.062 | 0.270 | 0.149 | -0.158 | 0.405 | 0.038 | 0.841 | 0.471 | 0.009 |
| *Ovch2* | -0.167 | 0.377 | -0.156 | 0.412 | -0.181 | 0.339 | 0.420 | 0.021 | -0.212 | 0.260 | 0.120 | 0.528 |
| *Parm1* | -0.426 | 0.019 | -0.252 | 0.179 | -0.227 | 0.228 | 0.059 | 0.759 | -0.010 | 0.959 | -0.468 | 0.009 |
| *Pcdh1* | 0.622 | <0.001 | 0.377 | 0.040 | 0.552 | 0.002 | -0.447 | 0.013 | 0.338 | 0.068 | 0.299 | 0.109 |
| *Pcdh12* | 0.697 | <0.001 | 0.439 | 0.015 | 0.524 | 0.003 | -0.392 | 0.032 | 0.165 | 0.382 | 0.463 | 0.010 |
| *Pcdh19* | 0.644 | <0.001 | 0.446 | 0.014 | 0.568 | 0.001 | -0.529 | 0.003 | 0.177 | 0.349 | 0.446 | 0.013 |
| *Pcsk4* | -0.477 | 0.008 | -0.211 | 0.264 | -0.337 | 0.069 | 0.111 | 0.558 | -0.159 | 0.403 | -0.400 | 0.029 |
| *Pla2g12a* | -0.609 | <0.001 | -0.336 | 0.069 | -0.390 | 0.033 | 0.409 | 0.025 | -0.267 | 0.153 | -0.240 | 0.202 |
| *Plxna1* | 0.669 | <0.001 | 0.512 | 0.004 | 0.599 | <0.001 | -0.512 | 0.004 | 0.315 | 0.090 | 0.392 | 0.032 |
| *Plxnd1* | 0.691 | <0.001 | 0.541 | 0.002 | 0.565 | 0.001 | -0.534 | 0.002 | 0.262 | 0.161 | 0.427 | 0.019 |
| *Pon1* | 0.482 | 0.007 | 0.216 | 0.252 | 0.259 | 0.167 | -0.225 | 0.232 | 0.056 | 0.768 | 0.357 | 0.053 |
| *Prelp* | 0.640 | <0.001 | 0.555 | 0.001 | 0.463 | 0.010 | -0.456 | 0.011 | 0.040 | 0.835 | 0.520 | 0.003 |
| *Proz* | 0.403 | 0.027 | 0.201 | 0.286 | 0.292 | 0.117 | -0.247 | 0.189 | 0.092 | 0.628 | 0.296 | 0.112 |
| *Pth1r* | -0.512 | 0.004 | -0.268 | 0.152 | -0.164 | 0.385 | 0.102 | 0.592 | -0.025 | 0.896 | -0.408 | 0.025 |
| *Pvr* | 0.655 | <0.001 | 0.514 | 0.004 | 0.352 | 0.057 | -0.365 | 0.047 | 0.050 | 0.793 | 0.460 | 0.011 |
| *Pxdn* | 0.543 | 0.002 | 0.518 | 0.003 | 0.455 | 0.011 | -0.440 | 0.015 | 0.161 | 0.396 | 0.285 | 0.126 |
| *Pzp* | 0.376 | 0.040 | 0.218 | 0.247 | 0.286 | 0.125 | -0.209 | 0.267 | 0.092 | 0.629 | 0.272 | 0.146 |
| *Rarres2* | 0.658 | <0.001 | 0.570 | 0.001 | 0.490 | 0.006 | -0.390 | 0.033 | 0.016 | 0.931 | 0.535 | 0.002 |
| *Rdh11* | -0.500 | 0.005 | -0.255 | 0.174 | -0.123 | 0.517 | 0.242 | 0.198 | -0.090 | 0.637 | -0.377 | 0.040 |
| *Rdh7* | 0.383 | 0.037 | 0.225 | 0.232 | 0.293 | 0.116 | -0.212 | 0.260 | 0.092 | 0.628 | 0.274 | 0.143 |
| *Rnase13* | -0.108 | 0.570 | -0.177 | 0.350 | -0.176 | 0.352 | 0.378 | 0.039 | -0.126 | 0.508 | 0.155 | 0.415 |
| *Ros1* | -0.215 | 0.254 | -0.138 | 0.466 | -0.115 | 0.546 | 0.439 | 0.015 | -0.082 | 0.668 | -0.001 | 0.996 |
| *Rtn4rl1* | 0.675 | <0.001 | 0.546 | 0.002 | 0.519 | 0.003 | -0.523 | 0.003 | 0.112 | 0.555 | 0.517 | 0.003 |
| *Sema4c* | 0.668 | <0.001 | 0.432 | 0.017 | 0.477 | 0.008 | -0.365 | 0.047 | 0.281 | 0.132 | 0.482 | 0.007 |
| *Serpina1a* | 0.376 | 0.040 | 0.221 | 0.241 | 0.289 | 0.121 | -0.212 | 0.260 | 0.095 | 0.617 | 0.269 | 0.151 |
| *Serpina1b* | 0.382 | 0.037 | 0.220 | 0.242 | 0.291 | 0.119 | -0.214 | 0.257 | 0.093 | 0.624 | 0.275 | 0.141 |
| *Serpina1c* | 0.377 | 0.040 | 0.219 | 0.244 | 0.286 | 0.125 | -0.212 | 0.261 | 0.096 | 0.613 | 0.271 | 0.147 |
| *Serpina1d* | 0.382 | 0.037 | 0.223 | 0.235 | 0.293 | 0.116 | -0.214 | 0.256 | 0.093 | 0.626 | 0.272 | 0.146 |
| *Serpina1e* | 0.374 | 0.042 | 0.217 | 0.249 | 0.284 | 0.128 | -0.210 | 0.264 | 0.099 | 0.602 | 0.269 | 0.150 |
| *Serpinc1* | 0.381 | 0.038 | 0.221 | 0.240 | 0.291 | 0.118 | -0.213 | 0.259 | 0.090 | 0.638 | 0.273 | 0.144 |
| *Serpinf1* | 0.652 | <0.001 | 0.453 | 0.012 | 0.498 | 0.005 | -0.312 | 0.093 | 0.223 | 0.236 | 0.516 | 0.004 |
| *Sfrp5* | 0.439 | 0.015 | 0.496 | 0.005 | 0.470 | 0.009 | -0.348 | 0.059 | -0.202 | 0.285 | 0.294 | 0.115 |
| *Slc25a16* | -0.404 | 0.027 | -0.411 | 0.024 | -0.223 | 0.236 | -0.070 | 0.712 | 0.010 | 0.958 | -0.433 | 0.017 |
| *Slc27a1* | -0.630 | <0.001 | -0.326 | 0.079 | -0.402 | 0.027 | 0.150 | 0.430 | -0.039 | 0.839 | -0.604 | <0.001 |
| *Slc9a2* | -0.100 | 0.600 | -0.251 | 0.182 | -0.106 | 0.577 | 0.242 | 0.197 | -0.128 | 0.501 | 0.108 | 0.569 |
| *Smo* | 0.535 | 0.002 | 0.481 | 0.007 | 0.346 | 0.061 | -0.397 | 0.030 | 0.117 | 0.538 | 0.539 | 0.002 |
| *Smoc2* | 0.559 | 0.001 | 0.445 | 0.014 | 0.416 | 0.022 | -0.202 | 0.285 | 0.213 | 0.257 | 0.486 | 0.006 |
| *Sorl1* | -0.435 | 0.016 | -0.284 | 0.128 | -0.249 | 0.185 | 0.038 | 0.840 | 0.011 | 0.952 | -0.585 | 0.001 |
| *Spag11b* | -0.231 | 0.220 | -0.170 | 0.368 | -0.078 | 0.680 | 0.419 | 0.021 | -0.075 | 0.693 | 0.004 | 0.984 |
| *Spink12* | -0.127 | 0.502 | -0.203 | 0.283 | -0.157 | 0.409 | 0.383 | 0.037 | -0.134 | 0.482 | 0.154 | 0.415 |
| *Spink2* | -0.093 | 0.627 | -0.190 | 0.315 | -0.200 | 0.289 | 0.373 | 0.042 | -0.180 | 0.340 | 0.204 | 0.281 |
| *Spink5* | -0.145 | 0.446 | -0.211 | 0.264 | -0.141 | 0.458 | 0.388 | 0.034 | -0.131 | 0.492 | 0.140 | 0.461 |
| *Spock1* | -0.186 | 0.326 | -0.164 | 0.387 | -0.128 | 0.500 | 0.415 | 0.023 | -0.092 | 0.630 | 0.048 | 0.801 |
| *Taok3* | 0.688 | <0.001 | 0.464 | 0.010 | 0.514 | 0.004 | -0.560 | 0.001 | 0.245 | 0.192 | 0.512 | 0.004 |
| *Tfpi* | 0.604 | <0.001 | 0.364 | 0.048 | 0.390 | 0.033 | -0.358 | 0.052 | 0.283 | 0.130 | 0.443 | 0.014 |
| *Tgfb3* | 0.540 | 0.002 | 0.442 | 0.014 | 0.352 | 0.057 | -0.259 | 0.167 | 0.089 | 0.641 | 0.512 | 0.004 |
| *Thbd* | 0.570 | 0.001 | 0.473 | 0.008 | 0.427 | 0.019 | -0.581 | 0.001 | 0.192 | 0.310 | 0.385 | 0.036 |
| *Tril* | 0.536 | 0.002 | 0.486 | 0.007 | 0.481 | 0.007 | -0.434 | 0.017 | 0.304 | 0.102 | 0.415 | 0.023 |
| *Ugt3a2* | 0.400 | 0.029 | 0.245 | 0.193 | 0.316 | 0.089 | -0.237 | 0.208 | 0.100 | 0.600 | 0.293 | 0.116 |
| *Unc5a* | -0.369 | 0.045 | -0.164 | 0.386 | -0.128 | 0.501 | 0.104 | 0.585 | -0.063 | 0.741 | -0.281 | 0.133 |
| *Vasn* | 0.648 | <0.001 | 0.537 | 0.002 | 0.502 | 0.005 | -0.402 | 0.027 | 0.199 | 0.292 | 0.447 | 0.013 |
| *Vegfc* | 0.586 | 0.001 | 0.368 | 0.045 | 0.424 | 0.020 | -0.283 | 0.130 | 0.270 | 0.149 | 0.495 | 0.005 |
| *Vtn* | 0.421 | 0.020 | 0.246 | 0.190 | 0.332 | 0.073 | -0.257 | 0.170 | 0.137 | 0.472 | 0.275 | 0.141 |

Supplementary Table 12. Expression levels of signaling genes correlated with glucose homeostasis measurements. Glucose endpoint refers to the glucose levels measured at the end of the study. Fasting glucose overnight refers to the glucose levels measured after fasting for a night before the glucose tolerance test was performed (GTT). AUC-GTT refers to the area under the curve analysis performed for the GTT (Methods see (*19*)).

|  | **Glucose**  **endpoint** |  | **Insulin sensitivity** |  | **Insulin resistance** |  | **Fasting glucose**  **overnight** |  | **AUC GTT** |  |
| --- | --- | --- | --- | --- | --- | --- | --- | --- | --- | --- |
|  | **r** | **p-value** | **r** | **p-value** | **r** | **p-value** | **r** | **p-value** | **r** | **p-value** |
| *9230104L09Rik* | -0.160 | 0.443 | 0.015 | 0.942 | -0.123 | 0.558 | 0.011 | 0.957 | -0.210 | 0.315 |
| *9230110F15Rik* | -0.198 | 0.342 | 0.014 | 0.946 | -0.124 | 0.554 | -0.033 | 0.875 | -0.234 | 0.260 |
| *Acvrl1* | 0.610 | 0.001 | -0.432 | 0.031 | 0.486 | 0.014 | 0.599 | 0.002 | 0.699 | <0.001 |
| *Adam15* | 0.653 | <0.001 | -0.462 | 0.020 | 0.479 | 0.015 | 0.636 | 0.001 | 0.680 | <0.001 |
| *Adam28* | -0.178 | 0.394 | 0.043 | 0.839 | -0.147 | 0.483 | 0.013 | 0.952 | -0.204 | 0.328 |
| *Adamts16* | 0.380 | 0.061 | -0.376 | 0.064 | 0.494 | 0.012 | 0.472 | 0.017 | 0.618 | 0.001 |
| *Adamts5* | 0.719 | <0.001 | -0.517 | 0.008 | 0.669 | <0.001 | 0.713 | <0.001 | 0.801 | <0.001 |
| *Adamtsl4* | 0.650 | <0.001 | -0.334 | 0.103 | 0.382 | 0.060 | 0.639 | 0.001 | 0.623 | 0.001 |
| *Ahsg* | 0.223 | 0.283 | -0.174 | 0.405 | 0.177 | 0.398 | 0.198 | 0.342 | 0.428 | 0.033 |
| *AI182371* | 0.217 | 0.297 | -0.159 | 0.447 | 0.161 | 0.442 | 0.197 | 0.344 | 0.424 | 0.035 |
| *Alb* | 0.230 | 0.269 | -0.176 | 0.401 | 0.179 | 0.392 | 0.204 | 0.329 | 0.433 | 0.031 |
| *Ambp* | 0.220 | 0.291 | -0.173 | 0.408 | 0.178 | 0.395 | 0.195 | 0.351 | 0.425 | 0.034 |
| *Angptl3* | 0.239 | 0.250 | -0.186 | 0.374 | 0.195 | 0.351 | 0.208 | 0.318 | 0.441 | 0.027 |
| *Antxr2* | 0.685 | <0.001 | -0.390 | 0.054 | 0.453 | 0.023 | 0.655 | <0.001 | 0.673 | <0.001 |
| *Apln* | 0.539 | 0.005 | -0.583 | 0.002 | 0.733 | <0.001 | 0.553 | 0.004 | 0.799 | <0.001 |
| *Apoa2* | 0.222 | 0.287 | -0.171 | 0.413 | 0.174 | 0.406 | 0.197 | 0.344 | 0.426 | 0.034 |
| *Apoe* | 0.615 | 0.001 | -0.373 | 0.066 | 0.472 | 0.017 | 0.478 | 0.016 | 0.641 | 0.001 |
| *Apoh* | 0.223 | 0.284 | -0.176 | 0.400 | 0.178 | 0.394 | 0.198 | 0.343 | 0.427 | 0.033 |
| *Azgp1* | 0.173 | 0.409 | -0.168 | 0.423 | 0.151 | 0.471 | 0.169 | 0.419 | 0.403 | 0.046 |
| *Bace2* | 0.690 | <0.001 | -0.453 | 0.023 | 0.540 | 0.005 | 0.733 | <0.001 | 0.777 | <0.001 |
| *C4b* | 0.613 | 0.001 | -0.385 | 0.057 | 0.426 | 0.034 | 0.566 | 0.003 | 0.570 | 0.003 |
| *C6* | 0.678 | <0.001 | -0.466 | 0.019 | 0.509 | 0.009 | 0.561 | 0.004 | 0.566 | 0.003 |
| *C7* | 0.520 | 0.008 | -0.328 | 0.110 | 0.318 | 0.122 | 0.527 | 0.007 | 0.538 | 0.006 |
| *Cd248* | 0.571 | 0.003 | -0.497 | 0.012 | 0.531 | 0.006 | 0.610 | 0.001 | 0.648 | <0.001 |
| *Cd79a* | 0.559 | 0.004 | -0.418 | 0.038 | 0.600 | 0.002 | 0.468 | 0.018 | 0.709 | <0.001 |
| *Cdh11* | 0.282 | 0.171 | -0.420 | 0.036 | 0.562 | 0.003 | 0.308 | 0.135 | 0.517 | 0.008 |
| *Cdh2* | 0.388 | 0.055 | -0.460 | 0.021 | 0.560 | 0.004 | 0.384 | 0.058 | 0.590 | 0.002 |
| *Cdnf* | -0.406 | 0.044 | 0.404 | 0.045 | -0.558 | 0.004 | -0.404 | 0.045 | -0.542 | 0.005 |
| *Cercam* | 0.499 | 0.011 | -0.533 | 0.006 | 0.585 | 0.002 | 0.518 | 0.008 | 0.599 | 0.002 |
| *Ces2g* | 0.601 | 0.001 | -0.418 | 0.038 | 0.506 | 0.010 | 0.641 | 0.001 | 0.663 | <0.001 |
| *Chrnb2* | -0.516 | 0.008 | 0.562 | 0.003 | -0.634 | 0.001 | -0.476 | 0.016 | -0.662 | <0.001 |
| *Col15a1* | 0.639 | 0.001 | -0.485 | 0.014 | 0.537 | 0.006 | 0.585 | 0.002 | 0.674 | <0.001 |
| *Col18a1* | 0.648 | <0.001 | -0.511 | 0.009 | 0.531 | 0.006 | 0.654 | <0.001 | 0.763 | <0.001 |
| *Col6a2* | 0.494 | 0.012 | -0.496 | 0.012 | 0.594 | 0.002 | 0.496 | 0.012 | 0.709 | <0.001 |
| *Col6a5* | 0.515 | 0.008 | -0.338 | 0.098 | 0.322 | 0.116 | 0.535 | 0.006 | 0.500 | 0.011 |
| *Col8a1* | 0.362 | 0.076 | -0.494 | 0.012 | 0.554 | 0.004 | 0.443 | 0.027 | 0.573 | 0.003 |
| *Crispld2* | 0.367 | 0.072 | -0.463 | 0.020 | 0.572 | 0.003 | 0.430 | 0.032 | 0.662 | <0.001 |
| *Csf1* | 0.529 | 0.007 | -0.418 | 0.038 | 0.486 | 0.014 | 0.561 | 0.003 | 0.713 | <0.001 |
| *Cst11* | -0.174 | 0.405 | 0.014 | 0.948 | -0.123 | 0.559 | -0.003 | 0.990 | -0.217 | 0.298 |
| *Cst12* | -0.158 | 0.449 | -0.002 | 0.991 | -0.107 | 0.610 | -0.002 | 0.993 | -0.218 | 0.294 |
| *Cst8* | -0.166 | 0.428 | 0.016 | 0.940 | -0.123 | 0.557 | 0.004 | 0.986 | -0.214 | 0.304 |
| *Cyp2c44* | 0.224 | 0.281 | -0.176 | 0.399 | 0.183 | 0.381 | 0.198 | 0.342 | 0.423 | 0.035 |
| *D730048I06Rik* | -0.094 | 0.654 | -0.006 | 0.979 | -0.104 | 0.620 | 0.091 | 0.664 | -0.168 | 0.423 |
| *Ddo* | -0.777 | <0.001 | 0.506 | 0.010 | -0.533 | 0.006 | -0.827 | <0.001 | -0.681 | <0.001 |
| *Defb20* | -0.188 | 0.369 | -0.003 | 0.990 | -0.110 | 0.602 | -0.029 | 0.890 | -0.232 | 0.265 |
| *Defb25* | -0.164 | 0.432 | 0.013 | 0.949 | -0.122 | 0.562 | 0.007 | 0.972 | -0.212 | 0.310 |
| *Defb47* | -0.271 | 0.191 | -0.031 | 0.885 | -0.082 | 0.695 | -0.170 | 0.416 | -0.302 | 0.143 |
| *Defb48* | -0.150 | 0.475 | 0.030 | 0.886 | -0.135 | 0.521 | 0.038 | 0.857 | -0.192 | 0.359 |
| *Ecm2* | 0.598 | 0.002 | -0.379 | 0.062 | 0.487 | 0.014 | 0.571 | 0.003 | 0.611 | 0.001 |
| *Egfr* | 0.585 | 0.002 | -0.374 | 0.066 | 0.406 | 0.044 | 0.548 | 0.005 | 0.687 | <0.001 |
| *Eln* | 0.287 | 0.164 | -0.324 | 0.114 | 0.413 | 0.040 | 0.396 | 0.050 | 0.615 | 0.001 |
| *Ephb2* | 0.433 | 0.030 | -0.436 | 0.029 | 0.484 | 0.014 | 0.490 | 0.013 | 0.680 | <0.001 |
| *F10* | 0.240 | 0.249 | -0.173 | 0.407 | 0.177 | 0.398 | 0.207 | 0.322 | 0.444 | 0.026 |
| *F5* | 0.385 | 0.058 | -0.204 | 0.328 | 0.203 | 0.330 | 0.314 | 0.127 | 0.439 | 0.028 |
| *Fam150b* | 0.585 | 0.002 | -0.380 | 0.061 | 0.454 | 0.023 | 0.563 | 0.003 | 0.642 | 0.001 |
| *Fbln5* | 0.607 | 0.001 | -0.467 | 0.018 | 0.541 | 0.005 | 0.561 | 0.004 | 0.654 | <0.001 |
| *Fbn1* | 0.484 | 0.014 | -0.395 | 0.051 | 0.414 | 0.040 | 0.542 | 0.005 | 0.608 | 0.001 |
| *Fgfr1* | 0.635 | 0.001 | -0.423 | 0.035 | 0.476 | 0.016 | 0.596 | 0.002 | 0.666 | <0.001 |
| *Gm15386* | -0.130 | 0.537 | 0.022 | 0.917 | -0.128 | 0.542 | 0.057 | 0.788 | -0.186 | 0.374 |
| *Gm17727* | -0.081 | 0.699 | 0.007 | 0.974 | -0.112 | 0.594 | 0.110 | 0.602 | -0.150 | 0.473 |
| *Gm4788* | 0.251 | 0.226 | -0.199 | 0.340 | 0.222 | 0.286 | 0.234 | 0.259 | 0.455 | 0.022 |
| *Gm7120* | -0.234 | 0.260 | 0.263 | 0.204 | -0.320 | 0.119 | -0.163 | 0.435 | -0.397 | 0.050 |
| *Gpx5* | -0.218 | 0.294 | -0.027 | 0.896 | -0.089 | 0.671 | -0.085 | 0.686 | -0.266 | 0.199 |
| *H2.Ab1* | 0.533 | 0.006 | -0.457 | 0.022 | 0.545 | 0.005 | 0.540 | 0.005 | 0.681 | <0.001 |
| *Hpx* | 0.222 | 0.286 | -0.171 | 0.414 | 0.174 | 0.407 | 0.197 | 0.346 | 0.425 | 0.034 |
| *Htra1* | 0.637 | 0.001 | -0.540 | 0.005 | 0.642 | 0.001 | 0.613 | 0.001 | 0.677 | <0.001 |
| *Icam2* | 0.627 | 0.001 | -0.373 | 0.066 | 0.514 | 0.009 | 0.567 | 0.003 | 0.755 | <0.001 |
| *Igsf10* | 0.482 | 0.015 | -0.374 | 0.066 | 0.367 | 0.071 | 0.489 | 0.013 | 0.539 | 0.005 |
| *Il18r1* | 0.361 | 0.076 | -0.408 | 0.043 | 0.491 | 0.013 | 0.326 | 0.111 | 0.442 | 0.027 |
| *Il1rap* | 0.351 | 0.086 | -0.364 | 0.074 | 0.434 | 0.030 | 0.311 | 0.130 | 0.599 | 0.002 |
| *Il27ra* | 0.539 | 0.005 | -0.401 | 0.047 | 0.577 | 0.003 | 0.519 | 0.008 | 0.668 | <0.001 |
| *Il2rg* | 0.543 | 0.005 | -0.452 | 0.023 | 0.520 | 0.008 | 0.597 | 0.002 | 0.662 | <0.001 |
| *Itgb5* | 0.535 | 0.006 | -0.551 | 0.004 | 0.615 | 0.001 | 0.470 | 0.018 | 0.631 | 0.001 |
| *Itgbl1* | 0.317 | 0.123 | -0.417 | 0.038 | 0.568 | 0.003 | 0.292 | 0.157 | 0.539 | 0.005 |
| *Itih2* | 0.218 | 0.295 | -0.177 | 0.398 | 0.174 | 0.404 | 0.202 | 0.333 | 0.427 | 0.033 |
| *Itih3* | 0.235 | 0.258 | -0.182 | 0.384 | 0.184 | 0.379 | 0.211 | 0.311 | 0.441 | 0.027 |
| *Kirrel* | 0.658 | <0.001 | -0.422 | 0.036 | 0.480 | 0.015 | 0.624 | 0.001 | 0.671 | <0.001 |
| *Kng1* | 0.227 | 0.275 | -0.178 | 0.394 | 0.183 | 0.381 | 0.200 | 0.337 | 0.430 | 0.032 |
| *Lamb2* | 0.606 | 0.001 | -0.434 | 0.030 | 0.417 | 0.038 | 0.590 | 0.002 | 0.619 | 0.001 |
| *Lbp* | 0.632 | 0.001 | -0.478 | 0.016 | 0.567 | 0.003 | 0.634 | 0.001 | 0.762 | <0.001 |
| *Lcat* | 0.235 | 0.259 | -0.173 | 0.409 | 0.178 | 0.395 | 0.221 | 0.288 | 0.443 | 0.026 |
| *Lcn5* | -0.101 | 0.630 | -0.052 | 0.805 | -0.074 | 0.725 | 0.070 | 0.739 | -0.211 | 0.312 |
| *Lcn8* | -0.184 | 0.379 | 0.027 | 0.899 | -0.134 | 0.523 | -0.008 | 0.971 | -0.219 | 0.293 |
| *Lcn9* | -0.206 | 0.323 | 0.006 | 0.979 | -0.117 | 0.578 | -0.047 | 0.824 | -0.241 | 0.246 |
| *Ldlrad3* | -0.618 | 0.001 | 0.403 | 0.046 | -0.490 | 0.013 | -0.507 | 0.010 | -0.635 | 0.001 |
| *Lfng* | 0.624 | 0.001 | -0.519 | 0.008 | 0.545 | 0.005 | 0.603 | 0.001 | 0.662 | <0.001 |
| *Lipg* | -0.101 | 0.633 | -0.029 | 0.889 | -0.094 | 0.653 | 0.015 | 0.945 | -0.266 | 0.199 |
| *Lrtm1* | -0.493 | 0.012 | 0.440 | 0.028 | -0.443 | 0.027 | -0.569 | 0.003 | -0.579 | 0.002 |
| *Ltbp3* | 0.531 | 0.006 | -0.451 | 0.024 | 0.514 | 0.009 | 0.520 | 0.008 | 0.668 | <0.001 |
| *Ly6g5b* | -0.185 | 0.377 | 0.010 | 0.963 | -0.120 | 0.568 | -0.021 | 0.921 | -0.228 | 0.272 |
| *Mcam* | 0.738 | <0.001 | -0.327 | 0.110 | 0.360 | 0.077 | 0.620 | 0.001 | 0.622 | 0.001 |
| *Mpzl2* | -0.609 | 0.001 | 0.396 | 0.050 | -0.443 | 0.026 | -0.557 | 0.004 | -0.636 | 0.001 |
| *Muc5b* | -0.072 | 0.733 | -0.082 | 0.698 | -0.049 | 0.815 | 0.088 | 0.676 | -0.220 | 0.291 |
| *Mup20* | 0.217 | 0.297 | -0.175 | 0.402 | 0.177 | 0.397 | 0.195 | 0.351 | 0.425 | 0.034 |
| *Mup3* | 0.218 | 0.295 | -0.173 | 0.407 | 0.174 | 0.404 | 0.196 | 0.349 | 0.425 | 0.034 |
| *Mxra7* | 0.435 | 0.030 | -0.512 | 0.009 | 0.614 | 0.001 | 0.446 | 0.026 | 0.619 | 0.001 |
| *Mxra8* | 0.508 | 0.010 | -0.502 | 0.010 | 0.616 | 0.001 | 0.514 | 0.009 | 0.676 | <0.001 |
| *Nid2* | 0.705 | <0.001 | -0.450 | 0.024 | 0.520 | 0.008 | 0.637 | 0.001 | 0.669 | <0.001 |
| *Nov* | 0.449 | 0.025 | -0.535 | 0.006 | 0.717 | <0.001 | 0.457 | 0.022 | 0.617 | 0.001 |
| *Npdc1* | 0.635 | 0.001 | -0.558 | 0.004 | 0.591 | 0.002 | 0.641 | 0.001 | 0.720 | <0.001 |
| *Npr3* | 0.746 | <0.001 | -0.446 | 0.026 | 0.526 | 0.007 | 0.640 | 0.001 | 0.639 | 0.001 |
| *Ntn4* | 0.546 | 0.005 | -0.437 | 0.029 | 0.488 | 0.013 | 0.538 | 0.005 | 0.658 | <0.001 |
| *Ogn* | 0.481 | 0.015 | -0.466 | 0.019 | 0.589 | 0.002 | 0.419 | 0.037 | 0.581 | 0.002 |
| *Ovch2* | -0.192 | 0.358 | 0.032 | 0.881 | -0.138 | 0.509 | -0.020 | 0.925 | -0.228 | 0.273 |
| *Parm1* | -0.578 | 0.002 | 0.319 | 0.120 | -0.387 | 0.056 | -0.608 | 0.001 | -0.504 | 0.010 |
| *Pcdh1* | 0.538 | 0.006 | -0.358 | 0.079 | 0.337 | 0.099 | 0.480 | 0.015 | 0.672 | <0.001 |
| *Pcdh12* | 0.540 | 0.005 | -0.390 | 0.054 | 0.450 | 0.024 | 0.475 | 0.016 | 0.717 | <0.001 |
| *Pcdh19* | 0.681 | <0.001 | -0.584 | 0.002 | 0.670 | <0.001 | 0.688 | <0.001 | 0.768 | <0.001 |
| *Pcsk4* | -0.526 | 0.007 | 0.427 | 0.033 | -0.430 | 0.032 | -0.634 | 0.001 | -0.496 | 0.012 |
| *Pla2g12a* | -0.258 | 0.214 | 0.152 | 0.467 | -0.226 | 0.278 | -0.194 | 0.353 | -0.415 | 0.039 |
| *Plxna1* | 0.558 | 0.004 | -0.429 | 0.032 | 0.456 | 0.022 | 0.552 | 0.004 | 0.648 | <0.001 |
| *Plxnd1* | 0.628 | 0.001 | -0.425 | 0.034 | 0.486 | 0.014 | 0.616 | 0.001 | 0.705 | <0.001 |
| *Pon1* | 0.415 | 0.039 | -0.293 | 0.155 | 0.319 | 0.120 | 0.328 | 0.110 | 0.487 | 0.014 |
| *Prelp* | 0.742 | <0.001 | -0.426 | 0.034 | 0.471 | 0.018 | 0.636 | 0.001 | 0.627 | 0.001 |
| *Proz* | 0.228 | 0.273 | -0.174 | 0.405 | 0.176 | 0.400 | 0.206 | 0.324 | 0.443 | 0.026 |
| *Pth1r* | -0.602 | 0.001 | 0.214 | 0.303 | -0.278 | 0.179 | -0.617 | 0.001 | -0.517 | 0.008 |
| *Pvr* | 0.768 | <0.001 | -0.266 | 0.199 | 0.424 | 0.035 | 0.648 | <0.001 | 0.760 | <0.001 |
| *Pxdn* | 0.676 | <0.001 | -0.323 | 0.115 | 0.402 | 0.046 | 0.591 | 0.002 | 0.582 | 0.002 |
| *Pzp* | 0.214 | 0.303 | -0.174 | 0.405 | 0.175 | 0.402 | 0.193 | 0.355 | 0.422 | 0.036 |
| *Rarres2* | 0.734 | <0.001 | -0.540 | 0.005 | 0.708 | <0.001 | 0.653 | <0.001 | 0.813 | <0.001 |
| *Rdh11* | -0.591 | 0.002 | 0.089 | 0.673 | -0.185 | 0.375 | -0.557 | 0.004 | -0.450 | 0.024 |
| *Rdh7* | 0.227 | 0.276 | -0.177 | 0.396 | 0.180 | 0.389 | 0.203 | 0.330 | 0.431 | 0.031 |
| *Rnase13* | -0.092 | 0.661 | -0.062 | 0.767 | -0.066 | 0.752 | 0.077 | 0.716 | -0.217 | 0.299 |
| *Ros1* | -0.247 | 0.235 | -0.031 | 0.883 | -0.092 | 0.660 | -0.120 | 0.566 | -0.304 | 0.140 |
| *Rtn4rl1* | 0.760 | <0.001 | -0.469 | 0.018 | 0.535 | 0.006 | 0.719 | <0.001 | 0.729 | <0.001 |
| *Sema4c* | 0.633 | 0.001 | -0.465 | 0.019 | 0.461 | 0.020 | 0.593 | 0.002 | 0.626 | 0.001 |
| *Serpina1a* | 0.220 | 0.291 | -0.175 | 0.404 | 0.177 | 0.398 | 0.195 | 0.349 | 0.423 | 0.035 |
| *Serpina1b* | 0.226 | 0.278 | -0.174 | 0.406 | 0.174 | 0.404 | 0.201 | 0.336 | 0.430 | 0.032 |
| *Serpina1c* | 0.214 | 0.305 | -0.172 | 0.410 | 0.172 | 0.410 | 0.192 | 0.357 | 0.422 | 0.036 |
| *Serpina1d* | 0.225 | 0.279 | -0.176 | 0.400 | 0.180 | 0.390 | 0.200 | 0.339 | 0.430 | 0.032 |
| *Serpina1e* | 0.208 | 0.318 | -0.171 | 0.413 | 0.170 | 0.418 | 0.189 | 0.367 | 0.418 | 0.038 |
| *Serpinc1* | 0.224 | 0.282 | -0.176 | 0.400 | 0.181 | 0.386 | 0.199 | 0.341 | 0.429 | 0.033 |
| *Serpinf1* | 0.421 | 0.036 | -0.452 | 0.023 | 0.547 | 0.005 | 0.427 | 0.033 | 0.702 | <0.001 |
| *Sfrp5* | 0.650 | <0.001 | -0.381 | 0.060 | 0.542 | 0.005 | 0.554 | 0.004 | 0.550 | 0.004 |
| *Slc25a16* | -0.362 | 0.076 | 0.197 | 0.344 | -0.356 | 0.080 | -0.349 | 0.087 | -0.440 | 0.028 |
| *Slc27a1* | -0.579 | 0.002 | 0.554 | 0.004 | -0.504 | 0.010 | -0.637 | 0.001 | -0.583 | 0.002 |
| *Slc9a2* | -0.328 | 0.109 | 0.016 | 0.940 | -0.179 | 0.392 | -0.123 | 0.557 | -0.293 | 0.155 |
| *Smo* | 0.520 | 0.008 | -0.552 | 0.004 | 0.678 | <0.001 | 0.519 | 0.008 | 0.640 | 0.001 |
| *Smoc2* | 0.352 | 0.085 | -0.382 | 0.059 | 0.505 | 0.010 | 0.417 | 0.038 | 0.655 | <0.001 |
| *Sorl1* | -0.702 | <0.001 | 0.441 | 0.027 | -0.487 | 0.014 | -0.721 | <0.001 | -0.596 | 0.002 |
| *Spag11b* | -0.238 | 0.252 | -0.033 | 0.875 | -0.081 | 0.700 | -0.125 | 0.552 | -0.280 | 0.175 |
| *Spink12* | -0.113 | 0.591 | -0.052 | 0.806 | -0.075 | 0.723 | 0.053 | 0.803 | -0.217 | 0.297 |
| *Spink2* | -0.073 | 0.730 | -0.034 | 0.871 | -0.088 | 0.677 | 0.114 | 0.587 | -0.186 | 0.375 |
| *Spink5* | -0.134 | 0.523 | -0.046 | 0.825 | -0.077 | 0.715 | 0.026 | 0.901 | -0.223 | 0.284 |
| *Spock1* | -0.197 | 0.346 | -0.044 | 0.835 | -0.084 | 0.689 | -0.054 | 0.796 | -0.275 | 0.184 |
| *Taok3* | 0.597 | 0.002 | -0.464 | 0.019 | 0.532 | 0.006 | 0.609 | 0.001 | 0.728 | <0.001 |
| *Tfpi* | 0.549 | 0.004 | -0.398 | 0.049 | 0.488 | 0.013 | 0.553 | 0.004 | 0.678 | <0.001 |
| *Tgfb3* | 0.390 | 0.054 | -0.496 | 0.012 | 0.616 | 0.001 | 0.414 | 0.040 | 0.601 | 0.001 |
| *Thbd* | 0.678 | <0.001 | -0.410 | 0.042 | 0.560 | 0.004 | 0.673 | <0.001 | 0.716 | <0.001 |
| *Tril* | 0.593 | 0.002 | -0.393 | 0.052 | 0.454 | 0.023 | 0.587 | 0.002 | 0.616 | 0.001 |
| *Ugt3a2* | 0.255 | 0.220 | -0.198 | 0.342 | 0.199 | 0.340 | 0.229 | 0.271 | 0.446 | 0.025 |
| *Unc5a* | -0.536 | 0.006 | 0.182 | 0.384 | -0.256 | 0.216 | -0.558 | 0.004 | -0.495 | 0.012 |
| *Vasn* | 0.568 | 0.003 | -0.482 | 0.015 | 0.636 | 0.001 | 0.619 | 0.001 | 0.805 | <0.001 |
| *Vegfc* | 0.504 | 0.010 | -0.338 | 0.098 | 0.441 | 0.028 | 0.518 | 0.008 | 0.683 | <0.001 |
| *Vtn* | 0.263 | 0.203 | -0.198 | 0.343 | 0.204 | 0.327 | 0.232 | 0.265 | 0.473 | 0.017 |
